# Extensive Studies on the Synthesis and Characterization of *π*‐Arene Chromium Complexes and Their Performance in S_N_Ar and Suzuki–Miyaura Cross‐Coupling Reactions

**DOI:** 10.1002/asia.202500139

**Published:** 2025-04-25

**Authors:** Clemens Maurer, Ruben Fleischer, Anup Mandal, Kavin Raj Kumar Chandramohan, Fabian Christophe Herpell, Christopher Heinz Köhler, Defne Cetin, Gregor Schnakenburg, Ala Bunescu

**Affiliations:** ^1^ Kekulé Institute of Organic Chemistry and Biochemistry University of Bonn Gerhard‐Domagk‐Straße 1 Bonn 53121 Germany; ^2^ Institute of Inorganic Chemistry University of Bonn Gerhard‐Domagk‐Straße1 Bonn 53121 Germany

**Keywords:** Densely functionalized polyaryl complexes, Full spectroscopic characterization, Organometallic synthesis, Suzuki–Miyaura cross‐coupling, *π*‐Arene chromium complexes

## Abstract

We report an efficient synthesis of a series of new (η^6^‐arene)Cr(CO)_3_ complexes via ligand exchange strategy under thermal conditions using chromium hexacarbonyl and readily accessible arene feedstocks. We optimized the previously reported procedures and found that the high excess of arenes usually employed is not required to access the *π*‐arene chromium complexes in high yields. Consequently, the revised procedure simplifies the purification of chromium tricarbonyl complexes bearing arenes with high boiling points. The reaction is amenable to provide (η^6^‐arene)Cr(CO)_3_ complexes decorated with various functional groups. Notably, the novel and already reported chromium complexes with missing spectroscopic characterization were fully characterized by nuclear magnetic resonance (NMR), infrared (IR) spectroscopies, and high‐resolution mass spectrometry (HRMS). Besides, we report the structure of 20 chromium complexes characterized via X‐ray crystallography. The potential application of these *π*‐arene chromium complexes has also been exploited toward the selective construction of poly(hetero)aryls possessing a single chromium tricarbonyl unit through S_N_Ar and Suzuki–Miyaura cross‐coupling reactions. This work is meant to be a practical guide for the synthesis of *π*‐arene chromium complexes and to fill the gap in their spectroscopic characterization.

## Graphical abstract

*π*‐Arene chromium complexation: An advancement in the direct thermolysis approach has been demonstrated, showcasing its broad applicability with over 50 examples, including heterocyclic ring‐tethered and densely functionalized poly(hetero)aryl Cr(CO)₃ complexes, achieving very high yields.

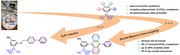

## 1 Introduction

Arenes are ubiquitous motifs in organic chemistry, playing pivotal roles in the pharma, agrochemical, and plastic industries.^[^ ^1^ ^]^ Aromatic compounds display distinctive bonding features, have remarkable stability due to the conjugated system, and are characterized by rich and intricate chemistry. One noteworthy reactivity of arenes is the π‐coordination with transition metals that induces drastic changes in chemical properties, providing new opportunities for the functionalization of aromatic systems.^[^ ^2^ ^]^ This change in reactivity is strongly dependent on the nature of the metal and its supporting ligands, which offers an additional dimension to tune and control the reactivity of the arenes. A well‐explored coordination mode between arenes and transition metals is the η^6^‐complexation, which gained tremendous attention in synthetic organic chemistry, as it facilitates transformations that are otherwise unachievable with the corresponding uncomplexed substrates.^[^ ^3^ ^]^ Numerous tractable *π*‐arene complexes derived from various transition metals, such as Cr, Mn, Fe, Ru, among others, are well‐known in the literature.^[^ ^2^, ^3^, ^4^ ^]^ However, among them, (η^6^‐arene)Cr(CO)_3_ complexes are one of the most valuable and investigated moieties employed in organic synthesis.

**Scheme 1:**
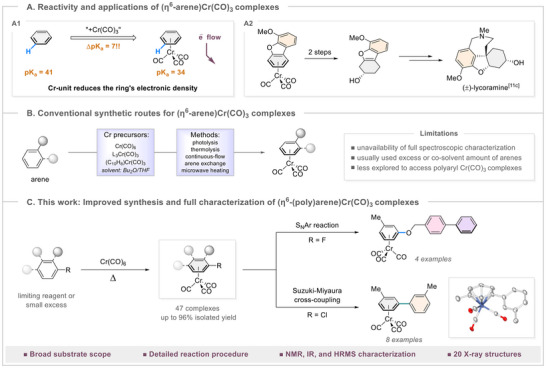
Enhanced synthetic strategy and comprehensive characterization of the (η^6^‐arene)Cr(CO)_3_ complexes.

The extensive use of the chromium π‐arene tricarbonyl complexes can be attributed to numerous key factors, as they are air‐stable, often crystalline, and the chromium tricarbonyl fragment can be readily removed after the desired transformation with oxidizing agents or light irradiation, making them robust and versatile synthetic intermediates.^[^ ^4^ ^]^ Most importantly, the coordination of the chromium tricarbonyl tripod to the arene moiety dramatically influences its reactivity, primarily through its strong electron‐withdrawing effect, which enhances the acidity of both aromatic and benzylic C─H bonds, significantly altering the chemical behavior of the aromatic system (Scheme 1A1).^[^ ^5^ ^]^ Thanks to remarkable influences exerted on the properties of the aromatic ring by the chromium tricarbonyl unit, (η⁶‐arene)chromium complexes have been employed in many types of transformations such as transition metal‐catalyzed C─H functionalization,^[^ ^6^ ^]^ phase‐transfer‐catalyzed asymmetric S_N_Ar reactions,^[^ ^7^ ^]^ enantiospecific C(sp^2^)–C(sp^3^) cross‐couplings,^[^ ^8^ ^]^ and asymmetric relay syntheses,^[^ ^9^ ^]^ including the construction of natural products, dendrimers, and polymers.^[^ ^3^, ^10^ ^]^ Furthermore, the *π‐*arene chromium tricarbonyl complexes have been widely utilized for arene dearomatization reactions, enabling direct access to useful bioactive molecular scaffolds (Scheme 1A2).^[^ ^11^ ^]^

However, the preparation of (η⁶‐arene)chromium complexes continues to pose significant challenges in their investigation. Fischer and Öfele first reported the synthesis of tricarbonyl (η⁶‐benzene)chromium, the first arene tricarbonylchromium complex, in 1957.^[^ ^12^ ^]^ Since then, a plethora of (arene)tricarbonylchromium(0) complexes have been synthesized over the decades (Scheme 1B).^[^ ^3^, ^4^, ^13^ ^]^ The predominant method to access (η⁶‐arene)Cr(CO)_3_ complexes involves the thermolysis of hexacarbonylchromium(0) in an inert condition, generally using a large excess of arene (2–50 equiv) or as a cosolvent, with di *n*‐butyl ether and tetrahydrofuran being the most common solvent combination.^[^ ^3^, ^4^, ^14^ ^]^ This operation very often requires prolonged reaction times, ing in suboptimal yields. To mitigate these issues, various complexation reagents have been introduced, including (η⁶‐naphthalene)Cr(CO)_3_, (NH_3_)_3_Cr(CO)_3_, (MeCN)_3_Cr(CO)_3_, and (pyridine)_3_Cr(CO)_3_, although their application requires an extra synthetic step from hexacarbonylchromium(0).^[^ ^3b^ ^]^ Developing a direct reaction between arene and chromium hexacarbonyl with a small excess of arene or arene as a limiting reagent could significantly enhance atom‐ and step‐economy. The large excess of arenes that is usually employed in the synthesis of *π*‐arene chromium complexes becomes a serious liability with arenes that are solid, have similar polarity as the chromium complex, or display a high boiling point, since the purification turns out to be much more challenging. We hypothesized that ligand exchange reactions under thermal conditions between chromium hexacarbonyl and arene as a limiting reagent or a smaller excess have the potential to provide an alternative and more practical route toward (η⁶‐arene)Cr(CO)_3_ complexes. Despite advancements in synthetic methods, a major hurdle for the practitioner in the field remains the lack of comprehensive spectroscopic data for the thorough characterization of (η⁶‐arene)Cr(CO)_3_ complexes.^[^ ^15^ ^]^ Most of the literature known complexes reported in this manuscript, published before 2000, have primarily been characterized by ¹H NMR or IR spectroscopy, which limits the depth of their characterization.^[^ ^16^ ^]^ This realization was surprising given the extensive literature reports on the reactivity and synthetic applications of *π*‐arene chromium complexes. Because of this lack or only partial analytical data for this important class of compounds and our recent interest in the application of *π*‐arene chromium complexes, we decided to report our findings and extensive studies on the synthesis and characterization of (η⁶‐arene)Cr(CO)_3_ complexes under optimized condition comparing with the previously reported one.

In this study, we report the advancement of this direct thermolysis approach, demonstrating the wide‐spreading applicability with over 40 examples, and yields up to 96%, along with full characterization by NMR, IR, HRMS, and X‐ray crystallographic analysis (Scheme 1C). Notably, this straightforward protocol is scalable to multigram quantities, accommodates various functional groups, and facilitates the formation of heterocyclic ring‐tethered Cr(CO)_3_ complexes and densely functionalized poly(hetero)aryl Cr(CO)_3_ complexes through nucleophilic aromatic substitution (S_N_Ar) and Suzuki–Miyaura cross‐coupling reactions, respectively.

## 2 Results and Discussion

**Scheme 2:**
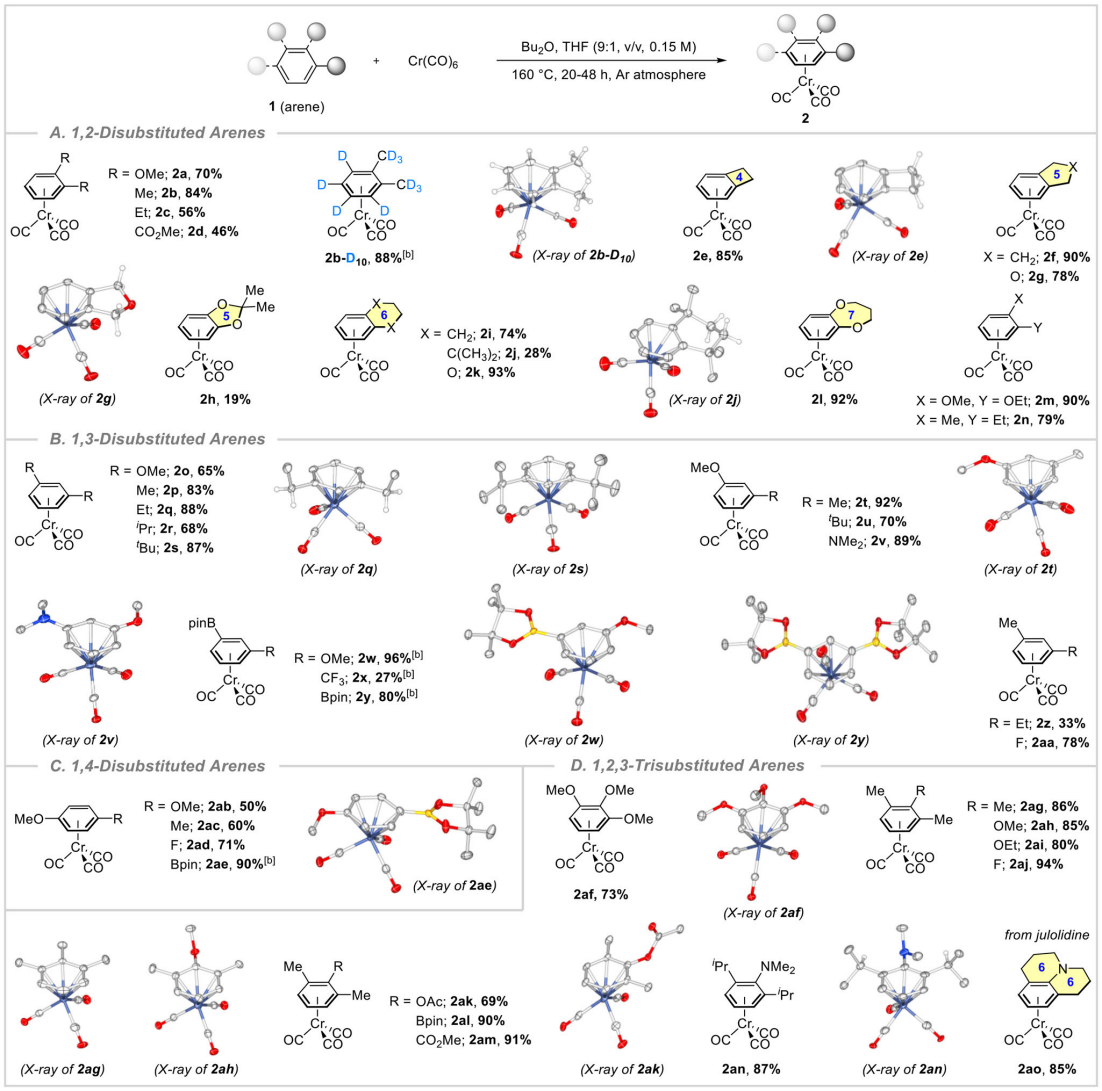
Scopes of the *π*‐arene chromium complexation with Cr(CO)_6_ and a variety of arenes. (a) Reaction conditions: Cr(CO)_6_ (1.0 equiv), arene (1.0–1.2 equiv), Bu_2_O/THF (9:1, v/v, 0.15 M) for 20–48 h at 160 °C under Ar. Isolated yields are given. (b) Arene (1.0 equiv) and Cr(CO)_6_ (1.5 equiv) are used. Aromatic and methyl C─H bonds are omitted in the crystal structures for clarity. See Supporting Information for detailed reaction conditions for individual substrates.

At the beginning of our study, we focused on the synthesis of (1,2‐dimethoxybenzene)Cr(CO)_3_ complex. To our delight, when the mixture of Cr(CO)_6_ (1.0 equiv, 10.0 mmol) and 1,2dimethoxybenzene (1.0 equiv, 10.0 mmol) in Bu_2_O/THF (9 : 1, v/v, 0.15 M) solvent was heated at 160 °C for 24 h under a positive argon atmosphere, the *π*‐arene complexation proceeded smoothly, providing the desired product **2a** in 70% isolated yield (Scheme 2). Using the 1:1 ratio of Cr(CO)_6_ versus arene as the suitable atom‐economical reaction conditions for thermolysis, a variety of substituted arenes, irrespective of their steric and electronic properties, can also be accommodated to give a fruitful transformation to their corresponding (ƞ^6^‐arene)Cr(CO)_3_ complexes (Scheme 2). Notably, 1,2‐dialkylbenzene derivatives effectively participated in these reaction conditions, resulting in products **2b** and **2c** with yields ranging from 56%–84%. Interestingly, the developed method worked well with electron‐poor dimethyl phthalate and furnished the (ƞ^6^‐dimethylphthalate)Cr(CO)_3_ complex **2d** in moderate 46% yield. Next, we introduced 1,2‐fused/disubstituted arenes, such as four‐membered ring fused benzocyclobutene, five‐membered ring fused indane, phthalane, 2,2‐dimethyl‐1,3‐benzodioxole, six‐membered ring fused 1,2,3,4‐tetrahydronaphthalene, 1,1,4,4‐tetramethyl‐1,2,3,4tetrahydronaphthalene, benzo‐1,4‐dioxane and seven‐membered ring fused 3,4‐dihydro‐2*H*‐1,5‐benzodioxepine, under the similar conditions and they efficiently reacted with Cr(CO)_6_, affording the (1,2‐fused/disubstituted arene)Cr(CO)_3_ complexes **2e**–**l** in synthetically useful yields (19%–93%). 1,2‐Disubstituted unsymmetrical arenes, such as 1‐ethoxy‐2‐methoxybenzene and 2‐ethyltoluene, were also effortlessly transformed to chromium tricarbonyl *π*‐complexed arenes **2m** and **2n** in high yields (79%–90%). We further explored the scope of chromium tricarbonyl *π*‐complexation with 1,3‐disubstituted arenes, with the reaction outcomes summarized in Scheme 2B. Symmetrical 1,3‐disubstituted arenes as well as unsymmetrical arenes possessing various electron‐withdrawing functional groups (─Bpin, ─CF_3_, ─F) furnished the half‐sandwich Cr(CO)_3_ complexes **2o**–**2z**, **2aa** in 27%–96% yields.

Similarly, the reactions with 1,4‐disubstituted arenes offered the expected chromium complexes **2ab**–**2ae** in good yields (50%–90%; Scheme 2C). Moreover, densely functionalized 1,2,3‐trisubstituted arenes were also suitable for this transformation and provided (ƞ^6^‐1,2,3‐trisubstitutedarene)Cr(CO)_3_ complexes **2af**–**2ao** in good to excellent yields (69%–94%). A wide variety of functional groups, such as fluoro (**2aj**), acetoxy (**2ak**), Bpin (**2al**), ester (**2am**), and ‐*N*,*N*‐dimethylamine (**2an**), were well‐tolerated under the present protocol (Scheme 2D). The current synthetic approach was successfully applied to julolidine, a complex heterocyclic aromatic compound, and the corresponding 2,3,6,7‐tetrahydro‐1*H*,5*H*‐pyrido[3,2,1‐*ij*]quinoline chromium tricarbonyl **2ao** was obtained in 85% isolated yield. Pleasingly, when the reaction was performed with *o*‐xylene‐d_10_, fully deuterated *o‐*xylene chromium tricarbonyl (**2b**‐**D_10_**) was easily formed in 88% yield. Noteworthy, in the case of deuterium and ‐Bpin substituted arenes (**1d‐D_10_**, **1w**–**y**, **1ae**), the arene was wisely chosen as limiting reagent using 1.5 equivalents of Cr(CO)_6_ to obtain the valuable products **2b**‐**D_10_**, **2w**–**y**, and **2ae**. The comparatively decreased yields in some cases can be attributed to the oxidative decomposition of the products during the course of thermolysis or column chromatography on silica gel.^[^ ^17^ ^]^ It is very important to mention that all the synthesized (ƞ^6^‐arene)Cr(CO)_3_ complexes are thoroughly characterized by ^1^H, ^13^C NMR (also ^19^F NMR, F‐containing compound), IR, and HRMS techniques. Furthermore, the structural conformation of sixteen chromium tricarbonyl complexes (**2b**‐**D_10_**, **2e**, **2** **g**, **2j**, **2q**, **2s**, **2t**, **2v**, **2w**, **2y**, **2ae**, **2af**, **2ag**, **2ah**, **2ak**, **2an**) was unambiguously confirmed through X‐ray analysis.^[^ ^18^ ^]^ The limitation of this protocol is discussed in the Supporting Information.

**Scheme 3:**
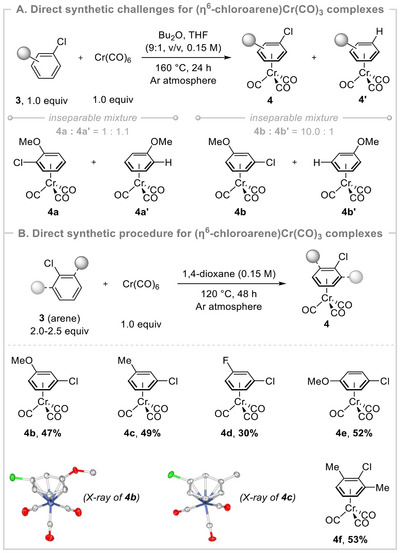
Direct synthetic approaches for (ƞ^6^‐chloroarene)Cr(CO)_3_ complexes. See Supporting Information for detailed reaction conditions. **4a′** = **4b′**.

The efficacy of this method inspired us to investigate the feasibility of direct *π*‐arene complexation with aryl chlorides, as the C─Cl bond often serves as a useful synthetic handle in various postsynthetic modifications to access complex molecular motifs. Accordingly, the combination of 2‐chloroanisole (1.0 equiv, 3.0 mmol) and Cr(CO)_6_ (1.0 equiv, 3.0 mmol) was examined under the established reaction conditions at 160 °C for 24 h. Unfortunately, an inseparable mixture of desired (ƞ^6^‐2‐chloroanisole)Cr(CO)_3_ and undesired (ƞ^6^‐anisole)Cr(CO)_3_ complex was obtained. The product ratio (**4a**:**4a′** = 1:1.1) was determined via ^1^H NMR of the crude reaction mixture after evaporation (Scheme 3A; see the Supporting Information for more details). The formation of undesired (ƞ^6^‐anisole)Cr(CO)_3_ complex may be attributed to hydrodechlorination during the thermolysis.^[^ ^14e^ ^]^ A similar kind of transformation was also observed for 3‐chloroanisole with an improved ratio (**4b**:**4b′** = 10.0:1); however, the isolation of pure product **4b** was still difficult from the mixture. To address this issue, we chose 1,4‐dioxane as a suitable solvent for direct *π*‐arene complexation to retard the unwanted hydrodechlorination step.^[^ ^14a^ ^]^ To our delight, when Cr(CO)_6_ (1.0 equiv) and aryl chlorides (**3**, 2.0–2.5 equiv) were exposed in 1,4‐dioxane at 120 °C, *π*‐arene complexation proceeded smoothly without formation of any hydrodechlorination side product to deliver (ƞ^6^‐arylchloride)Cr(CO)_3_ complexes (Scheme 3B). Although the reaction progressed slowly (generally requiring 48 h), the reaction with the electron‐rich aryl chlorides was effective in delivering the targeted pure products (**4b**–**c**, **4e**–**f**) in 47%–53% isolated yields. Comparatively, a low yield of 30% product (**4d**) was acquired for electron‐deficient 1‐chloro‐3‐fluorobenzene.

**Scheme 4:**
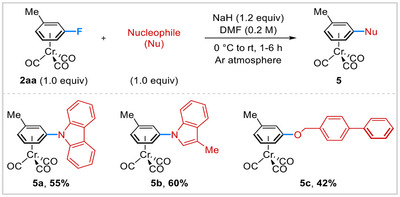
Synthesis of heterocyclic‐hinged (ƞ^6^‐arene)Cr(CO)_3_ complexes through S_N_Ar reaction. See Supporting Information for detailed reaction conditions.

The chromium tricarbonyl tripod coordinated to the arene acts as an electron‐withdrawing group (EWG) and increases the electrophilic character of the arene ring and thus facilitates the direct addition of the nucleophile to the aromatic ring.^[^ ^19^ ^]^ Taking advantage of this reactivity, we subjected (ƞ^6^‐fluoroarene)Cr(CO)_3_ complex to the reaction with various N‐ and O‐centered nucleophiles to access heterocyclic‐hinged (ƞ^6^‐arene)Cr(CO)_3_ complexes employing comparable conditions developed by Perez^[^ ^19e^ ^]^ and Maiorana^[^ ^19g^ ^]^ (Scheme 4). Similar to the reaction proceeding via aromatic nucleophilic substitution (S_N_Ar), (ƞ^6^‐3‐fluorotoluene)chromium tricarbonyl (**2aa**) rapidly reacted with 1*H*‐carbazole, 3‐methylindole, and 4‐phenylbenzyl alcohol using NaH base in DMF at 0 °C to room temperature, providing the desired chromium tricarbonyl complexes **5a**–**c** in moderate to good yields.

**Scheme 5:**
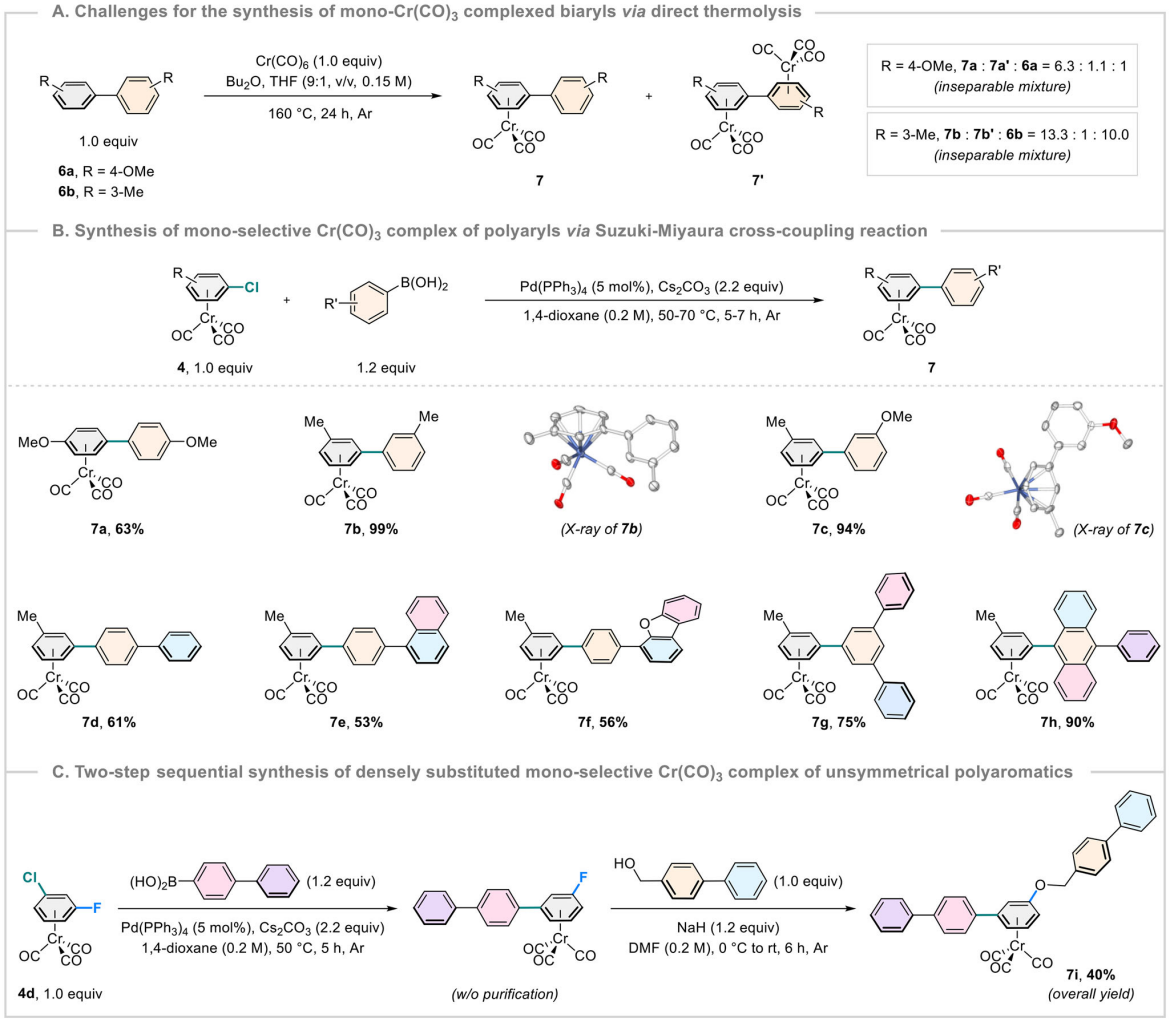
Synthetic challenges and development to access the mono‐selective Cr(CO)_3_ complex of polyaryls.

Biphenyl motifs are very appealing not only in asymmetric transformation as chiral ligands, but they are also often found in bioactive molecules and natural products.^[^ ^20^ ^]^ In this context, coordination of a chromium tricarbonyl unit to an arene ring induces planar chirality in ‐meta or ‐ortho disubstituted arenes, guiding to both planar and axial chirality in the monochromium complexes of ‐meta or ‐ortho disubstituted biphenyl compounds, which could be advantageous in asymmetric synthesis.^[^ ^21^ ^]^ Next, we sought to synthesize the mono‐Cr(CO)_3_ complex of biaryl derivatives with the utilization of previously established reaction conditions for direct *π*‐arene complexation of biaryls (Scheme 5A). Regrettably, the thermolysis of 4,4′‐dimethoxy‐1,1′‐biphenyl (**6a**, 1.0 equiv) and Cr(CO)_6_ (1.0 equiv) was not fruitful at all and ended up with the formation of inseparable mono as well as bis‐Cr(CO)_3_ complexes of biaryl (**7a** and **7a′**).^[^ ^22^ ^]^ The ratio of products and starting arene (**7a**:**7a′**:**6a **= 6.3:1.1:1) was determined via ^1^H NMR of the evaporated crude reaction mixture (see Supporting Information for more details).

The reaction of 3,3′‐dimethyl‐1,1′‐biphenyl (**6b**) also followed the same trend with the production of inseparable mono and bis‐Cr(CO)_3_ complexes of biaryl (**7b** and **7b′**) with starting biphenyl **6b** (ratio = **7b**:**7b′**:**6b **= 13.3:1:10.0; Scheme 5A). Since the construction of mono‐selective Cr(CO)_3_ complex of biaryls is difficult and often results in complex mixtures, an elegant route to access them with high selectivity would be Suzuki–Miyaura cross‐coupling reaction via direct coupling of (ƞ^6^‐chloroarene)Cr(CO)_3_ with diverse (hetero)aryl boronic acids.^[^ ^23^ ^]^ The palladium‐catalyzed cross‐coupling reactions of (ƞ^6^‐arylchloride)Cr(CO)_3_ and (ƞ^6^‐arylbromide)Cr(CO)_3_ with arylboronic acid were previously reported by several research groups, although the presented scope is very limited.^[^ ^24^ ^]^ An alternative cross‐coupling strategy was developed by the Widdowson group involving the Pd‐catalyzed reaction between (ƞ^6^‐arylfluoride)Cr(CO)_3_ and arylboronic acid.^[^ ^25^ ^]^ A complementary approach to access the Cr(CO)_3_ mono‐complexed biaryls consists of the direct Pd‐catalyzed *ortho*‐C–H arylation of (ƞ^6^‐arylfluoride)Cr(CO)_3_ and (ƞ^6^‐anisole)Cr(CO)_3_ complexes with iodoarene, developed by Larrosa.^[^ ^6^ ^]^ Based on these important precedents we conducted the reaction between 4‐chloroanisole chromium tricarbonyl (**4e**, 1.0 equiv) and (4‐methoxyphenyl)boronic acid (1.2 equiv) in the presence of Cs_2_CO_3_ (2.2 equiv), Pd(PPh_3_)_4_ (5 mol%) in dry 1,4‐dioxane for 5 h at 50 °C.^[^ ^26^ ^]^ Remarkably, the mono‐selective Cr(CO)_3_ complex of biaryl product **7a** was isolated in 63% yield (Scheme 5B). Later, mono‐selective Cr(CO)_3_ complex of 3,3′‐dimethyl‐1,1′‐biphenyl **7b** was secured with an excellent yield of 99% from (3‐chlorotoluene)Cr(CO)_3_ complex **4c** under the same reaction conditions. This method is quite general. A broad range of aryl and poly(hetero)aryl boronic acids encompassing methoxybenzene, biphenyl, phenylnaphthalene, phenyldibenzo[*b*,*d*]furan, terphenyl, phenylanthracene scaffolds were engaged smoothly, delivering the complex molecular architecture of mono‐selective Cr(CO)_3_ complex of poly(hetero)aryls (**7c**–**h**) in good to excellent yields (53%–94%).Complexes **7b** and **7c** were crystallized, and the bonding connectivity was explicitly established by X‐ray analysis.^[^ ^18^ ^]^ Further, the utility of this transformation was extended through a two‐step sequential synthesis of **7i**, a densely substituted mono‐selective Cr(CO)_3_ complex of unsymmetrical polyaromatics (Scheme 5C). We believe that the intrinsic behavior of the chromium tricarbonyl unit could be beneficial for the selective transformation/functionalization of the polyarene rings, complexed with a single Cr(CO)_3_ unit, and open a new avenue in modern organic synthesis.

## 3 Conclusion

We improved the synthesis of various (η^6^‐arene)chromium tricarbonyl complexes (47 examples), using commercially available arene feedstocks, which are hitherto unknown or reported (<10%) with appropriate spectroscopic characterization data.^[^ ^27^, ^28^ ^]^ This protocol is simple, generally permits up to multigram scale, accommodates different common functional groups, including fluoro, chloro, acetoxy, Bpin, ester, dimethylamine, and delivers the valuable *π*‐arene chromium complexes in 19%–96% isolated yields. The challenges associated with the selective synthesis of mono‐Cr(CO)_3_ complex of biaryls directly from arene have also been depicted. The (ƞ^6^‐arene)Cr(CO)_3_ complexes have been further employed to build heterocyclic ring‐tethered (arene)Cr(CO)_3_ complexes through nucleophilic aromatic substitution (S_N_Ar) reaction (3 examples) and densely functionalized poly(hetero)aryl complexes embracing with single Cr(CO)_3_ unit via Suzuki–Miyaura cross‐coupling reactions (8 examples). The efficiency of this protocol has also been demonstrated for the expedient construction of the unsymmetrical complex molecular framework **7i** in a two‐step sequential fashion. Finally, we anticipate that the unique properties of the chromium tricarbonyl tripod, attached to a single arene ring of polyarene, could enhance the selective transformation and functionalization of the (ƞ^6^‐(poly)arene)Cr(CO)_3_ complex, paving innovative pathways in the synthetic method development.

## 4 Experimental Section

General procedure for the synthesis of (η^6^‐arene)Cr(CO)_3_ complexes with Cr(CO)_6_ and corresponding arenes under direct thermolysis: An oven‐dried round‐bottom flask equipped with a magnetic stir bar was charged with Cr(CO)_6_ (2.0–10.0 mmol, 1.0 equiv) and corresponding arene (1.0–2.0 equiv), evacuated, and backfilled with argon. Then, dibutyl ether and THF (9:1, v/v, 0.15 M) were added with the syringe, and the resulting mixture was degassed for 20–30 min. After that, an oven‐dried reflux condenser was connected to the round‐bottom flask under a positive argon atmosphere. The reaction suspension was subjected to three freeze‐pump‐thaw cycles and then refluxed at 160 °C for the specified reaction time. After completion of the reaction, the reaction mixture was cooled down to room temperature and filtered over a short pad of silica gel or celite (rinsed with EtOAc three times). The combined reaction mixture was concentrated under reduced pressure with the aid of a rotary evaporator. The crude residue was then purified by flash column chromatography to provide the arene chromium tricarbonyl complexes (2a–v, 2z, 2aa–ad, 2af–ao).

## Conflict of Interests

The authors declare no conflict of interest.

Deposition Numbers 2393581 (for **2b‐D10**), 2392930 (for **2e**), 2392931 (for **2** **g**), 2392932 (for **2j**), 2392933 (for **2q**), 2392934 (for **2s**), 2392935 (for **2t**), 2392936 (for **2v**), 2392937 (for **2w**), 2392938 (for **2y**), 2392939 (for **2ae**), 2392940 (for **2af**), 2392941 (for **2ag**), 2392942 (for **2ah**), 2392943 (for **2ak**), 2392944 (for **2an**), 2392945 (for **4b**), 2409305 (for **4c**), 2392946 (for **7b**), 2392947 (for **7c**), 2392948 (for **8**) contain(s) the supplementary crystallographic data for this paper. These data are provided free of charge by the joint Cambridge Crystallographic Data Center and Karlsruhe Specialized Information Center Access Structures service.

## Supporting information



Supporting Information

## Data Availability

The data that support the findings of this study are available in the supplementary material of this article.
