# Supplementary material for: Extensive Studies on the Synthesis and Characterization of π‐Arene Chromium Complexes and Their Performance in SNAr and Suzuki–Miyaura Cross‐Coupling Reactions
Source: Chem Asian J. 2025 Apr 25;20(12):e202500139. doi: 10.1002/asia.202500139 (PMC12204384; doi:10.1002/asia.202500139)
Supplement: Supplementary file 1 — Supporting Information [file ASIA-20-e202500139-s001.pdf]

Extensive Studies on the Synthesis and Characterization of *π*-Arene Chromium Complexes and Their Performance in S$_N$Ar and Suzuki-Miyaura Cross-Coupling Reactions

Clemens Maurer,[‡a] Ruben Fleischer,[‡a] Anup Mandal,[‡*a] Kavin Raj Kumar Chandramohan,[a] Christopher Heinz Köhler,[a] Fabian Christophe Herpell,[a] Defne Cetin,[a] Gregor Schnakenburg,[b] and Ala Bunescu*[a]

[a]Kekulé Institute of Organic Chemistry and Biochemistry, University of Bonn, Gerhard-Domagk-Straße 1, 53121 Bonn, Germany

[b]Institute of Inorganic Chemistry, University of Bonn, Gerhard-Domagk-Straße 1, 53121 Bonn, Germany

[‡]These authors contributed equally to this work.

Email: amandal@uni-bonn.de;
ala.bunescu@uni-bonn.de

Table of Contents

1. General Information S3

2. Synthesis and Characterization of (η^6-Arene)$Cr(CO)_3$ Complexes S5

2.1. General procedure for the synthesis of (η^6-arene)$Cr(CO)_3$ complexes with $Cr(CO)_6$ and corresponding arenes (GP-A) S5

2.2. General procedure for the synthesis of (η^6-arene)$Cr(CO)_3$ complexes with $Cr(CO)_6$ and corresponding arenes (GP-B) S6

2.3. General procedure for the synthesis of (η^6-arene)$Cr(CO)_3$ complexes with $Cr(CO)_6$ and corresponding aryl chlorides (GP-C) S6

2.4. General procedure for the synthesis of (η^6-arene)$Cr(CO)_3$ complexes via nucleophilic aromatic substitution (S_NAr) reaction (GP-D) S7

2.5. General procedure for the synthesis of (η^6-arene)$Cr(CO)_3$ complexes via Suzuki-Miyaura cross-coupling reaction (GP-E) S8

2.6. Characterization of (η^6-arene)$Cr(CO)_3$ complexes S10

2.7. Unsuccessful substrates *via* direct π-arene complexation S46

2.8. Removal of chromium tricarbonyl fragment from the *π*-arene complex through light irradiation (GP-F) S51

3. X-Ray Crystallographic Data S54

4. NMR Spectra of (η^6-Arene)$Cr(CO)_3$ Complexes S98

5. References S229

1. General Information

All reactions were carried out under an argon atmosphere with standard Schlenk techniques or inside the glovebox in oven-dried glassware and stirred using a magnetic stir plate. All reactions were conducted using anhydrous solvent unless otherwise noted. Dry 1,4-dioxane and DMF were purchased from Acros Organics. Synthetic grade Dibutyl ether and THF were purchased from Thermo Fisher Scientific. Chromium(0) hexacarbonyl was purchased from Sigma-Aldrich with 98% purity and used under the exclusion of light. Unless otherwise specified, all of the reagents were purchased from commercial suppliers and used without purification. All reactions were monitored by thin-layer chromatography (TLC). Thin-layer chromatography was performed on silica gel coated on aluminum plates, and visualized using a UV lamp (366 or 254 nm) or by use of one of the following visualization reagents: PMA: 10.0 g of phosphomolybdic acid/100.0 mL of ethanol, $KMnO_4$: 0.75 g of potassium permanganate, 5.0 g of K_2CO_3/100.0 mL of water. Products were isolated by flash column chromatography (Merck silica gel 100−200 µm). Reaction yields refer to chromatographically and spectroscopically homogeneous materials unless noted otherwise. ^{1}H, ^{13}C NMR, and ^{19}F spectra were recorded on a Bruker Avance 400 MHz or Bruker Avance 500 MHz spectrometer. Chemical shift values (δ) are reported in ppm and calibrated to the residual solvent peak C_6D_6 δ = 7.16 ppm for ^{1}H, δ = 128.06 ppm for ^{13}C; Acetone-d_6 δ= 2.05 ppm for ^{1}H, δ = 29.84 ppm for ^{13}C. All NMR spectra were recorded at ambient temperature (298 K) unless otherwise noted. ^{1}H NMR spectra are reported as follows: chemical shift (multiplicity, coupling constant, integration). The following abbreviations are used to indicate multiplicities: s, singlet; d, doublet; t, triplet; q, quartet; p = pentet, h = heptet, m, multiplet; dd, doublet of doublet; dt, doublet of triplet; dq, doublet of quartet; td, triplet of doublet; tt, triplet of triplet; dq, doublet of quartet; br, broad; app, apparent. Infrared (IR) spectra were acquired on IRSpirit Shimadzu spectrometer. Frequencies are given in wave numbers (cm^{-1}) and only selected peaks were reported. High resolution mass spectra analysis was performed on a Thermoquest MAT 95 CL instrument (Thermo Finnigan, EI/ESI) or an Orbitrap XL mass spectrometer (Thermo Fisher Scientific, APCI/ESI). X-ray data of the crystals were collected and integrated using a STOE STADIVARI Eulerian 4-circle diffractometer equipped with a low temperature device (100(2)K, Oxford Cryostream 800er series, Oxford Cryosystems) by using Cu-Kα-radiation (λ = 1.54186 Å, ASTIX++-optics) and a PILATUS 200K Pixel detector system. Intensities were measured by fine-slicing φ- and ω-scans and corrected for background, polarization and Lorentz effects. A semi-empirical absorption correction by scaling of reflection intensities with a subsequent spherical absorption correction was performed with LANA.[1] Further data X-Ray diffractions were mounted on a Bruker D8 Venture 4-circle Kappa-diffractometer equipped with a

low-temperature device (100(2)K, Oxford Cryostream 800er series, Oxford Cryosystems) by using Mo-Kα radiation (λ = 0.71073 Å, Helios mirror optics) and a PHOTONIII/C14 CMOS detector system. Intensities were measured by fine-slicing φ- and ω-scans and corrected for background, polarization and Lorentz effects. A semi-empirical absorption correction from equivalent reflections (multi-scan type) was performed by using SADABS.[2]

Abbreviations used: Me: methyl, Ph: phenyl, ^{t}Bu: *tert*-butyl, Ac: acetyl, ^{i}Pr: isopropyl, *n*Bu: *n*-butyl, THF: tetrahydrofuran, DMF: *N*,*N*-dimethylformamide.

2. Synthesis and Characterization of (η^6-Arene)Cr(CO)$_3$ Complexes

2.1. General procedure for the synthesis of (η^6-arene)Cr(CO)$_3$ complexes with Cr(CO)$_6$ and corresponding arenes (GP-A)

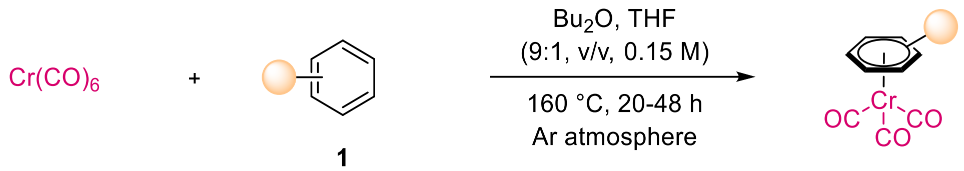

1 (1.0-2.0 equiv) **2**

(2.0-10.0 mmol, 1.0 equiv)

An oven-dried round-bottom flask equipped with a magnetic stir bar was charged with $Cr(CO)_6$ (2.0-10.0 mmol, 1.0 equiv) and corresponding arene (1.0-2.0 equiv), evacuated, and backfilled with argon. Then, dibutyl ether and THF (9:1, v/v, 0.15 M) were added with the syringe and the resulting mixture was degassed for 20-30 minutes. After that, an oven-dried reflux condenser was connected to the round-bottom flask under a positive argon atmosphere. The reaction suspension was subjected to three freeze-pump-thaw cycles and then refluxed at 160 °C for the specified reaction time. After completion of the reaction, the reaction mixture was cooled down to room temperature and filtered over a short pad of silica gel or celite (rinsed with EtOAc for three times). The combined reaction mixture was concentrated under reduced pressure with the aid of a rotary evaporator. The crude residue was then purified by flash column chromatography to provide the arene chromium tricarbonyl complexes (**2a–v, 2z, 2aa–ad, 2af–ao**).

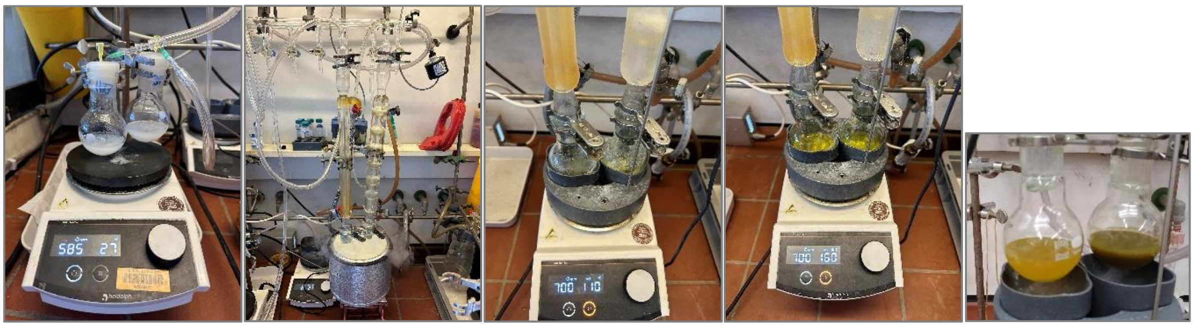

Degassing | freeze-pump-thaw | color changes at 110 °C | at 160 °C | after completion

Figure S1. Representative reaction set-up for the synthesis of (η^6-arene)Cr(CO)$_3$ complexes.

2.2. General procedure for the synthesis of (η^6-arene)$Cr(CO)_3$ complexes with $Cr(CO)_6$ and corresponding arenes (GP-B)

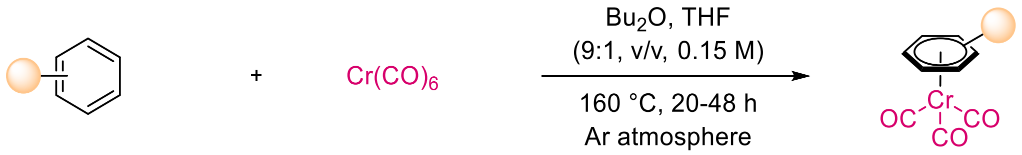

1 (3.0-5.0 mmol, 1.0 equiv) (1.5 equiv) **2**

An oven-dried round-bottom flask equipped with a magnetic stir bar was charged with corresponding arene (3.0-5.0 mmol, 1.0 equiv) and $Cr(CO)_6$ (1.5 equiv), evacuated, and backfilled with argon. Then, dibutyl ether and THF (9:1, v/v, 0.15 M) were added with the syringe and the resulting mixture was degassed for 20-30 minutes. After that, an oven-dried reflux condenser was connected to the round-bottom flask under a positive argon atmosphere. The reaction suspension was subjected to three freeze-pump-thaw cycles and then refluxed at 160 °C for the specified reaction time. After completion of the reaction, the reaction mixture was cooled down to room temperature and filtered over a short pad of silica gel or celite (rinsed with EtOAc for three times). The combined reaction mixture was concentrated under reduced pressure with the aid of a rotary evaporator. The crude residue was then purified by flash column chromatography to provide the arene chromium tricarbonyl complexes (**2b-D$_{10}$, 2w–y, 2ae**).

2.3. General procedure for the synthesis of (η^6-arene)$Cr(CO)_3$ complexes with $Cr(CO)_6$ and corresponding aryl chlorides (GP-C)

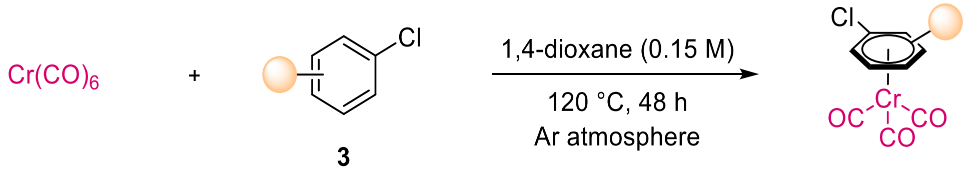

(5.0-8.0 mmol, 1.0 equiv) **3** (2.0-2.5 equiv) **4**

An oven-dried round-bottom flask equipped with a magnetic stir bar was charged with $Cr(CO)_6$ (2.0-10.0 mmol, 1.0 equiv) and corresponding arene (2.0-2.5 equiv), evacuated, and backfilled with argon. Then, 1,4-dioxane (0.15 M) was added with the syringe and the resulting mixture was degassed for 20-30 minutes. After that, an oven-dried reflux condenser was connected to the round-bottom flask under a positive argon atmosphere. The reaction suspension was subjected to four freeze-pump-thaw cycles and then refluxed at 120 °C for

48 h. After completion of the reaction, the reaction mixture was cooled down to room temperature and filtered over a short pad of silica gel or celite (rinsed with EtOAc for three times). The combined reaction mixture was concentrated under reduced pressure with the aid of a rotary evaporator. The crude residue was then purified by flash column chromatography to provide the arene chromium tricarbonyl complexes (**4b–f**).

2.4. General procedure for the synthesis of (η^6-arene)$Cr(CO)_3$ complexes via nucleophilic aromatic substitution (S_NAr) reaction (GP-D)

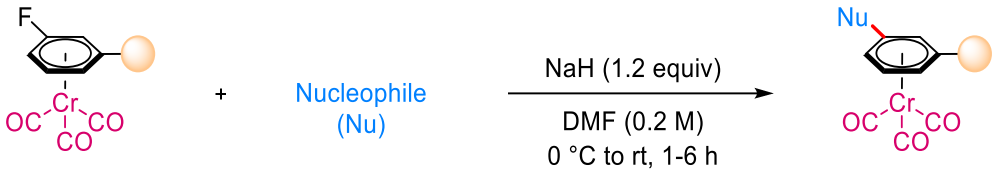

2aa (0.8-1.0 mmol, 1.0 equiv) (1.0 equiv) **5**

An oven-dried screw-capped vial equipped with a magnetic stir bar was charged with NaH (1.2 equiv) and dry DMF. Then a solution of corresponding arene nucleophile (Nu, 1.0 equiv) dissolved in dry DMF was added dropwise to the slurry of NaH at 0 ^oC under argon atmosphere. After 20 min, solution of (η^6-3-fluoroarene)$Cr(CO)_3$ complex in dry DMF was added slowly. The resulting mixture was stirred for 30 minutes and then left at room temperature. After completion of the reaction (TLC monitored), the mixture was then treated with saturated brine solution and extracted with Et_2O (three times). The combined organic layer was washed with water and dried over anhy. Na_2SO_4. The filtrate was concentrated under reduced pressure. The crude residue was then purified by flash column chromatography to provide the desired arene chromium tricarbonyl complex (**5a–c**).

2.5. General procedure for the synthesis of (η^6-arene)$Cr(CO)_3$ complexes via Suzuki-Miyaura cross-coupling reaction (GP-E)

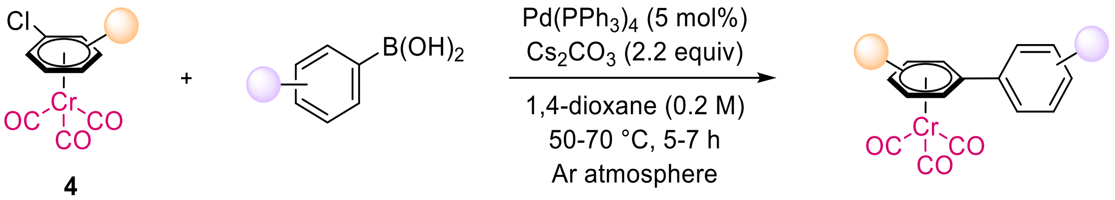

4 (1.0 mmol, 1.0 equiv) (1.2 equiv) **7**

Inside glovebox, an oven-dried screw-capped vial equipped with a magnetic stir bar was charged with corresponding (η^6-chloroarene)$Cr(CO)_3$ complex (1.0 mmol, 1.0 equiv), boronic acid derivative (1.2 equiv), Cs_2CO_3 (2.2 equiv), $Pd(PPh_3)_4$ (5 mol%), and dry 1,4-dioxane (0.2 M). The resulting heterogeneous, yellow-colored mixture was then taken out from the glovebox and allowed to stir at 50-70 °C for the specified reaction time. After completion of the reaction (TLC or ^{1}H NMR monitored), the reaction mixture was cooled down to room temperature and filtered over a short pad of silica gel or celite (rinsed with EtOAc for three times). The combined reaction mixture was concentrated under reduced pressure. The crude residue was then purified by flash column chromatography to provide the arene chromium tricarbonyl complex (**7a–i**).

Note S1 – on preparation, purification, and handling the (η^6-arene)$Cr(CO)_3$ complexes:

1) π-Arene complexation reactions were carried out under reflux in the dark, with the reaction vessels wrapped in aluminum foil, and under a positive argon atmosphere.

2) Under reflux conditions, the reaction should be stopped immediately if black or greenish decomposition appears, to prevent rapid product degradation.

3) Although (η^6-arene)$Cr(CO)_3$ complexes are stable in the solid state and can be stored for extended periods under aerial conditions, they exhibited lower stability on silica gel or in solution. To minimize decomposition, target compounds were rapidly isolated using flash column chromatography on silica gel. Despite purification, the products gradually decomposed over time when stored in NMR tubes for spectroscopic analysis.

4) (η^6-Arene)$Cr(CO)_3$ complexes incorporating polyaromatic systems were particularly sensitive to polar solvents and light. Special precautions were required for the synthesis and handling of these complexes.

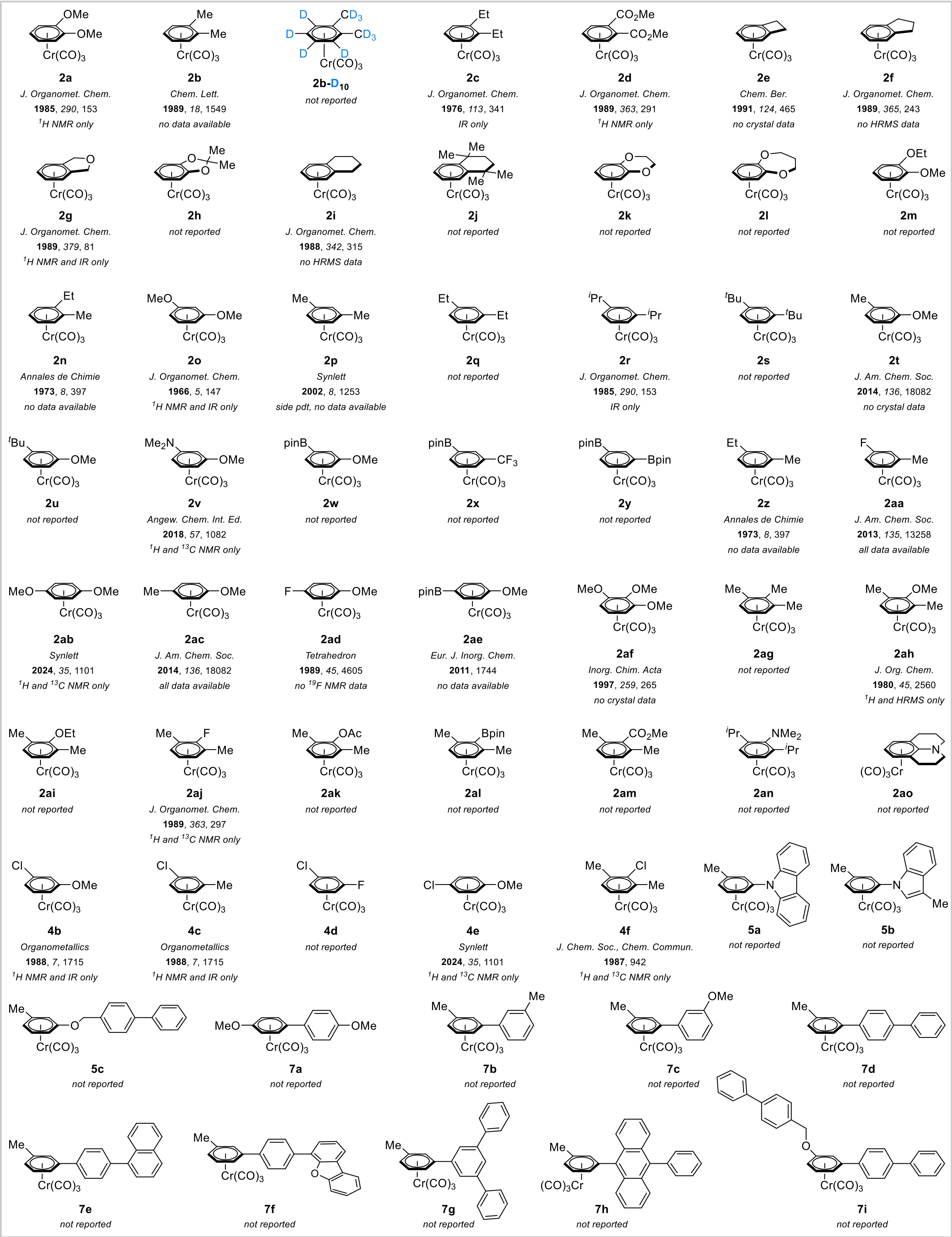

Figure S2. Available spectroscopic data in the literature (based on the search from SciFinder®) for (η^6-arene)$Cr(CO)_3$ complexes, till date.[3-24]

2.6. Characterization of (η^6-arene)$Cr(CO)_3$ complexes

Note S2 – ^{13}C NMR peak characterization of (η^6-arene)$Cr(CO)_3$ complexes: The carbon-center directly attached to the boron atom was not detected due to quadrupolar broadening.

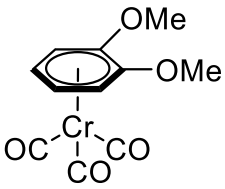

2a

1,2-Dimethoxybenzene chromium tricarbonyl (2a): Prepared according to **GP-A** using $Cr(CO)_6$ (2.2 g, 10.0 mmol, 1.0 equiv) and 1,2-dimethoxybenzene (1.28 mL, 10.0 mmol, 1.0 equiv) for 24 h. The crude reaction mixture was purified by flash column chromatography on silica gel (eluent: 1→20% ethyl acetate in cyclohexane) to afford the title compound **2a** as a yellow solid (1.92 g, 70%).

^{1}H NMR (500 MHz, C_6D_6) δ 4.36 (dd, *J* = 4.9, 2.9 Hz, 2H), 4.20 (dd, *J* = 4.9, 2.9 Hz, 2H), 3.05 (s, 6H).

^{13}C NMR (125 MHz, C_6D_6) δ 234.2, 133.7, 86.8, 78.4, 56.6.

IR (neat, cm^{-1}) 1947, 1828, 1528, 1483, 1439, 1416, 1268, 1014, 671, 633.

HRMS (APCI) exact mass calcd. for $C_{11}H_{11}CrO_5$ $[M + H]^+$: 275.0006, found 275.0009.

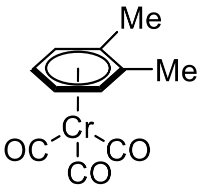

2b

1,2-Dimethylbenzene chromium tricarbonyl (2b): Prepared according to **GP-A** using $Cr(CO)_6$ (1.1 g, 5.0 mmol, 1.0 equiv) and *ortho*-xylene (0.60 mL, 5.0 mmol, 1.0 equiv) for 36 h. The crude reaction mixture was purified by flash column chromatography on silica gel (eluent: 1→10% ethyl acetate in cyclohexane) to afford the title compound **2b** as a yellow solid (1.02 g, 84%).

^{1}H NMR (400 MHz, C_6D_6) δ 4.50 – 4.41 (m, 2H), 4.40 – 4.29 (m, 2H), 1.51 (s, 6H).

^{13}C NMR (100 MHz, C_6D_6) δ 234.2, 107.4, 95.0, 91.5, 18.3.

IR (neat, cm^{-1}) 1941, 1836, 1448, 1386, 1027, 831, 667, 628, 537, 506.

HRMS (APCI) exact mass calcd. for $C_{11}H_{11}CrO_3$ $[M + H]^+$: 243.0108; found: 243.0111.

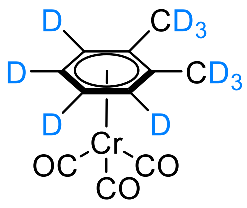

2b-D$_{10}$

***o*-Xylene-d$_{10}$ chromium tricarbonyl (2b-D$_{10}$):** Prepared according to **GP-B** using *o*-xylene-d$_{10}$ (0.62 mL, 4.0 mmol, 1.0 equiv) and $Cr(CO)_6$ (3.3 g, 6.0 mmol, 1.5 equiv) for 24 h. The crude reaction mixture was purified by flash column chromatography on silica gel (eluent: 1→10% ethyl acetate in cyclohexane) to afford the title compound **2b-D$_{10}$** as a yellow solid (2.22 g, 88%).

^{2}H NMR (77 MHz, C_6H_6) δ 4.64 – 4.16 (m, 4D), 1.44 (s, 6D).

^{13}C NMR (125 MHz, C_6H_6) δ 233.7, 106.7, 94.1 (t, J = 26.5 Hz), 90.7 (t, J = 24.3 Hz), 16.7 (t, J = 35.5 Hz).

IR (neat, cm^{-1}) 1940, 1832, 1034, 680, 630, 617, 534, 526, 483.

HRMS (APCI) exact mass calcd. for $C_{11}D_{10}HCrO_3$ $[M + H]^+$: 253.0736, found 253.0730.

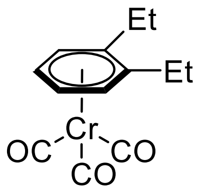

2c

1,2-Diethylbenzene chromium tricarbonyl (2c): Prepared according to **GP-A** using $Cr(CO)_6$ (440 mg, 2.0 mmol, 1.0 equiv) and 1,2-diethylbenzene (0.30 mL, 2.0 mmol, 1.0 equiv) for 24 h. The crude reaction mixture was purified by flash column chromatography on silica gel

(eluent: 1→10% ethyl acetate in cyclohexane) to afford the title compound **2c** as a yellow liquid (303 mg, 56%).

^{1}H NMR (400 MHz, C_6D_6) δ 4.52 – 4.42 (m, 4H), 2.02 (dq, *J* = 15.1, 7.6 Hz, 2H), 1.88 (dq, *J* = 15.1, 7.6 Hz, 2H), 0.75 (t, *J* = 7.6 Hz, 6H).

^{13}C NMR (125 MHz, C_6D_6) δ 234.2, 113.1, 93.0, 91.8, 24.8, 14.6.

IR (neat, cm^{-1}) 1948, 1844, 663, 626, 531, 475.

HRMS (EI) exact mass calcd. for $C_{13}H_{14}CrO_3$ $[M]^+$: 270.0348; found: 270.0334.

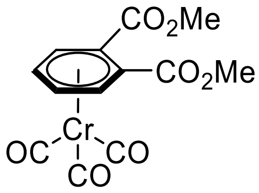

2d

Dimethyl phthalate chromium tricarbonyl (2d): Prepared according to **GP-A** using $Cr(CO)_6$ (1.1 g, 5.0 mmol, 1.0 equiv) and dimethyl phthalate (0.82 mL, 5.00 mmol, 1.00 equiv) for 24 h. The crude reaction mixture was purified by flash column chromatography on silica gel (eluent: 1→20% ethyl acetate in cyclohexane) to afford the title compound **2d** as a red liquid (767 mg, 46%).

^{1}H NMR (500 MHz, C_6D_6) δ 5.00 (dd, *J* = 4.8, 2.8 Hz, 2H), 4.21 (dd, *J* = 4.8, 2.8 Hz, 2H), 3.39 (s, 6H).

^{13}C NMR (125 MHz, C_6D_6) δ 230.6, 165.8, 97.4, 91.1, 90.1, 52.8.

IR (neat, cm^{-1}) 1974, 1872, 1725, 1446, 1273, 1111, 643, 611, 525.

HRMS (APCI) exact mass calcd. for $C_{13}H_{11}CrO_7$ $[M + H]^+$: 330.9905; found: 330.9904.

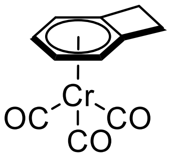

2e

Benzocyclobutene chromium tricarbonyl (2e): Prepared according to **GP-A** using $Cr(CO)_6$ (1.1 g, 5.0 mmol, 1.0 equiv) and benzocyclobutene (0.54 mL, 5.00 mmol, 1.00 equiv) for 24 h. The crude reaction mixture was purified by flash column chromatography on silica gel (eluent: 1→10% ethyl acetate in cyclohexane) to afford the title compound **2e** as a yellow solid (1.02 g, 85%).

^{1}H NMR (500 MHz, C_6D_6) δ 4.68 – 4.54 (m, 2H), 4.26 – 4.04 (m, 2H), 2.57 (d, *J* = 11.8 Hz, 2H), 2.27 (d, *J* = 11.5 Hz, 2H).

^{13}C NMR (125 MHz, C_6D_6) δ 234.0, 115.9, 90.5, 89.5, 29.7.

IR (neat, cm^{-1}) 1941, 1821, 1732, 1415, 1260, 1076, 1016, 796, 619, 528.

HRMS (APCI) exact mass calcd. for $C_{11}H_9CrO_3$ $[M + H]^+$: 240.9951; found: 240.9960.

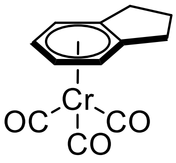

2f

Indane chromium tricarbonyl (2f): Prepared according to **GP-A** using $Cr(CO)_6$ (440 mg, 2.0 mmol, 1.0 equiv) and indane (0.24 mL, 2.0 mmol, 1.0 equiv) for 48 h. The crude reaction mixture was purified by flash column chromatography on silica gel (eluent: 1→10% ethyl acetate in cyclohexane) to afford the title compound **2f** as a yellow solid (459 mg, 90%).

^{1}H NMR (500 MHz, C_6D_6) δ 4.61 – 4.56 (m, 2H), 4.39 – 4.34 (m, 2H), 2.18 (dd, *J* = 15.5, 8.6 Hz, 2H), 2.02 (ddd, *J* = 15.4, 10.5, 7.9 Hz, 2H), 1.74 (dt, *J* = 20.0, 9.7 Hz, 1H), 1.39 (dt, *J* = 14.0, 7.9 Hz, 1H).

^{13}C NMR (125 MHz, C_6D_6) δ 234.0, 114.0, 91.4, 90.1, 31.6, 23.5.

IR (neat, cm^{-1}) 1938, 1862, 1842, 1449, 1436, 1039, 799, 664, 628, 545, 529, 408, 471.

HRMS (APCI) exact mass calcd. for $C_{12}H_{11}CrO_3$ $[M + H]^+$: 255.0108, found 255.0102.

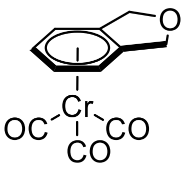

2g

Phthalane chromium tricarbonyl (2g): Prepared according to **GP-A** using $Cr(CO)_6$ (1.1 g, 5.0 mmol, 1.0 equiv) and phthalane (0.55 mL, 5.0 mmol, 1.0 equiv) for 48 h. The crude reaction mixture was purified by flash column chromatography on silica gel (eluent: 1→10% ethyl acetate in cyclohexane) to afford the title compound **2g** as a yellow solid (1.0 g, 78%).

^{1}H NMR (400 MHz, C_6D_6) δ 4.42 – 4.34 (m, 4H), 4.29 – 4.20 (m, 2H), 4.17 – 4.09 (m, 2H).

^{13}C NMR (100 MHz, C_6D_6) δ 232.9, 109.4, 90.5, 85.7, 71.6.

IR (neat, cm^{-1}) 1953, 1853, 1729, 1699, 1432, 1151, 1042, 901, 667, 628, 531, 482.

HRMS (APCI) exact mass calcd. for $C_{11}H_9CrO_4$ [M + H]$^+$: 256.9901; found: 256.9904.

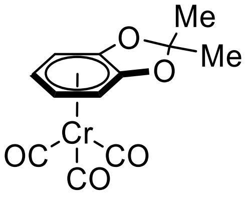

2h

2,2-Dimethylbenzo[*d*][1,3]dioxole chromium tricarbonyl (2h): Prepared according to **GP-A** using $Cr(CO)_6$ (1.1 g, 5.0 mmol, 1.0 equiv) and 2,2-dimethylbenzo[*d*][1,3]dioxole (0.70 mL, 5.0 mmol, 1.0 equiv) for 24 h. The crude reaction mixture was purified by flash column chromatography on silica gel (eluent: 1→20% ethyl acetate in cyclohexane) to afford the title compound **2h** as a yellow solid (272 mg, 19%).

^{1}H NMR (400 MHz, C_6D_6) δ 4.67 (dd, *J* = 4.8, 2.7 Hz, 2H), 3.93 (dd, *J* = 4.8, 2.7 Hz, 2H), 1.48 (s, 3H), 0.97 (s, 3H).

^{13}C NMR (100 MHz, C_6D_6) δ 234.3, 129.7, 121.9, 85.9, 76.2, 26.3, 23.8.

IR (neat, cm^{-1}) 1933, 1835, 1824, 1469, 1379, 1271, 1099, 971, 957, 806, 766, 628, 484.

HRMS (APCI) exact mass calcd. for $C_{12}H_{11}CrO_5$ [M + H]$^+$: 287.0006; found: 287.0011.

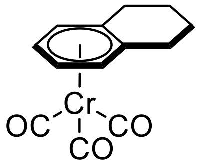

2i

1,2,3,4-Tetrahydronaphthalene chromium tricarbonyl (2i): Prepared according to **GP-A** using $Cr(CO)_6$ (1.10 g, 5.0 mmol, 1.0 equiv) and 1,2,3,4-tetrahydronaphthalene (0.68 mL, 5.0 mmol, 1.0 equiv) for 48 h. The crude reaction mixture was purified by flash column chromatography on silica gel (eluent: 1→10% ethyl acetate in cyclohexane) to afford the title compound **2i** as a yellow solid (0.99 g, 74%).

^{1}H NMR (500 MHz, C_6D_6) δ 4.72 – 4.30 (m, 4H), 2.22 (dt, *J* = 17.1, 6.2 Hz, 2H), 1.90 (dt, *J* = 16.5, 6.2 Hz, 2H), 1.44 – 1.33 (m, 2H), 1.17 – 1.06 (m, 2H).

^{13}C NMR (125 MHz, C_6D_6) δ 234.3, 109.3, 93.7, 91.6, 28.0, 22.0.

IR (neat, cm^{-1}) 1943, 1841, 1531, 1435, 1250, 825, 671, 630, 535, 504, 484.

HRMS (APCI) exact mass calcd. for $C_{13}H_{13}CrO_3$ $[M + H]^+$: 269.0264; found: 269.0260.

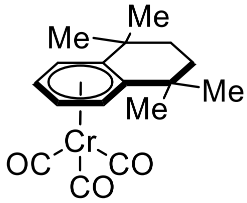

2j

1,1,4,4-Tetramethyl-1,2,3,4-tetrahydronaphthalene chromium tricarbonyl (2j): Prepared according to **GP-A** using $Cr(CO)_6$ (1.1 g, 5.0 mmol, 1.0 equiv) and 1,1,4,4-tetramethyl-1,2,3,4-tetrahydronaphthalene (1.02 mL, 5.0 mmol, 1.0 equiv) for 24 h. The crude reaction mixture was purified by flash column chromatography on silica gel (eluent: 1→10% ethyl acetate in cyclohexane) to afford the title compound **2j** as a yellow solid (454 mg, 28%).

^{1}H NMR (500 MHz, C_6D_6) δ 4.66 (dd, *J* = 6.2, 3.2 Hz, 2H), 4.48 (dd, *J* = 4.8, 2.9 Hz, 2H), 1.59 – 1.49 (m, 2H), 1.27 – 1.17 (m, 2H), 1.15 (s, 6H), 0.81 (s, 6H).

^{13}C NMR (125 MHz, C_6D_6) δ 234.5, 121.8, 91.9, 90.5, 34.0, 33.2, 33.0, 32.2.

IR (neat, cm^{-1}) 1937, 1872, 1835, 1461, 669, 630, 532, 491.

HRMS (APCI) exact mass calcd. for $C_{17}H_{21}CrO_3$ [M + H]$^+$: 325.0890; found: 325.0897.

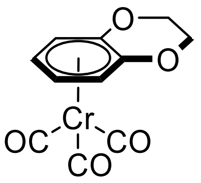

2k

2,3-Dihydrobenzo[*b*][1,4]dioxine chromium tricarbonyl (2k): Prepared according to **GP-A** using $Cr(CO)_6$ (440 mg, 5.0 mmol, 1.0 equiv) and 2,3-dihydrobenzo[*b*][1,4]dioxine (0.58 mL, 5.0 mmol, 1.0 equiv) for 48 h. The crude reaction mixture was purified by flash column chromatography on silica gel (eluent: 1→20% ethyl acetate in cyclohexane) to afford the title compound **2k** as a yellow solid (1.26 g, 93%).

^{1}H NMR (500 MHz, C_6D_6) δ 4.79 (dd, *J* = 5.0, 2.9 Hz, 2H), 4.07 (dd, *J* = 5.0, 2.9 Hz, 2H), 3.36 – 3.27 (m, 2H), 3.12 – 3.03 (m, 2H).

^{13}C NMR (125 MHz, C_6D_6) δ 234.3, 126.2, 87.6, 83.4, 64.4.

IR (neat, cm^{-1}) 1941, 1836, 1529, 1471, 1456, 1274, 1253, 1049, 825, 629.

HRMS (APCI) exact mass calcd. for $C_{11}H_9CrO_5$ [M + H]$^+$: 272.9850; found: 272.9846.

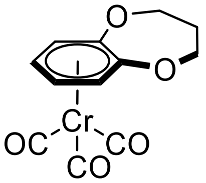

2l

3,4-Dihydro-2*H*-benzo[*b*][1,4]dioxepine chromium tricarbonyl (2l): Prepared according to **GP-A** using $Cr(CO)_6$ (1.1 g, 5.0 mmol, 1.0 equiv) and 3,4-Dihydro-2*H*-benzo[*b*][1,4]dioxepine (0.68 mL, 5.0 mmol, 1.0 equiv) for 48 h. The crude reaction mixture was purified by flash column chromatography on silica gel (eluent: 1→20% ethyl acetate in cyclohexane) to afford the title compound **2l** as a yellow solid (1.32 g, 92%).

^{1}H NMR (400 MHz, C_6D_6) δ 4.91 – 4.84 (m, 2H), 4.15 – 4.07 (m, 2H), 3.85 – 3.74 (m, 2H), 3.50 – 3.37 (m, 2H), 1.46 – 1.27 (m, 1H), 1.02 – 0.88 (m, 1H).

^{13}C NMR (100 MHz, C_6D_6) δ 234.2, 132.1, 89.6, 88.5, 74.1, 31.9.

IR (neat, cm^{-1}) 1941, 1829, 1511, 1451, 1232, 1039, 1014, 824, 667, 630, 528, 481.

HRMS (APCI) exact mass calcd. for $C_{12}H_{11}CrO_5$ $[M + H]^+$: 287.0006; found: 287.0000.

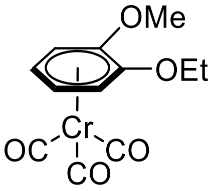

2m

1-Ethoxy-2-methoxybenzene chromium tricarbonyl (2m): Prepared according to **GP-B** using 1-ethoxy-2-methoxybenzene (0.61 mL, 4.0 mmol, 1.0 equiv) and $Cr(CO)_6$ (1.32 g, 6.0 mmol, 1.5 equiv) for 48 h. The crude reaction mixture was purified by flash column chromatography on silica gel (eluent: 1→20% ethyl acetate in cyclohexane) to afford the title compound **2m** as a yellow solid (1.04 g, 90%).

^{1}H NMR (500 MHz, C_6D_6) δ 4.40 (dd, J = 18.2, 6.5 Hz, 2H), 4.23 (dt, J = 23.0, 6.3 Hz, 2H), 3.40 (p, J = 6.9 Hz, 1H), 3.26 (p, J = 6.8 Hz, 1H), 3.06 (s, 3H), 1.03 (t, J = 7.0 Hz, 3H).

^{13}C NMR (125 MHz, C_6D_6) δ 234.3, 133.5, 133.1, 87.1, 86.5, 79.1, 78.7, 66.2, 56.6, 14.6.

IR (cm^{-1}) 2981, 1974, 1898, 1862, 1383, 1360, 1298, 1267, 1125, 1060, 861, 636, 613, 528.

HRMS (APCI) exact mass calcd. for $C_{12}H_{13}CrO_5$ $[M + H]^+$: 289.0163; found: 289.0159.

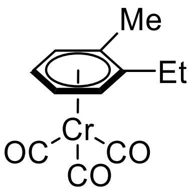

2n

1-Ethyl-2-methylbenzene chromium tricarbonyl (2n): Prepared according to **GP-A** using $Cr(CO)_6$ (1.1 g, 5.0 mmol, 1.0 equiv) and 1-ethyl-2-methylbenzene (1.40 mL, 10.0 mmol, 2.0 equiv) for 30 h. The crude reaction mixture was purified by flash column chromatography on silica gel (eluent: 1→10% ethyl acetate in cyclohexane) to afford the title compound **2n** as a yellow liquid (1.01 g, 79%).

^{1}H NMR (500 MHz, C_6D_6) δ 4.54 – 4.49 (m, 1H), 4.47 – 4.42 (m, 1H), 4.42 – 4.36 (m, 2H), 1.97 (dq, *J* = 15.0, 7.5 Hz, 1H), 1.81 (dq, *J* = 15.0, 7.6 Hz, 1H), 1.57 (s, 3H), 0.70 (t, *J* = 7.5 Hz, 3H).

^{13}C NMR (125 MHz, C_6D_6) δ 234.2, 112.9, 107.4, 94.6, 93.6, 92.1, 91.2, 25.5, 17.9, 14.2.

IR (neat, cm^{-1}) 2973, 1947, 1841, 1461, 1383, 825, 663, 624.

HRMS (EI) exact mass calcd. for $C_{12}H_{12}CrO_3$ $[M]^+$: 256.0191; found: 256.0186.

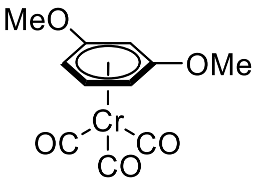

2o

1,3-Dimethoxybenzene chromium tricarbonyl (2o): Prepared according to **GP-A** using $Cr(CO)_6$ (1.54 g, 7.0 mmol, 1.0 equiv) and 1,3-dimethoxybenzene (1.82 mL, 14.0 mmol, 2.0 equiv) for 24 h. The crude reaction mixture was purified by flash column chromatography on silica gel (eluent: 1→20% ethyl acetate in cyclohexane) to afford the title compound **2o** as a yellow solid (1.24 g, 65%).

^{1}H NMR (500 MHz, C_6D_6) δ 4.78 – 4.70 (m, 2H), 4.06 (dd, *J* = 6.7, 2.0 Hz, 2H), 2.95 (s, 6H).

^{13}C NMR (125 MHz, C_6D_6) δ 234.1, 143.9, 93.0, 72.8, 69.3, 55.2.

IR (neat, cm^{-1}) 1940, 1881, 1824, 1750, 1545, 1415, 1274, 1166, 857, 626.

HRMS (EI) exact mass calcd. for $C_{11}H_{10}CrO_5$ $[M]^+$: 273.9933, found 273.9924.

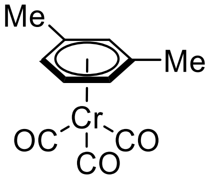

2p

***m*-Xylene chromium tricarbonyl (2p):** Prepared according to **GP-A** using $Cr(CO)_6$ (1.1 g, 5.0 mmol, 1.0 equiv) and *meta*-xylene (0.61 mL, 5.0 mmol, 1.0 equiv) for 48 h. The crude

reaction mixture was purified by flash column chromatography on silica gel (eluent: 1→10% ethyl acetate in cyclohexane) to afford the title compound **2p** as a yellow solid (1.00 g, 83%).

^{1}H NMR (500 MHz, C_6D_6) δ 4.63 (t, *J* = 6.3 Hz, 1H), 4.16 – 4.10 (m, 3H), 1.59 (s, 6H).

^{13}C NMR (125 MHz, C_6D_6) δ 234.1, 110.2, 95.1, 93.5, 89.8, 20.3.

IR (neat, cm^{-1}) 1944, 1836, 1824, 1539, 1452, 1379, 1037, 670, 630.

HRMS (APCI) exact mass calcd. for $C_{11}H_{11}CrO_3$ [M + H]$^+$: 243.0108, found 243.0109.

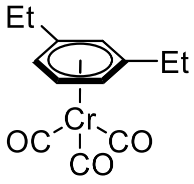

2q

1,3-Diethylbenzene chromium tricarbonyl (2q): Prepared according to **GP-A** using $Cr(CO)_6$ (1.97 g, 8.95 mmol, 1.0 equiv) and 1,3-diethylbenzene (1.40 mL, 8.95 mmol, 1.0 equiv) for 36 h. The crude reaction mixture was purified by flash column chromatography on silica gel (eluent: 1→10% ethyl acetate in cyclohexane) to afford the title compound **2q** as a yellow solid (2.13 mg, 88%).

^{1}H NMR (500 MHz, C_6D_6) δ 4.64 (t, *J* = 6.5 Hz, 1H), 4.41 (s, 1H), 4.28 (d, *J* = 6.4 Hz, 2H), 1.95 (q, *J* = 7.7 Hz, 4H), 0.79 (t, *J* = 7.6 Hz, 6H).

^{13}C NMR (125 MHz, C_6D_6) δ 234.1, 115.8, 94.6, 92.0, 89.7, 28.0, 14.7.

IR (neat, cm^{-1}) 2974, 1951, 1862, 1839, 1453, 666, 620.

HRMS (APCI) exact mass calcd. for $C_{13}H_{15}CrO_3$ [M + H]$^+$: 271.0421; found: 271.0416.

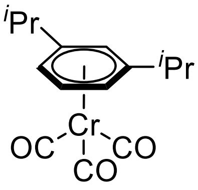

2r

1,3-Diisopropylbenzene chromium tricarbonyl (2r): Prepared according to **GP-A** using $Cr(CO)_6$ (1.1 g, 5.0 mmol, 1.0 equiv) and 1,3-diisopropylbenzene (0.94 mL, 5.0 mmol, 1.0 equiv) for 48 h. The crude reaction mixture was purified by flash column chromatography on silica gel (eluent: 1→10% ethyl acetate in cyclohexane) to afford the title compound **2r** as a yellow solid (1.01 g, 68%).

^{1}H NMR (500 MHz, C_6D_6) δ 4.79 (s, 1H), 4.60 – 4.55 (m, 1H), 4.54 – 4.48 (m, 2H), 2.22 (hept, *J* = 6.9 Hz, 2H), 0.90 (t, *J* = 6.7 Hz, 12H).

^{13}C NMR (125 MHz, C_6D_6) δ 234.1, 119.6, 92.6, 91.2, 90.4, 32.8, 23.5, 22.9.

IR (neat, cm^{-1}) 2974, 1944, 1868, 1851, 1463, 667, 624, 535, 486, 479.

HRMS (APCI) exact mass calcd. for $C_{15}H_{19}CrO_3$ [M + H]$^+$: 299.0739; found: 299.0734.

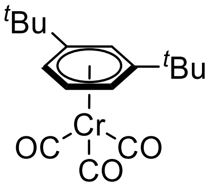

2s

1,3-Di-*tert*-butylbenzene chromium tricarbonyl (2s): Prepared according to **GP-A** using $Cr(CO)_6$ (1.1 g, 5.0 mmol, 1.0 equiv) and 1,3-di-*tert*-butylbenzene (1.11 mL, 5.0 mmol, 1.0 equiv) for 24 h. The crude reaction mixture was purified by flash column chromatography on silica gel (eluent: 1→10% ethyl acetate in cyclohexane) to afford the title compound **2s** as a yellow solid (1.42 g, 87%).

^{1}H NMR (500 MHz, C_6D_6) δ 5.53 (s, 1H), 4.97 (d, *J* = 6.6 Hz, 2H), 4.35 (t, *J* = 6.6 Hz, 1H), 1.02 (s, 18H).

^{13}C NMR (125 MHz, C_6D_6) δ 234.6, 120.4, 93.2, 92.3, 88.3, 33.9, 31.2.

IR (neat, cm^{-1}) 1946, 1850, 1515, 1433, 1372, 1029, 841, 663, 623.

HRMS (APCI) exact mass calcd. for $C_{17}H_{23}CrO_3$ [M + H]$^+$: 327.1047; found: 327.1057.

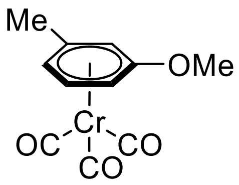

2t

3-Methyl anisole chromium tricarbonyl (2t): Prepared according to **GP-A** using $Cr(CO)_6$ (2.24 g, 10.2 mmol, 1.0 equiv) and 3-methyl anisole (2.58 mL, 20.4 mmol, 2.0 equiv) for 48 h. The crude reaction mixture was purified by flash column chromatography on silica gel (eluent: 1→20% ethyl acetate in cyclohexane) to afford the title compound **2t** as a yellow solid (2.41 g, 92%).

^{1}H NMR (500 MHz, C_6D_6) δ 4.72 (dd, *J* = 6.7, 6.5 Hz, 1H), 4.43 (s, 1H), 4.23 (d, *J* = 5.9 Hz, 1H), 3.90 (d, *J* = 6.3 Hz, 1H), 2.96 (s, 3H), 1.64 (s, 3H).

^{13}C NMR (125 MHz, C_6D_6) δ 234.1, 143.7, 110.4, 94.8, 86.5, 80.3, 75.6, 55.0, 20.4.

IR (neat, cm^{-1}) 1948, 1881, 1839, 1537, 1436, 1271, 1037, 826, 677, 664.

HRMS (EI) exact mass calcd. for $C_{11}H_{10}CrO_4$ [M]$^+$: 257.9984; found: 257.9980.

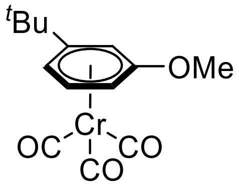

2u

1-(*Tert*-butyl)-3-methoxybenzene chromium tricarbonyl (2u): Prepared according to **GP-A** using $Cr(CO)_6$ (1.1 g, 5.0 mmol, 1.0 equiv) and 1-(*tert*-butyl)-3-methoxybenzene (0.90 mL, 5.0 mmol, 1.0 equiv) for 24 h. The crude reaction mixture was purified by flash column chromatography on silica gel (eluent: 1→20% ethyl acetate in cyclohexane) to afford the title compound **2u** as a yellow solid (1.05 g, 70%).

^{1}H NMR (500 MHz, C_6D_6) δ 5.02 – 4.98 (m, 1H), 4.72 (t, *J* = 6.6 Hz, 1H), 4.35 – 4.28 (m, 2H), 2.99 (s, 3H), 1.00 (s, 9H).

^{13}C NMR (125 MHz, C_6D_6) δ 234.2, 142.8, 126.1, 93.7, 84.1, 78.8, 76.0, 55.1, 34.0, 30.5.

IR (neat, cm^{-1}) 2981, 1943, 1845, 1280, 1232, 1027, 666, 634, 611, 541, 529.

HRMS (EI) exact mass calcd. for $C_{14}H_{16}CrO_4$ $[M]^+$: 300.0454; found: 300.0451.

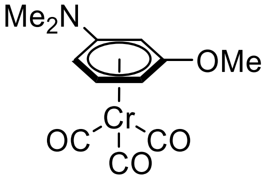

2v

3-Methoxy-*N*,*N*-dimethylaniline chromium tricarbonyl (2v): Prepared according to **GP-A** using $Cr(CO)_6$ (1.1 g, 5.0 mmol, 1.0 equiv) and 3-methoxy-*N*,*N*-dimethylaniline (0.76 mL, 5.0 mmol, 1.0 equiv) for 48 h. The crude reaction mixture was purified by flash column chromatography on silica gel (eluent: 1→30% ethyl acetate in cyclohexane) to afford the title compound **2v** as a yellow solid (1.28 g, 89%).

^{1}H NMR (500 MHz, C_6D_6) δ 4.95 (t, *J* = 6.8 Hz, 1H), 4.54 (dd, *J* = 2.1, 2.1 Hz, 1H), 4.12 (dd, *J* = 6.6, 2.0 Hz, 1H), 3.79 (dd, *J* = 7.0, 2.3 Hz, 1H), 3.12 (s, 3H), 2.10 (s, 6H).

^{13}C NMR (125 MHz, C_6D_6) δ 235.4, 145.1, 135.0, 94.5, 70.4, 69.3, 66.9, 55.0, 39.1.

IR (cm^{-1}) 2981, 1931, 1822, 1245, 1156, 673, 626, 528, 478.

HRMS (APCI) exact mass calcd. for $C_{12}H_{14}CrNO_4$ $[M + H]^+$: 288.0322; found: 288.0323.

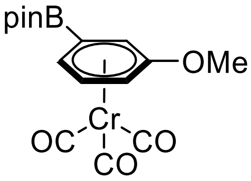

2w

2-(3-Methoxyphenyl)-4,4,5,5-tetramethyl-1,3,2-dioxaborolanechromium tricarbonyl (2w): Prepared according to **GP-B** using 4-(3-methoxyphenyl)-4,4,5,5-tetramethyl-1,3,2-dioxaborolane (468 mg, 2.0 mmol, 1.0 equiv) and $Cr(CO)_6$ (0.66 g, 3 mmol, 1.5 equiv) for 24 h. The crude reaction mixture was purified by flash column chromatography on silica gel

(eluent: 1→20% ethyl acetate in cyclohexane) to afford the title compound **2w** as a yellow solid (710 mg, 96%).

^{1}H NMR (500 MHz, C_6D_6) δ 5.35 (s, 1H), 5.06 (d, *J* = 5.9 Hz, 1H), 4.65 – 4.58 (m, 2H), 2.84 (s, 3H), 1.14 (s, 12H).

^{13}C NMR (125 MHz, C_6D_6) δ 233.7, 142.5, 94.7, 92.1, 85.0, 82.3, 81.5, 55.0, 25.0, 24.9.

IR (neat, cm^{-1}) 2980, 1956, 1859, 1375, 1141, 1080, 1030, 664, 626.

HRMS (EI) exact mass calcd. for $C_{16}H_{19}BCrO_6$ [M]$^+$: 370.0680; found: 370.0679.

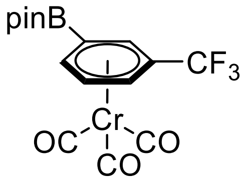

2x

4,4,5,5-Tetramethyl-2-(3-(trifluoromethyl)phenyl-1,3,2-dioxaborolane chromium tricarbonyl (2x): Prepared according to **GP-B** using 4,4,5,5-tetramethyl-2-(3-(trifluoromethyl)phenyl-1,3,2-dioxaborolane (0.95 mL, 4.0 mmol, 1.0 equiv) and $Cr(CO)_6$ (1.32 g, 6.0 mmol, 1.5 equiv) for 24 h. The crude reaction mixture was purified by flash column chromatography on silica gel (eluent: 1→20% ethyl acetate in cyclohexane) to afford the title compound **2x** as a yellow solid (441 mg, 27%).

^{1}H NMR (500 MHz, C_6D_6) δ 6.08 (s, 1H), 5.45 (d, *J* = 6.3 Hz, 1H), 5.02 (d, *J* = 6.6 Hz, 1H), 4.02 – 3.98 (m, 1H), 1.05 (s, 12H).

^{13}C NMR (125 MHz, C_6D_6) δ 230.1, 124.7 (q, *J* = 272.8 Hz), 100.1, 96.5 (q, *J* = 2.7 Hz), 94.9 (q, *J* = 36.4 Hz), 91.9 (q, *J* = 2.5 Hz), 87.3, 85.0, 24.6 (2C).

^{19}F NMR (471 MHz, C_6D_6) δ -60.9.

IR (neat, cm^{-1}) 2981, 1976, 1905, 1361, 1191, 1126, 1062, 643, 616.

HRMS (APCI) exact mass calcd. for $C_{16}H_{17}BCrF_3O_5$ [M + H]$^+$: 409.0522; found: 409.0533.

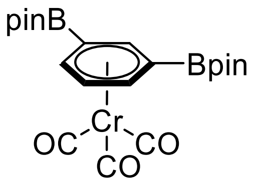

2y

1,3-Bis(4,4,5,5-tetramethyl-1,3,2-dioxaborolan-2-yl)benzene chromium tricarbonyl (2y): Prepared according to **GP-B** using 1,3-bis(4,4,5,5-tetramethyl-1,3,2-dioxaborolan-2-yl)benzene (1.32 g, 4.0 mmol, 1.0 equiv) and $Cr(CO)_6$ (1.32 g, 6.0 mmol, 1.5 equiv) for 20 h. The crude reaction mixture was purified by flash column chromatography on silica gel (eluent: 1→20% ethyl acetate in cyclohexane) to afford the title compound **2y** as a yellow solid (1.49 g, 80%).

^{1}H NMR (500 MHz, C_6D_6) δ 6.49 (s, 1H), 5.74 (d, J = 6.2 Hz, 2H), 4.29 (t, J = 6.8 Hz, 1H), 1.07 (s, 24H).

^{13}C NMR (125 MHz, C_6D_6) δ 232.5, 106.6, 101.6, 90.7, 84.5, 24.6 (2C).

IR (cm^{-1}) 1963, 1914, 1887, 1351, 1334, 1138, 654, 613, 531, 478.

HRMS (APCI) exact mass calcd. for $C_{21}H_{29}B_2CrO_7$ $[M + H]^+$: 467.1508; found: 467.1505.

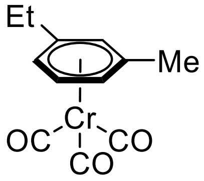

2z

1-Ethyl-3-methylbenzene chromium tricarbonyl (2z): Prepared according to **GP-A** using $Cr(CO)_6$ (440 mg, 2.0 mmol, 1.0 equiv) and 1-ethyl-3-methylbenzene (0.28 mL, 2.0 mmol, 1.0 equiv) for 48 h. The crude reaction mixture was purified by flash column chromatography on silica gel (eluent: 1→10% ethyl acetate in cyclohexane) to afford the title compound **2z** as a yellow solid (168 mg, 33%).

^{1}H NMR (500 MHz, C_6D_6) δ 4.68 – 4.61 (m, 1H), 4.28 (s, 1H), 4.23 – 4.16 (m, 2H), 1.94 (q, J = 7.7 Hz, 2H), 1.61 (s, 3H), 0.78 (t, J = 7.6 Hz, 3H).

^{13}C NMR (125 MHz, C_6D_6) δ 234.1, 116.1, 110.0, 94.9, 92.7, 90.4, 89.0, 28.0, 20.3, 14.7.

IR (cm^{-1}) 2967, 1947, 1841, 1535, 1436, 1382, 1152, 835, 792, 663, 624.

HRMS (APCI) exact mass calcd. for $C_{12}H_{13}CrO_3$ $[M + H]^+$: 257.0264; found: 257.0262.

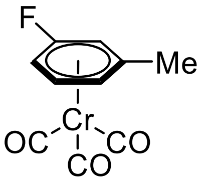

2aa

1-Fluoro-3-methylbenzene chromium tricarbonyl (2aa): Prepared according to **GP-A** using $Cr(CO)_6$ (660 mg, 3.0 mmol, 1.0 equiv) and 3-fluorotoluene (0.33 mL, 3.00 mmol, 1.00 equiv) for 24 h. The crude reaction mixture was purified by flash column chromatography on silica gel (eluent: 1→10% ethyl acetate in cyclohexane) to afford the title compound **2aa** as a yellow solid (577 mg, 78%).

^{1}H NMR (400 MHz, C_6D_6) δ 4.45 – 4.37 (m, 1H), 4.34 – 4.23 (m, 2H), 3.65 – 3.57 (m, 1H), 1.46 (s, 3H).

^{13}C NMR (100 MHz, C_6D_6) δ 232.6 (d, J = 2.5 Hz), 147.3 (d, J = 265.1 Hz), 109.1 (d, J = 7.3 Hz), 93.1 (d, J = 7.4 Hz), 87.1, 80.6 (d, J = 19.4 Hz), 76.8 (d, J = 19.9 Hz), 19.8.

^{19}F NMR (471 MHz, C_6D_6) δ -134.6

IR (cm^{-1}) 1945, 1874, 1263, 1136, 826, 670, 645, 623.

HRMS (EI) exact mass calcd. for $C_{10}H_7FCrO_3$ $[M]^+$: 245.9784; found: 245.9779.

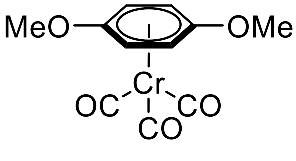

2ab

1,4-Dimethoxybenzenechromium tricarbonyl (2ab): Prepared according to **GP-A** using $Cr(CO)_6$ (2.2 g, 10.0 mmol, 1.0 equiv) and 1,4-dimethoxybenzene (2.76 g, 20.0 mmol, 2.0 equiv) for 48 h. The crude reaction mixture was purified by flash column chromatography on silica gel (eluent: 1→20% ethyl acetate in cyclohexane) to afford the title compound **2ab** as a yellow solid (1.37 g, 50%).

^{1}H NMR (500 MHz, C_6D_6) δ 4.50 (s, 4H), 2.90 (s, 6H).

^{13}C NMR (125 MHz, C_6D_6) δ 234.3, 136.8, 79.5, 55.8.

IR (neat, cm^{-1}) 1937, 1844, 1555, 1524, 1486, 1156, 1003, 673, 630.

HRMS (EI) exact mass calcd. for $C_{11}H_{10}CrO_5$ $[M]^+$: 273.9933, found 273.9924.

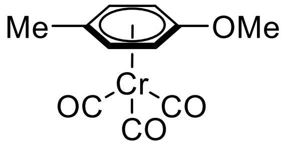

2ac

4-Methyl anisole chromium tricarbonyl (2ac): Prepared according to **GP-A** using $Cr(CO)_6$ (2.2 g, 10.0 mmol, 1.0 equiv) and 4-methyl anisole (2.52 mL, 20.0 mmol, 2.0 equiv) for 48 h. The crude reaction mixture was purified by flash column chromatography on silica gel (eluent: 1→20% ethyl acetate in cyclohexane) to afford the title compound **2ac** as a yellow solid (1.55 g, 60%).

^{1}H NMR (500 MHz, C_6D_6) δ 4.56 (d, J = 6.6 Hz, 2H), 4.40 (d, J = 6.6 Hz, 2H), 2.90 (s, 3H), 1.45 (s, 3H).

^{13}C NMR (125 MHz, C_6D_6) δ 234.1, 141.3, 101.3, 95.1, 78.7, 55.2, 19.3.

IR (neat, cm^{-1}) 2923, 1940, 1831, 1248, 1017, 674, 666, 621.

HRMS (APCI) exact mass calcd. for $C_{11}H_{11}CrO_4$ $[M + H]^+$: 259.0057; found: 259.0052.

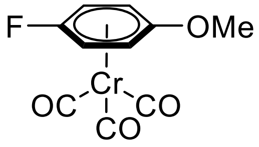

2ad

1-Fluoro-4-methoxybenzene chromium tricarbonyl (2ad): Prepared according to **GP-A** using $Cr(CO)_6$ (2.2 g, 10.0 mmol, 1.0 equiv) and 1-fluoro-4-methoxybenzene (2.27 mL, 20.0 mmol, 2.0 equiv) for 36 h. The crude reaction mixture was purified by flash column chromatography on silica gel (eluent: 1→10% ethyl acetate in cyclohexane) to afford the title compound **2ad** as a yellow solid (1.86 g, 71%).

^{1}H NMR (400 MHz, C_6D_6) δ 4.61 (dd, *J* = 7.0, 4.1 Hz, 2H), 4.20 (d, *J* = 5.7 Hz, 2H), 2.74 (s, 3H).

^{13}C NMR (100 MHz, C_6D_6) δ 232.7, 139.9 (d, *J* = 260.8 Hz), 137.6, 80.8 (d, *J* = 22.2 Hz), 77.5 (d, *J* = 7.4 Hz), 55.8.

^{19}F NMR (471 MHz, C_6D_6) δ -142.4.

IR (neat, cm^{-1}) 1960, 1836, 1478, 1435, 1248, 1220, 1136, 1013, 617.

HRMS (EI) exact mass calcd. for $C_{10}H_7FCrO_4$ $[M]^+$: 261.9733, found 261.9727.

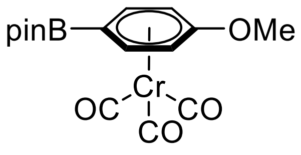

2ae

2-(4-Methoxyphenyl)-4,4,5,5-tetramethyl-1,3,2-dioxaborolane chromium tricarbonyl (2ae): Prepared according to **GP-B** using 2-(4-methoxyphenyl)-4,4,5,5-tetramethyl-1,3,2-dioxaborolane (936 mg, 4.0 mmol, 1.0 equiv) and $Cr(CO)_6$ (1.32 g, 6.0 mmol, 1.5 equiv) for 20 h. The crude reaction mixture was purified by flash column chromatography on silica gel (eluent: 1→20% ethyl acetate in cyclohexane) to afford the title compound **2ae** as a yellow solid (1.33 g, 90%).

^{1}H NMR (500 MHz, C_6D_6) δ 5.70 (d, *J* = 6.7 Hz, 2H), 4.34 (d, *J* = 6.7 Hz, 2H), 2.88 (s, 3H), 1.11 (s, 12H).

^{13}C NMR (125 MHz, C_6D_6) δ 233.1, 144.8, 100.6, 84.6, 78.5, 55.0, 24.7.

IR (neat, cm^{-1}) 2964, 1950, 1884, 1864, 1537, 1353, 1253, 1083, 1014, 798, 623.

HRMS (EI) exact mass calcd. for $C_{16}H_{19}BCrO_6$ $[M]^+$: 370.0680; found: 370.0679.

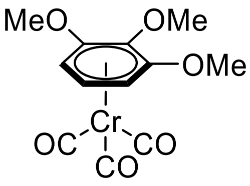

2af

1,2,3-Trimethoxylbenzene chromium tricarbonyl (2af): Prepared according to **GP-B** using 1,2,3-trimethoxybenzene (1.68 g, 10.0 mmol, 1.0 equiv) and $Cr(CO)_6$ (3.30 g, 15.0 mmol, 1.5 equiv) for 24 h. The crude reaction mixture was purified by flash column chromatography on silica gel (eluent: 1→30% ethyl acetate in cyclohexane) to afford the title compound **2af** as a yellow solid (2.22 g, 73%).

^{1}H NMR (500 MHz, C_6D_6) δ 4.59 (t, J = 6.7 Hz, 1H), 3.83 (d, J = 6.7 Hz, 2H), 3.80 (s, 3H), 3.05 (s, 6H).

^{13}C NMR (125 MHz, C_6D_6) δ 234.6, 140.3, 122.5, 89.2, 69.1, 66.3, 55.7.

IR (neat, cm^{-1}) 1940, 1849, 1828, 1522, 1459, 1418, 1294, 1247, 1098, 666.

HRMS (APCI) exact mass calcd. for $C_{12}H_{13}CrO_6$ [M + H]$^+$: 305.0112; found: 305.0115.

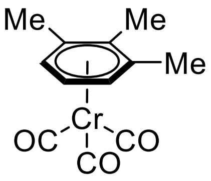

2ag

1,2,3-Trimethylbenzene chromium tricarbonyl (2ag): Prepared according to **GP-A** using $Cr(CO)_6$ (1.10 g, 5.0 mmol, 1.0 equiv) and 1,2,3-trimethylbenzene (0.68 mL, 5.0 mmol, 1.0 equiv) for 24 h. The crude reaction mixture was purified by flash column chromatography on silica gel (eluent: 1→10% ethyl acetate in cyclohexane) to afford the title compound **2ag** as a yellow solid (1.10 g, 86%).

^{1}H NMR (500 MHz, C_6D_6) δ 4.52 (t, J = 6.4 Hz, 1H), 4.34 (d, J = 6.3 Hz, 2H), 1.63 (s, 6H), 1.49 (s, 3H).

^{13}C NMR (125 MHz, C_6D_6) δ 234.6, 108.7, 106.2, 92.9, 92.6, 19.5, 14.5.

IR (neat, cm^{-1}) 2981, 1836, 1734, 1380, 672, 631, 526, 483.

HRMS (EI) exact mass calcd. for $C_{12}H_{12}CrO_3$ $[M]^+$: 256.0191; found: 256.0180.

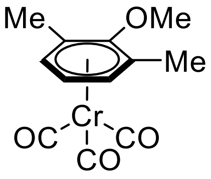

2ah

2,6-Dimethylanisole chromium tricarbonyl (2ah): Prepared according to **GP-A** using $Cr(CO)_6$ (1.1 g, 5.0 mmol, 1.0 equiv) and 2,6-dimethylanisole (0.71 mL, 5.0 mmol, 1.0 equiv) for 20 h. The crude reaction mixture was purified by flash column chromatography on silica gel (eluent: 1→20% ethyl acetate in cyclohexane) to afford the title compound **2ah** as a yellow solid (1.16 g, 85%).

^{1}H NMR (400 MHz, C_6D_6) δ 4.42 (t, J = 6.2 Hz, 1H), 4.23 (d, J = 6.2 Hz, 2H), 3.15 (s, 3H), 1.78 (s, 6H).

^{13}C NMR (100 MHz, C_6D_6) δ 234.2, 137.6, 105.5, 92.2, 90.9, 63.4, 15.8.

IR (neat, cm^{-1}) 1938, 1844, 1733, 1459, 1377, 1224, 996, 669, 628.

HRMS (APCI) exact mass calcd. for $C_{12}H_{13}CrO_4$ $[M + H]^+$: 273.0215; found: 273.0222.

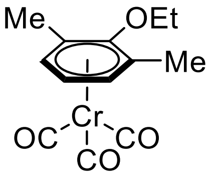

2ai

2-Ethoxy-1,3-dimethylbenzene chromium tricarbonyl (2ai): Prepared according to **GP-A** using $Cr(CO)_6$ (1.10 g, 5.0 mmol, 1.0 equiv) and 2-ethoxy-1,3-dimethylbenzene (0.75 g, 5.0 mmol, 1.0 equiv) for 24 h. The crude reaction mixture was purified by flash column chromatography on silica gel (eluent: 1→10% ethyl acetate in cyclohexane) to afford the title compound **2ai** as a yellow solid (1.14 g, 80%).

^{1}H NMR (500 MHz, C_6D_6) δ 4.43 (t, J = 6.2 Hz, 1H), 4.24 (d, J = 6.3 Hz, 2H), 3.41 (q, J = 7.0 Hz, 2H), 1.80 (s, 6H), 0.97 (t, J = 7.1 Hz, 3H).

^{13}C NMR (125 MHz, C_6D_6) δ 234.3, 136.6, 105.6, 92.3, 91.0, 73.0, 16.1, 15.4.

IR (neat, cm^{-1}) 2984, 1944, 1872, 1842, 1456, 1379, 1212, 1021, 630.

HRMS (APCI) exact mass calcd. for $C_{13}H_{15}CrO_4$ [M + H]$^+$: 287.0370; found: 287.0364.

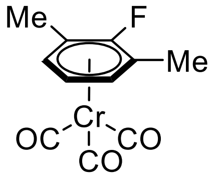

2aj

2-Fluoro-1,3-dimethylbenzene chromium tricarbonyl (2aj): Prepared according to **GP-A** using $Cr(CO)_6$ (2.20 g, 10.0 mmol, 1.0 equiv) and 2-fluoro-1,3-dimethylbenzene (1.24 g, 10.0 mmol, 1.0 equiv) for 48 h. The crude reaction mixture was purified by flash column chromatography on silica gel (eluent: 1→10% ethyl acetate in cyclohexane) to afford the title compound **2aj** as a yellow solid (2.44 g, 94%).

^{1}H NMR (500 MHz, C_6D_6) δ 4.27 – 4.19 (m, 2H), 4.12 – 4.02 (m, 1H), 1.70 (s, 6H).

^{13}C NMR (125 MHz, C_6D_6) δ 233.1, 142.6 (d, J = 260.6 Hz), 97.1 (d, J = 17.3 Hz), 92.1 (d, J = 4.2 Hz), 89.3, 14.3.

^{19}F NMR (471 MHz, C_6D_6) δ -144.3.

IR (neat, cm^{-1}) 1944, 1848, 1607, 1578, 1461, 1375, 1093, 822, 627.

HRMS (APCI) exact mass calcd. for $C_{11}H_{10}FCrO_3$ [M + H]$^+$: 261.0014, found 261.0020.

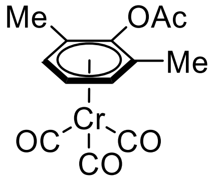

2ak

2,6-Dimethylphenyl acetate chromium tricarbonyl (2ak): Prepared according to **GP-A** using $Cr(CO)_6$ (1.10 g, 5.0 mmol, 1.0 equiv) and 2,6-dimethylphenyl acetate (0.82 g, 5.0 mmol, 1.0 equiv) for 20 h. The crude reaction mixture was purified by flash column chromatography on silica gel (eluent: 1→10% ethyl acetate in cyclohexane) to afford the title compound **1ak** as a yellow solid (1.03 g, 69%).

^{1}H NMR (500 MHz, C_6D_6) δ 4.42 – 4.18 (m, 3H), 1.70 (s, 6H), 1.55 (s, 3H).

^{13}C NMR (125 MHz, C_6D_6) δ 233.4, 168.6, 103.6, 91.8, 91.3, 19.3, 15.9. *(one peak merged with C_6D_6)*

IR (neat, cm^{-1}) 1951, 1855, 1757, 670, 631, 520, 500, 480.

HRMS (APCI) exact mass calcd. for $C_{13}H_{13}CrO_5$ $[M + H]^+$: 301.0163; found: 301.0154.

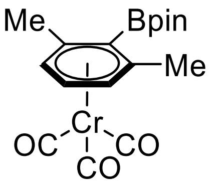

2al

2-(2,6-Dimethylphenyl)-4,4,5,5-tetramethyl-1,3,2-dioxaborolane chromium tricarbonyl (2al): Prepared according to **GP-A** using $Cr(CO)_6$ (1.10 g, 5.0 mmol, 1.0 equiv) and 2-(2,6-dimethylphenyl)-4,4,5,5-tetramethyl-1,3,2-dioxaborolane (1.16 g, 5.0 mmol, 1.0 equiv) for 24 h. The crude reaction mixture was purified by flash column chromatography on silica gel (eluent: 1→20% ethyl acetate in cyclohexane) to afford the title compound **2al** as a yellow solid (1.65 g, 90%).

^{1}H NMR (500 MHz, C_6D_6) δ 4.83 (t, J = 6.5 Hz, 1H), 4.23 (d, J = 6.5 Hz, 2H), 2.15 (s, 6H), 1.01 (s, 12H).

^{13}C NMR (125 MHz, C_6D_6) δ 234.0, 115.1, 95.6, 90.8, 84.2, 24.6, 22.1.

IR (neat, cm^{-1}) 1944, 1824, 1380, 1316, 1141, 1096, 851, 667, 626.

HRMS (APCI) exact mass calcd. for $C_{17}H_{22}BCrO_5$ $[M + H]^+$: 369.0965; found: 369.0986.

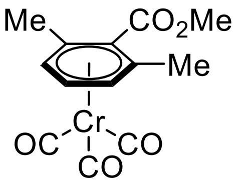

2am

Methyl 2,6-dimethylbenzoate chromium tricarbonyl (2am): Prepared according to **GP-A** using $Cr(CO)_6$ (1.10 g, 5.0 mmol, 1.0 equiv) and methyl 2,6-dimethylbenzoate (0.80 mL, 5.0 mmol, 1.0 equiv) for 24 h. The crude reaction mixture was purified by flash column chromatography on silica gel (eluent: 1→20% ethyl acetate in cyclohexane) to afford the title compound **2am** as a yellow solid (1.36 g, 91%).

^{1}H NMR (400 MHz, C_6D_6) δ 4.62 – 4.51 (m, 1H), 4.12 – 3.95 (m, 2H), 3.28 (s, 3H), 1.86 (s, 6H).

^{13}C NMR (125 MHz, C_6D_6) δ 232.7, 166.6, 108.1, 94.7, 88.5, 52.3, 19.4. *(one peak merged with C_6D_6)*

IR (neat, cm^{-1}) 2961, 1950, 1852, 1727, 1433, 1382, 1103, 798, 626.

HRMS (APCI) exact mass calcd. for $C_{13}H_{13}CrO_5$ $[M + H]^+$: 301.0163; found: 301.0162.

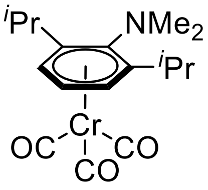

2an

2,6-Diisopropyl-*N,N*-dimethylaniline chromium tricarbonyl (2an): Prepared according to **GP-A** using $Cr(CO)_6$ (1.10 g, 5.0 mmol, 1.0 equiv) and 2,6-diisopropyl-*N,N*-dimethylaniline (1.14 mL, 5.0 mmol, 1.0 equiv) for 24 h. The crude reaction mixture was purified by flash column chromatography on silica gel (eluent: 1→30% ethyl acetate in cyclohexane) to afford the title compound **2an** as a yellow solid (1.49 g, 87%).

^{1}H NMR (400 MHz, C_6D_6) δ 4.65 – 4.59 (m, 2H), 4.58 – 4.53 (m, 1H), 2.89 – 2.71 (m, 5H), 2.34 (s, 3H), 1.20 (d, J = 7.0 Hz, 6H), 0.85 (d, J = 6.8 Hz, 6H).

^{13}C NMR (100 MHz, C_6D_6) δ 234.5, 129.8, 122.9, 91.3, 89.6, 47.8, 44.5, 28.0, 26.1, 23.2.

IR (neat, cm^{-1}) 2961, 1952, 1858, 1697, 1259, 1089, 1014, 798, 631.

HRMS (APCI) exact mass calcd. for $C_{17}H_{24}CrNO_3$ $[M + H]^+$: 342.1156; found: 342.1166.

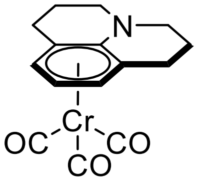

2ao

2,3,6,7-Tetrahydro-*1H,5H*-pyrido[3,2,1-*ij*]quinoline chromium tricarbonyl (2ao): Prepared according to **GP-A** using $Cr(CO)_6$ (1.10 g, 5.0 mmol, 1.0 equiv) and 2,3,6,7-tetrahydro-*1H,5H*-pyrido[3,2,1-*ij*]quinoline (0.87 g, 5.0 mmol, 1.0 equiv) for 36 h. The crude reaction mixture was purified by flash column chromatography on silica gel (eluent: 1→15% ethyl acetate in cyclohexane) to afford the title compound **2ao** as a yellow solid (1.31 g, 85%).

^{1}H NMR (500 MHz, C_6D_6) δ 4.76 (d, J = 6.2 Hz, 2H), 4.22 (t, J = 6.3 Hz, 1H), 2.60 – 2.43 (m, 2H), 2.39 – 2.25 (m, 2H), 2.23 – 2.10 (m, 2H), 1.99 – 1.82 (m, 2H), 1.75 – 1.53 (m, 2H), 1.36 – 1.11 (m, 2H).

^{13}C NMR (125 MHz, C_6D_6) δ 236.6, 129.8, 96.6, 90.5, 82.2, 49.1, 26.7, 21.1.

IR (cm^{-1}) 2961, 1924, 1827, 1543, 1454, 1308, 1208, 1038, 830, 632.

HRMS (APCI) exact mass calcd. for $C_{15}H_{16}CrNO_3$ $[M + H]^+$: 310.0530; found: 310.0529.

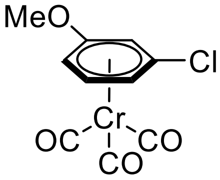

4b

1-Chloro-3-methoxybenzene chromium tricarbonyl (4b): Prepared according to **GP-C** using $Cr(CO)_6$ (1.10 g, 5.0 mmol, 1.0 equiv) and 3-chloroanisole (1.57 mL, 12.5 mmol, 2.5 equiv) for 48 h. The crude reaction mixture was purified by flash column chromatography on silica gel (eluent: 1→20% ethyl acetate in cyclohexane) to afford the title compound **4b** as a yellow solid (653 mg, 47%).

^{1}H NMR (400 MHz, C_6D_6) δ 4.72 (dd, *J* = 1.8, 1.8 Hz, 1H), 4.46 (t, *J* = 6.5 Hz, 1H), 4.23 (dd, *J* = 6.3, 1.5 Hz, 1H), 3.95 (dd, *J* = 6.7, 2.1 Hz, 1H), 2.77 (s, 3H).

^{13}C NMR (100 MHz, C_6D_6) δ 232.6, 142.6, 115.1, 92.9, 85.2, 78.5, 75.1, 55.1.

IR (neat, cm^{-1}) 1950, 1875, 1857, 1545, 1525, 1496, 1247, 1019, 855, 682, 656.

HRMS (EI) exact mass calcd. for $C_{10}H_7ClCrO_4$ [M]$^+$: calculated 277.9433, found 277.9429.

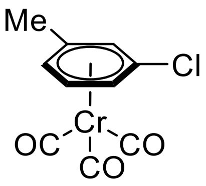

4c

1-Chloro-3-methylbenzene chromium tricarbonyl (4c): Prepared according to **GP-C** using $Cr(CO)_6$ (1.10 g, 5.0 mmol, 1.0 equiv) and 3-chlorotoluene (1.48 mL, 12.5 mmol, 2.5 equiv) for 48 h. The crude reaction mixture was purified by flash column chromatography on silica gel (eluent: 1→10% ethyl acetate in cyclohexane) to afford the title compound **4c** as a yellow solid (643 mg, 49%).

^{1}H NMR (500 MHz, C_6D_6) δ 4.46 – 4.40 (m, 2H), 4.40 – 4.35 (m, 1H), 3.74 (d, *J* = 6.3 Hz, 1H), 1.43 (s, 3H).

^{13}C NMR (125 MHz, C_6D_6) δ 232.5, 114.6, 109.3, 93.7, 91.8, 88.4, 88.3, 19.9.

IR (neat, cm^{-1}) 1951, 1915, 1857, 1518, 1491, 1438, 1377, 832, 685, 624.

HRMS (EI) exact mass calcd. for $C_{10}H_7ClCrO_3$ [M]$^+$: 261.9488, found 261.9482.

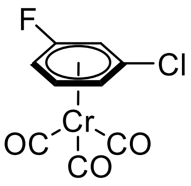

4d

1-Chloro-3-fluorobenzene chromium tricarbonyl (4d): Prepared according to **GP-C** using $Cr(CO)_6$ (11.8 g, 8.0 mmol, 1.0 equiv) and 1-chloro-3-fluorobenzene (2.14 mL, 20.0 mmol, 2.5 equiv) for 48 h. The crude reaction mixture was purified by flash column chromatography

on silica gel (eluent: 1→10% ethyl acetate in cyclohexane) to afford the title compound **4d** as a yellow solid (640 mg, 30%).

^{1}H NMR (500 MHz, C_6D_6) δ 4.58 – 4.53 (m, 1H), 4.19 – 4.12 (m, 1H), 3.96 – 3.90 (m, 2H).

^{13}C NMR (125 MHz, C_6D_6) δ 231.0, 145.9 (d, *J* = 268.4 Hz), 113.4 (d, *J* = 8.4 Hz), 90.9 (d, *J* = 7.6 Hz), 85.9, 79.6 (d, *J* = 22.6 Hz), 75.5 (d, *J* = 20.4 Hz).

^{19}F NMR (471 MHz, C_6D_6) δ -135.5.

IR (neat, cm^{-1}) 1948, 1855, 1597, 1492, 1152, 999, 828, 748, 660, 618.

HRMS (EI) exact mass calcd. for $C_9H_4ClFCrO_3$ $[M]^+$: 265.9238, found 265.9235.

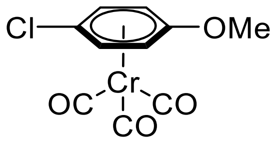

4e

1-Chloro-4-methoxybenzene chromium tricarbonyl (4e): Prepared according to **GP-C** using $Cr(CO)_6$ (1.10 g, 5.0 mmol, 1.0 equiv) and 1-chloro-4-methoxybenzene (1.25 mL, 10.0 mmol, 2.0 equiv) for 48 h. The crude reaction mixture was purified by flash column chromatography on silica gel (eluent: 1→20% ethyl acetate in cyclohexane) to afford the title compound **4e** as a yellow solid (724 mg, 52%).

^{1}H NMR (500 MHz, C_6D_6) δ 4.85 (d, *J* = 6.5 Hz, 2H), 4.13 (d, *J* = 6.5 Hz, 2H), 2.74 (s, 3H).

^{13}C NMR (125 MHz, C_6D_6) 232.3, 140.1, 102.9, 93.7, 77.4, 55.4.

IR (neat, cm^{-1}) 1950, 1885, 1845, 1524, 1435, 1181, 1083, 1000, 795, 677, 616.

HRMS (EI) exact mass calcd. for $C_{10}H_7ClCrO_4$ $[M]^+$: 277.9438, found 277.9432.

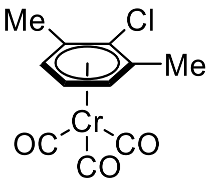

4f

2-Chloro-1,3-dimethylbenzene chromium tricarbonyl (4f): Prepared according to **GP-C** using $Cr(CO)_6$ (1.10 g, 5.0 mmol, 1.0 equiv) and 2-chloro-1,3-dimethylbenzene (1.67 mL, 12.5 mmol, 2.5 equiv) for 48 h. The crude reaction mixture was purified by flash column chromatography on silica gel (eluent: 1→10% ethyl acetate in cyclohexane) to afford the title compound **4f** as a yellow solid (733 mg, 53%).

^{1}H NMR (400 MHz, C_6D_6) δ 4.29 – 4.17 (m, 3H), 1.84 (s, 6H).

^{13}C NMR (100 MHz, C_6D_6) δ 232.9, 111.5, 107.5, 91.6, 91.3, 19.8.

IR (neat, cm^{-1}) 1944, 1848, 1455, 1440, 1254, 1095, 984, 661, 626.

HRMS (APCI) exact mass calcd. for $C_{11}H_{10}ClCrO_3$ $[M + H]^+$: 276.9719, found 276.9714.

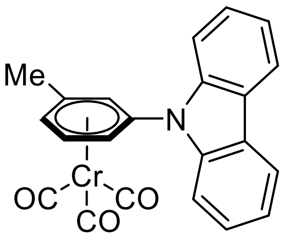

5a

9-(*m*-Tolyl)-9*H*-carbazole chromium tricarbonyl (5a): Prepared according to **GP-D** using (η^6-3-fluorotoluene)$Cr(CO)_3$ (246 mg, 1.0 mmol, 1.0 equiv), carbazole (167 mg, 1.0 mmol, 1.0 equiv) for 3 h. The crude reaction mixture was purified by flash column chromatography on silica gel (eluent: 1→10% ethyl acetate in cyclohexane) to afford the title compound **5a** as a yellow solid (216 mg, 55%).

^{1}H NMR (400 MHz, $(CD_3)_2CO$) δ 8.20 (d, J = 7.8 Hz, 2H), 7.99 (d, J = 8.4 Hz, 2H), 7.57 – 7.47 (m, 2H), 7.40 – 7.30 (m, 2H), 6.14 (s, 1H), 6.11 – 6.02 (m, 2H), 5.61 – 5.53 (m, 1H), 2.44 (s, 3H).

^{13}C NMR (100 MHz, $(CD_3)_2CO$) δ 234.2, 140.6, 127.2, 125.2, 122.1, 121.2, 117.5, 112.6, 111.5, 95.7, 93.0, 92.3, 89.4, 20.8.

IR (neat, cm^{-1}) 1951, 1878, 1858, 1616, 1519, 1380, 1237, 928, 662.

HRMS (EI) exact mass calcd. for $C_{22}H_{15}NCrO_3$ [M]$^+$:393.0457, found 393.0455.

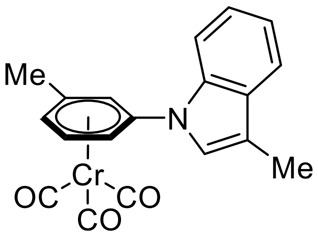

5b

3-Methyl-1-(*m*-tolyl)-1*H*-indole chromium tricarbonyl (5b): Prepared according to **GP-D** using (η^6-3-fluorotoluene)Cr(CO)$_3$ (197 mg, 0.8 mmol, 1.0 equiv), 3-methyl-1*H*-indole (105 mg, 0.8 mmol, 1.0 equiv) for 5 h. The crude reaction mixture was purified by flash column chromatography on silica gel (eluent: 1→10% ethyl acetate in cyclohexane) to afford the title compound **5b** as a yellow solid (171 mg, 60%).

^{1}H NMR (500 MHz, $(CD_3)_2CO$) δ 7.79 (d, *J* = 8.3 Hz, 1H), 7.58 (d, *J* = 7.7 Hz, 1H), 7.40 (s, 1H), 7.31 – 7.24 (m, 1H), 7.23 – 7.15 (m, 1H), 6.06 – 5.99 (m, 2H), 5.96 (d, *J* = 6.9 Hz, 1H), 5.36 (d, *J* = 6.4 Hz, 1H), 2.40 (s, 3H), 2.32 (s, 3H).

^{13}C NMR (125 MHz, $(CD_3)_2CO$) δ 234.5, 136.8, 131.8, 126.1, 123.9, 121.8, 120.8, 120.3, 115.3, 112.5, 112.4, 96.7, 91.4, 89.2, 85.8, 20.9, 9.6.

IR (neat, cm^{-1}) 1951, 1855, 1732, 1611, 1534, 1349, 1155, 755, 623.

HRMS (LIFDI) exact mass calcd. for $C_{19}H_{15}NCrO_3$ [M]$^+$: 357.0452, found 357.0445.

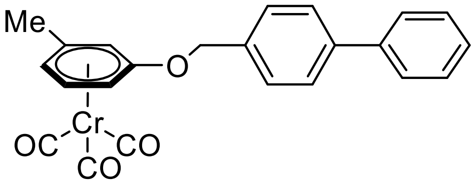

5c

4-((*m*-Tolyloxy)methyl)-1,1'-biphenyl chromium tricarbonyl (5c): Prepared according to **GP-D** using (η^6-3-fluorotoluene)Cr(CO)$_3$ (246 mg, 1.0 mmol, 1.0 equiv), [1,1'-biphenyl]-4-methanol (184 mg, 1.0 mmol, 1.0 equiv) for 1 h. The crude reaction mixture was purified by

flash column chromatography on silica gel (eluent: 1→25% dichloromethane in cyclohexane) to afford the title compound **5c** as a yellow solid (172 mg, 42%).

^{1}H NMR (400 MHz, $(CD_3)_2CO$) δ 7.70 (dd, *J* = 10.5, 7.8 Hz, 4H), 7.58 (d, *J* = 7.8 Hz, 2H), 7.52 – 7.43 (m, 2H), 7.38 (t, *J* = 7.3 Hz, 1H), 5.93 – 5.86 (m, 1H), 5.55 (s, 1H), 5.46 (dd, *J* = 7.1, 2.2 Hz, 1H), 5.13 – 5.07 (m, 3H), 2.29 (s, 3H).

^{13}C NMR (125 MHz, $(CD_3)_2CO$) δ 235.3, 144.5, 142.1, 141.3, 135.6, 129.8 (2C), 128.4, 127.9, 127.8, 113.2, 97.5, 88.7, 82.9, 78.7, 71.2, 20.9.

IR (neat, cm^{-1}) 1940, 1848, 1743, 1607, 1517, 1410, 1281, 819, 626.

HRMS (APCI) exact mass calcd. for $C_{23}H_{19}CrO_4$ $[M + H]^+$: 411.0683, found 411.0684.

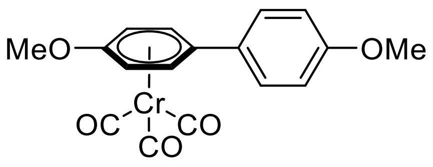

7a

4,4'-Dimethoxy-1,1'-biphenyl chromium tricarbonyl (7a): Prepared according to **GP-E** using (η^6-4-chloroanisole)$Cr(CO)_3$ (279 mg, 1.0 mmol, 1.0 equiv), (4-methoxyphenyl)boronic acid (182 mg, 1.2 mmol, 1.2 equiv) for 5 h at 50 °C. The crude reaction mixture was purified by flash column chromatography on silica gel (eluent: 1→10% ethyl acetate in cyclohexane) to afford the title compound **7a** as a yellow solid (221 mg, 63%).

^{1}H NMR (500 MHz, $(CD_3)_2CO$) δ 7.54 (d, *J* = 8.8 Hz, 2H), 6.96 (d, *J* = 8.8 Hz, 2H), 6.27 (d, *J* = 7.1 Hz, 2H), 5.60 (d, *J* = 7.2 Hz, 2H), 3.83 (s, 3H), 3.80 (s, 3H).

^{13}C NMR (125 MHz, $(CD_3)_2CO$) δ 234.8, 161.0, 144.1, 129.2, 128.7, 115.0, 105.0, 96.0, 80.1, 56.5, 55.7.

IR (neat, cm^{-1}) 1941, 1852, 1607, 1580, 1465, 1194, 1096, 928, 826, 624.

HRMS (LIFDI) exact mass calcd. for $C_{17}H_{14}CrO_5$ $[M]^+$: 350.0241, found 350.0234.

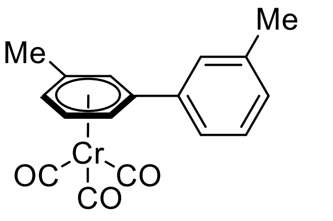

7b

3,3'-Dimethyl-1,1'-biphenyl chromium tricarbonyl (7b): Prepared according to **GP-E** using (η^6-3-chlorotoluene)Cr(CO)$_3$ (263 mg, 1.0 mmol, 1.0 equiv) and (3-methylphenyl)boronic acid (163 mg, 1.2 mmol, 1.2 equiv) for 5 h at 50 °C. The crude reaction mixture was purified by flash column chromatography on silica gel (eluent: 1→10% ethyl acetate in cyclohexane) to afford the title compound **7b** as a yellow solid (315 mg, 99%).

^{1}H NMR (400 MHz, C_6D_6) δ 7.09 (s, 1H),7.07 – 7.01 (m, 2H), 6.97 – 6.89 (m, 1H), 4.95 (s, 1H), 4.88 – 4.78 (m, 1H), 4.78 – 4.72 (m, 1H), 4.29 (d, *J* = 6.2 Hz, 1H), 2.11 (s, 3H), 1.69 (s, 3H).

^{13}C NMR (100 MHz, C_6D_6) δ 233.8, 138.4, 136.9, 130.1, 128.9, 128.2, 124.9, 112.3, 109.3, 94.3, 92.3, 90.9, 88.9, 21.3, 20.5.

IR (neat, cm^{-1}) 1940, 1858, 1842, 1727, 1478, 1212, 1093, 834, 623.

HRMS (APCI) exact mass calcd. for $C_{17}H_{14}CrO_5$ [M + H]$^+$: 319.0421, found 319.0425.

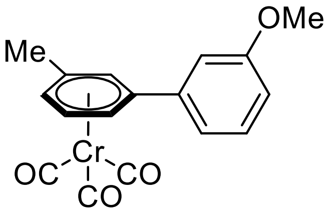

7c

3-Methoxy-3'-methyl-1,1'-biphenyl chromium tricarbonyl (7c): Prepared according to **GP-E** using (3-chlorotoluene)Cr(CO)$_3$ (263 mg, 1.0 mmol, 1.0 equiv) and (η^6-3-methoxyphenyl)boronic acid (182 mg, 1.2 mmol, 1.2 equiv) for 5 h at 50 °C. The crude reaction mixture was purified by flash column chromatography on silica gel (eluent: 1→15% ethyl acetate in cyclohexane) to afford the title compound **7c** as a yellow solid (314 mg, 94%).

^{1}H NMR (500 MHz, $(CD_3)_2CO$) δ 7.40 – 7.33 (m, 1H), 7.27 – 7.21 (m, 2H), 7.01 – 6.95 (m, 1H), 6.02 – 5.97 (m, 1H), 5.93 – 5.85 (m, 2H), 5.52 (d, *J* = 6.1 Hz, 1H), 3.86 (s, 3H), 2.33 (s, 3H).

^{13}C NMR (125 MHz, $(CD_3)_2CO$) δ 234.9, 160.9, 138.9, 130.7, 120.3, 115.5, 113.7, 113.0, 111.7, 96.4, 94.5, 93.9, 91.2, 55.7, 20.9.

IR (neat, cm^{-1}) 1948, 1852, 1732, 1581, 1383, 1276, 1033, 799, 620.

HRMS (EI) exact mass calcd. for $C_{17}H_{14}CrO_4$ $[M]^+$: 334.0292, found 334.0291.

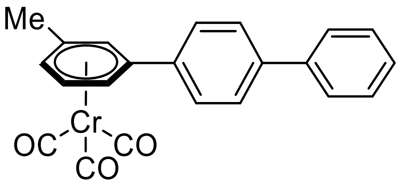

7d

3-Methyl-1,1':4',1''-terphenyl chromium tricarbonyl (7d): Prepared according to **GP-E** using (η^6-3-chlorotoluene)$Cr(CO)_3$ (263 mg, 1.0 mmol, 1.0 equiv) and [1,1'-biphenyl]-4-ylboronic acid (238 mg, 1.2 mmol, 1.2 equiv) for 5 h at 50 °C. The crude reaction mixture was purified by flash column chromatography on silica gel (eluent: 1→15% ethyl acetate in cyclohexane) to afford the title compound **7d** as a yellow solid (232 mg, 61%).

^{1}H NMR (500 MHz, $(CD_3)_2CO$) δ 7.80 – 7.74 (m, 4H), 7. – 7.69 (m, 2H) 7.53 – 7.45 (m, 2H), 7.43 – 7.33 (m, 1H), 6.08 – 6.01 (m, 1H), 6.00 – 5.89 (m, 2H), 5.57 – 5.51 (m, 1H), 2.36 (s, 3H).

^{13}C NMR (125 MHz, $(CD_3)_2CO$) δ 234.9, 142.5, 140.8, 136.4, 129.8, 128.6, 128.5, 128.0, 127.7, 112.4, 111.8, 96.5, 94.2, 93.8, 90.9, 20.9.

IR (neat, cm^{-1}) 1941, 1871, 1852, 1561, 1451, 1125, 829, 624.

HRMS (APCI) exact mass calcd. for $C_{22}H_{17}CrO_3$ $[M + H]^+$: 381.0582, found 381.0586.

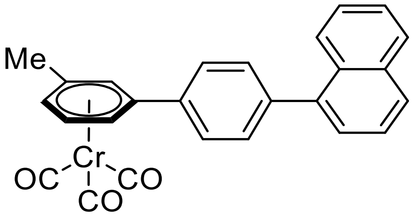

7e

1-(3'-Methyl-[1,1'-biphenyl]-4-yl)naphthalene chromium tricarbonyl (7e): Prepared according to **GP-E** using (η^6-3-chlorotoluene)$Cr(CO)_3$ (263 mg, 1.0 mmol, 1.0 equiv) and (4-(naphthalen-1-yl)phenyl)boronic acid (298 mg, 1.2 mmol, 1.2 equiv) for 5 h at 50 °C. The crude reaction mixture was purified by flash column chromatography on silica gel (eluent: 1→10% ethyl acetate in cyclohexane) to afford the title compound **7e** as a yellow solid (229 mg, 53%).

^{1}H NMR (400 MHz, $(CD_3)_2CO$) δ 7.99 (dd, *J* = 14.4, 8.1 Hz, 2H), 7.93 – 7.81 (m, 3H), 7.64 – 7.44 (m, 6H), 6.11 (s, 1H), 6.02 (d, *J* = 6.6 Hz, 1H), 5.95 (t, *J* = 6.5 Hz, 1H), 5.57 (d, *J* = 6.4 Hz, 1H), 2.38 (s, 3H).

^{13}C NMR (100 MHz, $(CD_3)_2CO$) δ 234.9, 142.3, 140.1, 136.5, 134.9, 132.2, 131.3, 131.1, 129.3, 128.9, 128.1, 127.8 (2C), 127.2, 126.8, 126.4, 126.3, 112.5, 111.8, 96.5, 94.3, 93.8, 91.0, 20.9.

IR (neat, cm^{-1}) 1963, 1953, 1878, 1851, 775, 663, 631, 585, 522.

HRMS (APCI) exact mass calcd. for $C_{26}H_{19}CrO_3$ $[M + H]^+$: 431.0734, found 431.0721.

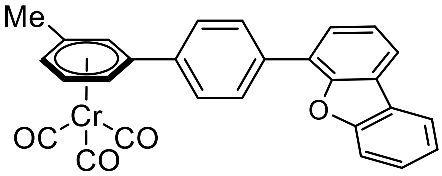

7f

4-(3'-Methyl-[1,1'-biphenyl]-4-yl)dibenzo[b,d]furan chromium tricarbonyl (7f): Prepared according to **GP-E** using (η^6-3-chlorotoluene)$Cr(CO)_3$ (263 mg, 1.0 mmol, 1.0 equiv) and (4-(dibenzo[*b*,*d*]furan-4-yl)phenyl)boronic acid (346 mg, 1.2 mmol, 1.2 equiv) for 7 h at 50 °C. The crude reaction mixture was purified by flash column chromatography on silica gel (eluent: 1→15% ethyl acetate in cyclohexane) to afford the title compound **7f** as a yellow solid (263 mg, 56%).

^{1}H NMR (400 MHz, $(CD_3)_2CO$) δ 8.17 (ddd, *J* = 7.7, 1.4, 0.7 Hz, 1H), 8.15 (dd, *J* = 7.7, 1.2 Hz, 1H), 8.08 (d, *J* = 8.5 Hz, 2H), 7.89 (d, *J* = 8.5 Hz, 2H), 7.78 (dd, *J* = 7.6, 1.2 Hz, 1H), 7.70 (dt, *J* = 8.3, 0.9 Hz, 1H), 7.59 – 7.50 (m, 2H), 7.44 (td, *J* = 7.5, 1.0 Hz, 1H), 6.12 (t, *J* = 1.6 Hz, 1H), 6.07 – 6.02 (m, 1H), 5.95 (t, *J* = 6.5 Hz, 1H), 5.57 (d, *J* = 6.4 Hz, 1H), 2.38 (s, 3H).

^{13}C NMR (100 MHz, $(CD_3)_2CO$) δ 234.9, 157.0, 137.9, 136.9, 129.8, 128.6, 128.3, 127.7, 125.9, 125.6, 124.9, 124.6, 124.1, 121.9, 121.3, 112.6, 112.3, 111.8, 96.5, 94.3, 93.9, 91.0, 21.0.

IR (neat, cm^{-1}) 1954, 1865, 1608, 1506, 1462, 1346, 1258, 855, 752, 626.

HRMS (EI) exact mass calcd. for $C_{28}H_{18}CrO_4$ $[M]^+$: 470.0610, found 470.0599.

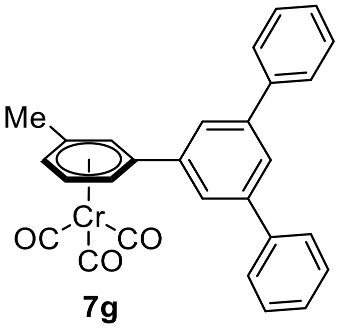

7g

3-Methyl-5'-phenyl-1,1':3',1''-terphenyl chromium tricarbonyl (7g): Prepared according to **GP-E** using (η^6-3-chlorotoluene)$Cr(CO)_3$ (26 mg, 0.1 mmol, 1.0 equiv) and [1,1':3',1''-terphenyl]-5'-ylboronic acid (33 mg, 0.12 mmol, 1.2 equiv) for 16 h at 70 °C. Similar type of 10 sets of reaction (in 0.1 mmol scale) were performed and the collective crude reaction mixture (from 10 reaction sets) was purified by flash column chromatography on silica gel (eluent: 1→15% ethyl acetate in cyclohexane) to afford the title compound **7g** as a yellow solid (342 mg, 75%; collective total yield). *However, the reaction in 1.0 mmol scale delivered very low yield. We are still working on it to optimize the reaction.*

^{1}H NMR (400 MHz, $(CD_3)_2CO$) δ 7.94 (s, 3H), 7.86 – 7.82 (m, 4H), 7.55 – 7.48 (m, 4H), 7.46 – 7.39 (m, 2H), 6.25 (t, *J* = 1.6 Hz, 1H), 6.15 (dt, *J* = 6.5, 1.3 Hz, 1H), 5.96 (t, *J* = 6.5 Hz, 1H), 5.56 (d, *J* = 6.4 Hz, 1H), 2.37 (s, 3H).

^{13}C NMR (100 MHz, $(CD_3)_2CO$) δ 235.0, 143.1, 141.3, 138.9, 129.8, 128.7, 128.2, 127.2, 126.0, 113.3, 112.1, 96.7, 94.7, 93.9, 91.4, 20.9.

IR (neat, cm^{-1}) 1951, 1859, 1594, 1575, 1496, 1412, 878, 755, 624.

HRMS (EI) exact mass calcd. for $C_{28}H_{20}CrO_3$ $[M]^+$: 456.0817, found 456.0807.

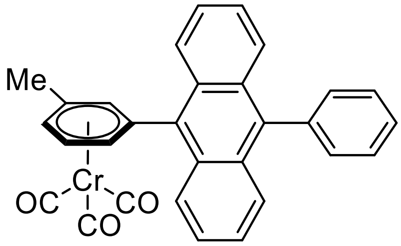

7h

9-Phenyl-10-(*m*-tolyl)anthracene chromium tricarbonyl (7h): Prepared according to **GP-E** using (η^6-3-chlorotoluene)$Cr(CO)_3$ (263 mg, 1.0 mmol, 1.0 equiv) and (10-phenylanthracen-9-yl)boronic acid (358 mg, 1.2 mmol, 1.2 equiv) for 5 h at 50 °C. The crude reaction mixture was purified by flash column chromatography on silica gel (eluent: 1→10% ethyl acetate in cyclohexane) to afford the title compound **7h** as a red solid (430 mg, 90%).

^{1}H NMR (400 MHz, $(CD_3)_2CO$) δ 9.55 (d, *J* = 9.0 Hz, 1H), 8.09 (d, *J* = 8.9 Hz, 1H), 7.69 – 7.58 (m, 6H), 7.53 – 7.36 (m, 5H), 6.07 (s, 1H), 6.01 – 5.93 (m, 3H), 2.42 (s, 3H).

^{13}C NMR (100 MHz, $(CD_3)_2CO$) δ 234.5, 140.3, 139.5, 132.6, 131.7, 130.7 (2C), 129.9, 129.5, 128.8 (2C), 128.1, 128.0, 127.2, 126.7, 126.6, 126.2, 126.1, 125.8, 110.9, 108.9, 102.5, 99.5, 96.9, 93.5, 20.8.

IR (neat, cm^{-1}) 1956, 1894, 1854, 769, 704, 694, 664, 659, 628.

HRMS (EI) exact mass calcd. for $C_{30}H_{20}CrO_3$ $[M]^+$: 480.0812, found 480.0808.

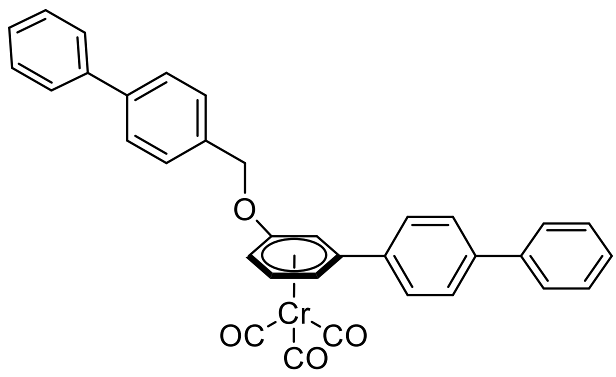

7i

3-([1,1'-Biphenyl]-4-ylmethoxy)-1,1':4',1''-terphenyl chromium tricarbonyl (7i): Prepared in a step-wise manner according to **GP-E**, followed by **GP-D**.

Step I: Inside glovebox, an oven-dried screw-capped vial equipped with a magnetic stir bar was charged with (η^6-1-chloro-3-fluorobenzene)$Cr(CO)_3$ (213 mg, 0.8 mmol, 1.0 equiv), [1,1'-biphenyl]-4-ylboronic acid (190 mg, 0.96 mmol, 1.2 equiv), Cs_2CO_3 (592 mg, 1.76 mmol, 2.2 equiv), $Pd(PPh_3)_4$ (48 mg, 0.04 mmol, 5 mol%), and dry 1,4-dioxane (4.2 mL, 0.2 M). The resulting heterogeneous, yellow-colored mixture was then taken out from the glovebox and allowed to stir at 50 °C for 5 h. After that the reaction mixture was cooled down to room temperature and filtered over a short pad of silica gel (rinsed with EtOAc for three times). The combined reaction mixture was concentrated under reduced pressure. This crude residue was used in the subsequent step without further purification.

Step II: An oven-dried screw-capped vial equipped with a magnetic stir bar was charged with NaH (1.2 equiv) and dry DMF. Then the solution of [1,1'-biphenyl]-4-methanol (147 mg, 0.8 mmol, 1.0 equiv) dissolved in dry DMF was added dropwise to the slurry of NaH at 0 ^oC under argon atmosphere. After 20 min, solution of crude (η^6-arene)$Cr(CO)_3$ complex from previous reaction step in dry DMF was added slowly. The resulting mixture was stirred for 30 minutes and then left at room temperature for 6 h. After completion of the reaction, the mixture was then treated with saturated brine solution and extracted with Et_2O (three times). The combined organic layer was washed with water and dried over anhy. Na_2SO_4. The filtrate was concentrated under reduced pressure. The crude reaction mixture was purified by flash column chromatography on silica gel (eluent: 1→20% ethyl acetate in cyclohexane) to afford the title compound **7i** as a yellow solid (175 mg, 40%, overall yield).

^{1}H NMR (400 MHz, $(CD_3)_2CO$) δ 7.88 – 7.81 (m, 2H), 7.80 – 7.76 (m, 2H) 7.75 – 7.68 (m, 6H), 7.64 (d, *J* = 7.8 Hz, 2H), 7.53 – 7.45 (m, 4H), 7.43 – 7.35 (m, 2H), 6.14 – 6.08 (m, 2H), 5.72 (d, *J* = 6.3 Hz, 1H), 5.64 (dd, *J* = 6.9, 2.3 Hz, 1H), 5.29 – 5.20 (m, 2H).

^{13}C NMR (100 MHz, $(CD_3)_2CO$) δ 235.0, 144.2, 142.7, 142.1, 141.3, 140.8, 136.2, 135.6, 129.9 (2C), 129.8, 128.8, 128.7, 128.4, 128.0 (2C), 127.8, 127.7, 113.4, 97.3, 86.8, 80.4, 79.6, 71.5.

IR (neat, cm^{-1}) 1957, 1880, 1861, 1568, 1373, 1042, 822, 628.

HRMS (APCI) exact mass calcd. for $C_{34}H_{25}CrO_4$ $[M + H]^+$: 549.1158, found 549.1169.

2.7. Unsuccessful substrates *via* direct π-arene complexation

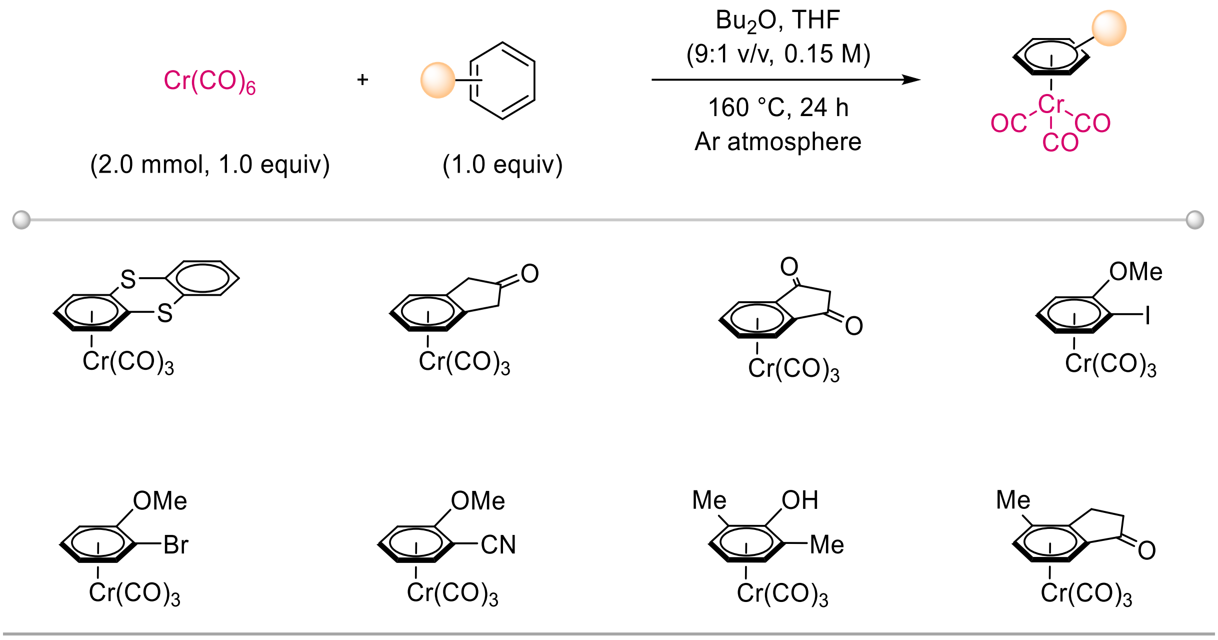

Note S3: The complexation of 2-methoxybenzonitrile with $Cr(CO)_6$ did not yield the desired (η^6-2-methoxybenzonitrile)$Cr(CO)_3$ complex. Instead, the thermolysis process predominantly produced the pentacarbonyl 2-methoxybenzonitrile chromium (**8**), which was successfully isolated.

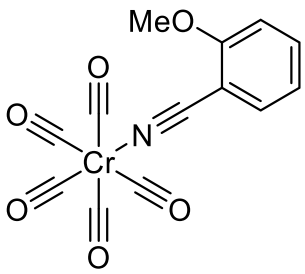

8

Pentacarbonyl 2-methoxybenzonitrile chromium (8):

^{1}H NMR (400 MHz, C_6D_6) δ 6.73 (t, *J* = 8.1 Hz, 1H), 6.44 (d, *J* = 7.7 Hz, 1H), 6.21 (t, *J* = 7.6 Hz, 1H), 5.92 (d, *J* = 8.6 Hz, 1H), 2.88 (s, 3H).

HRMS (EI) exact mass calcd. for $C_{13}H_7CrNO_6$ $[M]^+$: 324.9678, found 324.9680.

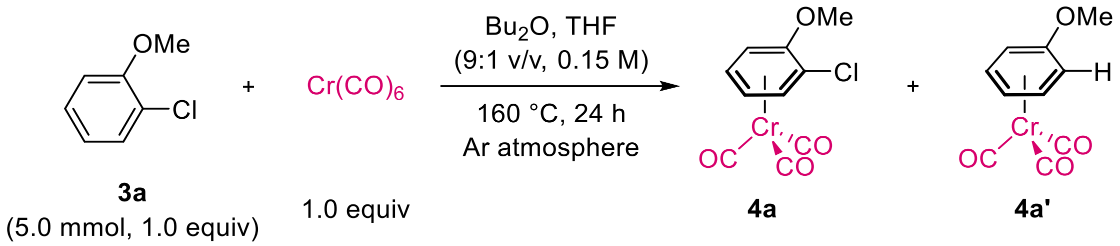

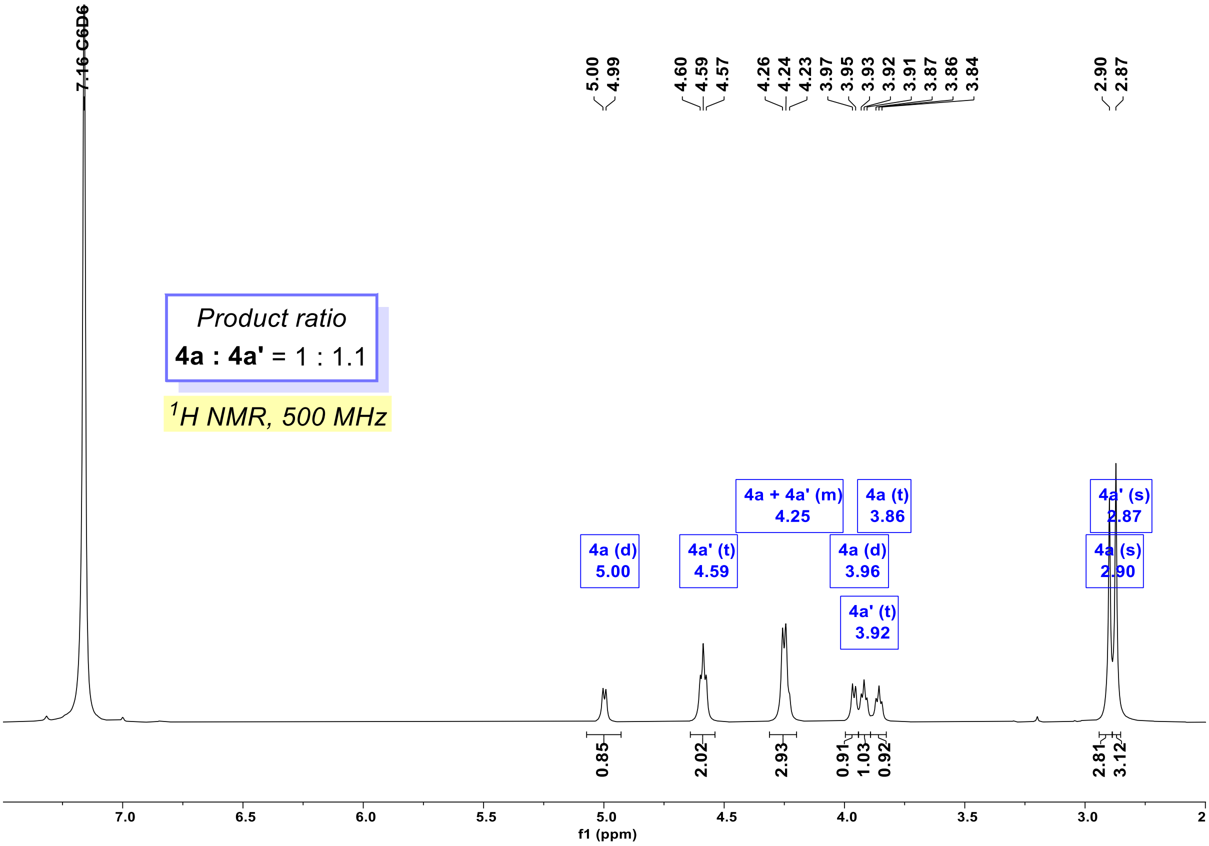

Figure S3. Direct synthetic challenge for (η^6-2-chloroanisole)$Cr(CO)_3$ complex.

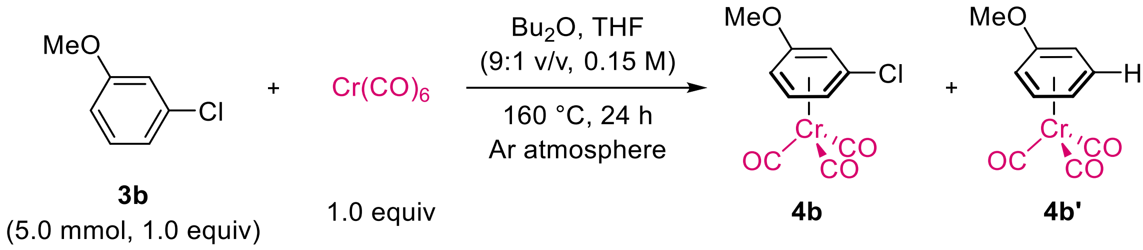

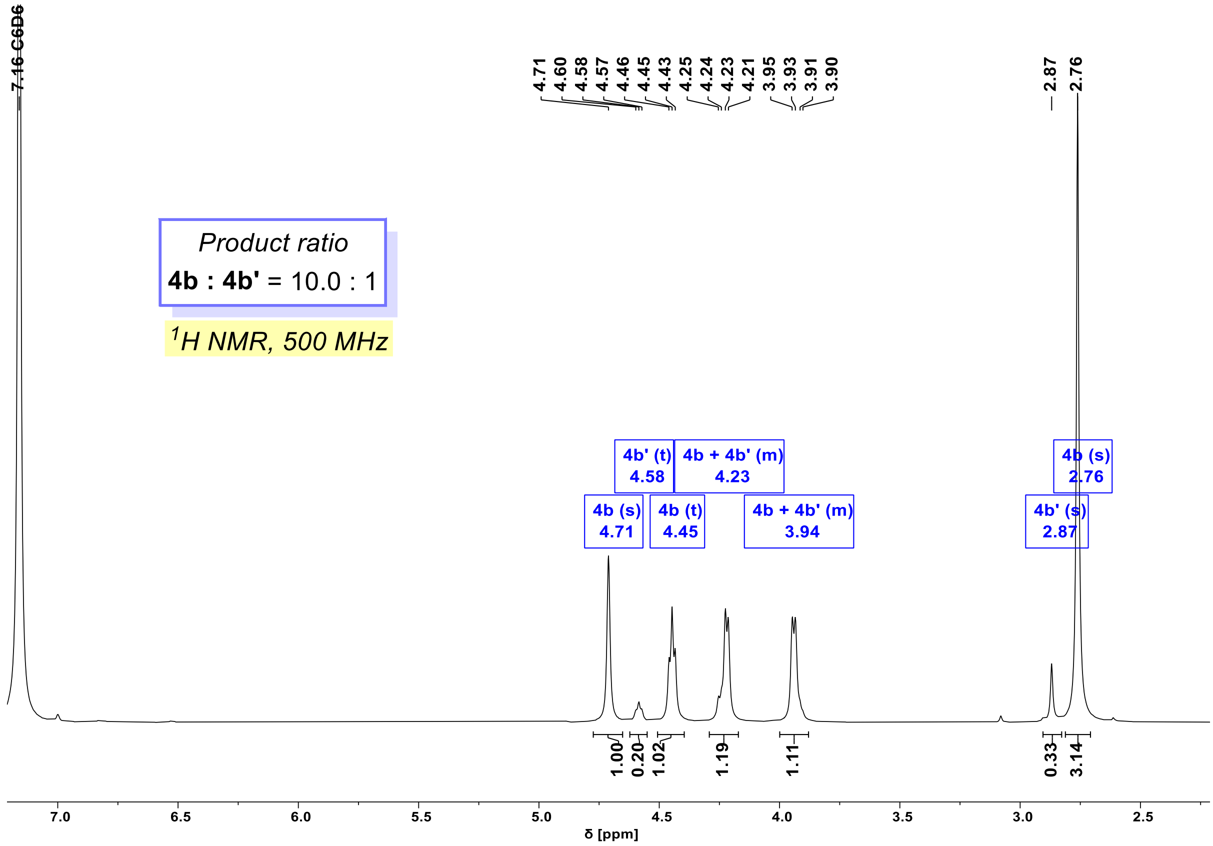

Figure S4. Direct synthetic challenge for (η^6-3-chloroanisole)$Cr(CO)_3$ complex (where **4b′** = **4a′**).

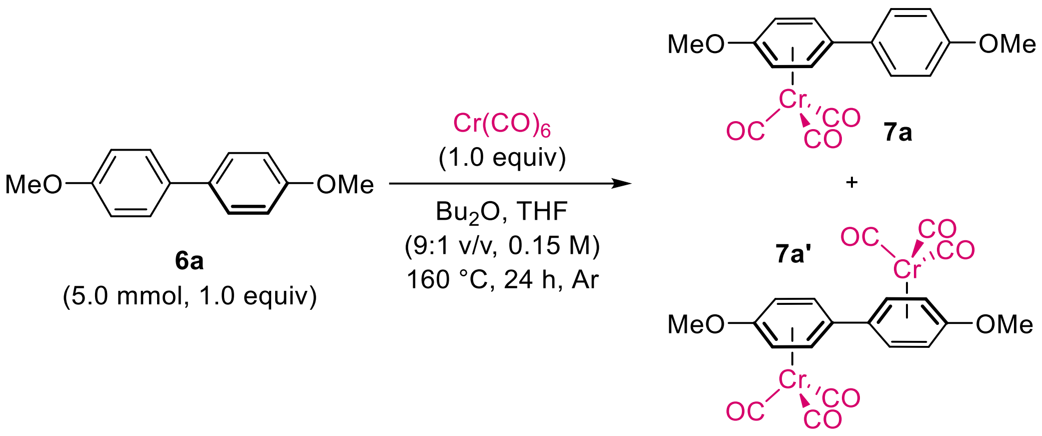

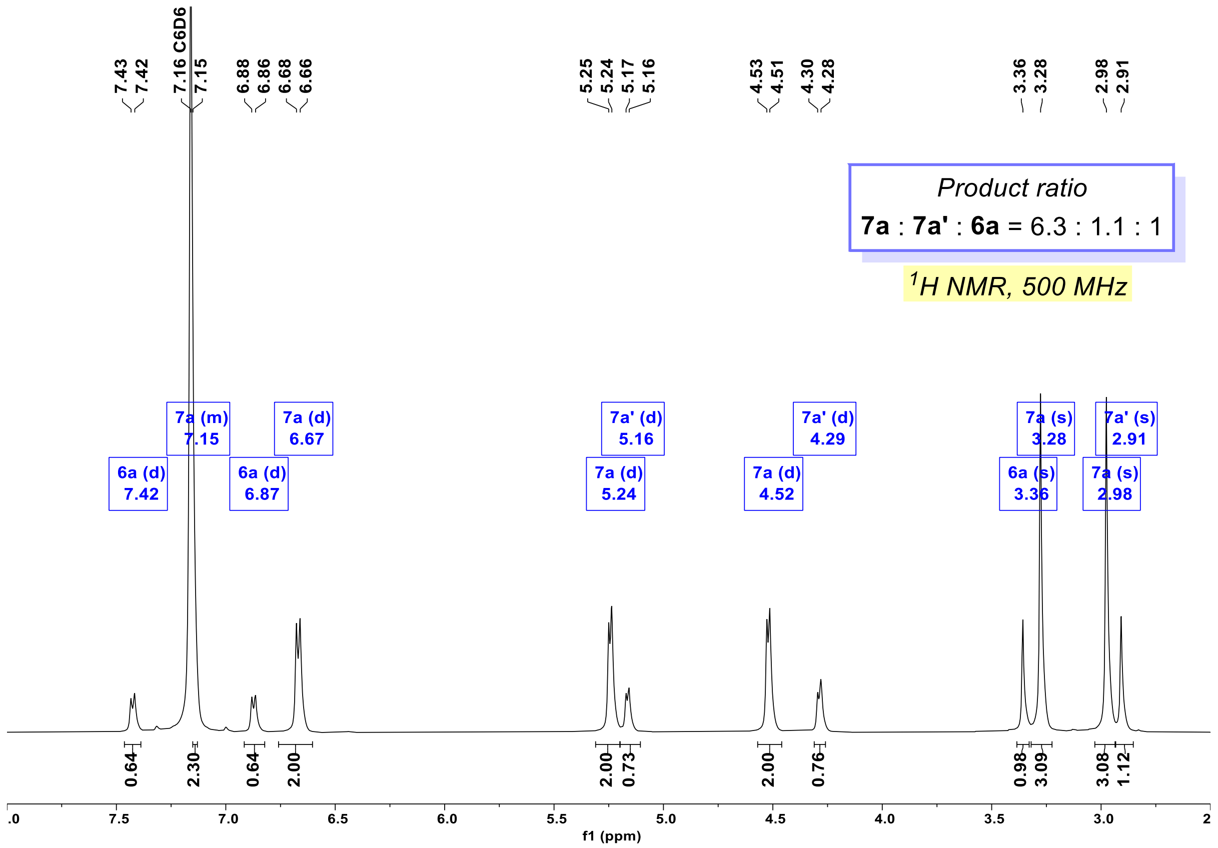

Figure S5. Direct synthetic challenge for mono-selective $Cr(CO)_3$ complexation of 4,4'-dimethoxy-1,1'-biphenyl.

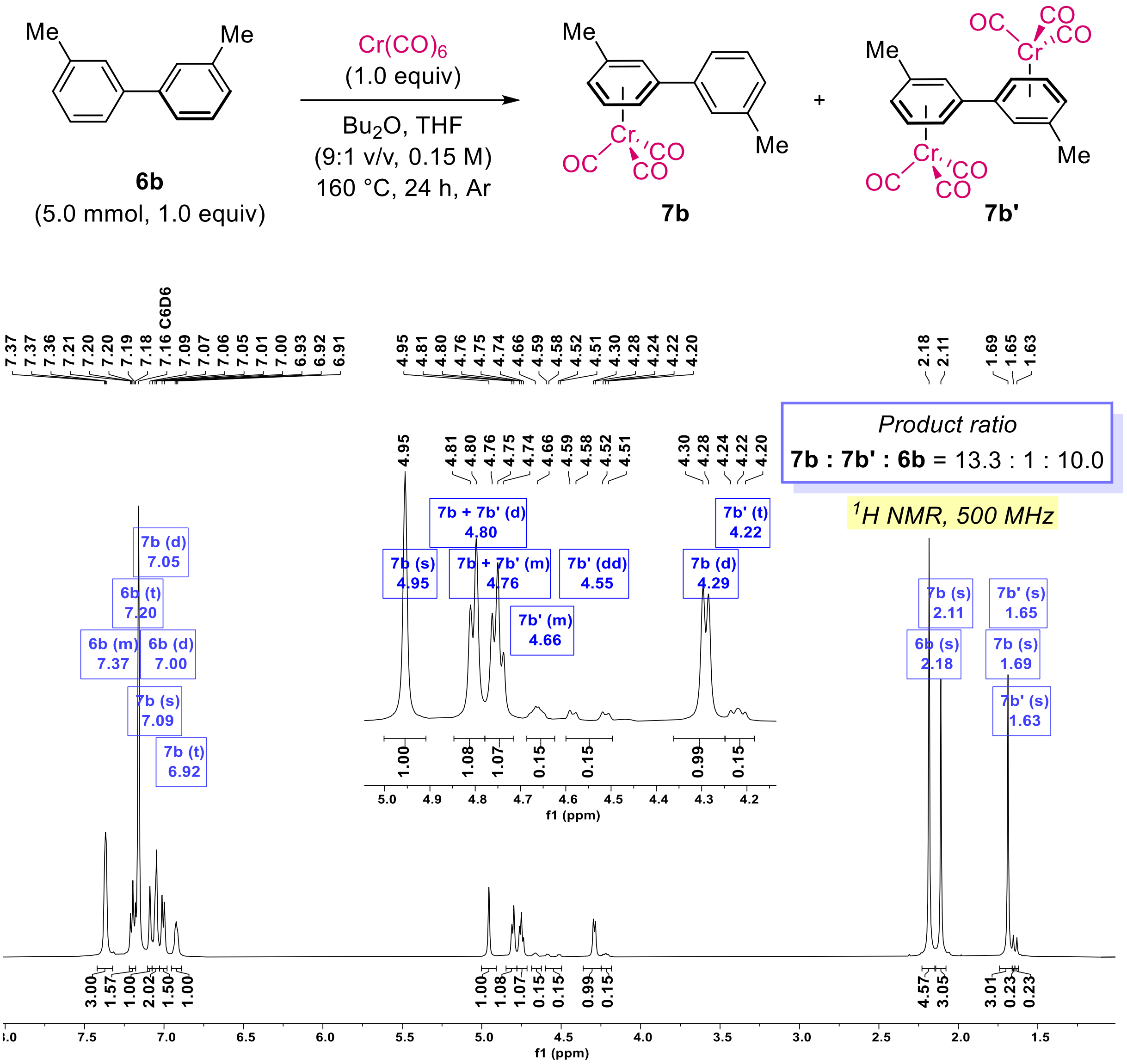

Figure S6. Direct synthetic challenge for mono-selective $Cr(CO)_3$ complexation of 3,3'-dimethyl-1,1'-biphenyl.

2.8. Removal of chromium tricarbonyl fragment from the *π*-arene complex through light irradiation (GP-F)

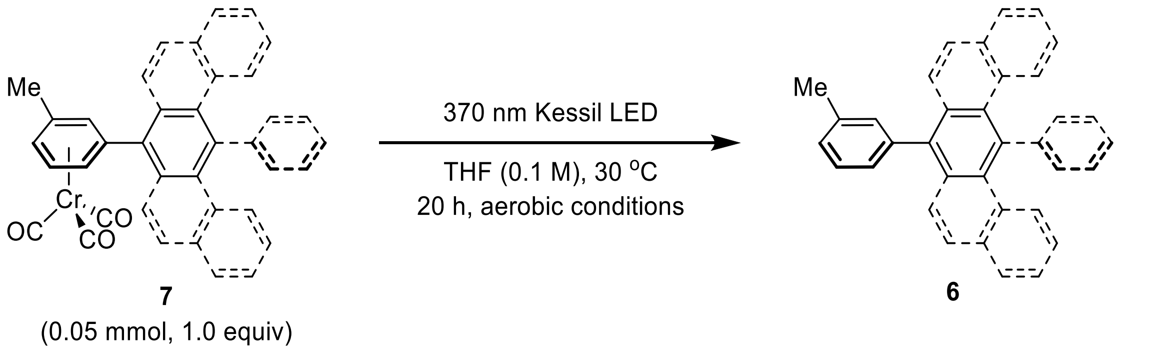

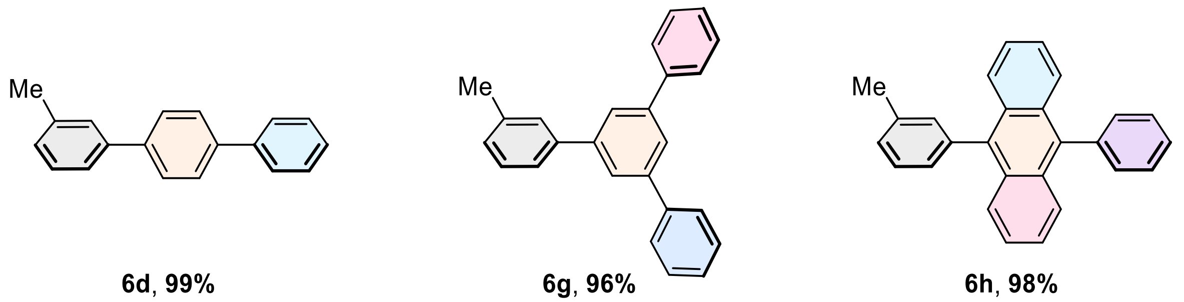

6d, **99%** **6g**, **96%** **6h**, **98%**

An oven-dried 4.0 mL screw-capped reaction vial equipped with a magnetic stir bar was charged with corresponding (η^6-arene)$Cr(CO)_3$ complex **7** (0.05 mmol, 1.0 equiv) and synthetic grade THF. Then the vial was closed with cap under aerobic conditions and irradiated with 370 nm Kessil LEDs for 20 hours. After the specified reaction time, the reaction mixture was concentrated and filtered over a short pad of celite (rinsed with EtOAc for three times). The combined reaction mixture was concentrated under reduced pressure with the aid of a rotary evaporator. The crude residue was then purified by flash column chromatography to provide the corresponding polyarenes **6** in high yields.

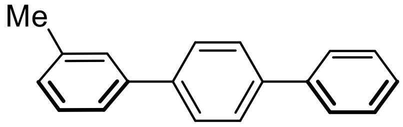

6d

3-Methyl-1,1':4',1''-terphenyl (6d): Prepared according to **GP-F** using 3-methyl-1,1':4',1''-terphenyl chromium tricarbonyl (19.0 mg, 0.05 mmol). The crude reaction mixture was purified by flash column chromatography on silica gel (eluent: 0→5% ethyl acetate in hexane) to afford the title compound **6d** as an off-white solid (12.1 mg, 99%).

^{1}H NMR (400 MHz, Acetone-d$_6$) δ 7.75 (s, 4H), 7.73 – 7.69 (m, 2H), 7.55 – 7.53 (m, 1H), 7.52 – 7.45 (m, 3H), 7.40 – 7.33 (m, 2H), 7.23 – 7.17 (m, 1H), 2.41 (s, 3H).

^{13}C NMR (100 MHz, Acetone-d$_6$) δ 141.3 (2C), 140.9, 140.7, 139.2, 129.8, 129.7, 129.0, 128.3 (2C), 128.2, 128.1, 127.6, 124.8, 21.5.

IR (neat, cm^{-1}) 1478, 1393, 836, 788, 758, 687, 580.

HRMS (APCI) exact mass calcd. for $C_{19}H_{16}$ $[M]^+$: 244.1247, found 244.1237.

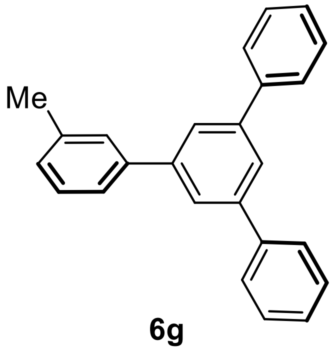

6g

3-Methyl-5'-phenyl-1,1':3',1''-terphenyl (6g): Prepared according to **GP-F** using 3-methyl-5'-phenyl-1,1':3',1''-terphenyl chromium tricarbonyl (22.8 mg, 0.05 mmol). The crude reaction mixture was purified by flash column chromatography on silica gel (eluent: 0→5% ethyl acetate in hexane) to afford the title compound **6g** as an off-white solid (15.4 mg, 96%).

^{1}H NMR (300 MHz, Acetone-d$_6$) δ 7.89 (s, 3H), 7.87 – 7.81 (m, 4H), 7.70 – 7.67 (m, 1H), 7.66 – 7.61 (m, 1H), 7.55 – 7.48 (m, 4H), 7.45 – 7.36 (m, 3H), 7.28 – 7.20 (m, 1H), 2.44 (s, 3H).

^{13}C NMR (125 MHz, Acetone-d$_6$) δ 143.4, 143.2, 141.9, 141.8, 139.3, 129.8, 129.7, 129.2, 128.9, 128.5, 128.2, 125.6, 125.5, 125.3, 21.5.

IR (neat, cm^{-1}) 1594, 1496, 1413, 875, 785, 755, 697.

HRMS (APCI) exact mass calcd. for $C_{25}H_{20}$ $[M]^+$: 320.1560, found 320.1554.

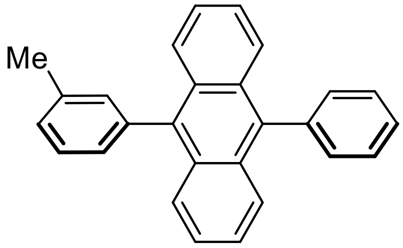

6h

9-Phenyl-10-(*m*-tolyl)anthracene (6h): Prepared according to **GP-F** using 9-phenyl-10-(*m*-tolyl)anthracene chromium tricarbonyl (24.0 mg, 0.05 mmol). The crude reaction mixture was purified by flash column chromatography on silica gel (eluent: 0→5% ethyl acetate in hexane) to afford the title compound **6h** as a light-yellow solid (16.9 mg, 98%).

^{1}H NMR (400 MHz, $CDCl_3$) δ ^{1}H NMR (400 MHz, $CDCl_3$) δ 7.77 – 7.69 (m, 4H), 7.65 – 7.59 (m, 2H), 7.59 – 7.54 (m, 1H), 7.54 – 7.48 (m, 3H), 7.41 – 7.29 (m, 7H), 2.51 (s, 3H).

^{13}C NMR (100 MHz, $CDCl_3$) δ 139.3, 139.1, 138.1, 137.5, 137.1, 132.1, 131.5, 130.0, 128.5 (2C), 128.4, 128.3, 127.6, 127.2, 127.1, 125.1 (2C), 21.7.

IR (neat, cm^{-1}) 1438, 1385, 1029, 766, 756, 702, 657.

HRMS (APCI) exact mass calcd. for $C_{27}H_{21}$ $[M + H]^+$: 345.1638, found 345.1627.

3. X-Ray Crystallographic Data

Crystal structure of compound 2b-D_{10}: CCDC 2393581

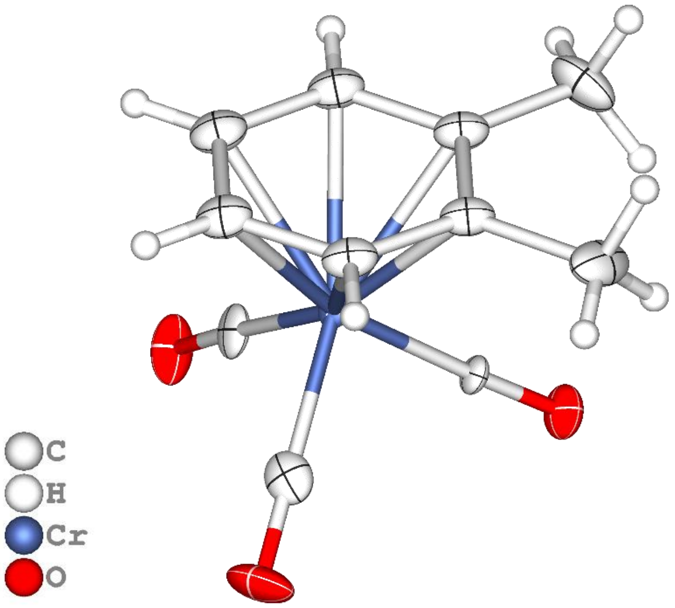

Single crystals were obtained using the slow-evaporation method with n-hexane as solvent at room temperature.

Table S1: Crystal data and structure refinement for **2b-D_{10}**.

Identification code	GBUN039, CM762 // GXray7572
Crystal Habitus	clear yellow block
Device Type	STOE STADIVARI
Empirical formula	$C_{11}H_{10}CrO_3$
Moiety formula	C11 H10 Cr O3
Formula weight	242.19
Temperature/K	100
Crystal system	orthorhombic
Space group	$P2_12_12_1$

a/Å	7.22294(16)
b/Å	11.6770(3)
c/Å	12.5046(4)
α/°	90.00
β/°	90.00
γ/°	90.00
Volume/Å^3	1054.67(4)
Z	4
ρ_{calc}g/cm^3	1.525
μ/mm^{-1}	8.81
F(000)	496.0
Crystal size/mm^3	0.35 × 0.35 × 0.3
Absorption correction	multi-scan
Tmin; Tmax	0.0044; 0.0175
Radiation	CuKα (λ = 1.54186)
2Θ range for data collection/°	10.366 to 140.798°
Completeness to theta	0.999
Index ranges	-7 ≤ h ≤ 8, -14 ≤ k ≤ 8, -15 ≤ l ≤ 15
Reflections collected	9461
Independent reflections	2014 [R_{int} = 0.0468, R_{sigma} = 0.0245]
Data/restraints/parameters	2014/36/139
Goodness-of-fit on F^2	1.033

Final R indexes [I>=2σ (I)]	R_1 = 0.0615, wR_2 = 0.1590
Final R indexes [all data]	R_1 = 0.0620, wR_2 = 0.1599
Largest diff. peak/hole / e $Å^{-3}$	1.09/-1.36
Flack parameter	0.011(13)

Crystal structure of compound 2e: CCDC 2392930

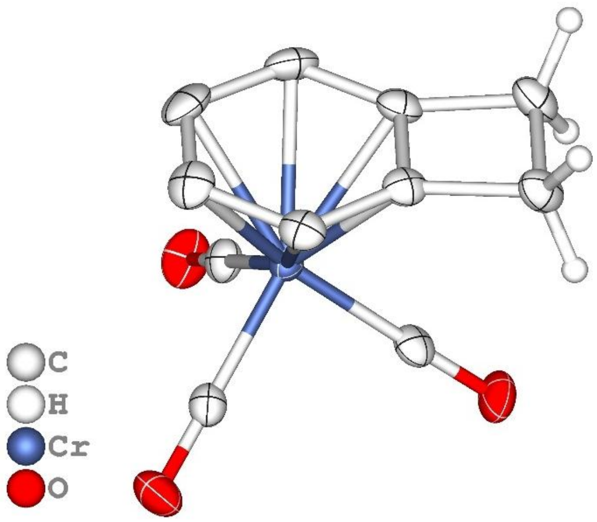

Single crystals were obtained using the slow-evaporation method with n-hexane as solvent at room temperature. (Aromatic hydrogen atoms are omitted for clarity)

Table S2: Crystal data and structure refinement for **2e**.

Identification code	GBUN052, CM857 // GXray7840b	
Crystal Habitus	clear yellow block	
Device Type	STOE STADIVARI	
Empirical formula	$C_{11}H_8CrO_3$	
Moiety formula	C11 H8 Cr O3	
Formula weight	240.17	
Temperature/K	100	
Crystal system	Orthorhombic	
Space group	$P2_12_12_1$	
	A = 6.88861(17) Å	α = 90.00°
Cell dimensions	b = 11.2594(4) Å	β = 90.00°
	c = 12.6061(3) Å	γ = 90.00°

Volume/Å^3	977.75(5)
Z	4
ρ_{calc} g/cm^3	1.632
μ/mm^{-1}	9.51
F(000)	488.0
Crystal size/mm^3	0.25 × 0.1 × 0.06
Absorption correction	multi-scan
T_{min}; T_{max}	0.0593; 0.1489
Radiation	CuKα (λ = 1.54186)
2Θ range for data collection/°	10.534 to 140.496°
Completeness to theta	0.997
Index ranges	-8 ≤ h ≤ 8, -13 ≤ k ≤ 13, -7 ≤ l ≤ 14
Reflections collected	15424
Independent reflections	1865 [R_{int} = 0.0273, R_{sigma} = 0.0126]
Data/restraints/ parameters	1865/0/137
Goodness-of-fit on F^2	1.037
Final R indexes [I>=2σ (I)]	R_1 = 0.0253, wR_2 = 0.0649
Final R indexes [all data]	R_1 = 0.0256, wR_2 = 0.0652
Largest diff. Peak/ hole / e Å^{-3}	0.25/-0.60
Flack parameter	0.034(7)

Crystal structure of compound 2g: CCDC 2392931

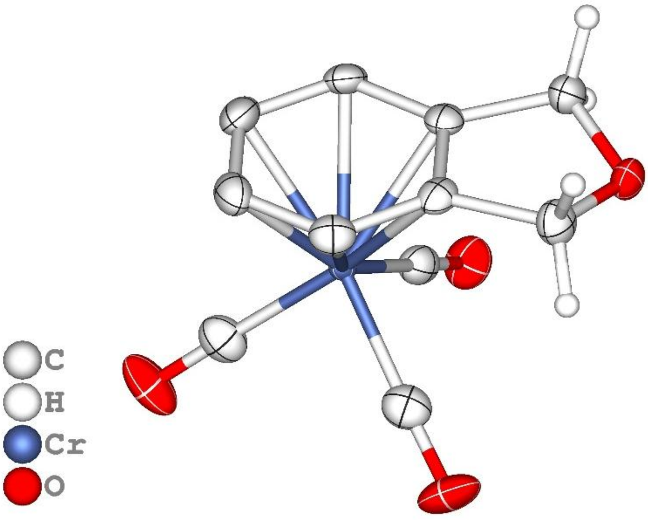

Single crystals were obtained using the slow-evaporation method with n-hexane as solvent at room temperature. (Aromatic hydrogen atoms are omitted for clarity)

Table S3: Crystal data and structure refinement for **2g**.

Identification code	GBUN050,CM847-DC14 // GXray7838	
Crystal Habitus	clear yellow plank	
Device Type	STOE STADIVARI	
Empirical formula	$C_{11}H_8CrO_4$	
Moiety formula	C11 H8 Cr O4	
Formula weight	256.17	
Temperature/K	100	
Crystal system	monoclinic	
Space group	$P2_1/c$	
	A = 13.0012(4) Å	α = 90°
Cell dimensions	b = 8.0631(2) Å	β = 111.0212(25)°
	c = 10.6144(4) Å	γ = 90°

Volume/$Å^3$	1038.66(6)
Z	4
ρ_{calc} g/cm^3	1.638
μ/mm^{-1}	9.07
F(000)	520.0
Crystal size/mm^3	0.21 × 0.09 × 0.04
Absorption correction	multi-scan
T_{min}; T_{max}	0.1112; 0.1906
Radiation	CuKα (λ = 1.54186)
2Θ range for data collection/°	13.184 to 140.604°
Completeness to theta	0.997
Index ranges	-10 ≤ h ≤ 15, -9 ≤ k ≤ 9, -12 ≤ l ≤ 12
Reflections collected	13353
Independent reflections	1975 [R_{int} = 0.0253, R_{sigma} = 0.0150]
Data/restraints/ parameters	1975/0/145
Goodness-of-fit on F^2	1.063
Final R indexes [I>=2σ (I)]	R_1 = 0.0286, wR_2 = 0.0724
Final R indexes [all data]	R_1 = 0.0316, wR_2 = 0.0743
Largest diff. Peak/ hole / e $Å^{-3}$	0.34/-0.70

Crystal structure of compound 2j: CCDC 2392932

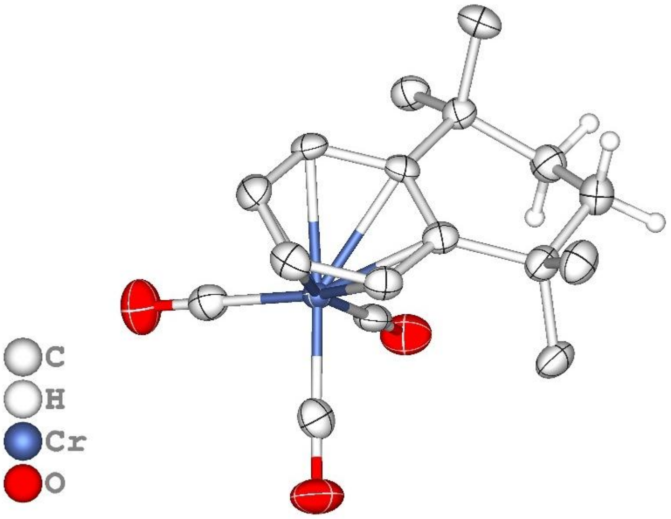

Single crystals were obtained using the slow-evaporation method with n-hexane as solvent at room temperature. (Aromatic and methyl hydrogen atoms are omitted for clarity)

Table S4: Crystal data and structure refinement for **2j**.

Identification code	GBUN051, CM848-DC15 // GXray783	
Crystal Habitus	clear light yellow plate	
Device Type	STOE STADIVARI	
Empirical formula	$C_{17}H_{20}CrO_3$	
Moiety formula	C17 H20 Cr O3	
Formula weight	324.33	
Temperature/K	100	
Crystal system	Orthorhombic	
Space group	Pbca	
	A =15.5066(5) Å	α = 90°
Cell dimensions	b = 11.4110(4) Å	β = 90°
	c = 17.1684(8) Å	γ = 90°

Volume/Å^3	3037.9(2)
Z	8
ρ_{calc} g/cm^3	1.418
μ/mm^{-1}	6.26
F(000)	1360.0
Crystal size/mm^3	0.22 × 0.16 × 0.01
Absorption correction	multi-scan
T_{min}; T_{max}	0.0320; 0.0581
Radiation	CuKα (λ = 1.54186)
2Θ range for data collection/°	10.306 to 140.964°
Completeness to theta	1.000
Index ranges	-16 ≤ h ≤ 18, -13 ≤ k ≤ 8, -20 ≤ l ≤ 20
Reflections collected	38800
Independent reflections	2898 [R_{int} = 0.1106, R_{sigma} = 0.0433]
Data/restraints/ parameters	2898/0/194
Goodness-of-fit on F^2	1.021
Final R indexes [I>=2σ (I)]	R_1 = 0.0426, wR_2 = 0.0921
Final R indexes [all data]	R_1 = 0.0755, wR_2 = 0.1077
Largest diff. Peak/ hole / e Å^{-3}	0.31/-0.40

Crystal structure of compound 2q: CCDC 2392933

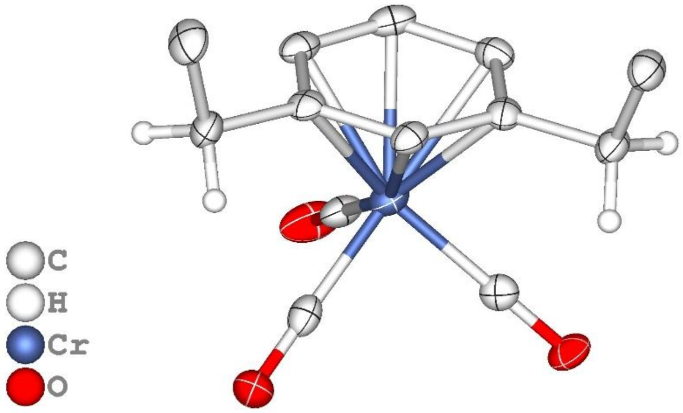

Single crystals were obtained using the slow-evaporation method with n-hexane as solvent at room temperature. (Aromatic and methyl hydrogen atoms are omitted for clarity)

Table S5: Crystal data and structure refinement for **2q**.

Identification code	GBUN048, CM790-DC03 // GXray7806	
Crystal Habitus	clear light green plate	
Device Type	STOE Stadivari	
Empirical formula	$C_{13}H_{14}CrO_3$	
Moiety formula	C13 H14 Cr O3	
Formula weight	270.24	
Temperature/K	100	
Crystal system	monoclinic	
Space group	$P2_1/c$	
	a = 11.4817(5) Å	α = 90°
Cell dimensions	b = 15.5213(5) Å	β = 96.147(4) °
	c = 13.9828(7) Å	γ = 90°
Volume/Å^3	2477.56(18)	
Z	8	

ρ_{calc} g/cm^3	1.449
μ/mm^{-1}	7.553
F(000)	1120.0
Crystal size/mm^3	0.2 × 0.117 × 0.02
Absorption correction	multi-scan
T_{min}; T_{max}	0.1555; 0.2698
Radiation	CuKα (λ = 1.54186)
2Θ range for data collection/°	7.744 to 140.942°
Completeness to theta	1.000
Index ranges	$-12 \le h \le 14$, $-18 \le k \le 9$, $-17 \le l \le 16$
Reflections collected	33511
Independent reflections	4718 [R_{int} = 0.1241, R_{sigma} = 0.0722
Data/restraints/ parameters	4718/0/311
Goodness-of-fit on F^2	0.996
Final R indexes [I>=2σ (I)]	R_1 = 0.0609, wR_2 = 0.1432
Final R indexes [all data]	R_1 = 0.1010, wR_2 = 0.1687
Largest diff. Peak/ hole / e Å^{-3}	0.89/-0.45

Crystal structure of compound 2s: CCDC 2392934

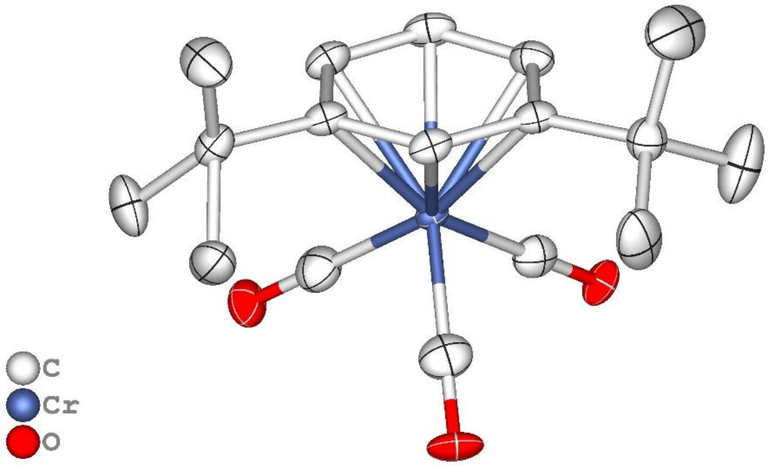

Single crystals were obtained using the slow-evaporation method with n-hexane/DCM as solvent at room temperature. (Hydrogen atoms are omitted for clarity)

Table S6: Crystal data and structure refinement for **2s**.

Identification code	GBUN061, AM-589 // GXray7740	
Crystal Habitus	clear yellow plate	
Device Type	STOE STADIVARI	
Empirical formula	$C_{17}H_{22}CrO_3$	
Moiety formula	C17 H22 Cr O3	
Formula weight	326.35	
Temperature/K	100	
Crystal system	orthorhombic	
Space group	Pbca	
	a = 11.9485(4) Å	α = 90°
Cell dimensions	b = 16.1977(7) Å	β = 90°
	c = 16.8644(5) Å	γ = 90°
Volume/Å^3	3263.89(20)	

Z	8
ρ_{calc} g/cm^3	1.328
μ/mm^{-1}	5.83
F(000)	1376.0
Crystal size/mm^3	0.23 × 0.22 × 0.12
Absorption correction	multi-scan
T_{min}; T_{max}	0.1161; 0.1692
Radiation	CuKα (λ = 1.54186)
2Θ range for data collection/°	10.492 to 141.038°
Completeness to theta	0.999
Index ranges	-12 ≤ h ≤ 14, -19 ≤ k ≤ 19, -20 ≤ l ≤ 8
Reflections collected	31025
Independent reflections	3109 [R_{int} = 0.0408, R_{sigma} = 0.0184]
Data/restraints/ parameters	3109/0/227
Goodness-of-fit on F^2	1.050
Final R indexes [I>=2σ (I)]	R_1 = 0.0318, wR_2 = 0.0792
Final R indexes [all data]	R_1 = 0.0400, wR_2 = 0.0835
Largest diff. Peak/ hole / e Å^{-3}	0.21/-0.62

Crystal structure of compound 2t: CCDC 2392935

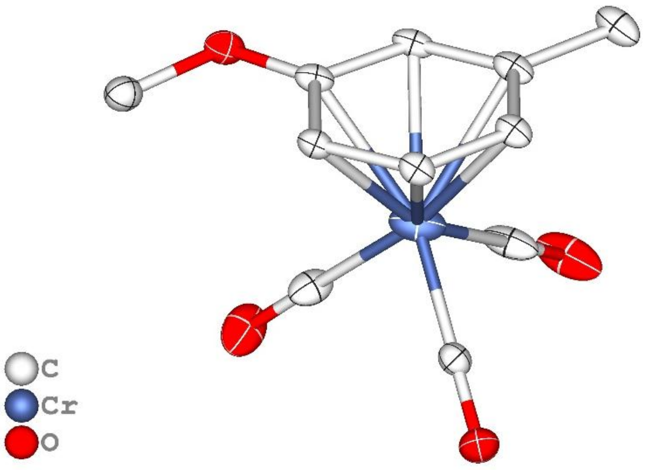

Single crystals were obtained using the slow-evaporation method with n-hexane as solvent at room temperature. (Hydrogen atoms are omitted for clarity)

Table S7: Crystal data and structure refinement for **2t**.

Identification code	GBUN029, CM701 // GXraymo_7433f	
Crystal Habitus	clear yellow plate	
Device Type	Bruker D8 Venture	
Empirical formula	$C_{11}H_{10}O_4Cr$	
Moiety formula	C11 H10 Cr O4	
Formula weight	258.19	
Temperature/K	100	
Crystal system	monoclinic	
Space group	$P2_1/n$	
	a = 6.1409(7) Å	α = 90°
Cell dimensions	b = 12.3802(14) Å	β = 10.456(4)°
	c =14.3384(13) Å	γ = 90°
Volume/Å^3	1072.0(2)	

Z	4
ρ_{calc} g/cm^3	1.600
μ/mm^{-1}	1.061
F(000)	528.0
Crystal size/mm^3	0.3 × 0.16 × 0.02
Absorption correction	multi-scan
T_{min}; T_{max}	0.6338; 0.7464
Radiation	MoKα (λ = 0.71073)
2Θ range for data collection/°	4.378 to 65.082°
Completeness to theta	1.000
Index ranges	-9 ≤ h ≤ 9, -18 ≤ k ≤ 18, -21 ≤ l ≤ 18
Reflections collected	29792
Independent reflections	3893 [R_{int} = 0.0718, R_{sigma} = 0.0418]
Data/restraints/ parameters	3893/294/222
Goodness-of-fit on F^2	1.057
Final R indexes [I>=2σ (I)]	R_1 = 0.0455, wR_2 = 0.1087
Final R indexes [all data]	R_1 = 0.0607, wR_2 = 0.1210
Largest diff. Peak/ hole / e Å^{-3}	0.70/-1.09

Crystal structure of compound 2v: CCDC 2392936

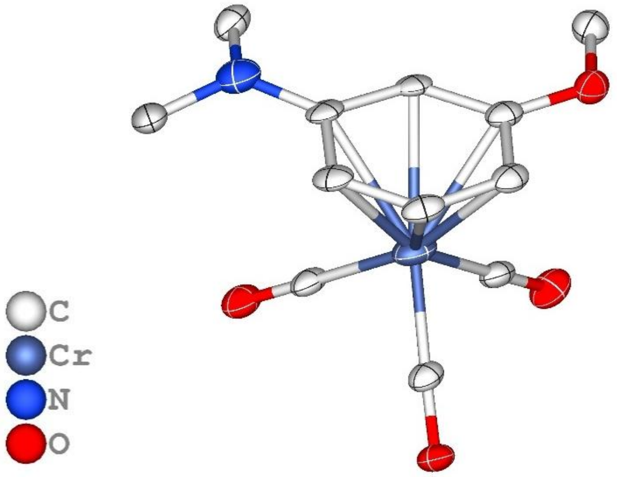

Single crystals were obtained using the slow-evaporation method with n-hexane as solvent at room temperature. (Hydrogen atoms are omitted for clarity)

Table S8: Crystal data and structure refinement for **2v**.

Identification code	GBUN057, CM-814-DC-II // GXray7675	
Crystal Habitus	clear yellow plate	
Device Type	STOE STADIVARI	
Empirical formula	$C_{12}H_{13}CrNO_4$	
Moiety formula	C12 H13 Cr N O4	
Formula weight	287.23	
Temperature/K	100	
Crystal system	triclinic	
Space group	P-1	
	a = 9.1437(5) Å	α = 98.794(4)°
Cell dimensions	b = 10.9624(6) Å	β = 92.299(4)°
	c = 12.4517(7) Å	γ = 99.399(4)°
Volume/Å^3	1214.17(12)	

Z	4
ρ_{calc} g/cm^3	1.571
μ/mm^{-1}	7.833
F(000)	592.0
Crystal size/mm^3	0.7 × 0.473 × 0.12
Absorption correction	multi-scan
T_{min}; T_{max}	0.0244; 0.0631
Radiation	CuKα (λ = 1.54186)
2Θ range for data collection/°	7.2 to 141.528°
Completeness to theta	0.990
Index ranges	-11 ≤ h ≤ 10, -13 ≤ k ≤ 12, -15 ≤ l ≤ 8
Reflections collected	16550
Independent reflections	4543 [R_{int} = 0.0689, R_{sigma} = 0.0446]
Data/restraints/ parameters	4543/0/331
Goodness-of-fit on F^2	1.056
Final R indexes [I>=2σ (I)]	R_1 = 0.0859, wR_2 = 0.2333
Final R indexes [all data]	R_1 = 0.0916, wR_2 = 0.2439
Largest diff. Peak/ hole / e Å^{-3}	1.18/-1.69

Crystal structure of compound 2w: CCDC 2392937

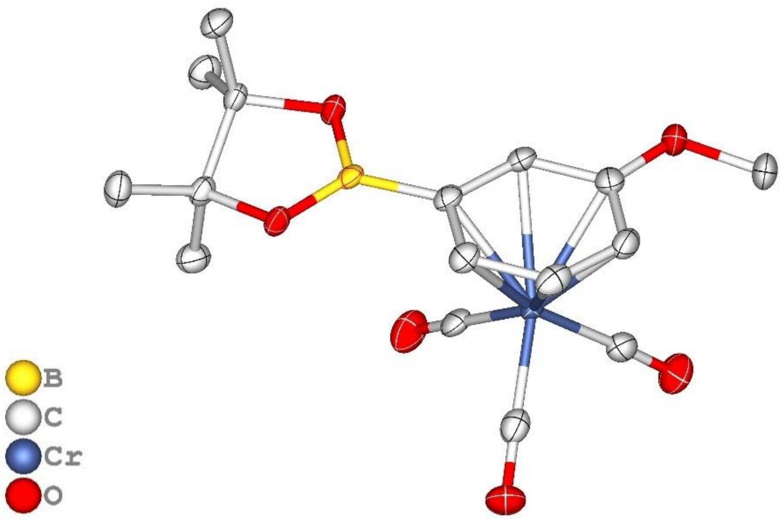

Single crystals were obtained using the slow-evaporation method with toluene as solvent at room temperature. (Hydrogen atoms are omitted for clarity)

Table S9: Crystal data and structure refinement for **2w**.

Identification code	GBUN038, CM742 // GXray7530	
Crystal Habitus	clear light yellow block	
Device Type	STOE STADIVARI	
Empirical formula	$C_{16}H_{19}BCrO_6$	
Moiety formula	C16 H19 B Cr O6	
Formula weight	370.12 g/mol	
Temperature/K	100	
Crystal system	triclinic	
Space group	P-1	
	a = 6.2935(2) Å	α = 81.876(3)°
Cell dimensions	b = 9.5281(3) Å	β = 86.520(3)°
	c = 14.1367(5) Å	γ = 86.209(3)°
Volume/$Å^3$	836.23(5)	

Z	2
ρ_{calc} g/cm^3	1.470
μ/mm^{-1}	5.90
F(000)	384.0
Crystal size/mm^3	0.2 × 0.18 × 0.1
Absorption correction	multi-scan
T_{min}; T_{max}	0.1603; 0.2343
Radiation	CuKα (λ = 1.54186)
2Θ range for data collection/°	9.392 to 140.82°
Completeness to theta	0.991
Index ranges	-7 ≤ h ≤ 7, -11 ≤ k ≤ 11, -7 ≤ l ≤ 17
Reflections collected	11268
Independent reflections	3129 [R_{int} = 0.0207, R_{sigma} = 0.0167]
Data/restraints/ parameters	3129/0/222
Goodness-of-fit on F^2	1.038
Final R indexes [I>=2σ (I)]	R_1 = 0.0334, wR_2 = 0.0870
Final R indexes [all data]	R_1 = 0.0350, wR_2 = 0.0883
Largest diff. Peak/ hole / e Å^{-3}	0.35/-0.64

Crystal structure of compound 2y: CCDC 2392938

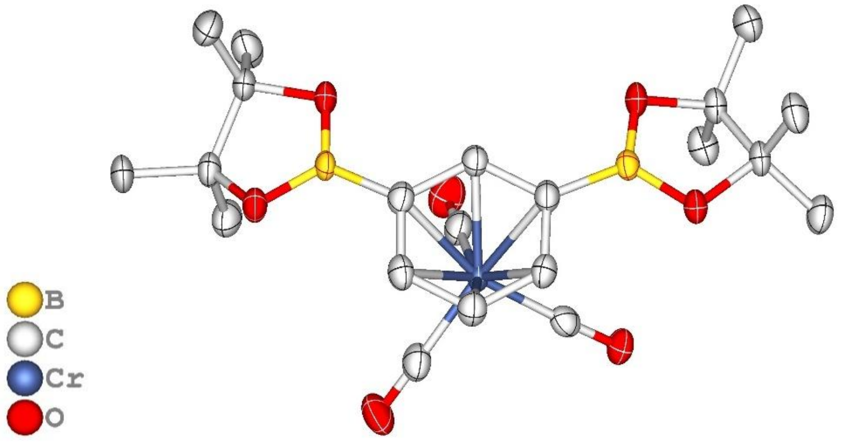

Single crystals were obtained using the slow-evaporation method with benzene as solvent at room temperature. (Hydrogen atoms are omitted for clarity)

Table S10: Crystal data and structure refinement for **2y**.

Identification code	GBUN062, CM823 // GXray7804	
Crystal Habitus	clear yellow block	
Device Type	STOE Stadivari	
Empirical formula	$C_{48}H_{62}B_4Cr_2O_{14}$	
Moiety formula	2(C21 H28 B2 Cr O7), C6 H6	
Formula weight	1010.22	
Temperature/K	100	
Crystal system	triclinic	
Space group	P-1	
	a = 11.4479(3) Å	α = 104.2759(23)°
Cell dimensions	b = 14.7511(4) Å	β = 97.3028(24)°
	c = 17.1838(5) Å	γ = 111.7065(22)°
Volume/Å^3	2534.82(13)	
Z	2	

ρ_{calc} g/cm^3	1.324
μ/mm^{-1}	4.02
F(000)	1060.0
Crystal size/mm^3	0.33 × 0.21 × 0.16
Absorption correction	multi-scan
T_{min}; T_{max}	0.1122; 0.2384
Radiation	CuKα (λ = 1.54186)
2Θ range for data collection/°	6.804 to 141.042°
Completeness to theta	0.994
Index ranges	-13 ≤ h ≤ 9, -17 ≤ k ≤ 17, -17 ≤ l ≤ 20
Reflections collected	59662
Independent reflections	9536 [R_{int} = 0.0625, R_{sigma} = 0.0369]
Data/restraints/ parameters	9536/0/629
Goodness-of-fit on F^2	1.030
Final R indexes [I>=2σ (I)]	R_1 = 0.0568, wR_2 = 0.1501
Final R indexes [all data]	R_1 = 0.0669, wR_2 = 0.1612
Largest diff. Peak/ hole / e Å^{-3}	0.78/-0.74

Crystal structure of compound 2ae: CCDC 2392939

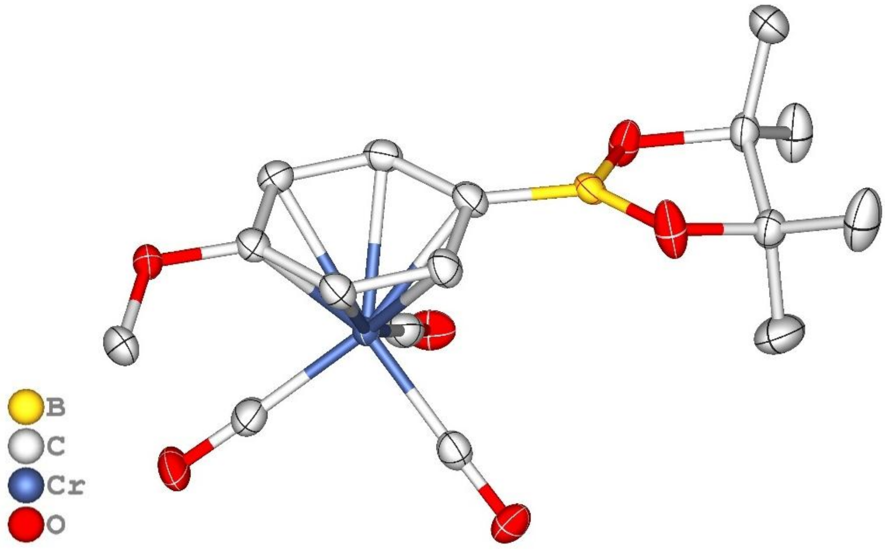

Single crystals were obtained using the slow-evaporation method with EtOAc as solvent at room temperature. (Hydrogen atoms are omitted for clarity)

Table S11: Crystal data and structure refinement for **2ae**.

Identification code	GBUN010, CM208 // GXraymo_7051f	
Crystal Habitus	clear light yellow plate	
Device Type	Bruker D8 Venture	
Empirical formula	$C_{16}H_{19}BCrO_6$	
Moiety formula	C16 H19 B Cr O6	
Formula weight	370.12 g/mol	
Temperature/K	100	
Crystal system	orthorhombic	
Space group	Pbca	
	a =12.5109(3) Å	α = 90°
Cell dimensions	b = 12.5031(2) Å	β = 90°
	c = 21.7107(5) Å	γ = 90°

Volume/Å^3	3396.10(13)
Z	8
ρ_{calc} g/cm^3	1.448
μ/mm^{-1}	0.701
F(000)	1536.0
Crystal size/mm^3	0.35 × 0.1 × 0.04
Absorption correction	mulit-scan
T_{min}; T_{max}	0.6393; 0.7461
Radiation	MoK_α (λ = 0.71073)
2Θ range for data collection/°	3.752 to 55.998°
Completeness to theta	0.999
Index ranges	-16 ≤ h ≤ 16, -16 ≤ k ≤ 14, -28 ≤ l ≤ 28
Reflections collected	34503
Independent reflections	4097 [R_{int} = 0.0358, R_{sigma} = 0.0197]
Data/restraints/ parameters	4097/330/261
Goodness-of-fit on F^2	1.049
Final R indexes [I>=2σ (I)]	R_1 = 0.0317, wR_2 = 0.0834
Final R indexes [all data]	R_1 = 0.0372, wR_2 = 0.0889
Largest diff. Peak/ hole / e Å^{-3}	1.15/-0.36

Crystal structure of compound 2af: CCDC 2392940

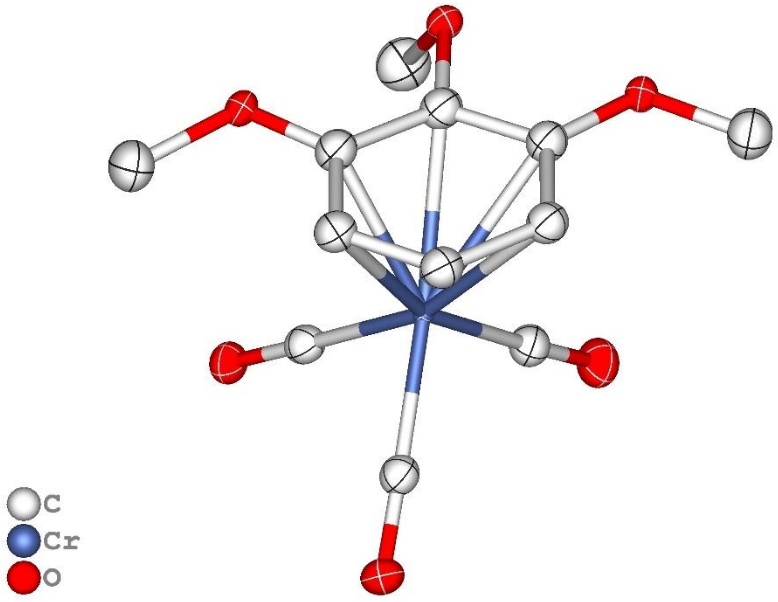

Single crystals were obtained using the slow-evaporation method with toluene as solvent at room temperature. (Hydrogen atoms are omitted for clarity)

Table S12: Crystal data and structure refinement for **2af**.

Identification code	GBUN070, CM789 // 7642	
Crystal Habitus	clear yellow block	
Device Type	STOE STRADIVARI	
Empirical formula	$C_{12}H_{12}CrO_6$	
Moiety formula	C12 H12 Cr O6	
Formula weight	304.22	
Temperature/K	100	
Crystal system	Monoclinic	
Space group	$P2_1/c$	
Cell dimensions	a = 6.8685(2) Å	α = 90°
	b = 24.3966(6) Å	β = 103.896(2)°

	c = 7.8760(2) Å γ = 90°
Volume/Å^3	1281.14(6)
Z	4
ρ_{calc}g/cm^3	1.577
μ/mm^{-1}	7.559
F(000)	624.0
Crystal size/mm^3	0.27 × 0.25 × 0.12
Absorption correction	multi-scan
T_{min}; T_{max}	0.0882; 0.0412
Radiation	Cu Kα (λ = 1.54186)
2Θ range for data collection/°	7.246 to 177.248
Index ranges	-8 ≤ h ≤ 8, -11 ≤ k ≤ 28, -7 ≤ l ≤ 8
Reflections collected	12072
Independent reflections	2362 [R_{int} = 0.0262, R_{sigma} = 0.0161]
Data/restraints/parameters	2362/402/187
Goodness-of-fit on F^2	1.033
Final R indexes [I>=2σ (I)]	R_1 = 0.0859, wR_2 = 0.2226
Final R indexes [all data]	R_1 = 0.0872, wR_2 = 0.2253
Largest diff. peak/hole / e Å^{-3}	2.43/-3.18

Crystal structure of compound 2ag: CCDC 2392941

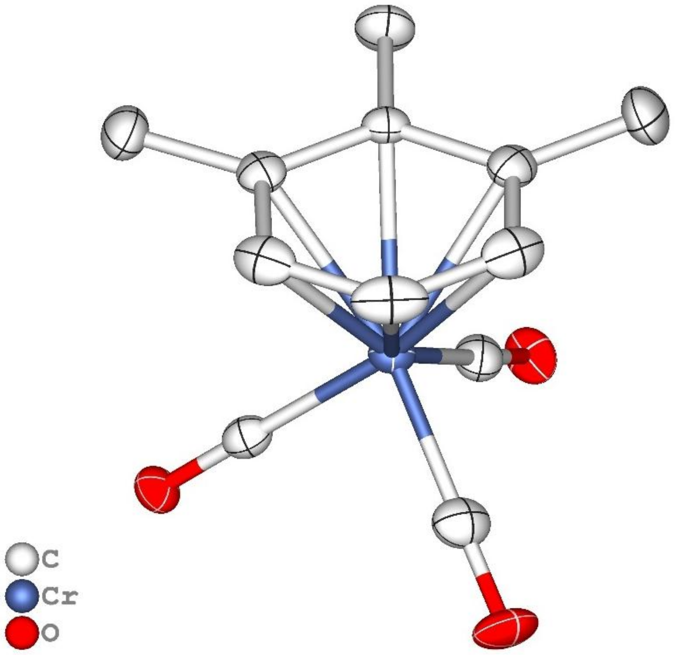

Single crystals were obtained using the slow-evaporation method with n-hexane/DCM as solvent at room temperature. (Hydrogen atoms are omitted for clarity)

Table S13: Crystal data and structure refinement for **2ag**.

Identification code	AM-582 // GXraymo_7708f	
Crystal Habitus	yellow block	
Device Type	Bruker D8 Venture	
Empirical formula	$C_{12}H_{12}CrO_3$	
Moiety formula	C12 H12 Cr O3	
Formula weight	256.22	
Temperature/K	100.00	
Crystal system	monoclinic	
Space group	$P2_1/n$	
Cell dimensions	a = 7.2191(4) Å	α = 90°

	b = 13.0306(7) Å	β = 99.727(2)°
	c = 12.3340(7) Å	γ = °
Volume/$Å^3$	1143.57(11)	
Z	4	
ρ_{calc} g/cm^3	1.488	
μ/mm^{-1}	0.987	
F(000)	528.0	
Crystal size/mm^3	0.4 × 0.16 × 0.06	
Absorption correction	multi-scan	
T_{min}; T_{max}	0.6003; 0.7456	
Radiation	MoKα (λ = 0.71073)	
2Θ range for data collection/°	4.582 to 54.99°	
Completeness to theta	0.999	
Index ranges	-9 ≤ h ≤ 9, -16 ≤ k ≤ 16, -15 ≤ l ≤ 16	
Reflections collected	45971	
Independent reflections	2624 [R_{int} = 0.0935, R_{sigma} = 0.0395]	
Data/restraints/ parameters	2624/0/148	
Goodness-of-fit on F^2	1.210	
Final R indexes [I>=2σ (I)]	R_1 = 0.0434, wR_2 = 0.1041	
Final R indexes [all data]	R_1 = 0.0451, wR_2 = 0.1054	
Largest diff. Peak/ hole / e $Å^{-3}$	0.86/-0.42	

Crystal structure of compound 2ah: CCDC 2392942

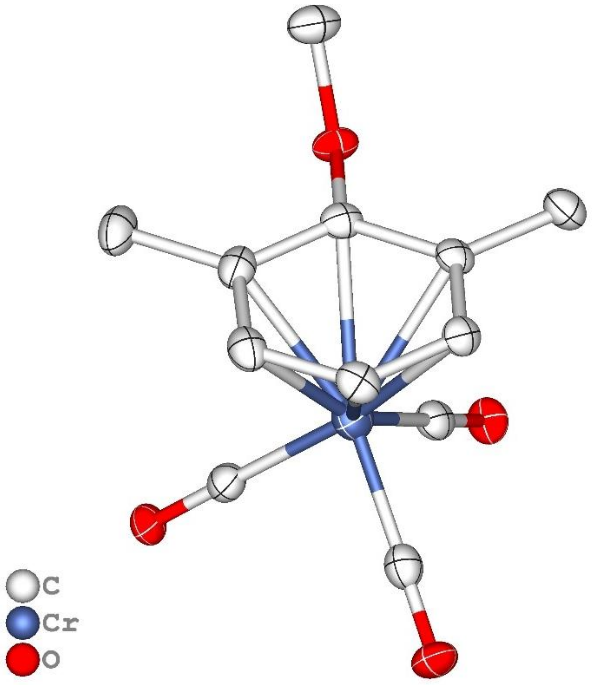

Single crystals were obtained using the slow-evaporation method with n-hexane/DCM as solvent at room temperature. (Hydrogen atoms are omitted for clarity)

Table S14: Crystal data and structure refinement for **2ah**.

Identification code	GBUN065, CM858 // GXray7841
Crystal Habitus	clear yellow plate
Device Type	STOE STADIVARI
Empirical formula	$C_{12}H_{12}CrO_4$
Moiety formula	C12 H12 Cr O4
Formula weight	272.22
Temperature/K	100
Crystal system	triclinic
Space group	P-1

a/Å	6.93154(22)
b/Å	7.60520(24)
c/Å	12.7659(4)
α/°	79.2305(25)
β/°	76.0776(25)
γ/°	66.0411(24)
Volume/Å^3	593.89(3)
Z	2
ρ_{calc}g/cm^3	1.522
μ/mm^{-1}	7.96
F(000)	280.0
Crystal size/mm^3	0.22 × 0.16 × 0.05
Absorption correction	multi-scan
Tmin; Tmax	0.1206; 0.1884
Radiation	CuKα (λ = 1.54186)
2Θ range for data collection/°	7.17 to 141.056°
Completeness to theta	0.994
Index ranges	-8 ≤ h ≤ 6, -8 ≤ k ≤ 9, -15 ≤ l ≤ 15
Reflections collected	18332
Independent reflections	2257 [R_{int} = 0.0391, R_{sigma} = 0.0177]
Data/restraints/parameters	2257/0/157
Goodness-of-fit on F^2	1.064

Final R indexes [I>=2σ (I)]	R_1 = 0.0347, wR_2 = 0.0945
Final R indexes [all data]	R_1 = 0.0372, wR_2 = 0.0964
Largest diff. peak/hole / e Å^{-3}	0.40/-0.58

Crystal structure of compound 2ak: CCDC 2392943

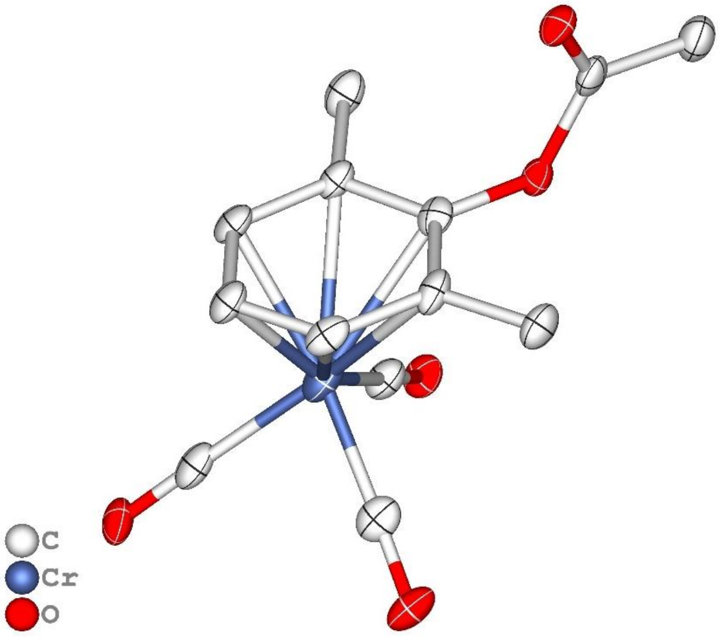

*Single crystals were obtained using the slow-evaporation method with n-*hexane/DCM *as solvent at room temperature. (Hydrogen atoms are omitted for clarity)*

Table S15: Crystal data and structure refinement for **2ak**.

Identification code	GBUN079, AM-756 // GXray8007	
Crystal Habitus	clear light yellow plank	
Device Type	STOE STADIVARI	
Empirical formula	$C_{13}H_{12}CrO_5$	
Moiety formula	C13 H12 Cr O5	
Formula weight	300.23	
Temperature/K	100	
Crystal system	orthorhombic	
Space group	$Pca2_1$	
Cell dimensions	a = 10.5328(5) Å	α = 90°
	b = 11.0249(4) Å	β = 90°

	c = 11.5452(4) Å γ = 90°
Volume/Å^3	1340.66(9)
Z	4
ρ_{calc} g/cm^3	1.487
μ/mm^{-1}	7.160
F(000)	616.0
Crystal size/mm^3	0.5 × 0.15 × 0.05
Absorption correction	multi-scan
T_{min}; T_{max}	0.0402; 0.0773
Radiation	Cu Kα (λ = 1.54186)
2Θ range for data collection/°	8.02 to 173.004°
Completeness to theta	0.974
Index ranges	-13 ≤ h ≤ 13, -12 ≤ k ≤ 12, -6 ≤ l ≤ 13
Reflections collected	28805
Independent reflections	1901 [R_{int} = 0.1287, R_{sigma} = 0.0328]
Data/restraints/ parameters	1901/1/176
Goodness-of-fit on F^2	1.168
Final R indexes [I>=2σ (I)]	R_1 = 0.0485, wR_2 = 0.1222
Final R indexes [all data]	R_1 = 0.0645, wR_2 = 0.1595
Largest diff. Peak/ hole / e Å^{-3}	0.94/-0.76
Flack parameter	0.002(17)

Crystal structure of compound 2an: CCDC 2392944

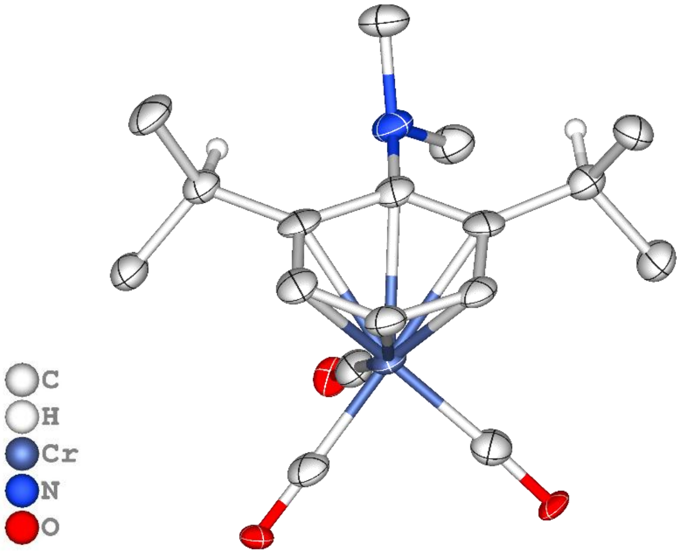

Single crystals were obtained using the slow-evaporation method with n-hexane as solvent at room temperature. (Aromatic and methyl hydrogen atoms are omitted for clarity)

Table S16: Crystal data and structure refinement for **2an**.

Identification code	GBUN066, CM861-DC18 // GXray7843	
Crystal Habitus	clear yellow plate	
Device Type	STOE STADIVARI	
Empirical formula	$C_{17}H_{23}CrNO_3$	
Moiety formula	C17 H23 Cr N O3	
Formula weight	341.37	
Temperature/K	100	
Crystal system	triclinic	
Space group	P-1	
	a = 8.5932(4) Å	α = 96.588(4)°
Cell dimensions	b = 9.5905(5) Å	β = 100.785(4)°
	c = 11.4427(6) Å	γ = 113.289(3)°

Volume/Å^3	832.07(7)
Z	2
ρ_{calc} g/cm^3	1.362
μ/mm^{-1}	5.76
F(000)	360.0
Crystal size/mm^3	0.16 × 0.13 × 0.08
Absorption correction	multi-scan
T_{min}; T_{max}	0.2137; 0.3630
Radiation	CuKα (λ = 1.54186)
2Θ range for data collection/°	8.044 to 141.012°
Completeness to theta	0.993
Index ranges	-10 ≤ h ≤ 10, -11 ≤ k ≤ 10, -13 ≤ l ≤ 10
Reflections collected	19048
Independent reflections	3134 [R_{int} = 0.0605, R_{sigma} = 0.0357]
Data/restraints/ parameters	3134/0/205
Goodness-of-fit on F^2	1.062
Final R indexes [I>=2σ (I)]	R_1 = 0.0622, wR_2 = 0.1525
Final R indexes [all data]	R_1 = 0.0664, wR_2 = 0.1594
Largest diff. Peak/ hole / e Å^{-3}	1.34/-0.72

Crystal structure of compound 4b: CCDC 2392945

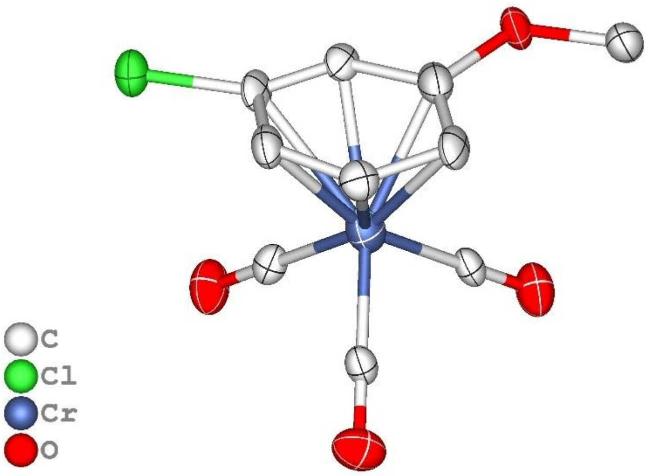

Single crystals were obtained using the slow-evaporation method with toluene as solvent at room temperature. (Hydrogen atoms are omitted for clarity)

Table S17: Crystal data and structure refinement for **4b**.

Identification code	GBUN054, CM704 // GXray7531	
Crystal Habitus	clear yellow plank	
Device Type	STOE STADIVARI	
Empirical formula	$C_{10}H_7ClCrO_4$	
Moiety formula	C10 H7 Cl Cr O4	
Formula weight	278.61	
Temperature/K	100	
Crystal system	triclinic	
Space group	P-1	
	a = 7.1012(4) Å	α = 72.577(5)°
Cell dimensions	b = 9.3161(6) Å	β = 67.142(5)°
	c = 9.3817(6) Å	γ = 68.118(5)°
Volume/Å^3	521.96(6)	

Z	2
ρ_{calc} g/cm^3	1.773
μ/mm^{-1}	11.38
F(000)	280.0
Crystal size/mm^3	0.22 × 0.17 × 0.05
Absorption correction	multi-scan
T_{min}; T_{max}	0.0277; 0.1244
Radiation	CuKα (λ = 1.54186)
2Θ range for data collection/°	10.404 to 140.392°
Completeness to theta	0.988
Index ranges	-7 ≤ h ≤ 8, -11 ≤ k ≤ 9, -10 ≤ l ≤ 11
Reflections collected	6001
Independent reflections	1957 [R_{int} = 0.0965, R_{sigma} = 0.0770]
Data/restraints/ parameters	1957/148/202
Goodness-of-fit on F^2	1.325
Final R indexes [I>=2σ (I)]	R_1 = 0.1128, wR_2 = 0.2906
Final R indexes [all data]	R_1 = 0.1283, wR_2 = 0.3136
Largest diff. Peak/ hole / e Å^{-3}	1.33/-0.90

Crystal structure of compound 4c: CCDC 2409305

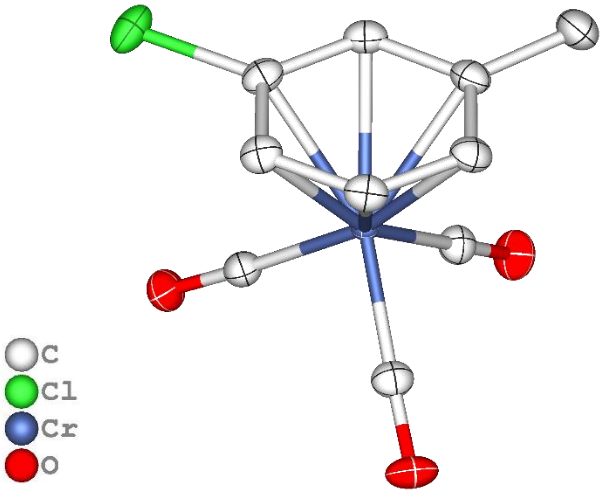

Single crystals were obtained using the slow-evaporation method with toluene as solvent at room temperature. (Hydrogen atoms are omitted for clarity)

Table S18: Crystal data and structure refinement for **4c**.

Identification code	GBUN121, FH-36 // GXraymo_8590f
Crystal Habitus	clear yellow block
Device Type	Bruker D8 Venture
Empirical formula	$C_{10}H_7O_3ClCr$
Moiety formula	C10 H7 Cl Cr O3
Formula weight	262.61
Temperature/K	104.00
Crystal system	monoclinic
Space group	$P2_1/n$
a/Å	7.4431(3)
b/Å	11.1413(4)

c/Å	12.8720(5)
α/°	90
β/°	106.1680(10)
γ/°	90
Volume/Å^3	1025.20(7)
Z	4
ρ_{calc}g/cm^3	1.701
μ/mm^{-1}	1.356
F(000)	528.0
Crystal size/mm^3	0.35 × 0.14 × 0.08
Absorption correction	multi-scan
Tmin; Tmax	0.6140; 0.7465
Radiation	MoKα (λ = 0.71073)
2Θ range for data collection/°	4.922 to 66.334°
Completeness to theta	0.996
Index ranges	-10 ≤ h ≤ 11, -17 ≤ k ≤ 17, -19 ≤ l ≤ 19
Reflections collected	33022
Independent reflections	3912 [R_{int} = 0.0370, R_{sigma} = 0.0235]
Data/restraints/parameters	3912/48/157
Goodness-of-fit on F^2	1.165
Final R indexes [I>=2σ (I)]	R_1 = 0.0307, wR_2 = 0.0756
Final R indexes [all data]	R_1 = 0.0340, wR_2 = 0.0774
Largest diff. peak/hole / e Å^{-3}	0.51/-0.60

Crystal structure of compound 7b: CCDC 2392946

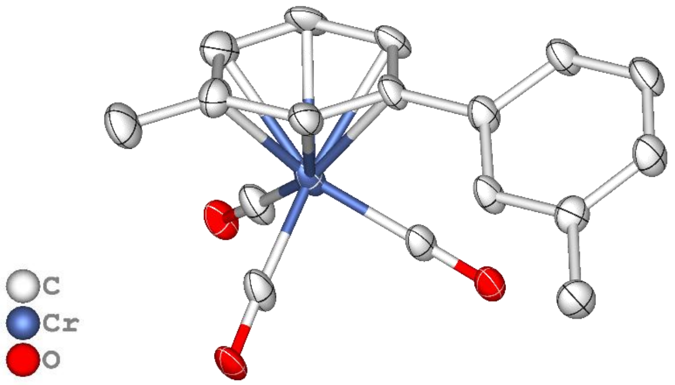

Single crystals were obtained using the slow-evaporation method with n-hexane/DCM as solvent at room temperature. (Hydrogen atoms are omitted for clarity)

Table S19: Crystal data and structure refinement for **7b**.

Identification code	GBUN083, AM-586 // GXray8005	
Crystal Habitus	clear yellow plate	
Device Type	STOE STADIVARI	
Empirical formula	$C_{17}H_{14}CrO_3$	
Moiety formula	C17 H14 Cr O3	
Formula weight	318.28	
Temperature/K	100	
Crystal system	monoclinic	
Space group	$P2_1$	
	a = 10.2291(4) Å	α = 90°
Cell dimensions	b = 7.2727(2) Å	β = 117.389(3)°
	c = 11.1399(5) Å	γ = 90°
Volume/Å^3	735.83(5)	
Z	2	

ρ_{calc} g/cm^3	1.437
μ/mm^{-1}	6.455
F(000)	328.0
Crystal size/mm^3	0.15 × 0.1 × 0.03
Absorption correction	multi-scan
T_{min}; T_{max}	0.0420; 0.6412
Radiation	Cu Kα (λ = 1.54186)
2Θ range for data collection/°	8.94 to 135.472°
Completeness to theta	1.000
Index ranges	-12 ≤ h ≤ 12, -8 ≤ k ≤ 4, -12 ≤ l ≤ 13
Reflections collected	13871
Independent reflections	2005 [R_{int} = 0.0791, R_{sigma} = 0.0582]
Data/restraints/ parameters	2005/1/193
Goodness-of-fit on F^2	2.179
Final R indexes [I>=2σ (I)]	R_1 = 0.2323, wR_2 = 0.4657
Final R indexes [all data]	R_1 = 0.2426, wR_2 = 0.4720
Largest diff. Peak/ hole / e Å^{-3}	2.10/-1.42
Crystal Habitus	0.23(8)

Crystal structure of compound 7c: CCDC 2392947

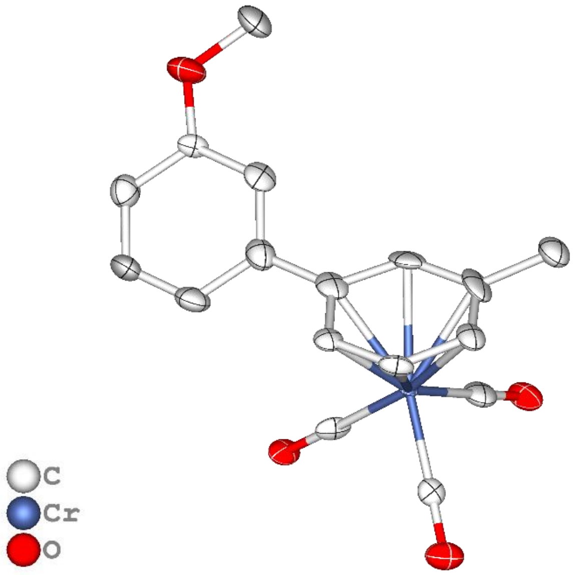

Single crystals were obtained using the slow-evaporation method with n-hexane/DCM as solvent at room temperature. (Hydrogen atoms are omitted for clarity)

Table S20: Crystal data and structure refinement for **7c**.

Identification code	RF577 // GXraymo_8334f	
Crystal Habitus	clear yellow needle	
Device Type	Bruker D8 Venture	
Empirical formula	$C_{17}H_{14}O_4Cr$	
Moiety formula	C17 H14 Cr O4	
Formula weight	334.28	
Temperature/K	100.00	
Crystal system	monoclinic	
Space group	$P2_1/c$	
Cell dimensions	a = 12.379(2) Å	α = 90°
	b = 7.1484(13) Å	β = 91.002(5)°

	c = 33.954(6) Å	γ = 90°
Volume/$Å^3$	3004.2(9)	
Z	8	
ρ_{calc} g/cm^3	1.478	
μ/mm^{-1}	0.776	
F(000)	1376.0	
Crystal size/mm^3	0.24 × 0.08 × 0.04	
Absorption correction	multi-scan	
T_{min}; T_{max}	0.5404; 0.7463	
Radiation	MoKα (λ = 0.71073)	
2Θ range for data collection/°	4.038 to 55.996°	
Completeness to theta	0.999	
Index ranges	-16 ≤ h ≤ 16, -9 ≤ k ≤ 9, -44 ≤ l ≤ 44	
Reflections collected	37897	
Independent reflections	7210 [R_{int} = 0.0882, R_{sigma} = 0.0945]	
Data/restraints/ parameters	7210/0/401	
Goodness-of-fit on F^2	1.272	
Final R indexes [I>=2σ (I)]	R_1 = 0.1367, wR_2 = 0.2961	
Final R indexes [all data]	R_1 = 0.1685, wR_2 = 0.3083	
Largest diff. peak/hole / e Å-3	1.92/-1.07	

Crystal structure of compound 8: CCDC 2392948

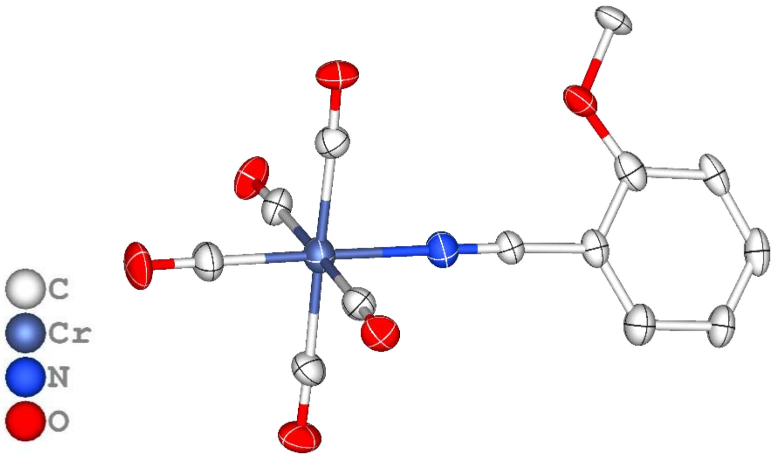

Single crystals were obtained using the slow-evaporation method with benzene as solvent at room temperature. (Hydrogen atoms are omitted for clarity)

Table S21: Crystal data and structure refinement for **8**.

Identification code	GBUN024, RF395 // GXraymo_7361f	
Crystal Habitus	clear yellow plate	
Device Type	Bruker D8 Venture	
Empirical formula	$C_{13}H_7CrNO_6$	
Moiety formula	C13 H7 Cr N O6	
Formula weight	325.20	
Temperature/K	100	
Crystal system	Monoclinic	
Space group	Cc	
	a =14.3064(4) Å	α = 90°
Cell dimensions	b =16.4675(4) Å	β = 116.8550(10)°
	c =7.0173(2) Å	γ = 90°
Volume/Å^3	1474.91(7)	

Z	4
ρ_{calc} g/cm^3	1.464
μ/mm^{-1}	0.800
F(000)	656.0
Crystal size/mm^3	0.32 × 0.31 × 0.11
Absorption correction	multi-scan
T_{min}; T_{max}	0.6536; 0.7462
Radiation	MoKα (λ = 0.71073)
2Θ range for data collection/°	4.948 to 56°
Completeness to theta	0.984
Index ranges	-18 ≤ h ≤ 18, -21 ≤ k ≤ 21, -9 ≤ l ≤ 8
Reflections collected	15319
Independent reflections	3379 [R_{int} = 0.0369, R_{sigma} = 0.0301]
Data/restraints/ parameters	3379/2/192
Goodness-of-fit on F^2	1.055
Final R indexes [I>=2σ (I)]	R_1 = 0.0250, wR_2 = 0.0653
Final R indexes [all data]	R_1 = 0.0263, wR_2 = 0.0667
Largest diff. Peak/ hole / e Å^{-3}	0.17/-0.15
Flack Parameter	0.44(2)

4. NMR Spectra of (η^6-Arene)$Cr(CO)_3$ Complexes

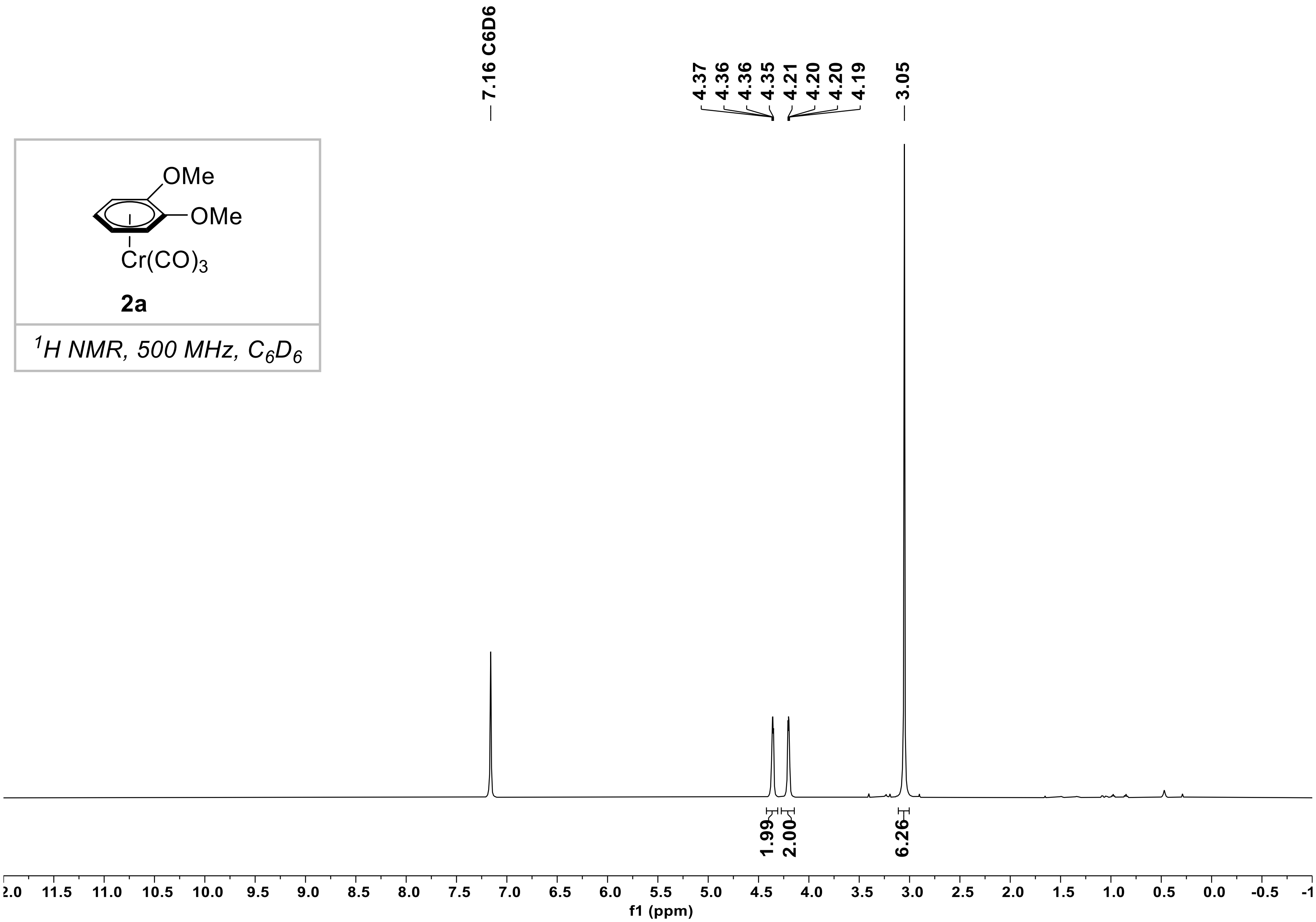
7.16 C6D6
4.37
4.36
4.36
4.35
4.21
4.20
4.20
4.19
3.05
OMe
OMe
Cr(CO)3
2a
1H NMR, 500 MHz, C6D6
1.99
2.00
6.26
f1 (ppm)

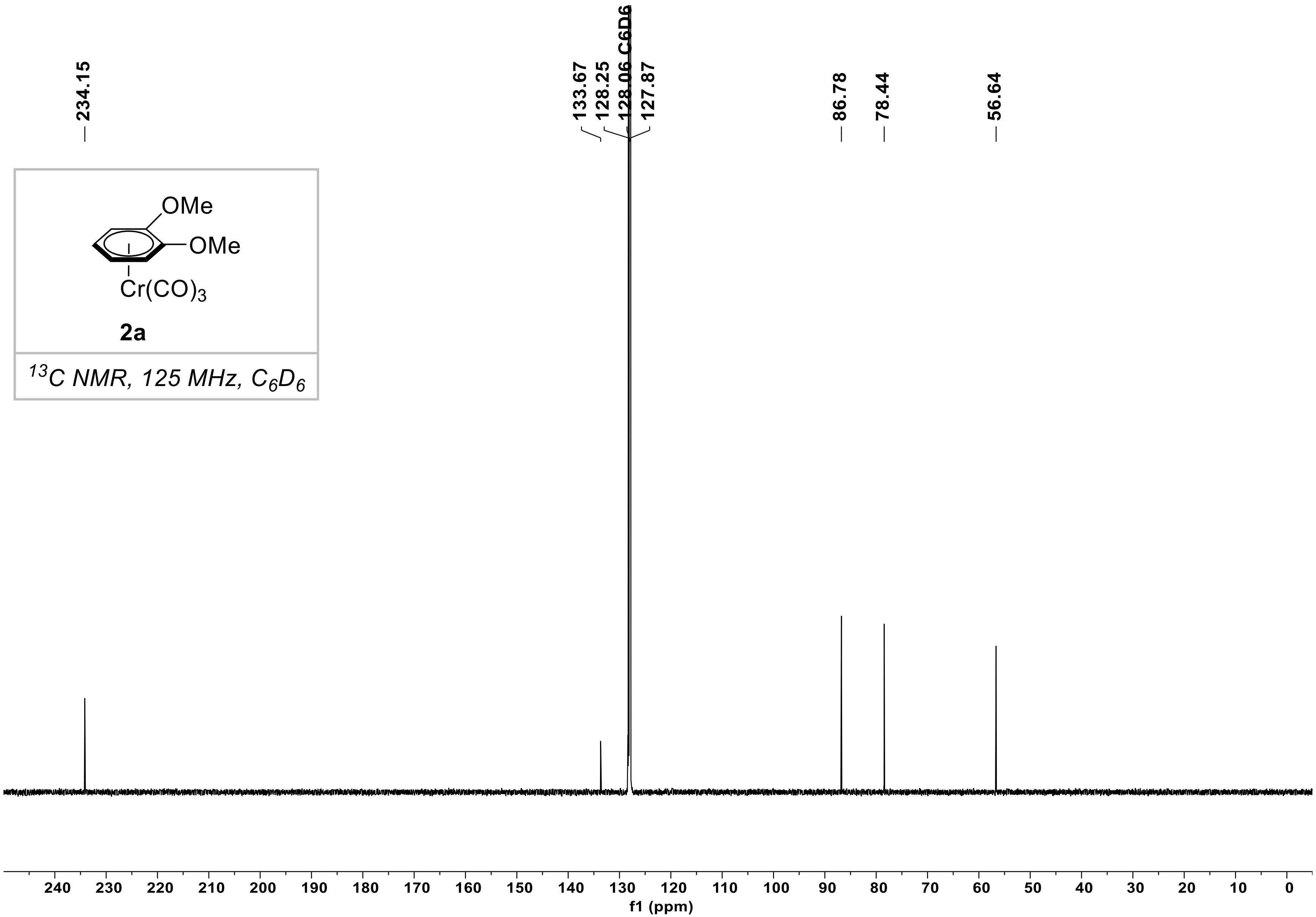
234.15
133.67
128.25
128.06 C6D6
127.87
86.78
78.44
56.64
OMe
OMe
Cr(CO)3
2a
13C NMR, 125 MHz, C6D6
240
230
220
210
200
190
180
170
160
150
140
130
120
110
100
90
80
70
60
50
40
30
20
10
0
f1 (ppm)

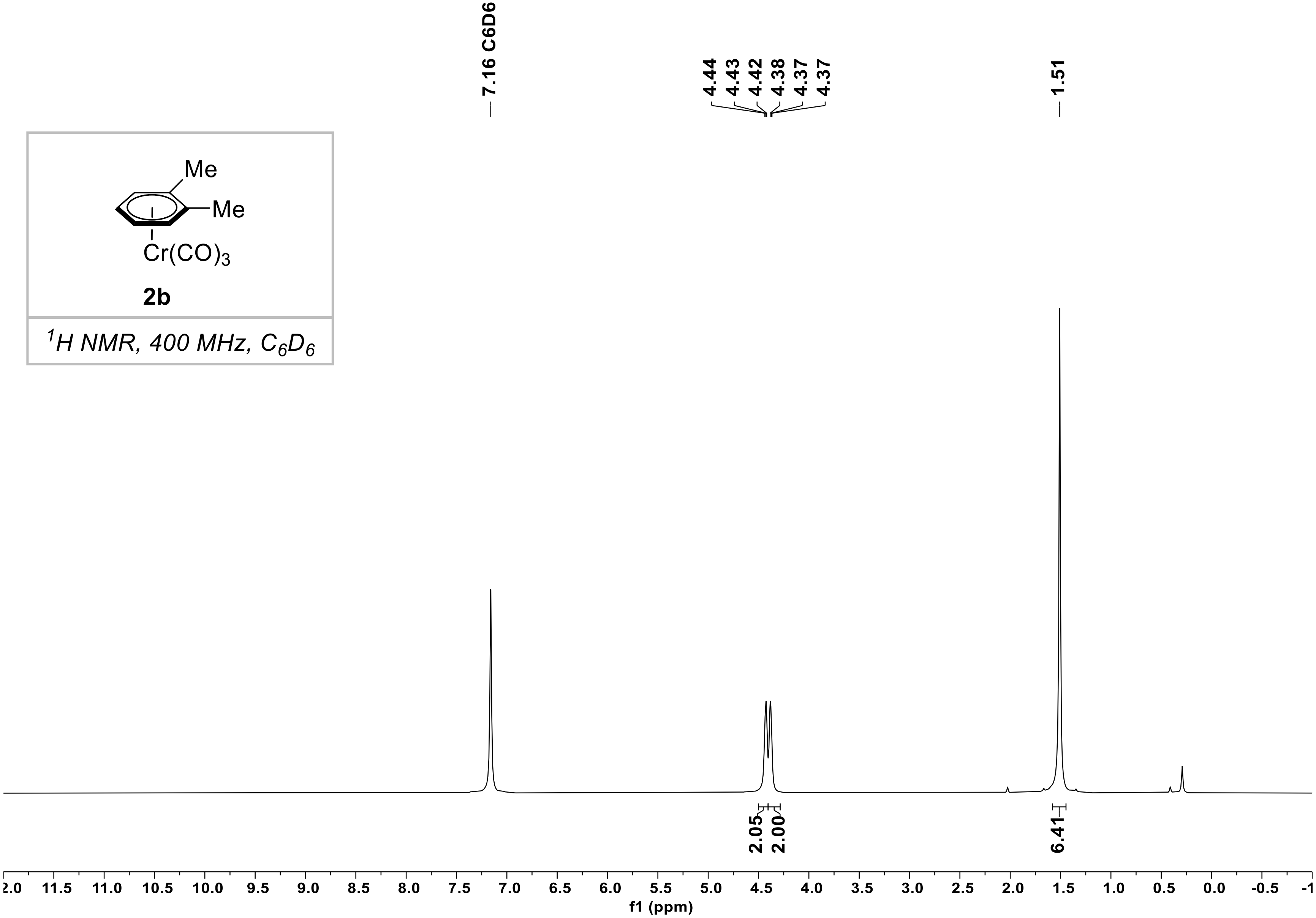

2b

^1H NMR, 400 MHz, C_6D_6

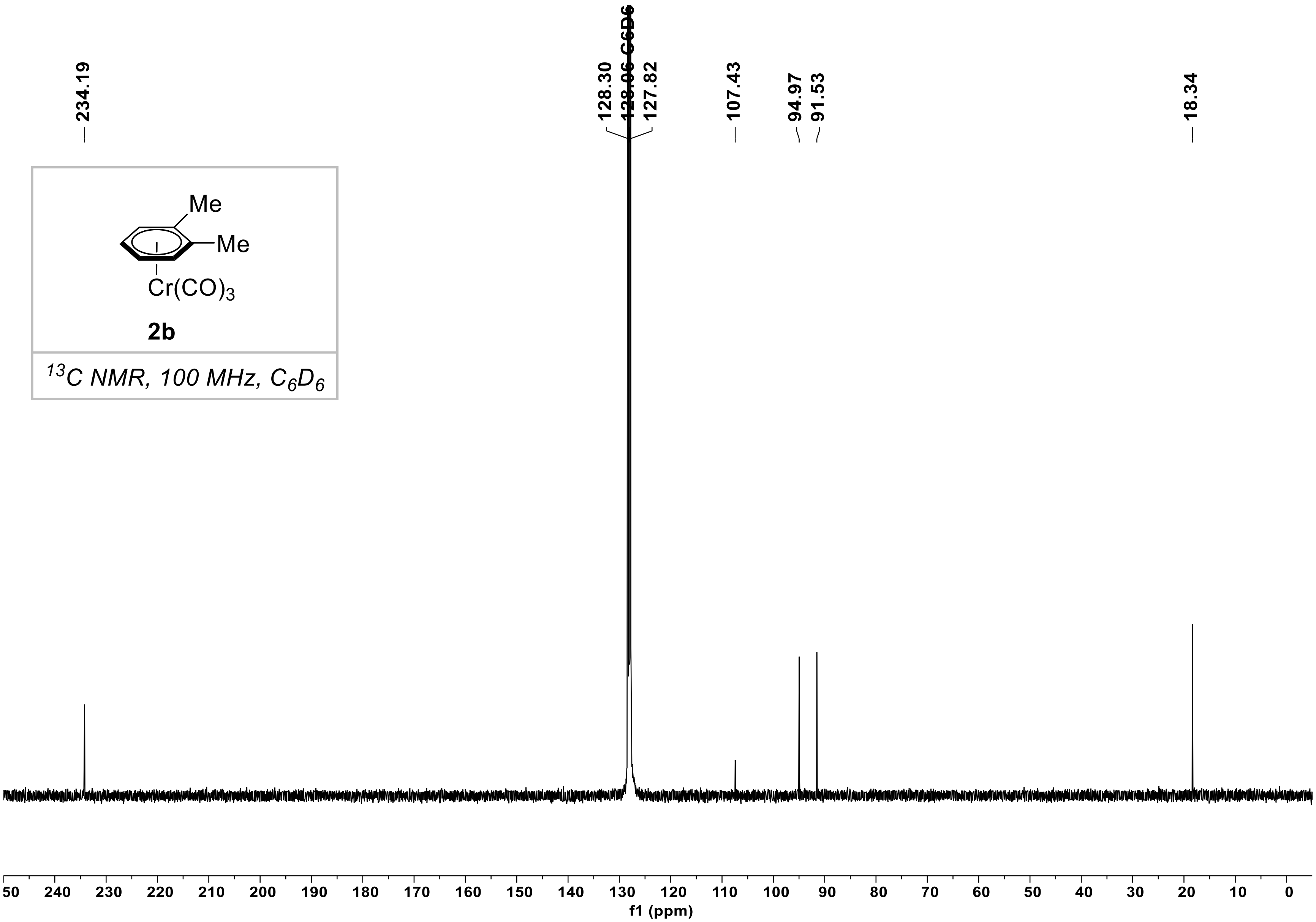

234.19
128.30
128.06 C6D6
127.82
107.43
94.97
91.53
18.34
Me
Me
Cr(CO)3
2b
13C NMR, 100 MHz, C6D6
250 240 230 220 210 200 190 180 170 160 150 140 130 120 110 100 90 80 70 60 50 40 30 20 10 0
f1 (ppm)

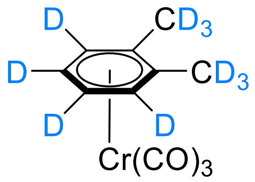

2b-D$_{10}$

^2H NMR, 77 MHz, C_6H_6

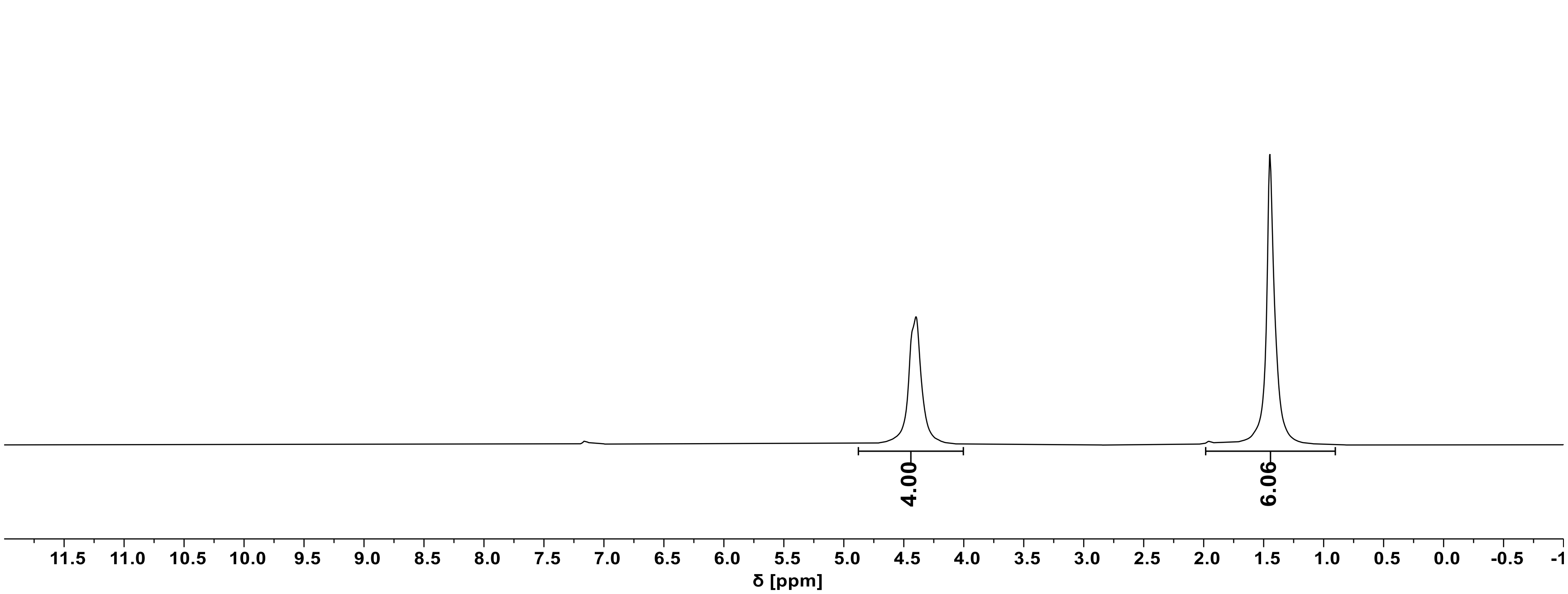

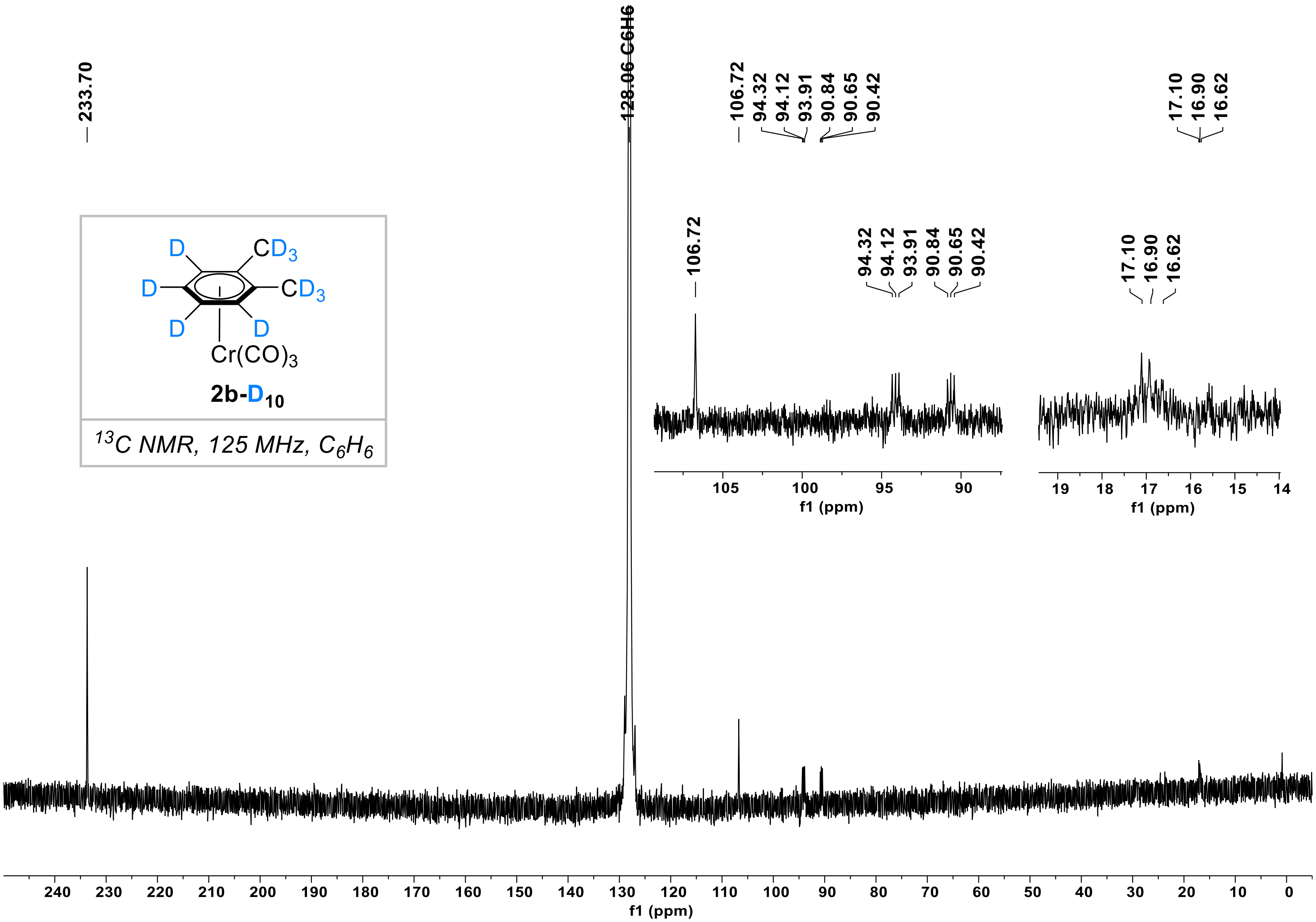
233.70
128.06 C6H6
106.72
94.32
94.12
93.91
90.84
90.65
90.42
17.10
16.90
16.62
D
CD3
D
CD3
D
D
Cr(CO)3
2b-D10
13C NMR, 125 MHz, C6H6
105
100
95
90
f1 (ppm)
19
18
17
16
15
14
f1 (ppm)
240
230
220
210
200
190
180
170
160
150
140
130
120
110
100
90
80
70
60
50
40
30
20
10
0
f1 (ppm)

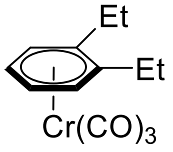

2c

^1H NMR, 400 MHz, C_6D_6

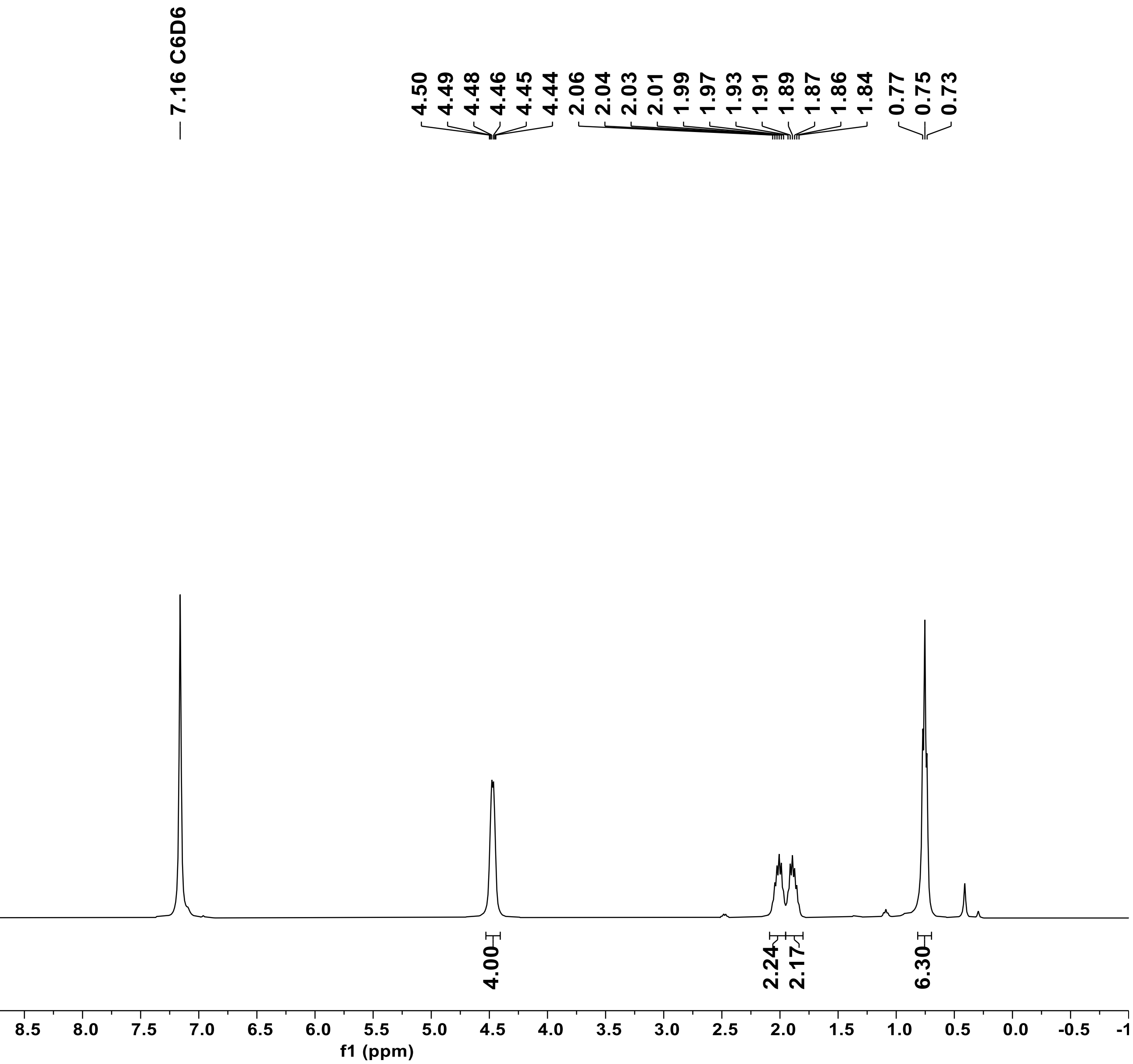

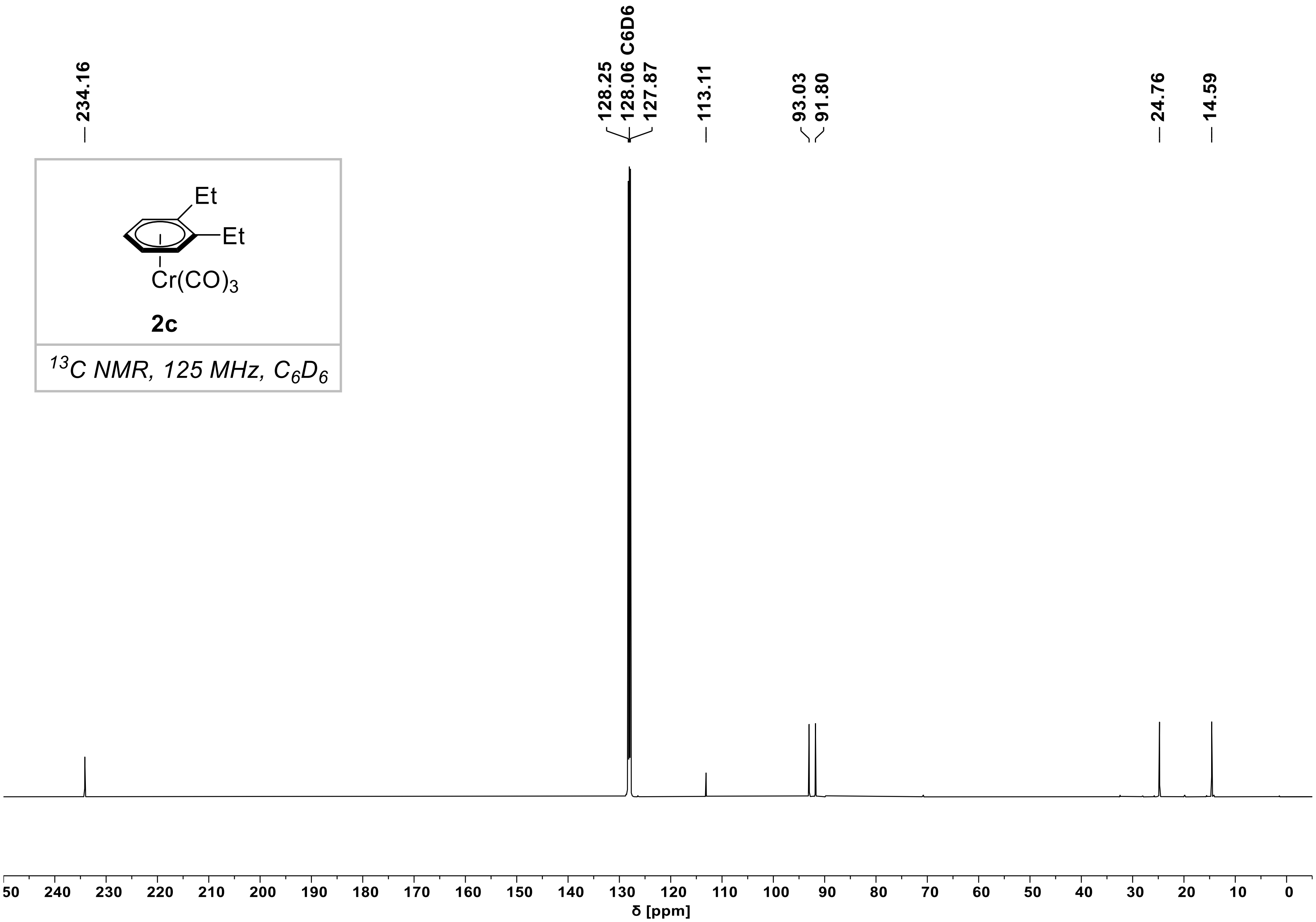

234.16
128.25
128.06 C6D6
127.87
113.11
93.03
91.80
24.76
14.59
Et
Et
Cr(CO)3
2c
13C NMR, 125 MHz, C6D6
50
240
230
220
210
200
190
180
170
160
150
140
130
120
110
100
90
80
70
60
50
40
30
20
10
0
δ [ppm]

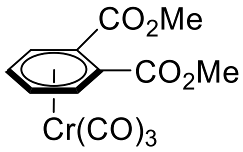

2d

¹H NMR, 500 MHz, C_6D_6

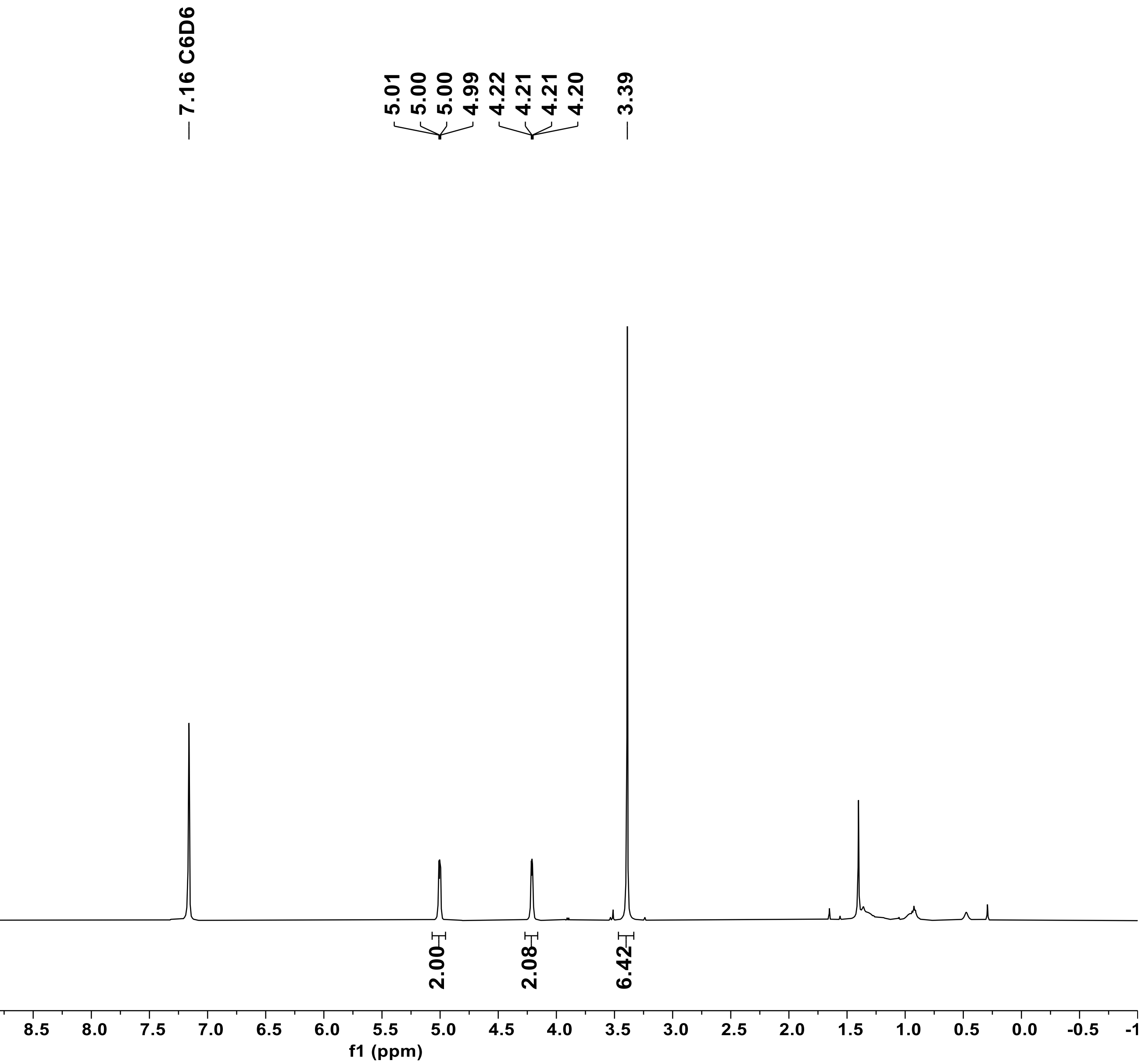

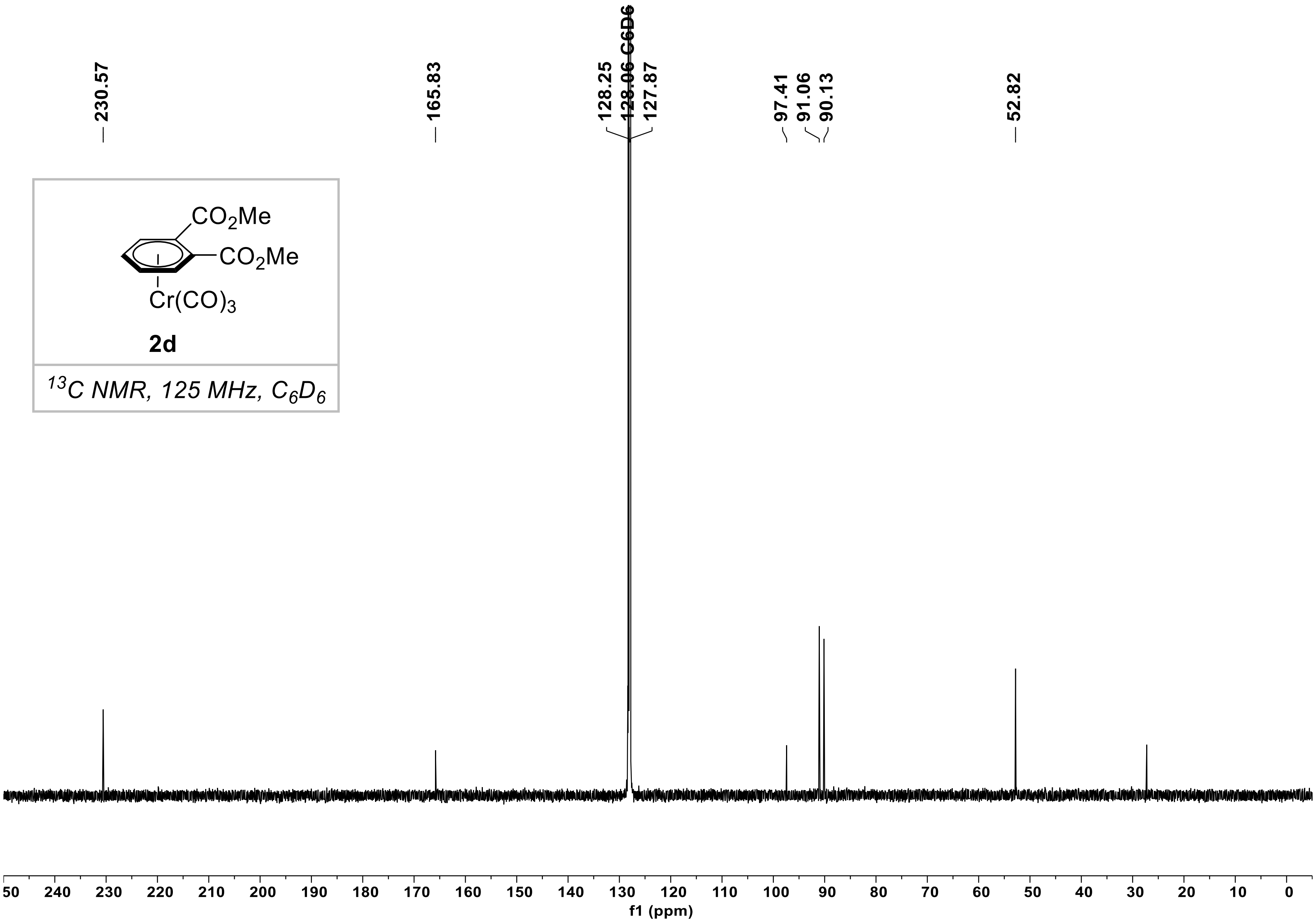
230.57
165.83
128.25
128.06 C6D6
127.87
97.41
91.06
90.13
52.82
CO2Me
CO2Me
Cr(CO)3
2d
13C NMR, 125 MHz, C6D6
50 240 230 220 210 200 190 180 170 160 150 140 130 120 110 100 90 80 70 60 50 40 30 20 10 0
f1 (ppm)

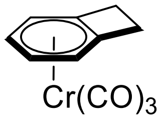

2e

^{1}H NMR, 500 MHz, C_6D_6

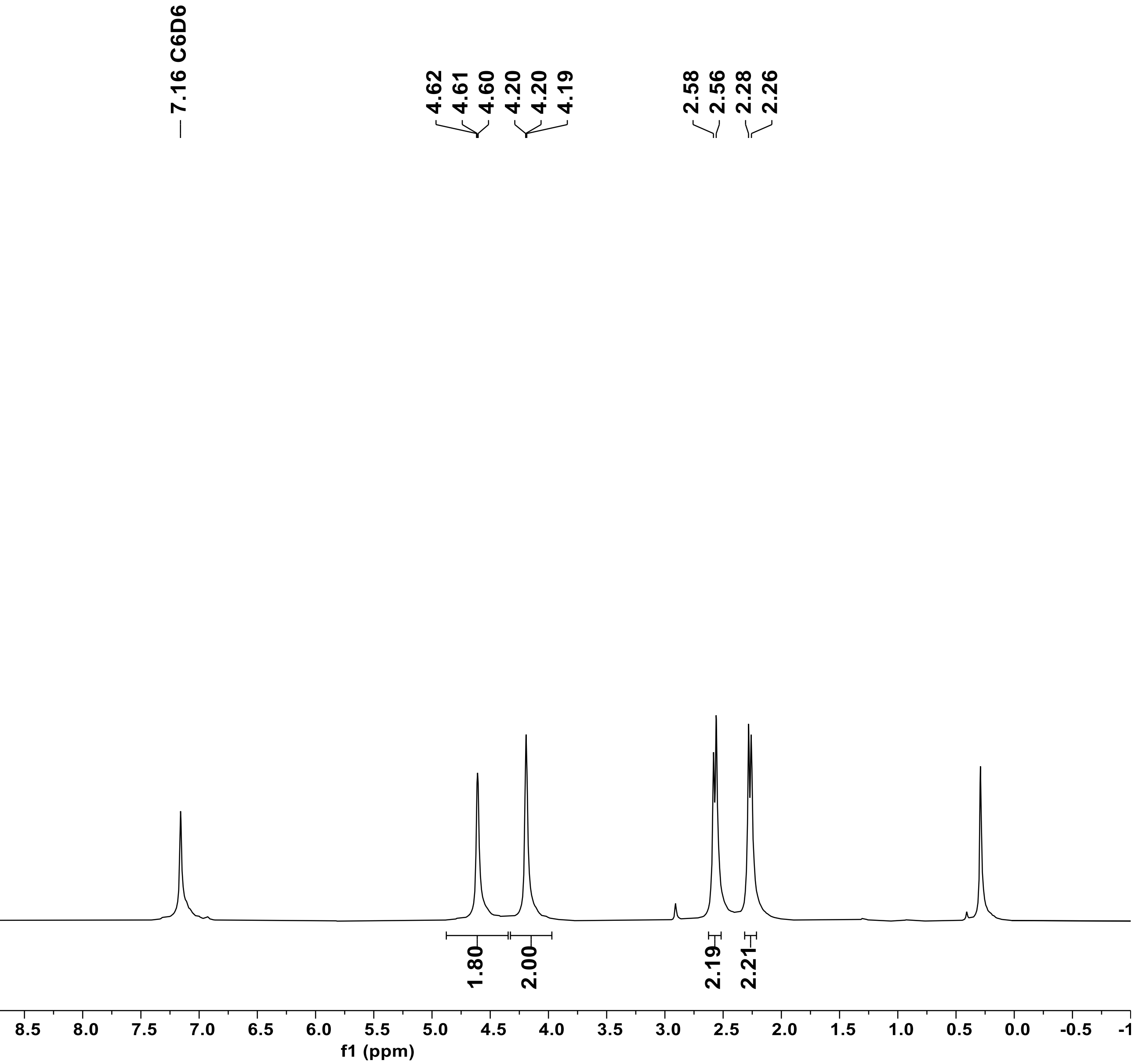

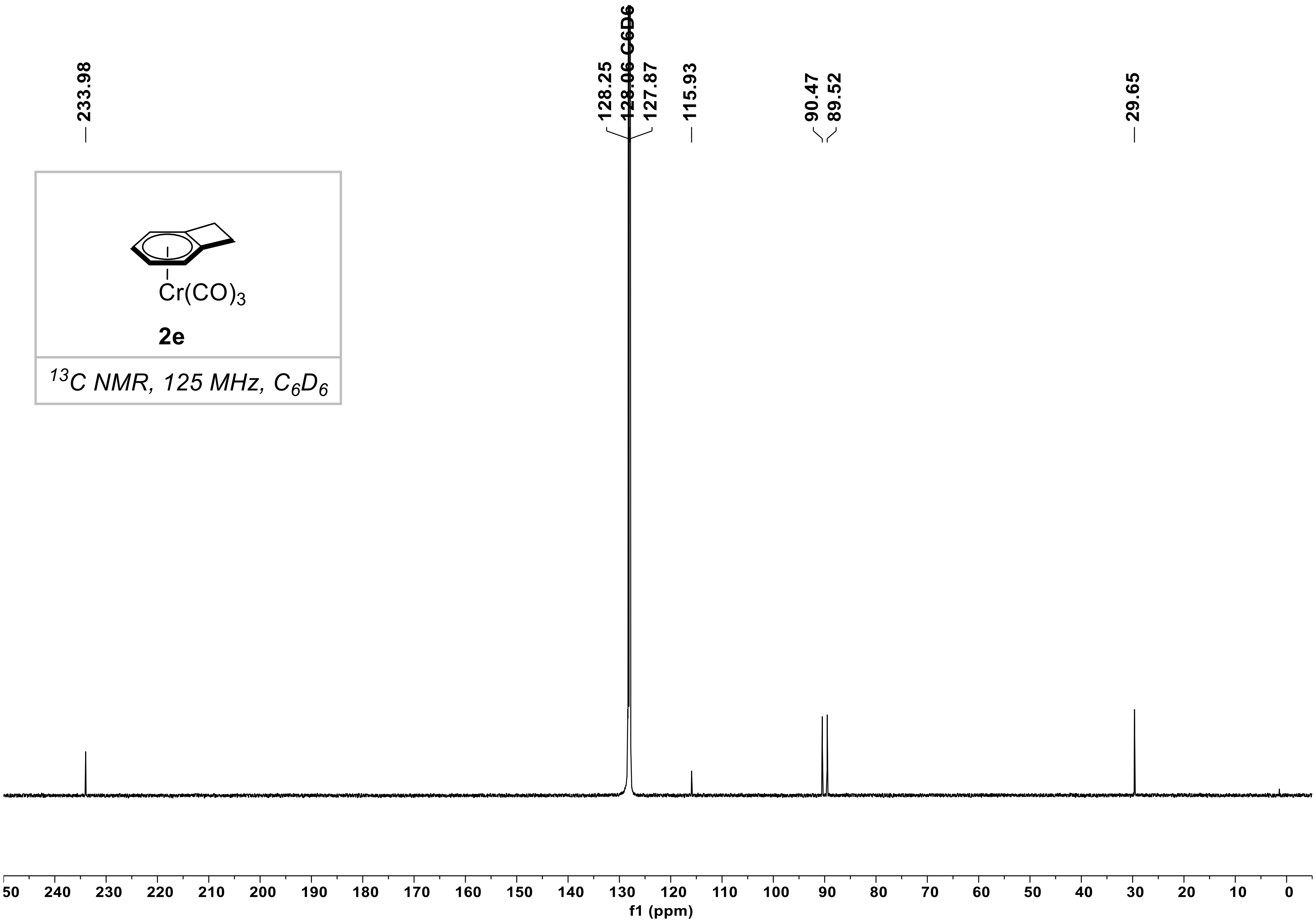
233.98
128.25
128.06 C6D6
127.87
115.93
90.47
89.52
29.65
Cr(CO)3
2e
13C NMR, 125 MHz, C6D6
50
240
230
220
210
200
190
180
170
160
150
140
130
120
110
100
90
80
70
60
50
40
30
20
10
0
f1 (ppm)

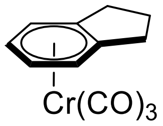

2f

^{1}H NMR, 500 MHz, C_6D_6

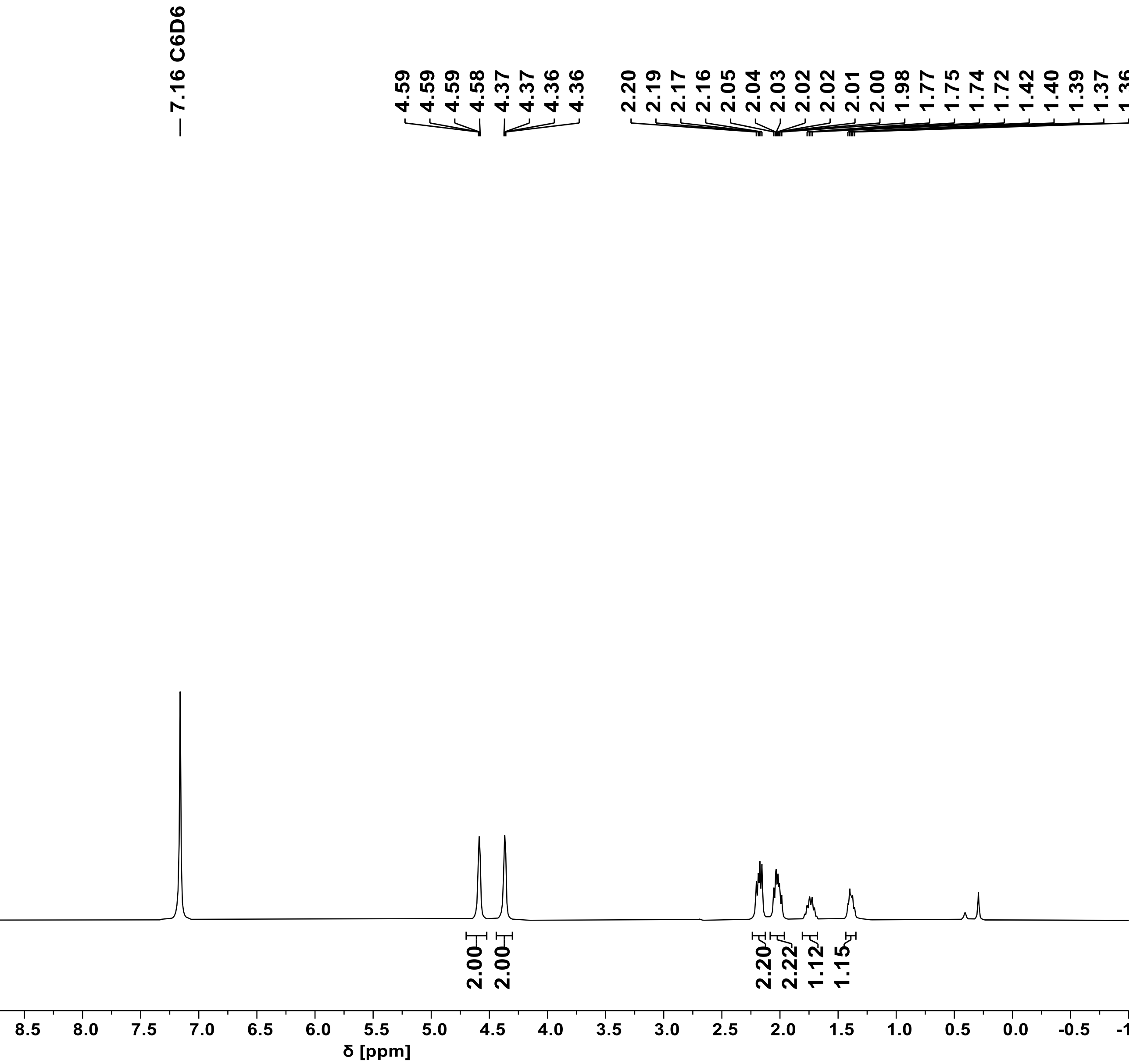

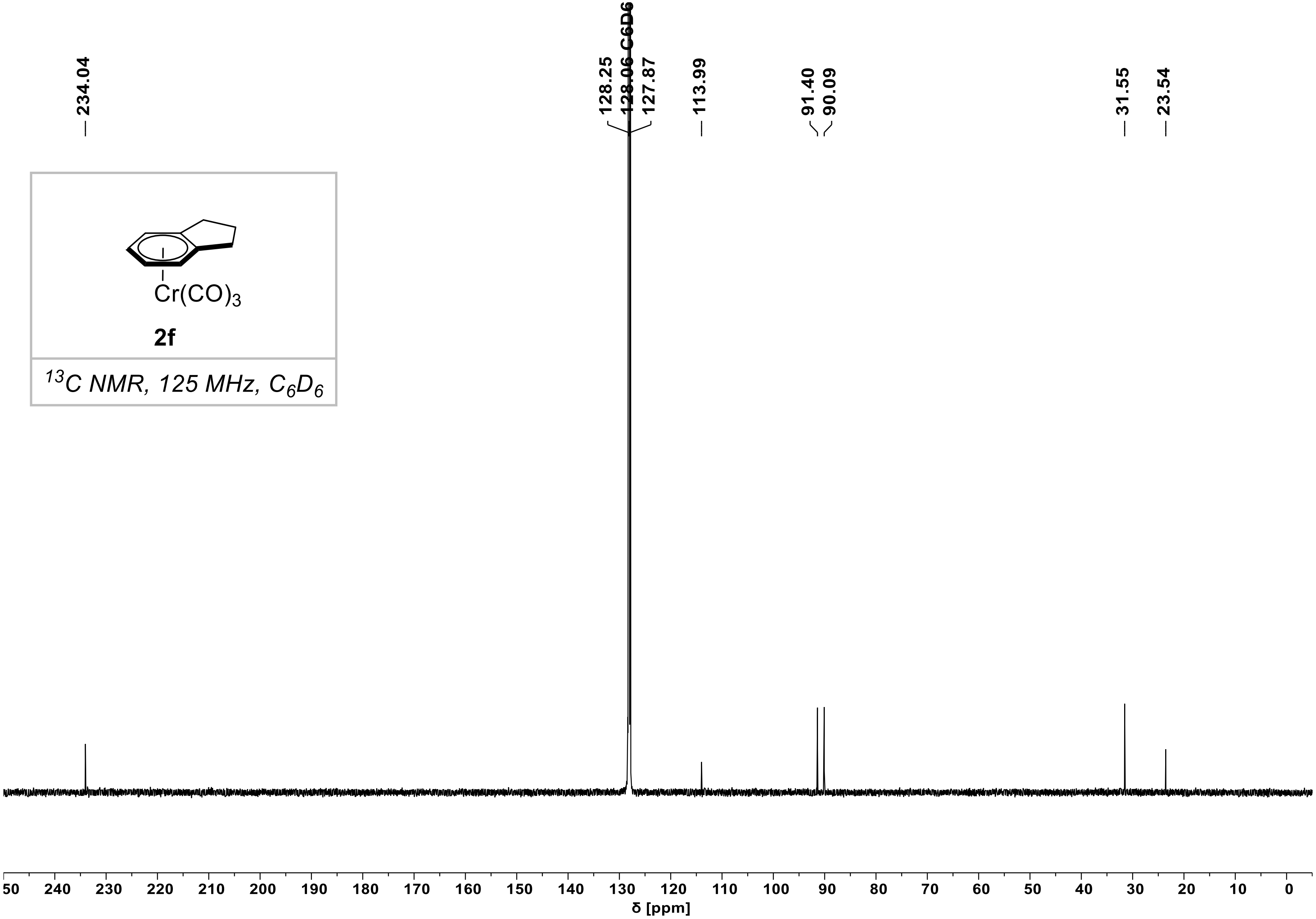

234.04
128.25
128.06 C6D6
127.87
113.99
91.40
90.09
31.55
23.54
Cr(CO)3
2f
13C NMR, 125 MHz, C6D6
250 240 230 220 210 200 190 180 170 160 150 140 130 120 110 100 90 80 70 60 50 40 30 20 10 0
δ [ppm]

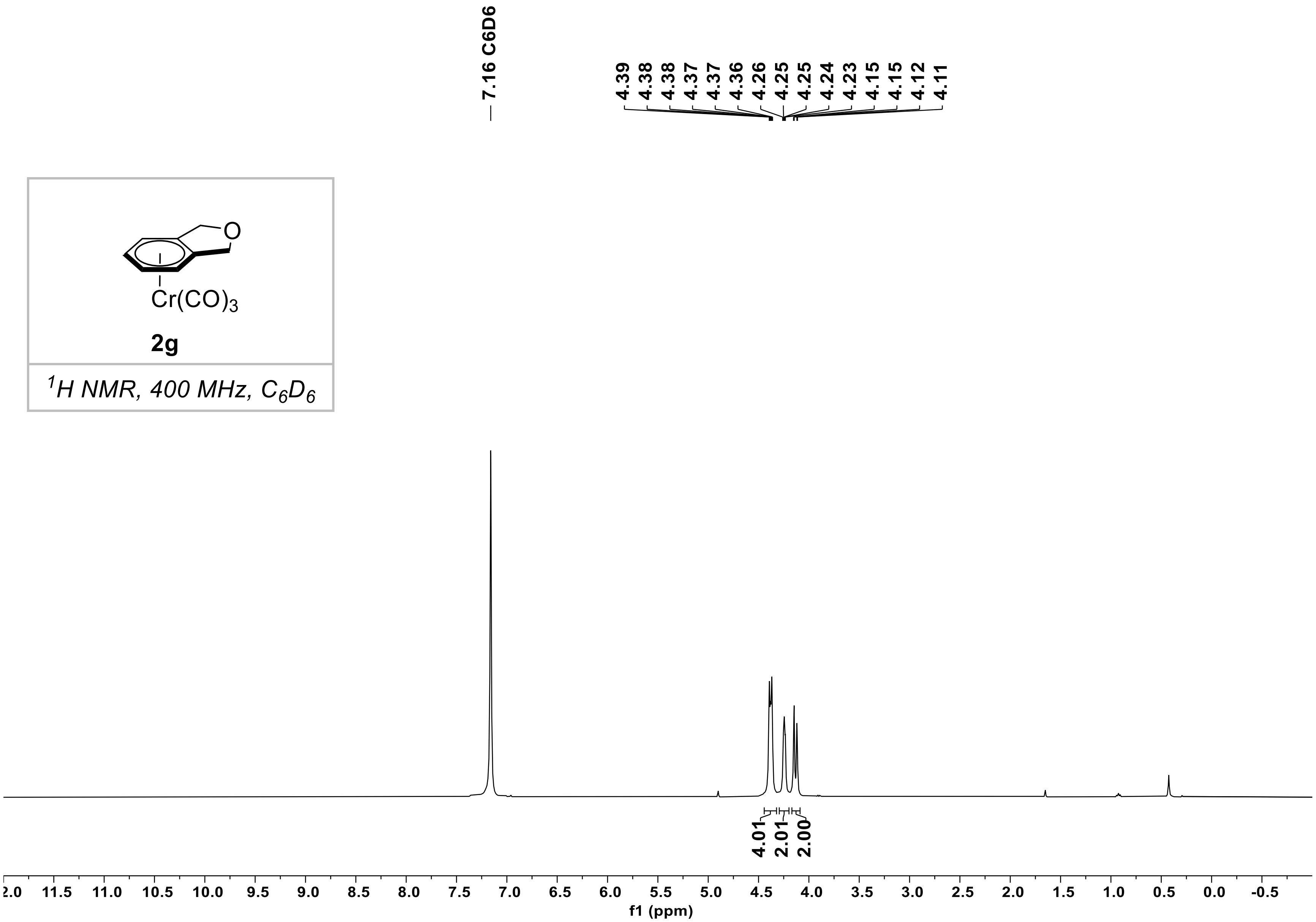
7.16 C6D6
4.39
4.38
4.38
4.37
4.37
4.36
4.26
4.25
4.25
4.24
4.23
4.15
4.15
4.12
4.11
Cr(CO)3
2g
1H NMR, 400 MHz, C6D6
4.01
2.01
2.00
12.0 11.5 11.0 10.5 10.0 9.5 9.0 8.5 8.0 7.5 7.0 6.5 6.0 5.5 5.0 4.5 4.0 3.5 3.0 2.5 2.0 1.5 1.0 0.5 0.0 -0.5
f1 (ppm)

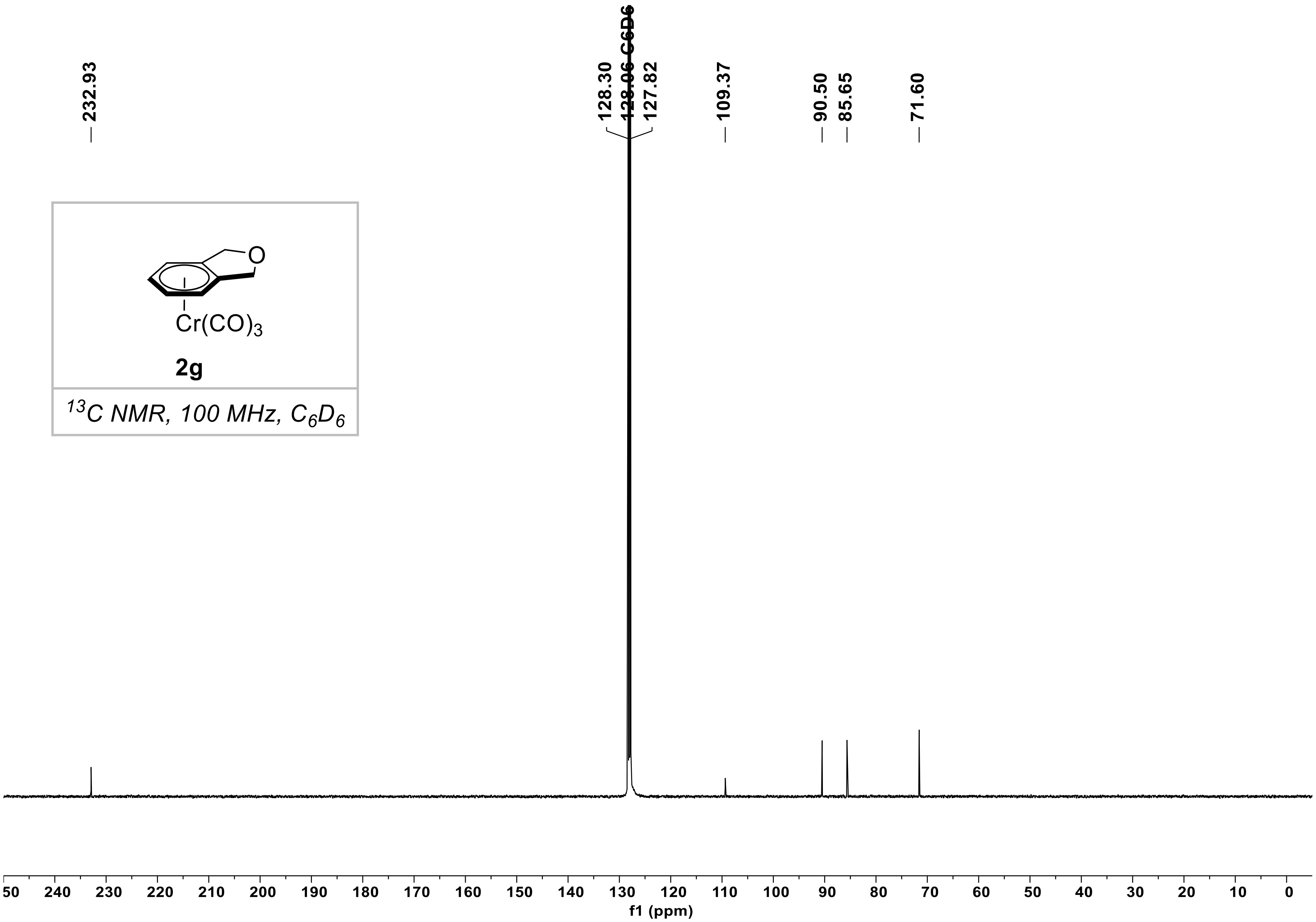

2g

^{13}C NMR, 100 MHz, C_6D_6

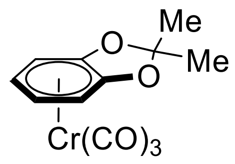

2h

^{1}H NMR, 400 MHz, C_6D_6

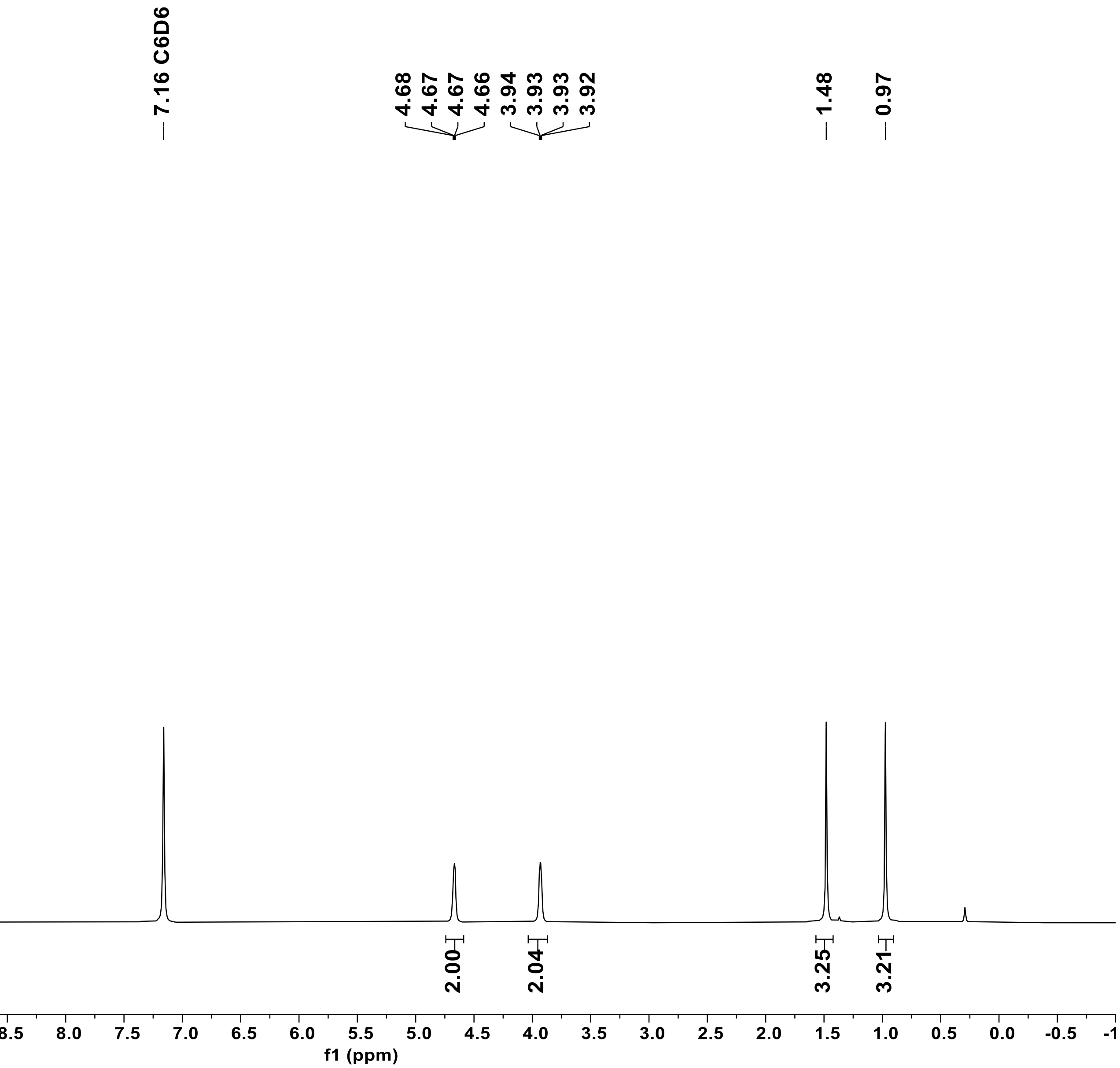

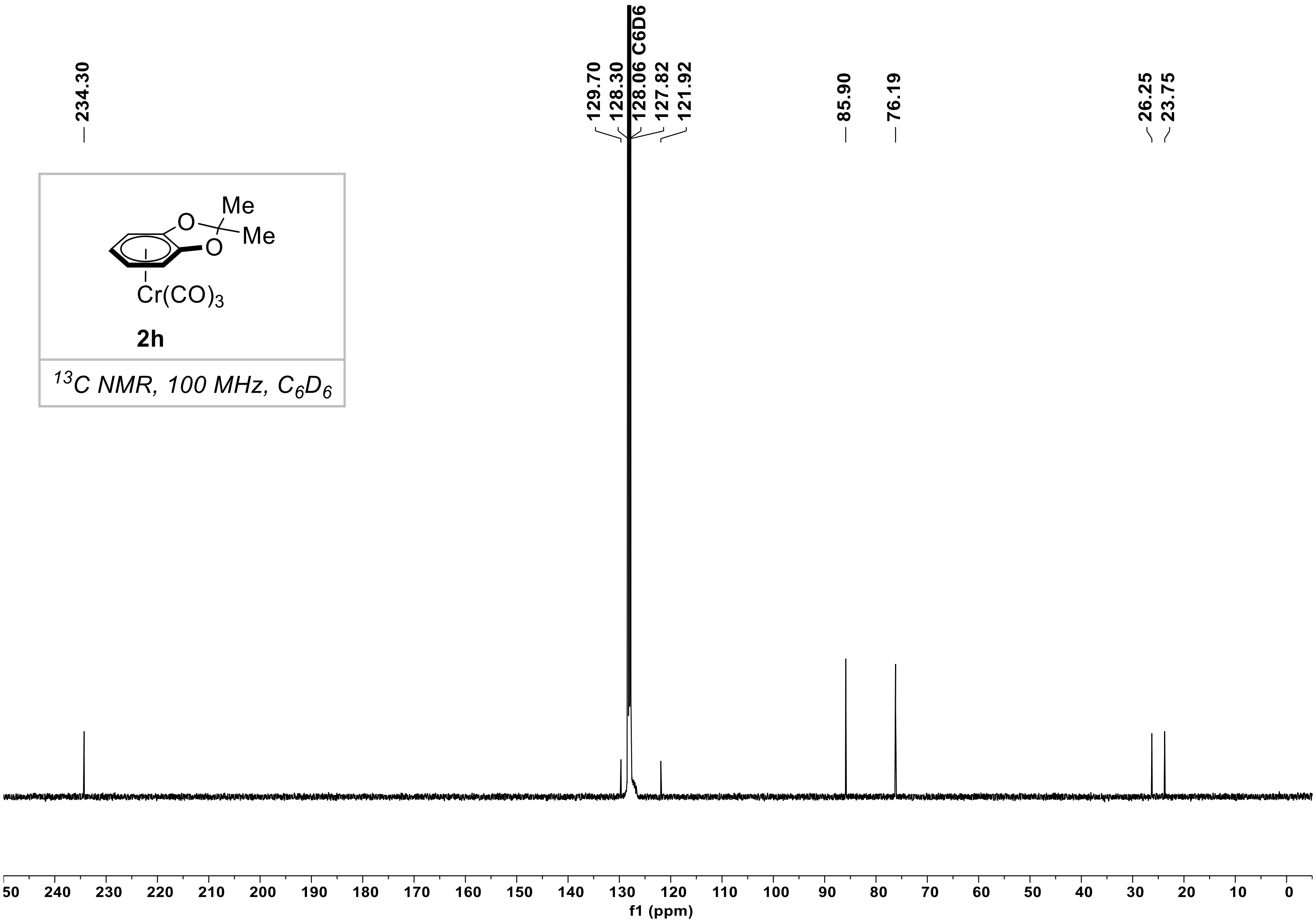
Me
O
O
Me
Cr(CO)3
2h
13C NMR, 100 MHz, C6D6
234.30
129.70
128.30
128.06 C6D6
127.82
121.92
85.90
76.19
26.25
23.75
50
240
230
220
210
200
190
180
170
160
150
140
130
120
110
100
90
80
70
60
50
40
30
20
10
0
f1 (ppm)

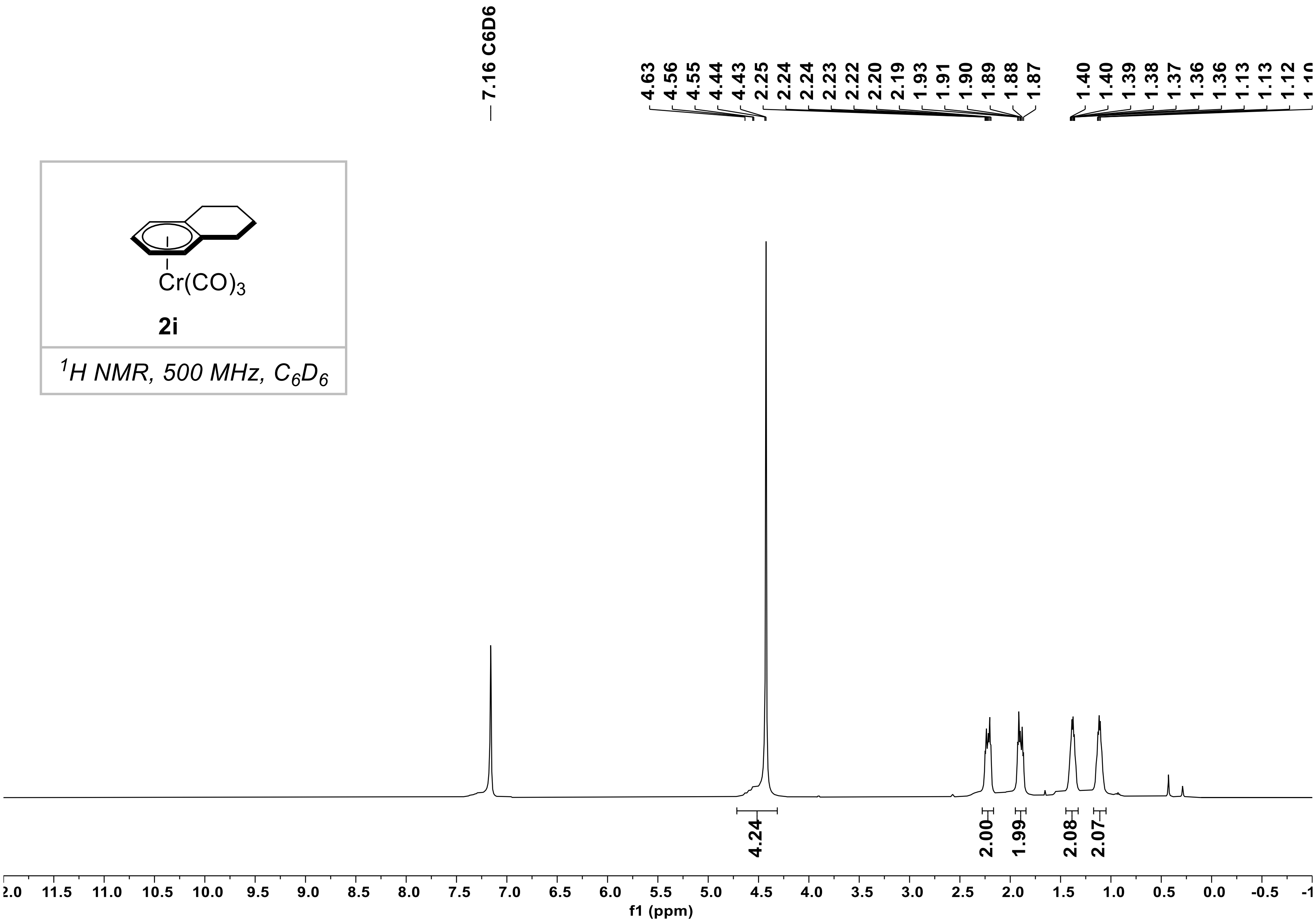
7.16 C6D6
4.63
4.56
4.55
4.44
4.43
2.25
2.24
2.24
2.23
2.22
2.20
2.19
1.93
1.91
1.90
1.89
1.88
1.87
1.40
1.40
1.39
1.38
1.37
1.36
1.36
1.13
1.13
1.12
Cr(CO)3
2i
1H NMR, 500 MHz, C6D6
4.24
2.00
1.99
2.08
2.07
11.5
11.0
10.5
10.0
9.5
9.0
8.5
8.0
7.5
7.0
6.5
6.0
5.5
5.0
4.5
4.0
3.5
3.0
2.5
2.0
1.5
1.0
0.5
0.0
-0.5
f1 (ppm)

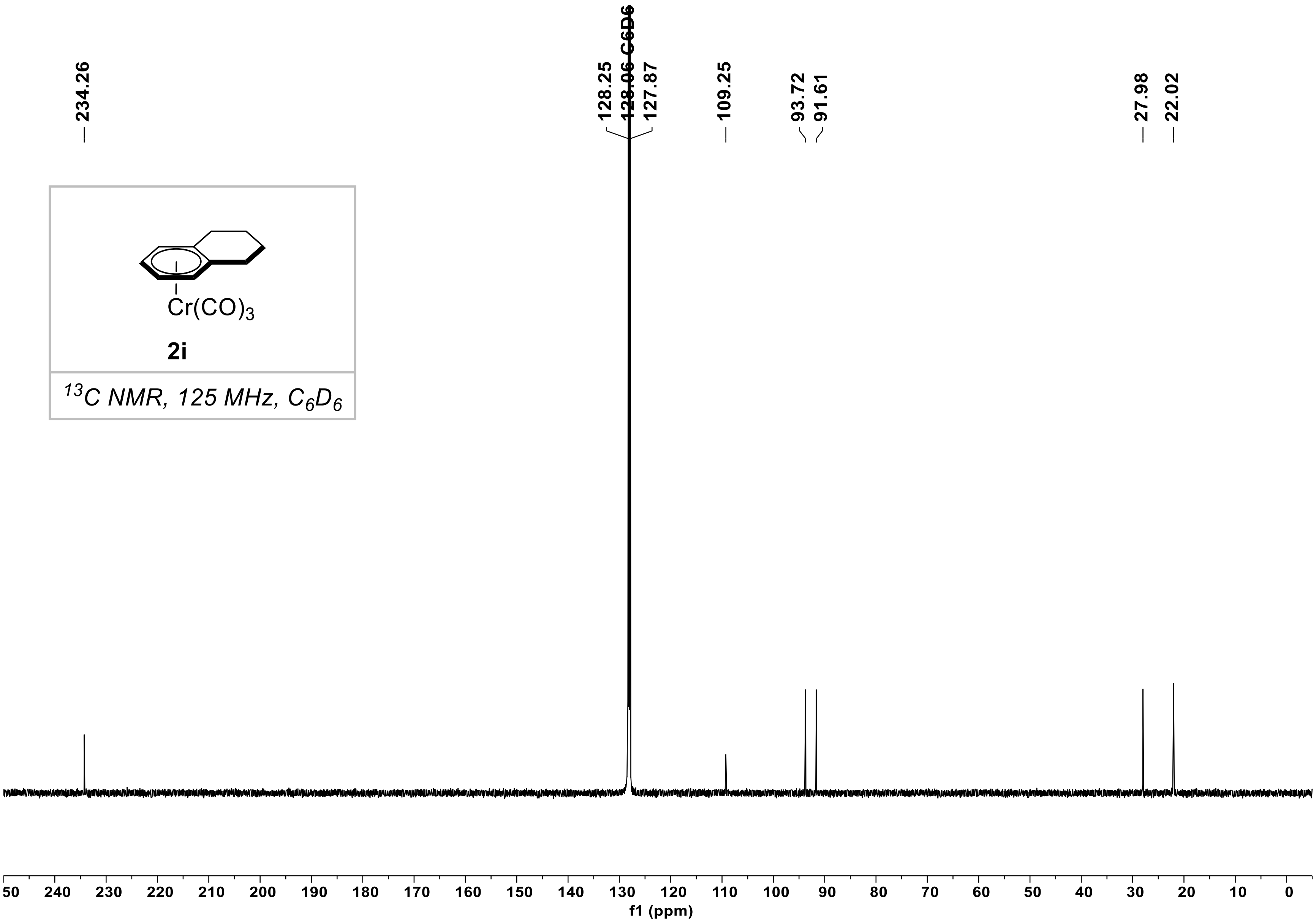
234.26
128.25
128.06 C6D6
127.87
109.25
93.72
91.61
27.98
22.02
Cr(CO)3
2i
13C NMR, 125 MHz, C6D6
250 240 230 220 210 200 190 180 170 160 150 140 130 120 110 100 90 80 70 60 50 40 30 20 10 0
f1 (ppm)

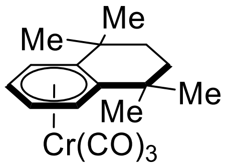

2j

^1H NMR, 500 MHz, C_6D_6

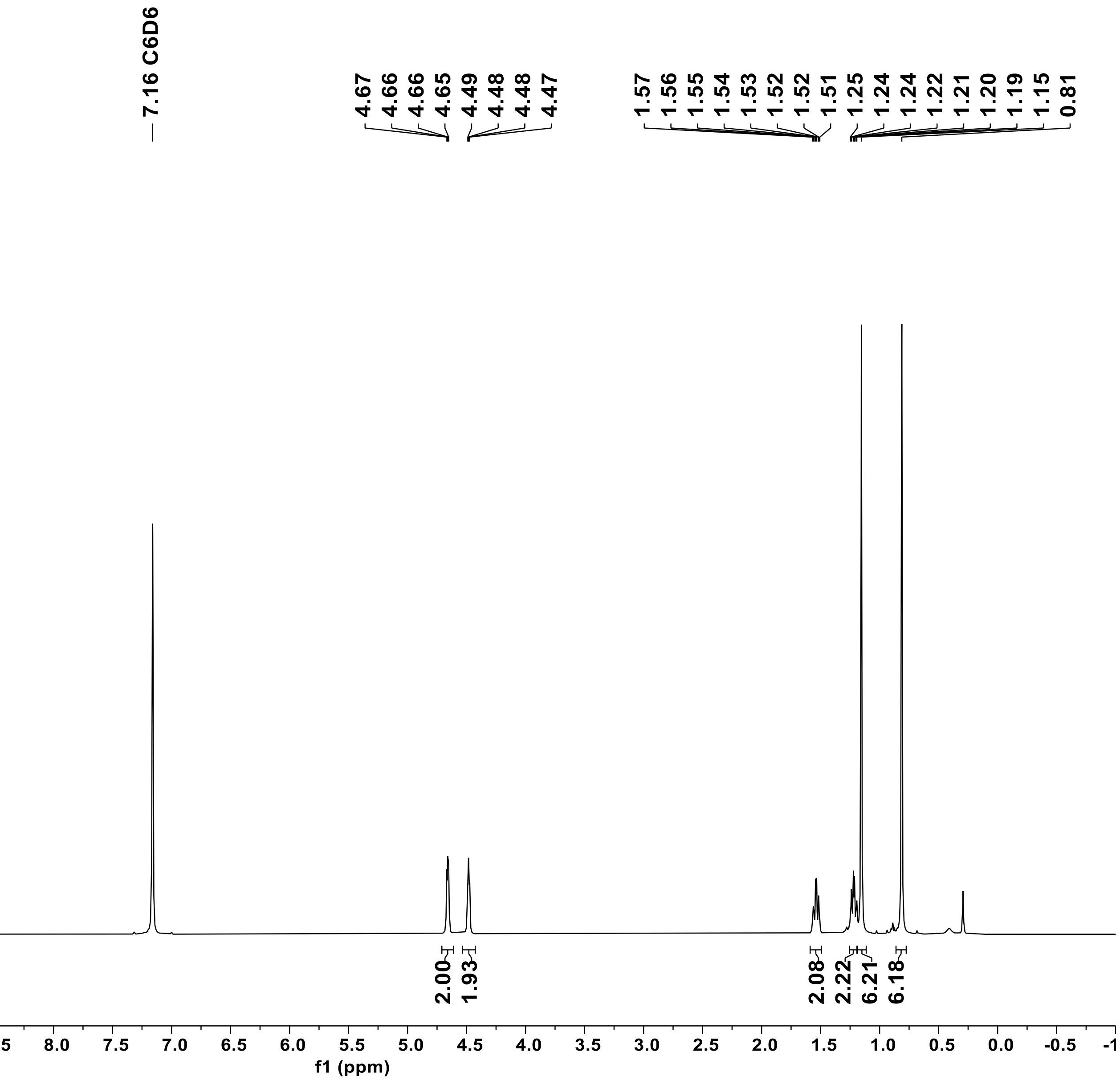

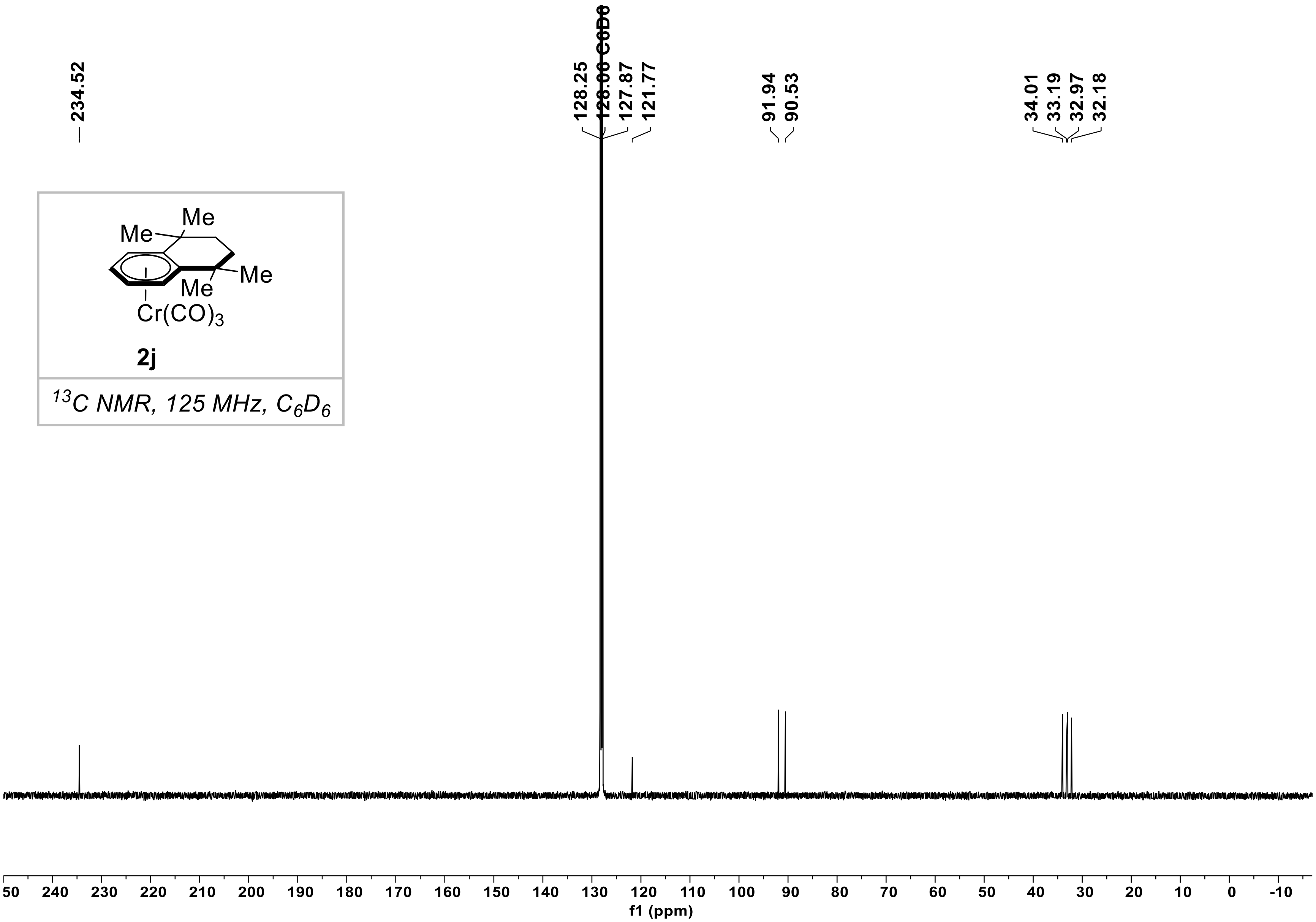

234.52
128.25
128.06 C6D6
127.87
121.77
91.94
90.53
34.01
33.19
32.97
32.18
Me
Me
Me
Me
Cr(CO)3
2j
13C NMR, 125 MHz, C6D6
50 240 230 220 210 200 190 180 170 160 150 140 130 120 110 100 90 80 70 60 50 40 30 20 10 0 -10
f1 (ppm)

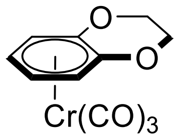

2k

^{1}H NMR, 500 MHz, C_6D_6

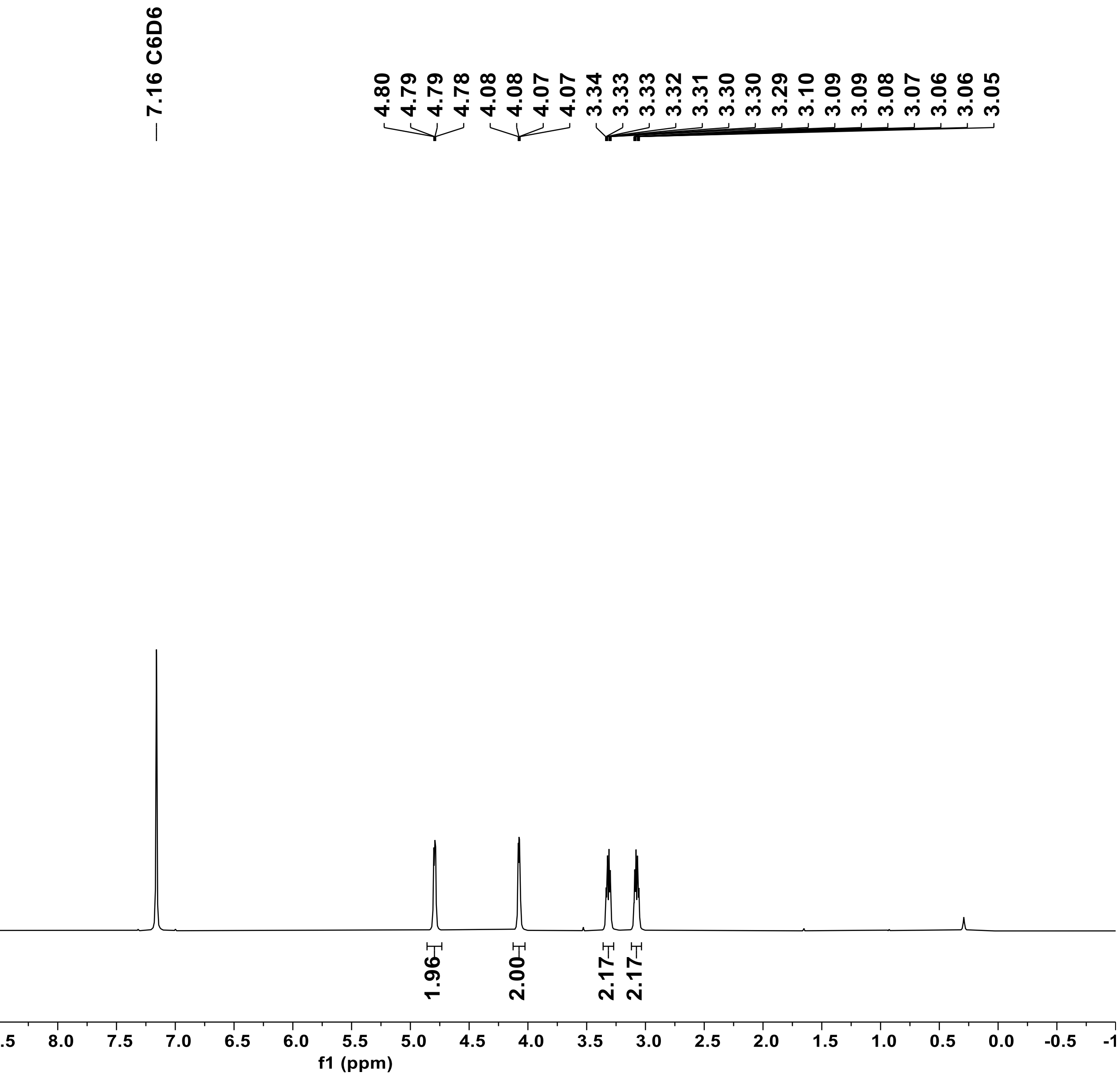

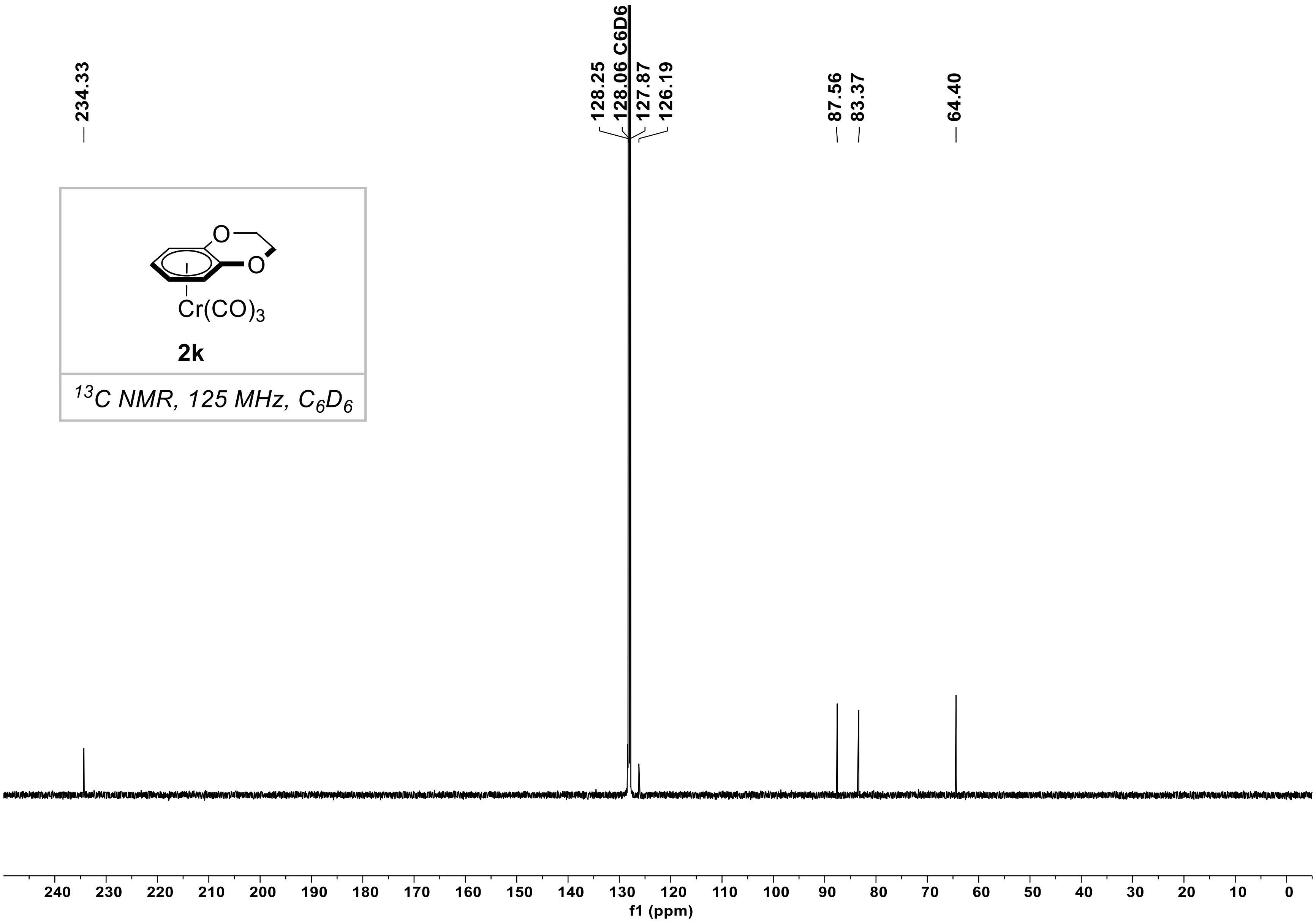

2k

^{13}*C NMR, 125 MHz,* C_6D_6

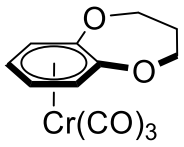

2l

^{1}H NMR, 400 MHz, C_6D_6

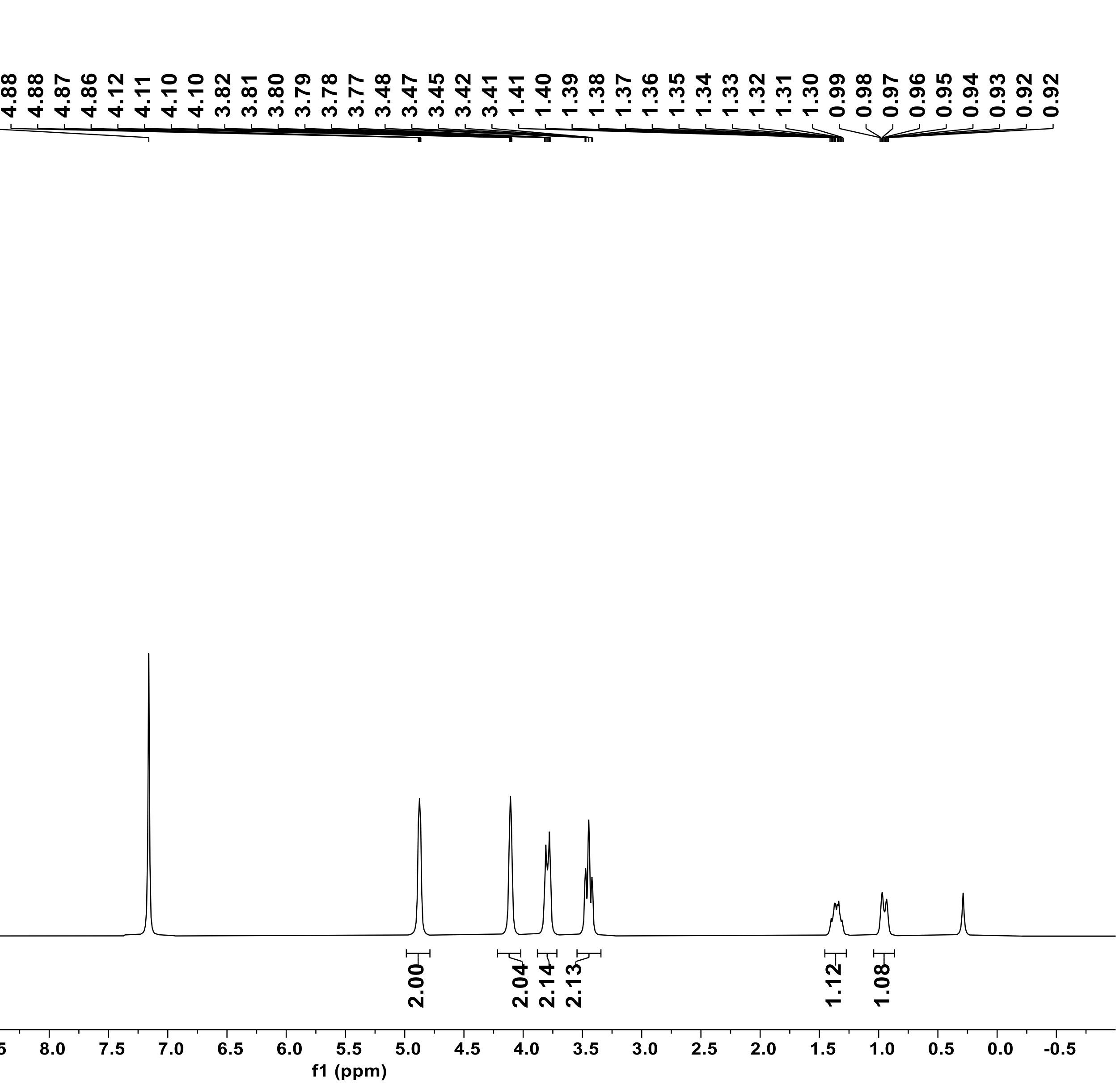

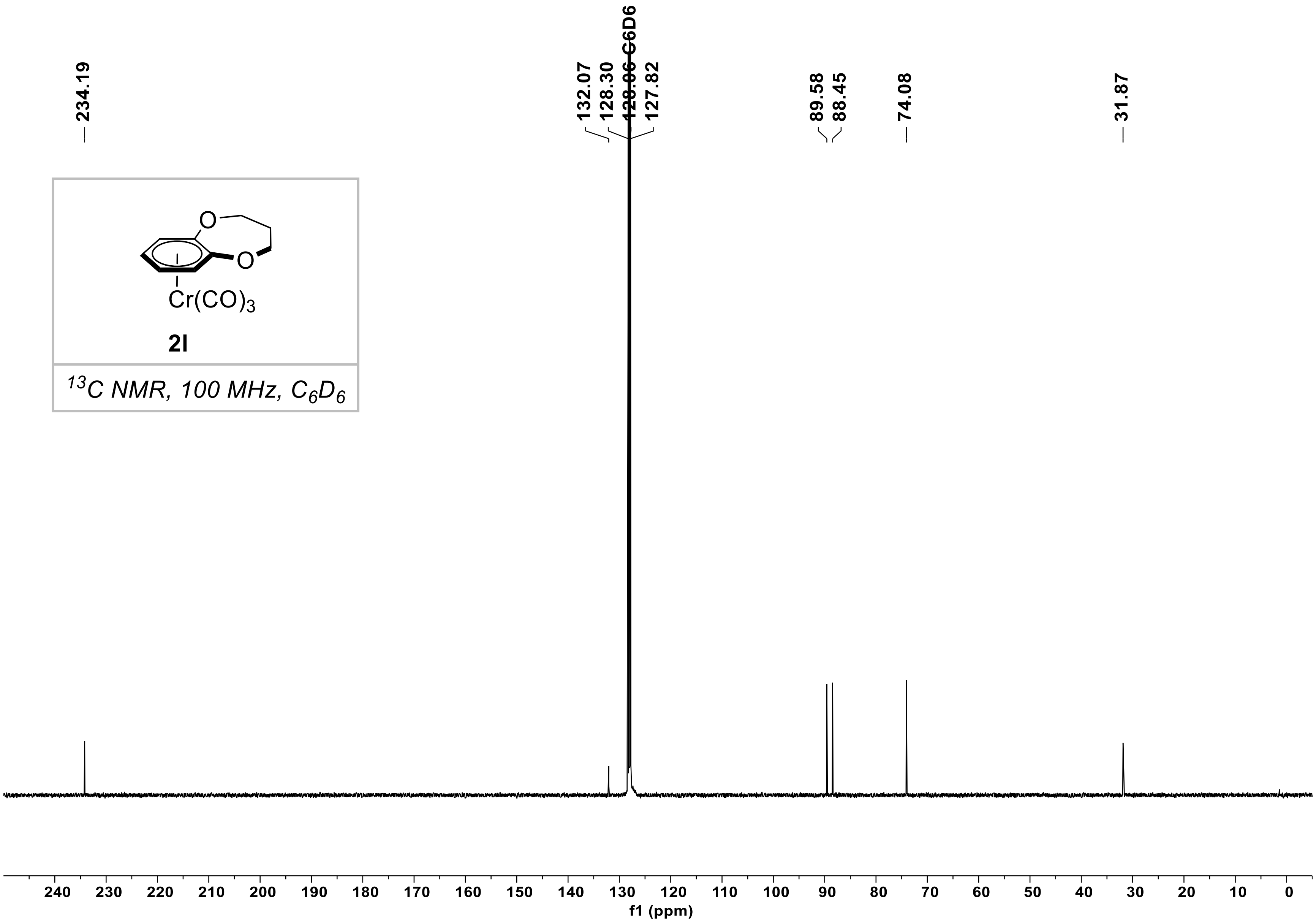
234.19
132.07
128.30
128.06 C6D6
127.82
89.58
88.45
74.08
31.87
Cr(CO)3
2l
13C NMR, 100 MHz, C6D6
240 230 220 210 200 190 180 170 160 150 140 130 120 110 100 90 80 70 60 50 40 30 20 10 0
f1 (ppm)

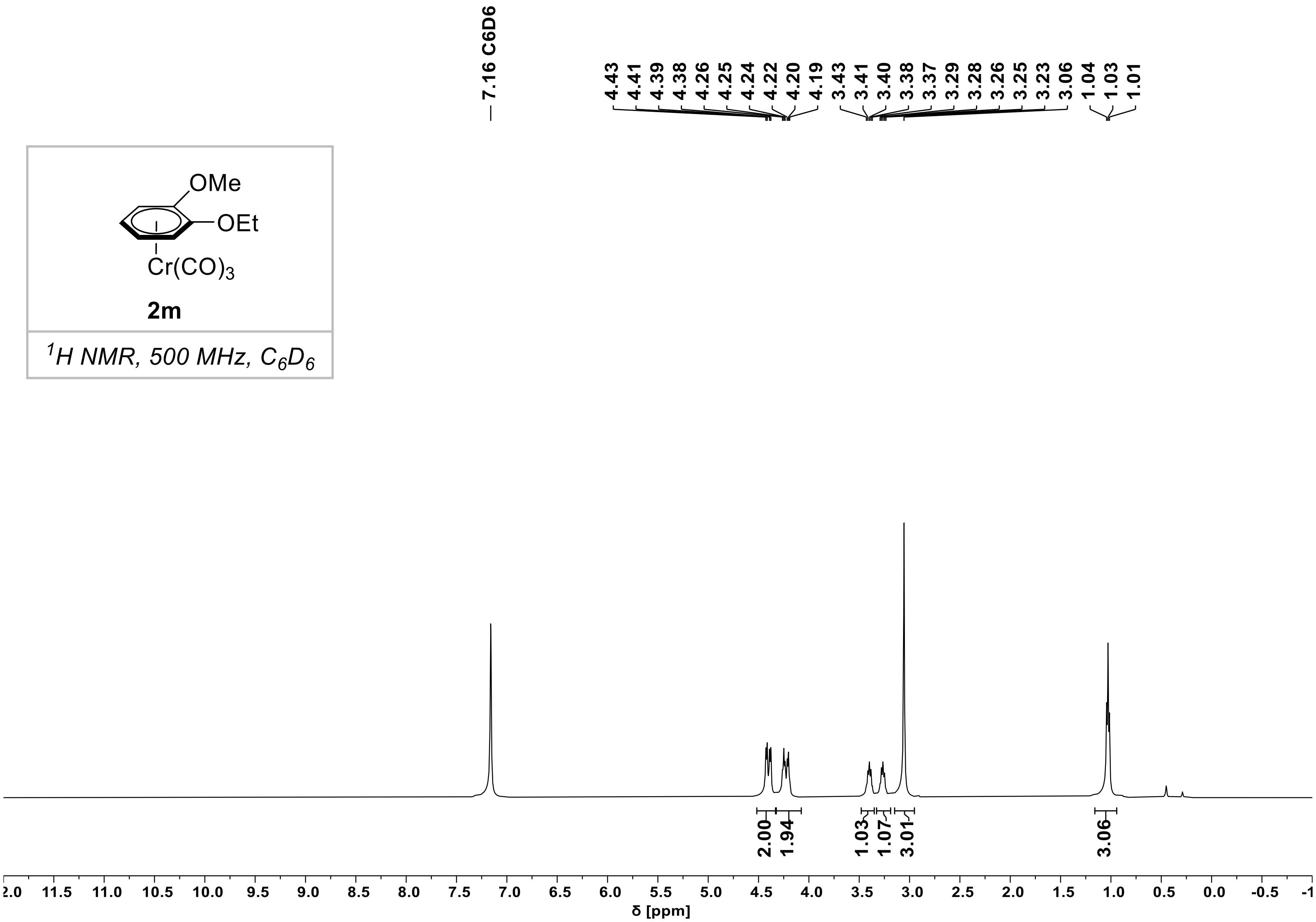
OMe
OEt
Cr(CO)3
2m
1H NMR, 500 MHz, C6D6
7.16 C6D6
4.43
4.41
4.39
4.38
4.26
4.25
4.24
4.22
4.20
4.19
3.43
3.41
3.40
3.38
3.37
3.29
3.28
3.26
3.25
3.23
3.06
1.04
1.03
1.01
2.00
1.94
1.03
1.07
3.01
3.06
δ [ppm]

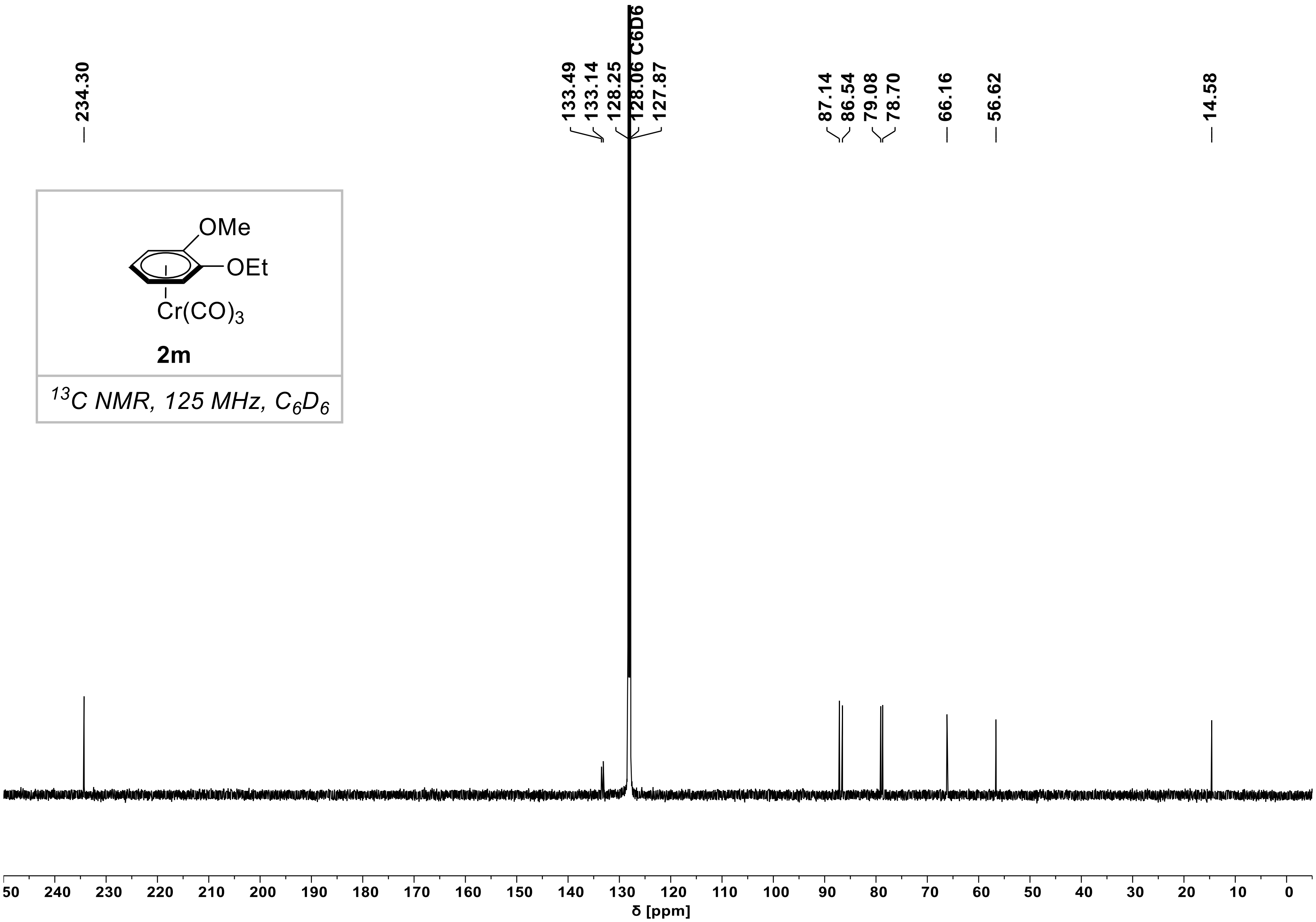
234.30
133.49
133.14
128.25
128.06 C6D6
127.87
87.14
86.54
79.08
78.70
66.16
56.62
14.58
OMe
OEt
Cr(CO)3
2m
13C NMR, 125 MHz, C6D6
50
240
230
220
210
200
190
180
170
160
150
140
130
120
110
100
90
80
70
60
50
40
30
20
10
0
δ [ppm]

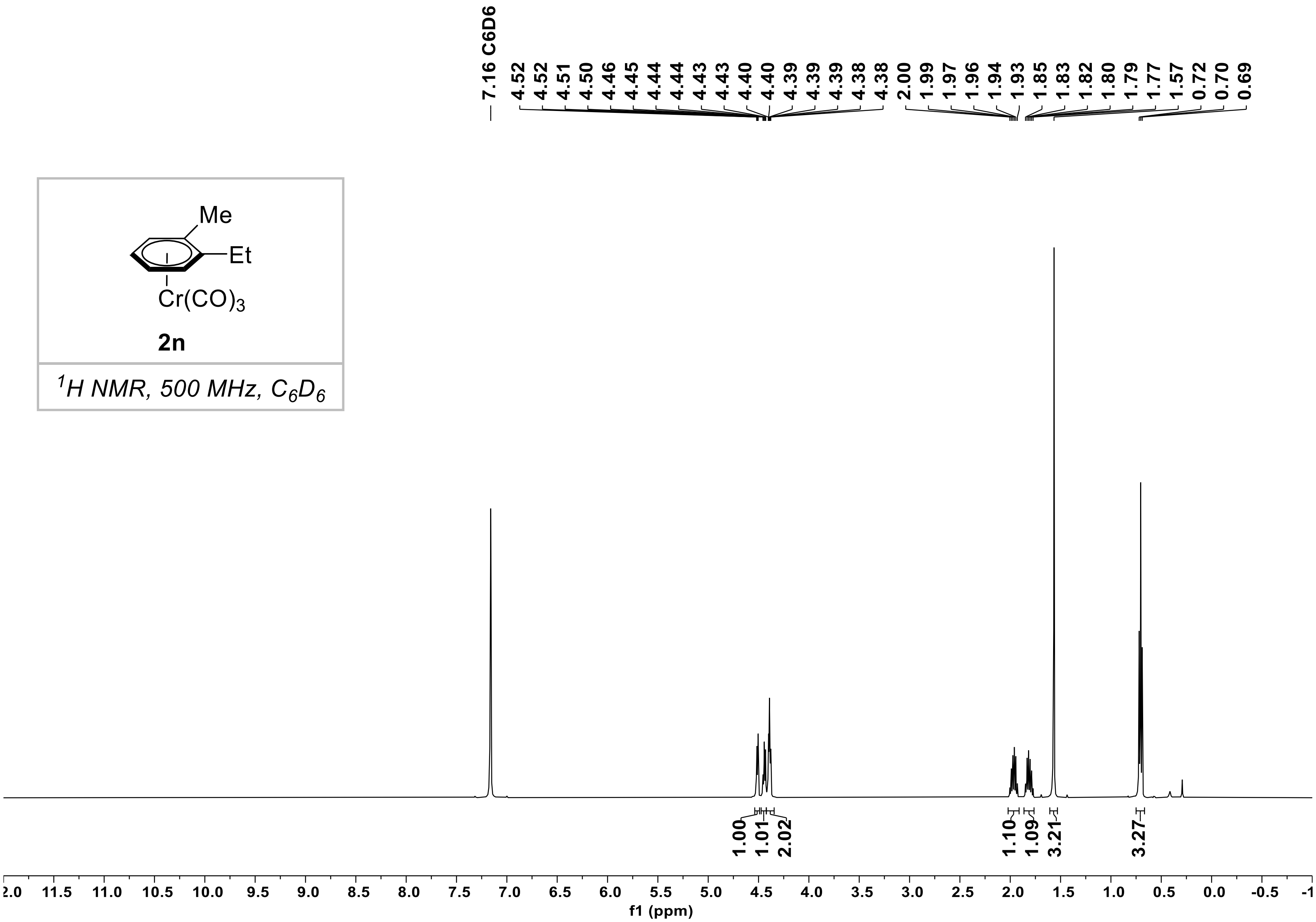
7.16 C6D6
4.52
4.52
4.51
4.50
4.46
4.45
4.44
4.44
4.43
4.43
4.40
4.40
4.39
4.39
4.39
4.38
4.38
2.00
1.99
1.97
1.96
1.94
1.93
1.85
1.83
1.82
1.80
1.79
1.77
1.57
0.72
0.70
0.69
Me
Et
Cr(CO)3
2n
1H NMR, 500 MHz, C6D6
1.00
1.01
2.02
1.10
1.09
3.21
3.27
f1 (ppm)

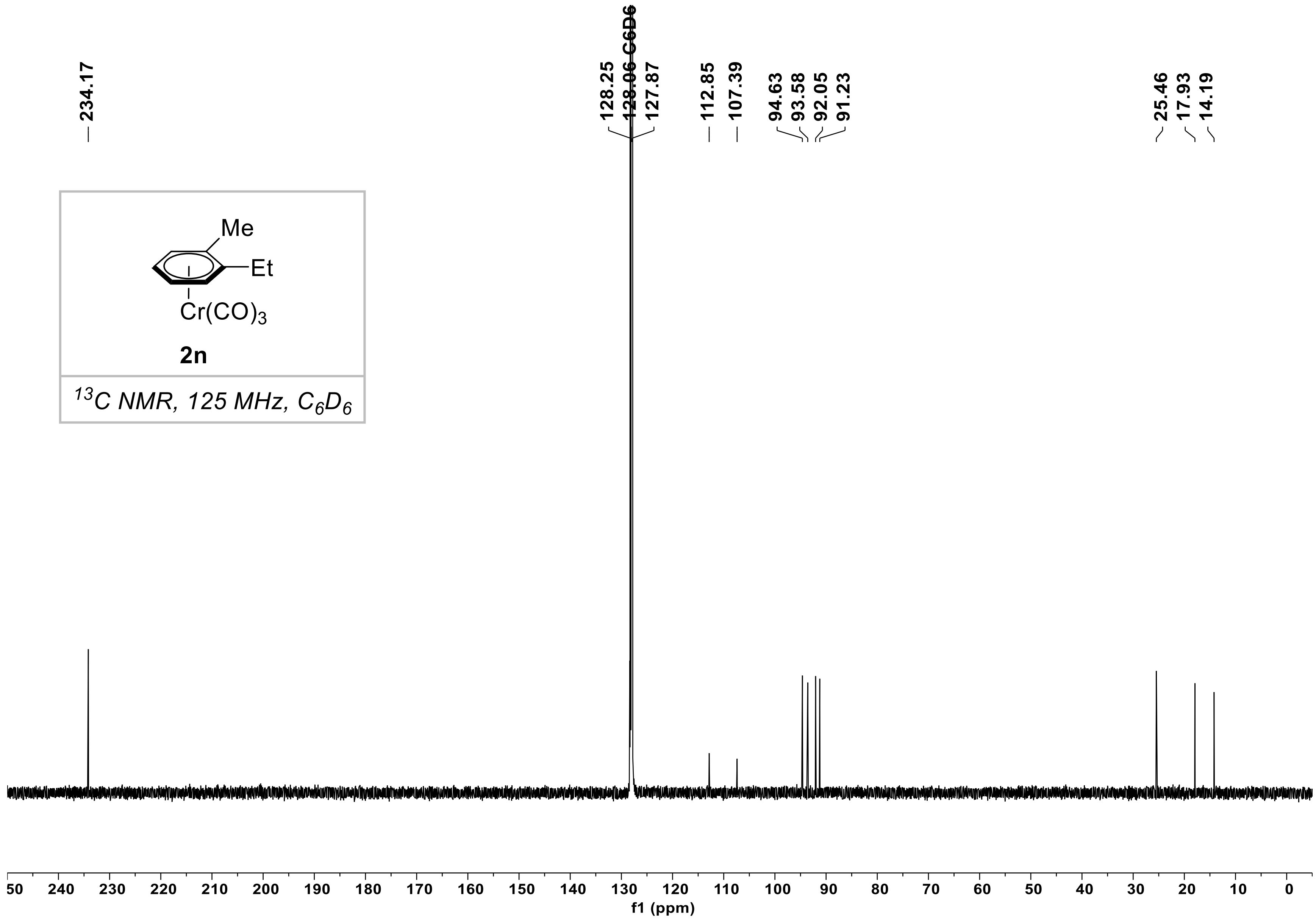
234.17
128.25
128.06 C6D6
127.87
112.85
107.39
94.63
93.58
92.05
91.23
25.46
17.93
14.19
Me
Et
Cr(CO)3
2n
13C NMR, 125 MHz, C6D6
50
240
230
220
210
200
190
180
170
160
150
140
130
120
110
100
90
80
70
60
50
40
30
20
10
0
f1 (ppm)

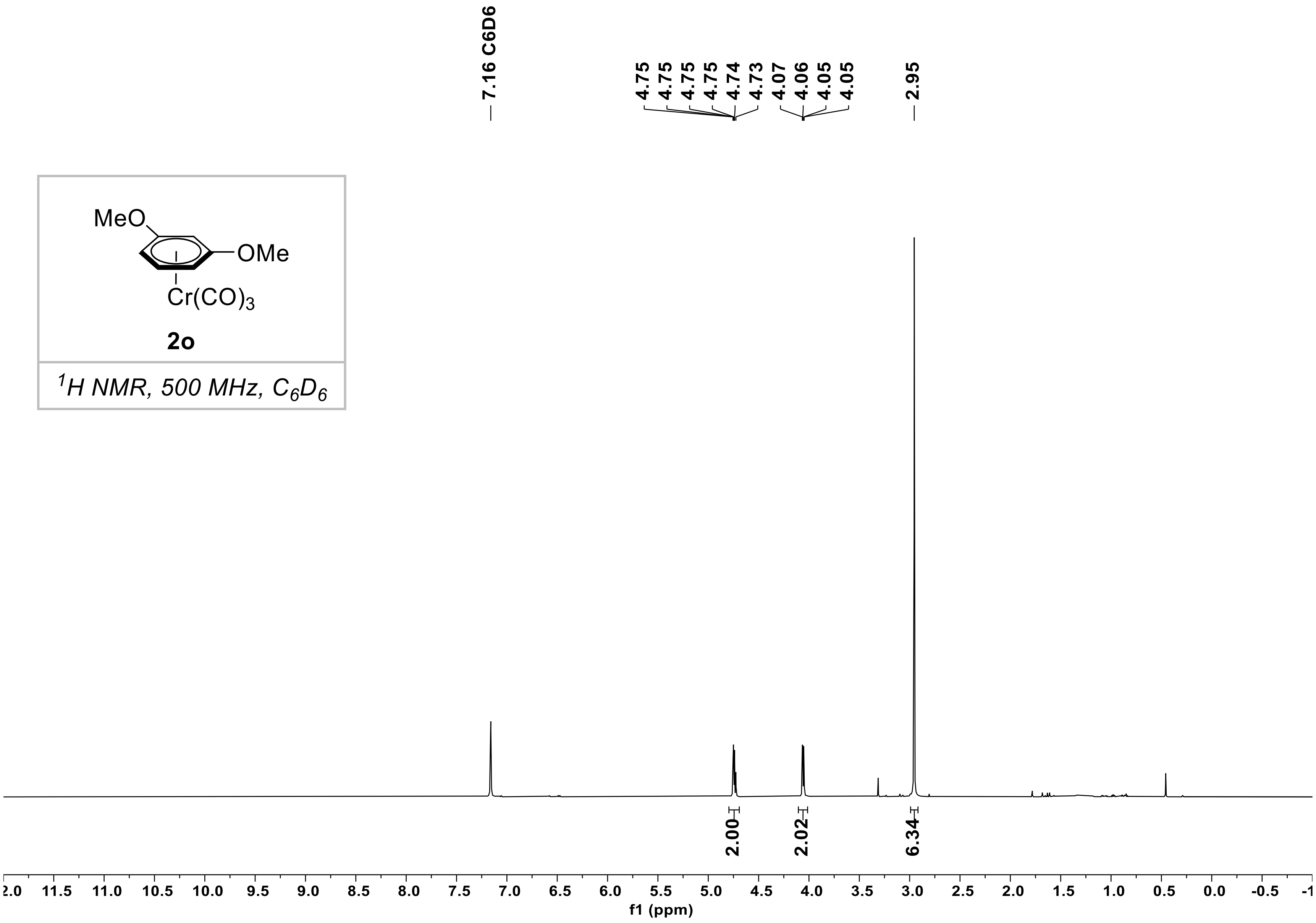
7.16 C6D6
4.75
4.75
4.75
4.75
4.74
4.73
4.07
4.06
4.05
4.05
2.95
MeO
OMe
Cr(CO)3
2o
1H NMR, 500 MHz, C6D6
2.00
2.02
6.34
f1 (ppm)

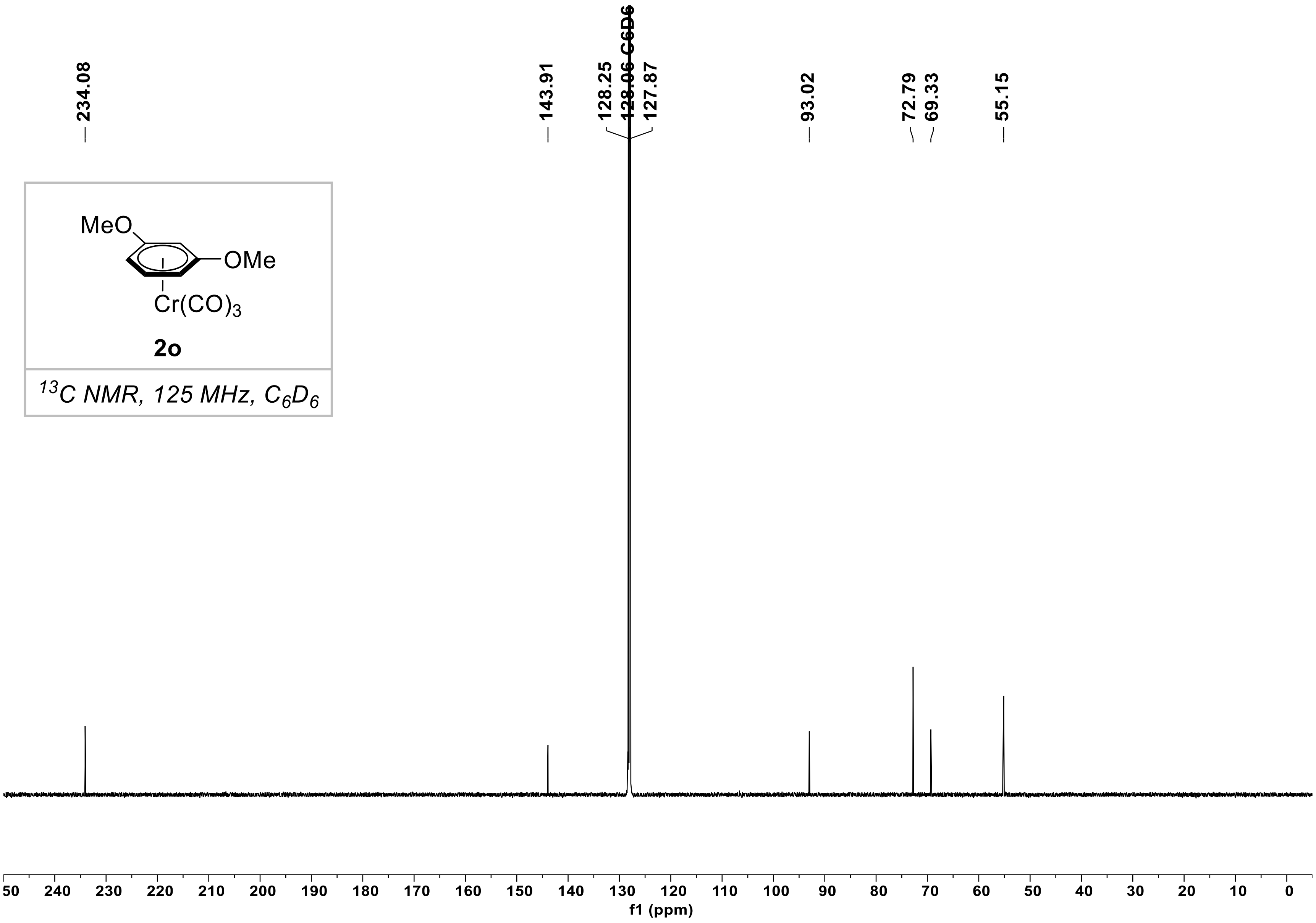
234.08
143.91
128.25
128.06 C6D6
127.87
93.02
72.79
69.33
55.15
MeO
OMe
Cr(CO)3
2o
13C NMR, 125 MHz, C6D6
f1 (ppm)

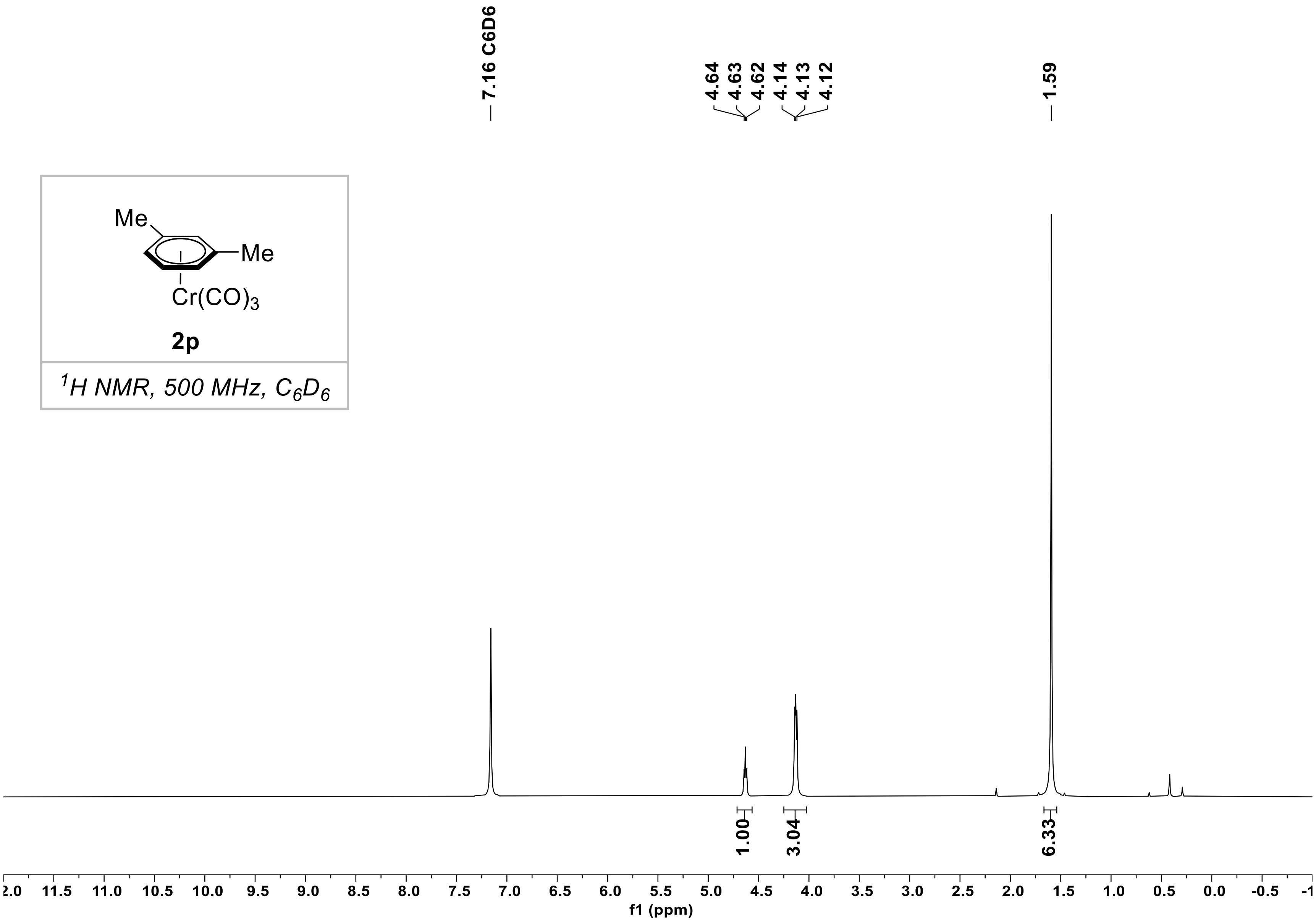
7.16 C6D6
4.64
4.63
4.62
4.14
4.13
4.12
1.59
Me
Me
Cr(CO)3
2p
1H NMR, 500 MHz, C6D6
1.00
3.04
6.33
2.0 11.5 11.0 10.5 10.0 9.5 9.0 8.5 8.0 7.5 7.0 6.5 6.0 5.5 5.0 4.5 4.0 3.5 3.0 2.5 2.0 1.5 1.0 0.5 0.0 -0.5 -1
f1 (ppm)

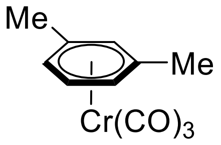

2p

^{13}C NMR, 125 MHz, C_6D_6

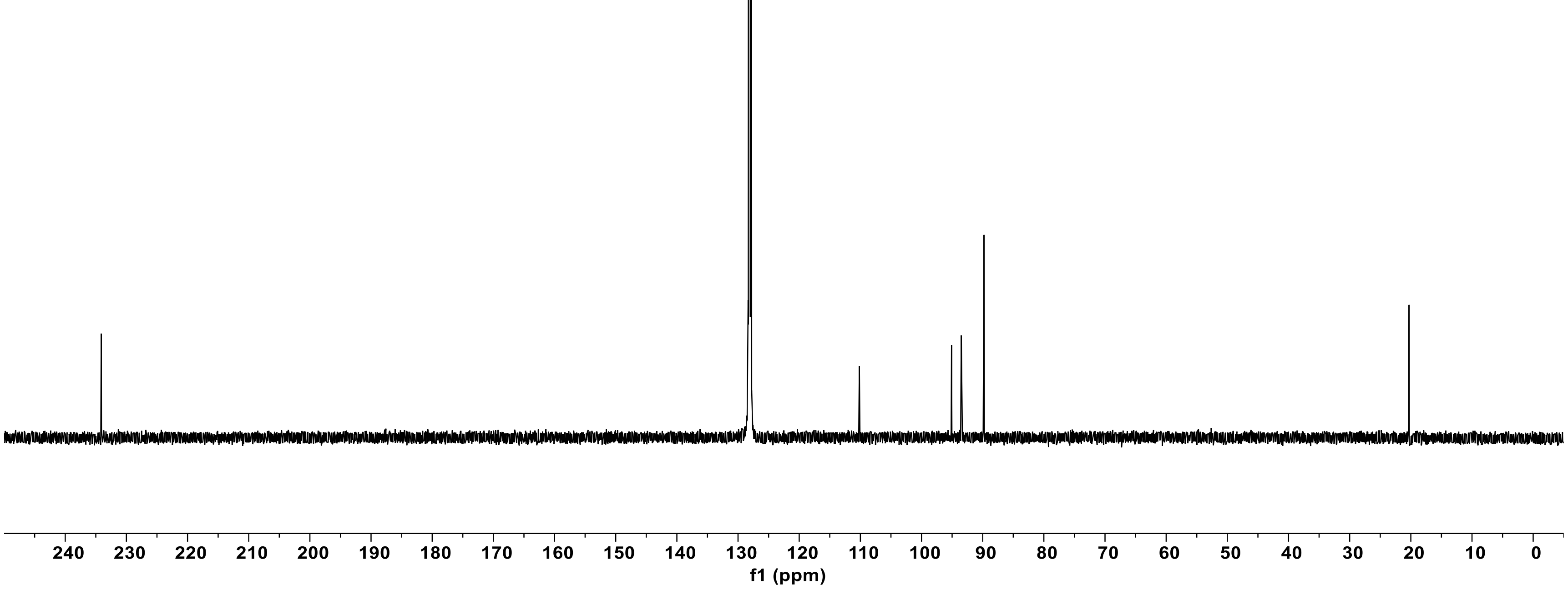

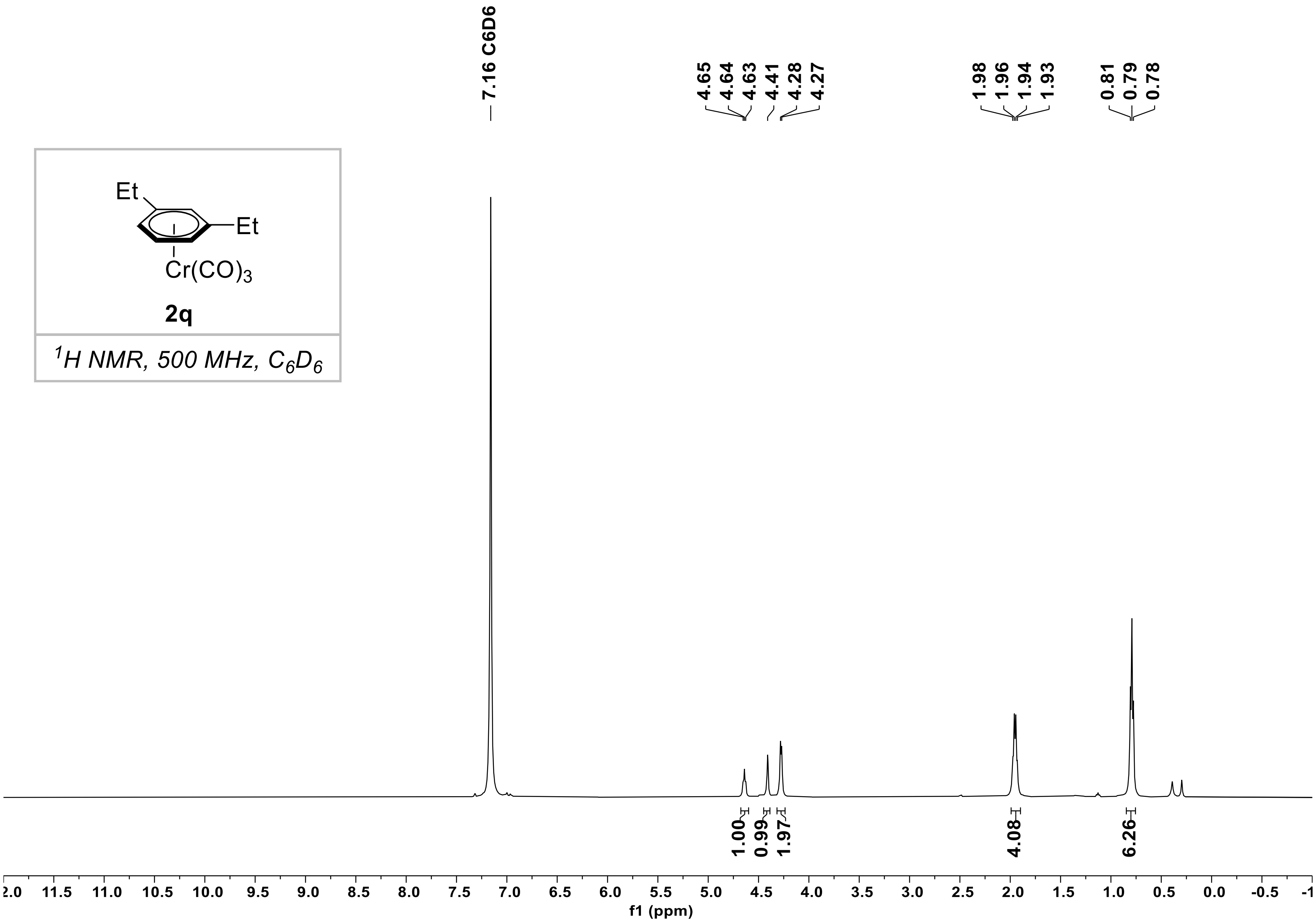
7.16 C6D6
4.65
4.64
4.63
4.41
4.28
4.27
1.98
1.96
1.94
1.93
0.81
0.79
0.78
Et
Et
Cr(CO)3
2q
1H NMR, 500 MHz, C6D6
1.00
0.99
1.97
4.08
6.26
f1 (ppm)

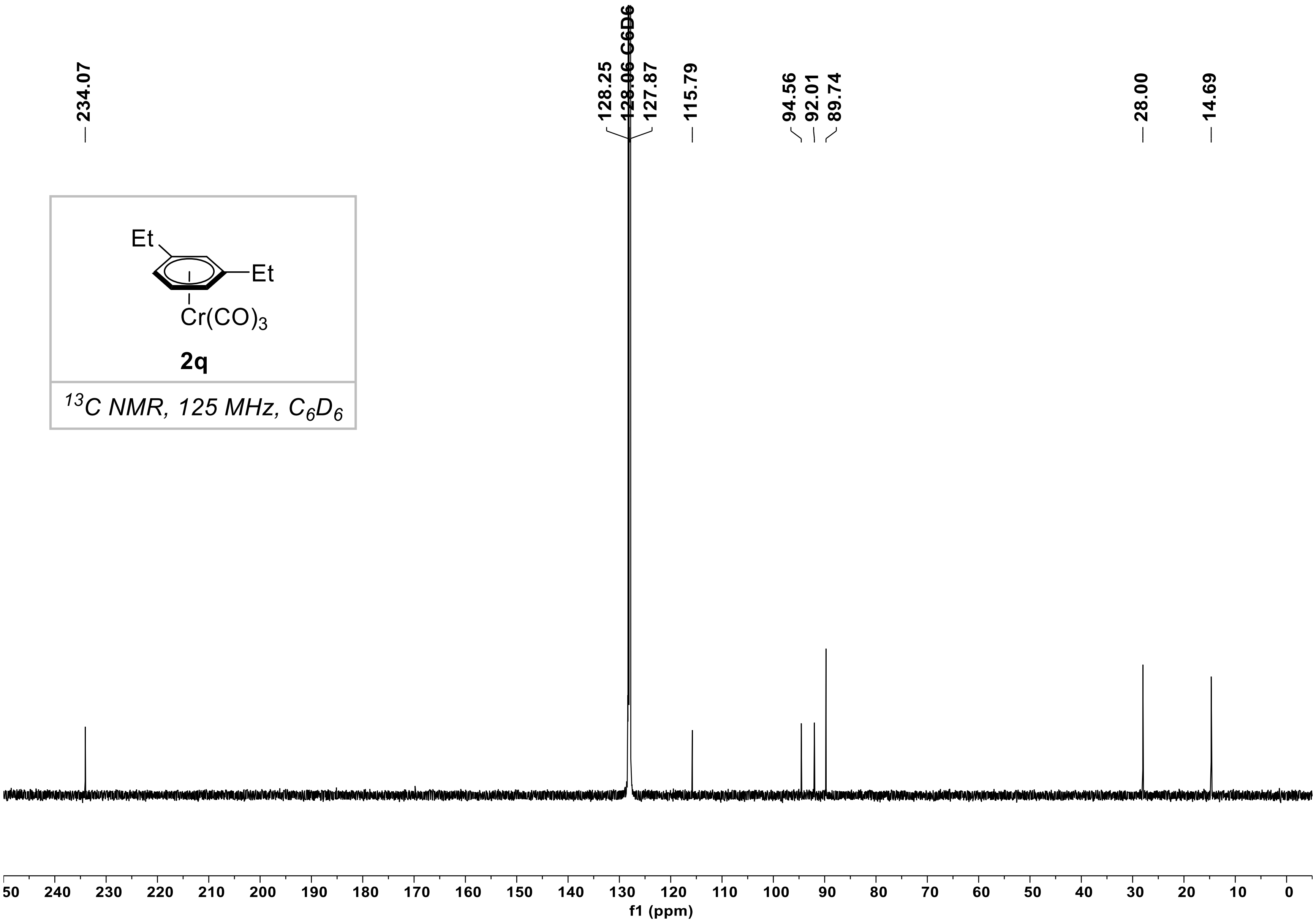

234.07
128.25
128.06 C6D6
127.87
115.79
94.56
92.01
89.74
28.00
14.69
Et
Et
Cr(CO)3
2q
13C NMR, 125 MHz, C6D6
50
240
230
220
210
200
190
180
170
160
150
140
130
120
110
100
90
80
70
60
50
40
30
20
10
0
f1 (ppm)

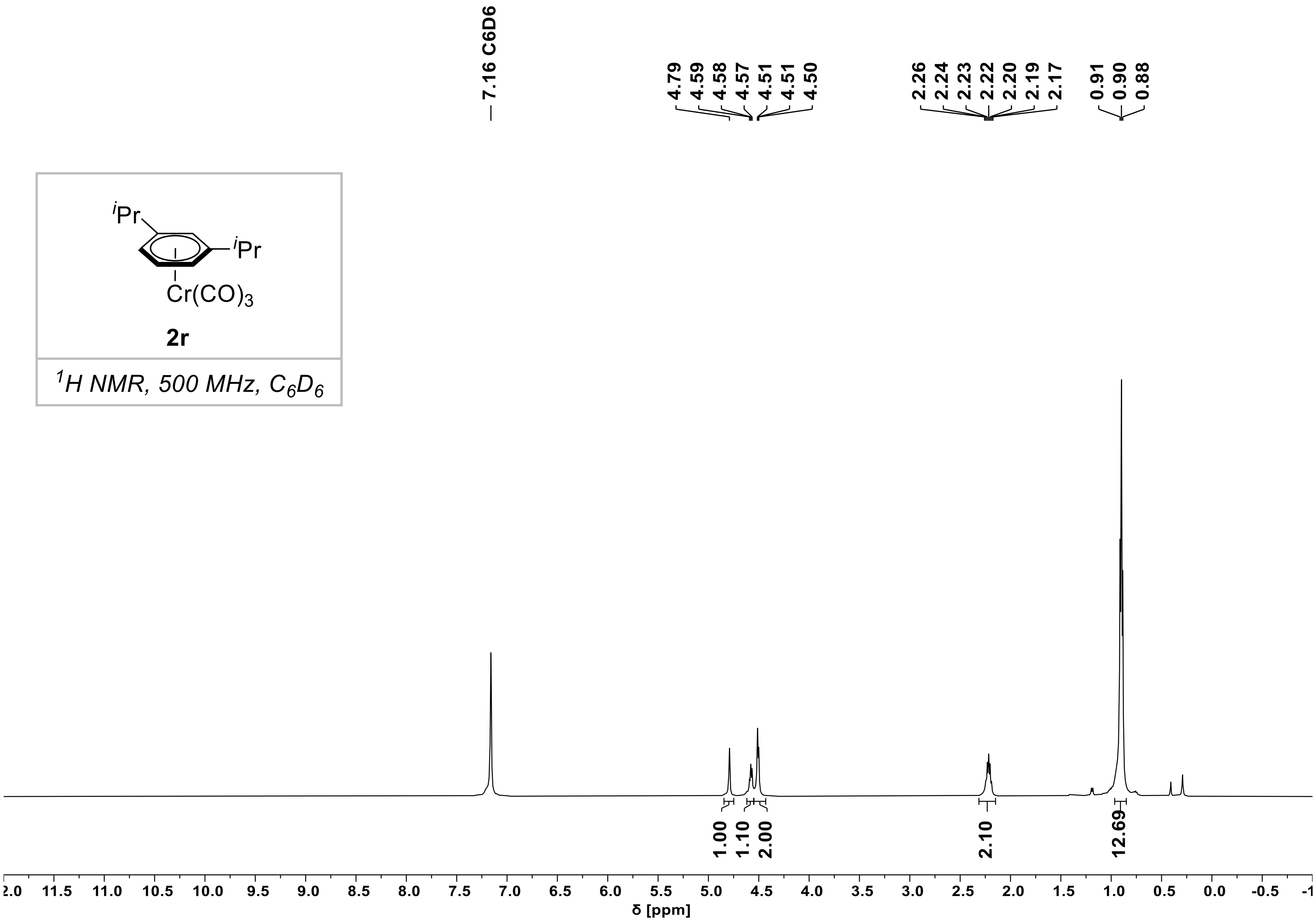

iPr
iPr
Cr(CO)3
2r
1H NMR, 500 MHz, C6D6
7.16 C6D6
4.79
4.59
4.58
4.57
4.51
4.51
4.50
2.26
2.24
2.23
2.22
2.20
2.19
2.17
0.91
0.90
0.88
1.00
1.10
2.00
2.10
12.69
δ [ppm]

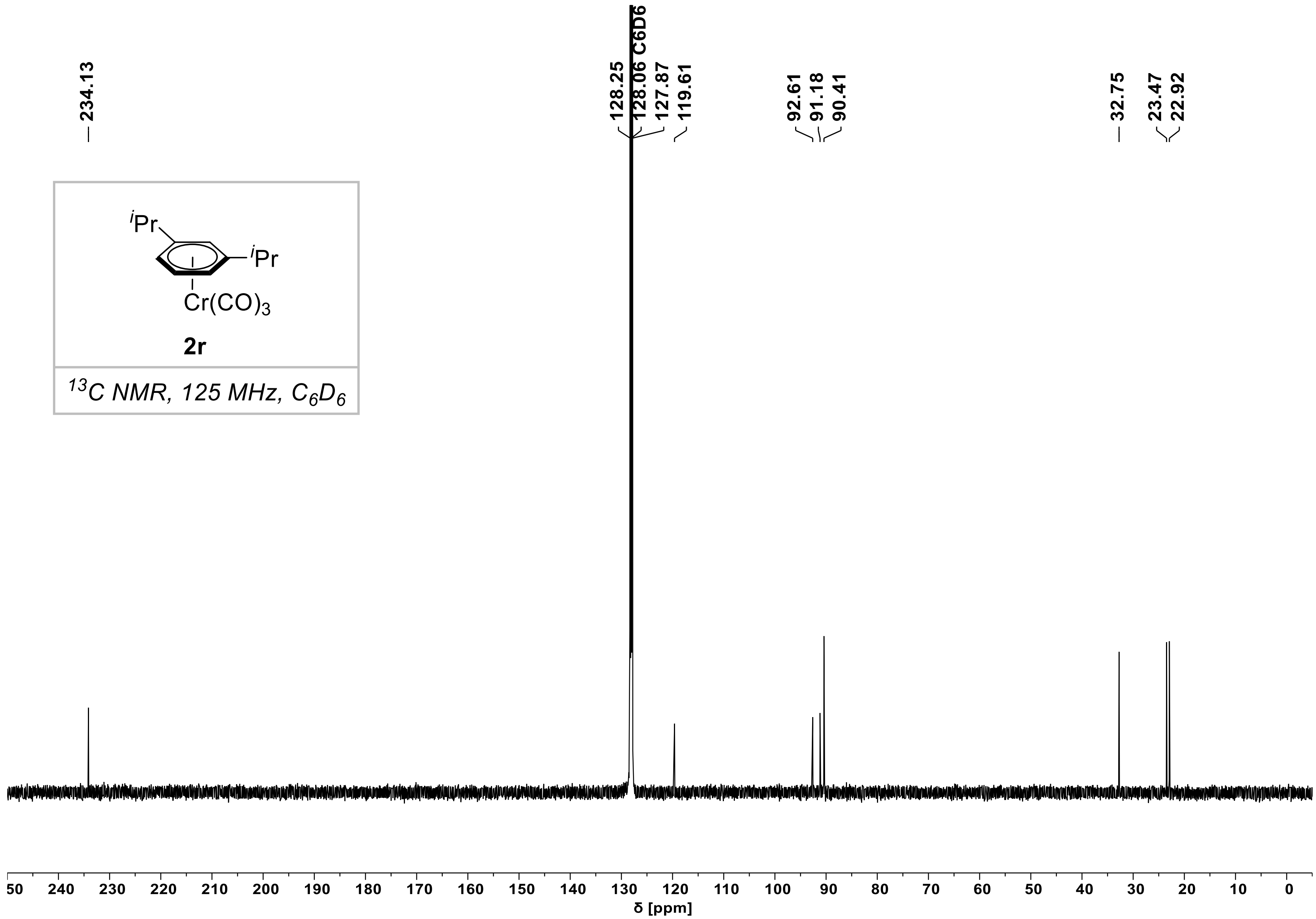
234.13
128.25
128.06 C6D6
127.87
119.61
92.61
91.18
90.41
32.75
23.47
22.92
iPr
iPr
Cr(CO)3
2r
13C NMR, 125 MHz, C6D6
50
240
230
220
210
200
190
180
170
160
150
140
130
120
110
100
90
80
70
60
50
40
30
20
10
0
δ [ppm]

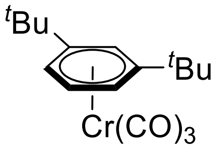

2s

^{1}H NMR, 500 MHz, C_6D_6

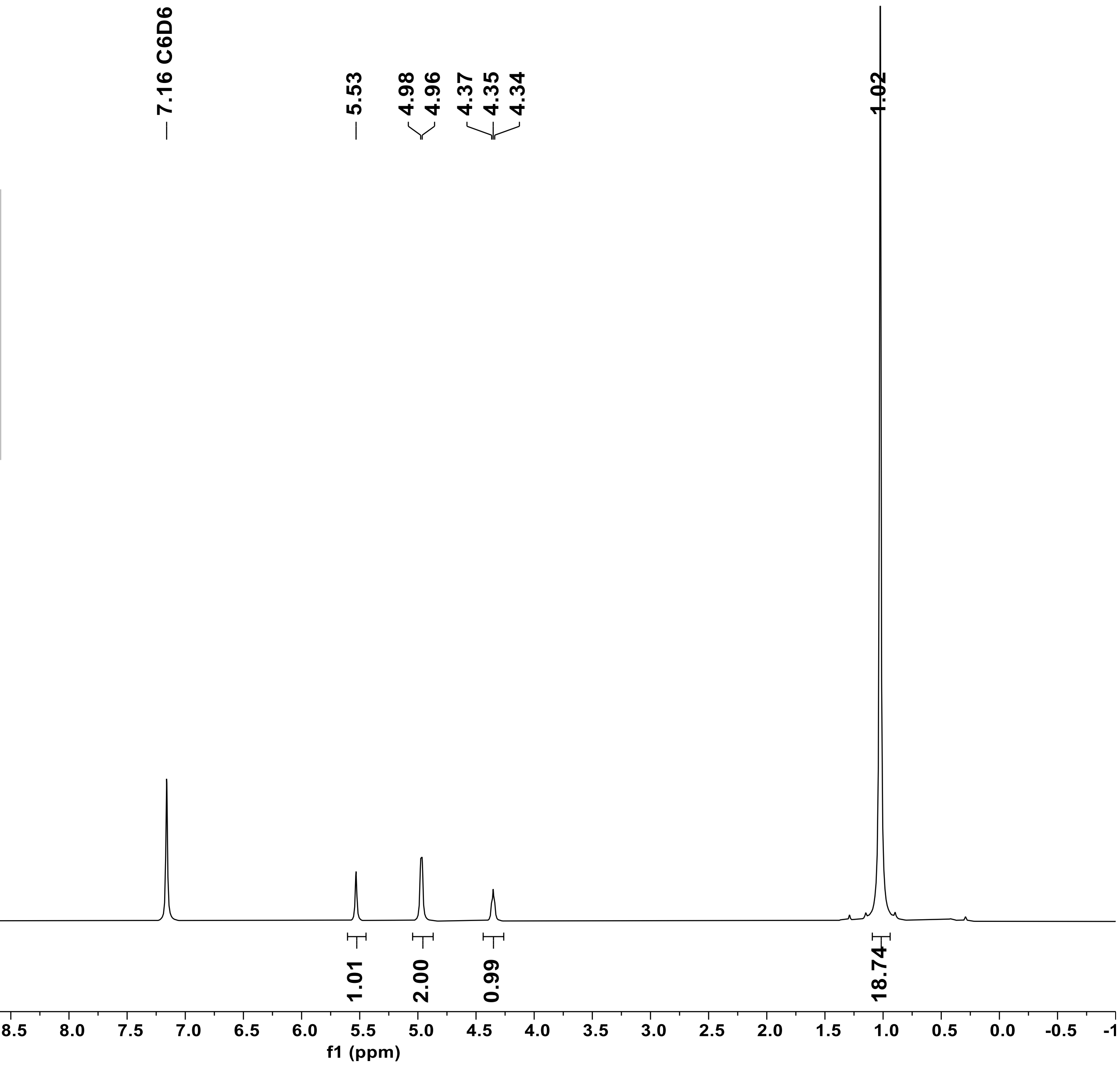

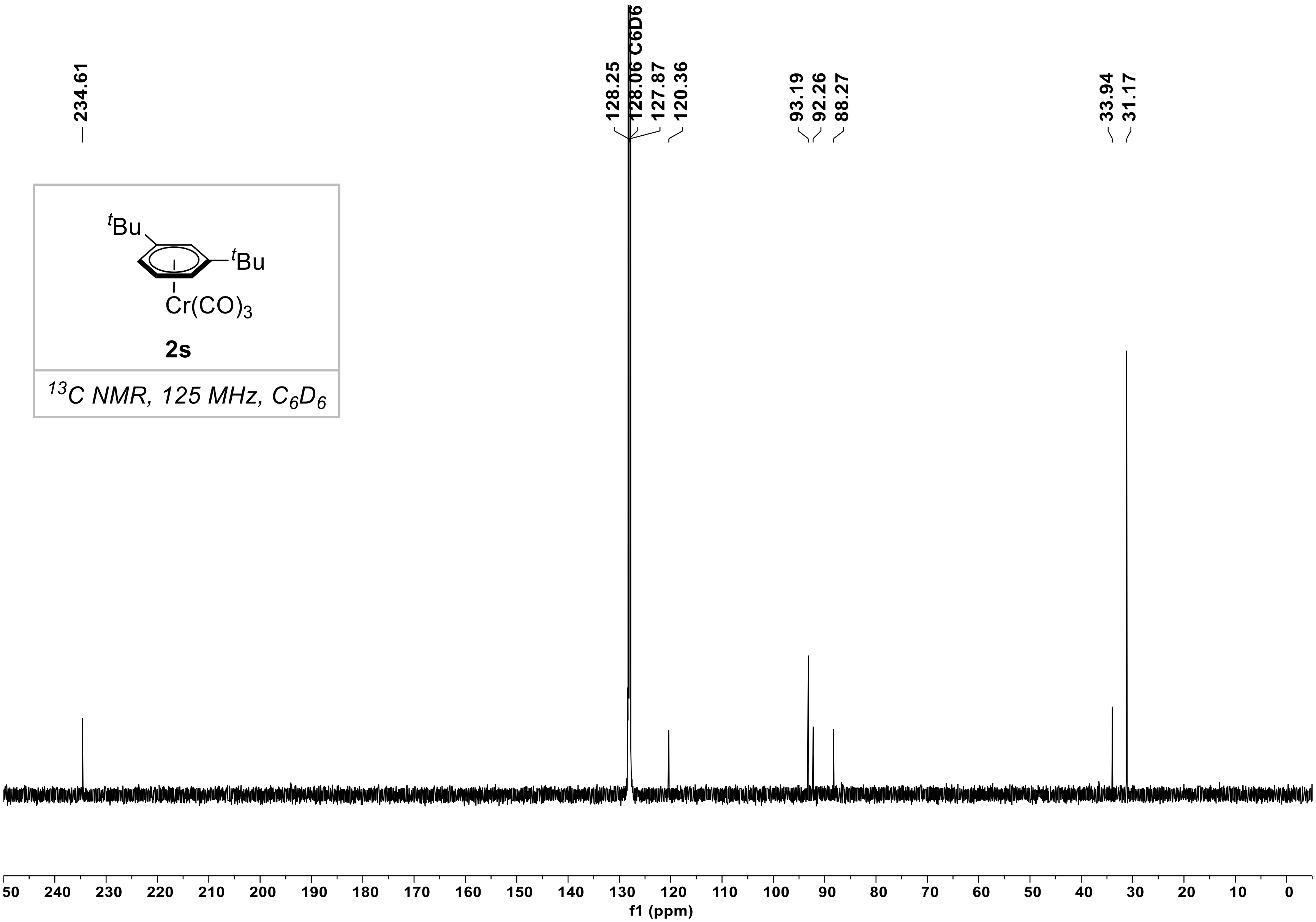
234.61
128.25
128.06 C6D6
127.87
120.36
93.19
92.26
88.27
33.94
31.17
^{t}Bu
^{t}Bu
$Cr(CO)_3$
2s
^{13}C NMR, 125 MHz, C_6D_6
50 240 230 220 210 200 190 180 170 160 150 140 130 120 110 100 90 80 70 60 50 40 30 20 10 0
f1 (ppm)

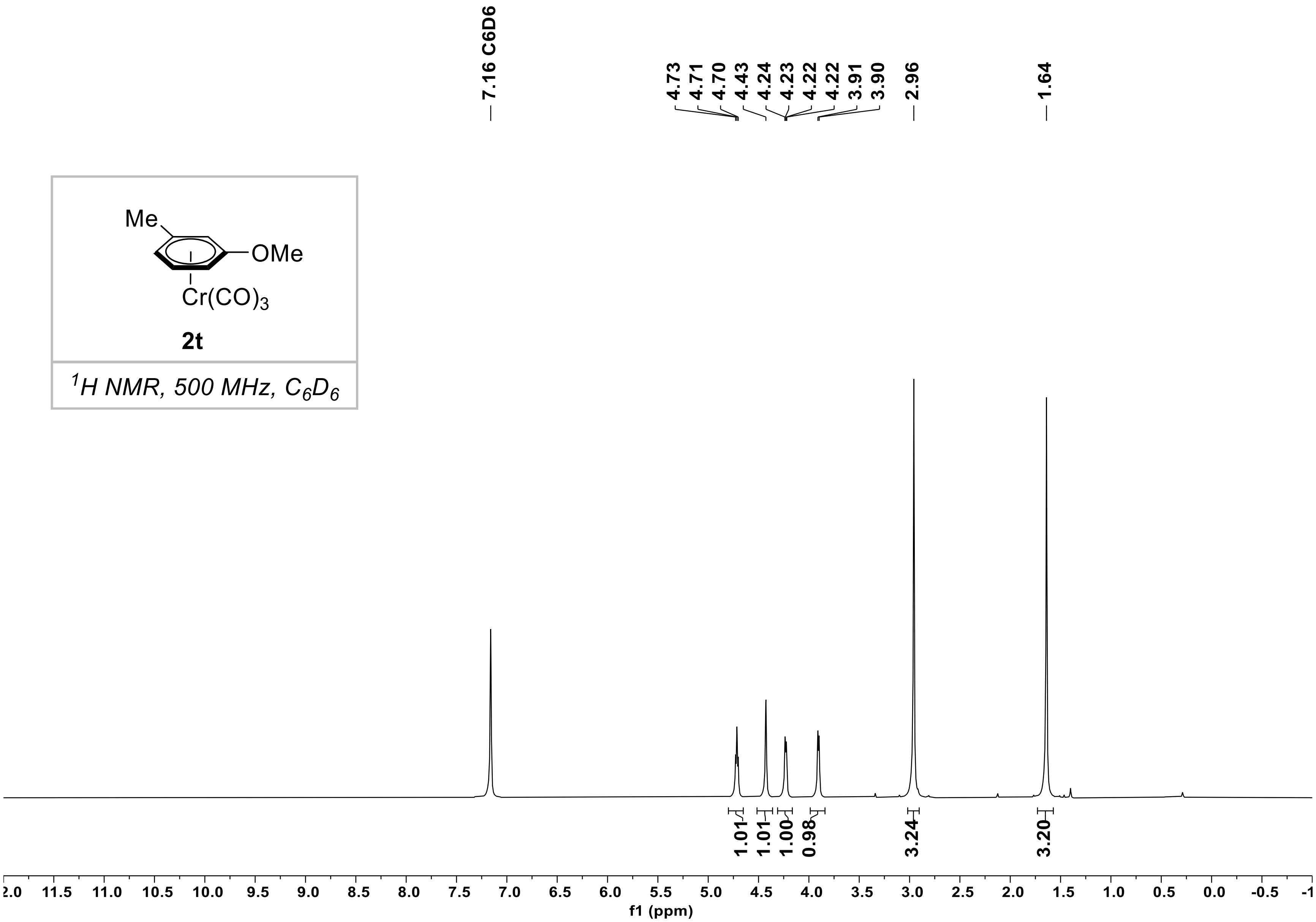
7.16 C6D6
4.73
4.71
4.70
4.43
4.24
4.23
4.22
4.22
3.91
3.90
2.96
1.64
Me
OMe
Cr(CO)3
2t
1H NMR, 500 MHz, C6D6
1.01
1.01
1.00
0.98
3.24
3.20
f1 (ppm)

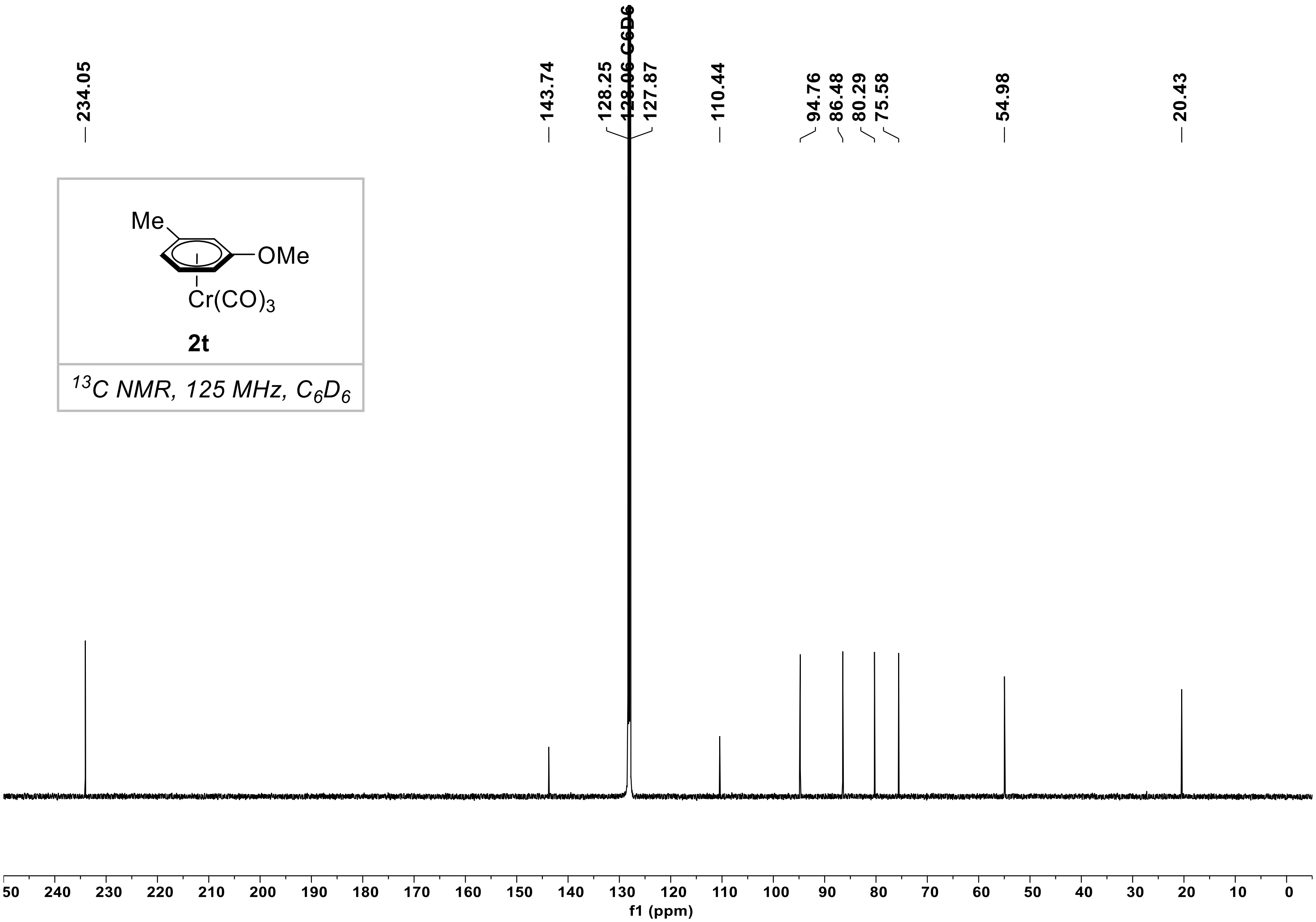

^{13}C NMR, 125 MHz, C_6D_6

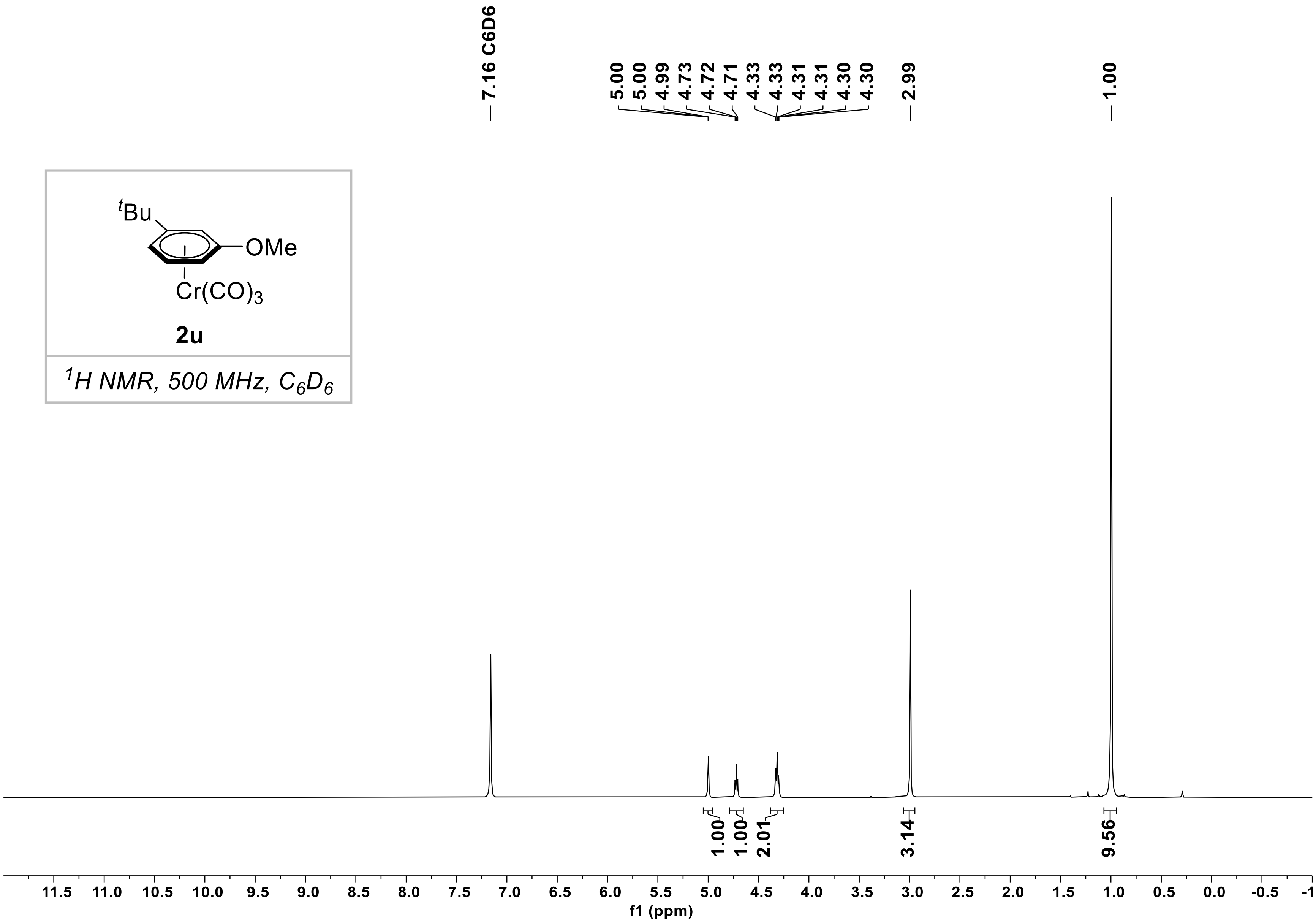
tBu
OMe
Cr(CO)3
2u
1H NMR, 500 MHz, C6D6
7.16 C6D6
5.00
5.00
4.99
4.73
4.72
4.71
4.33
4.33
4.31
4.31
4.30
4.30
2.99
1.00
1.00
1.00
2.01
3.14
9.56
f1 (ppm)

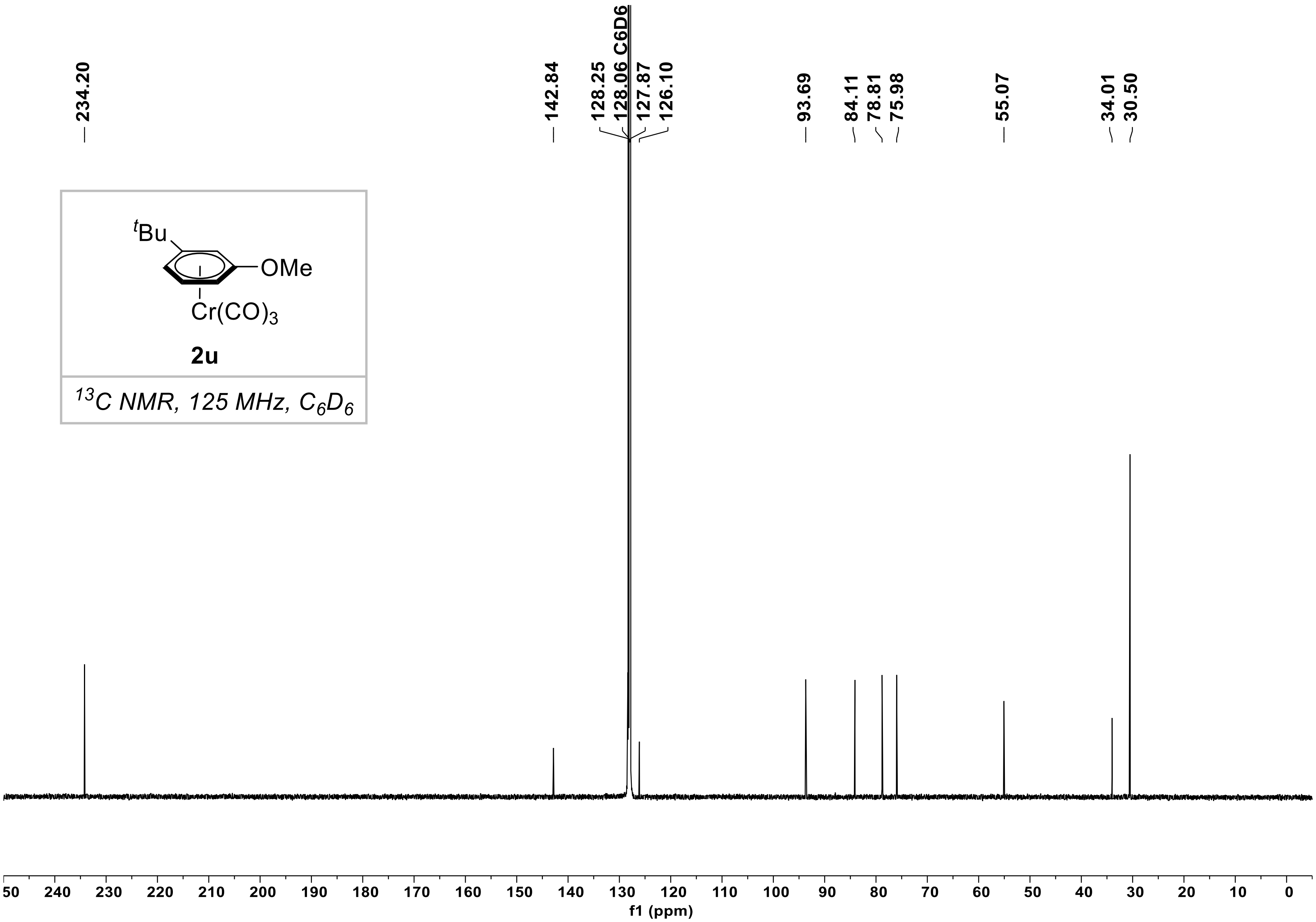
234.20
142.84
128.25
128.06 C6D6
127.87
126.10
93.69
84.11
78.81
75.98
55.07
34.01
30.50
tBu
OMe
Cr(CO)3
2u
13C NMR, 125 MHz, C6D6
50
240
230
220
210
200
190
180
170
160
150
140
130
120
110
100
90
80
70
60
50
40
30
20
10
0
f1 (ppm)

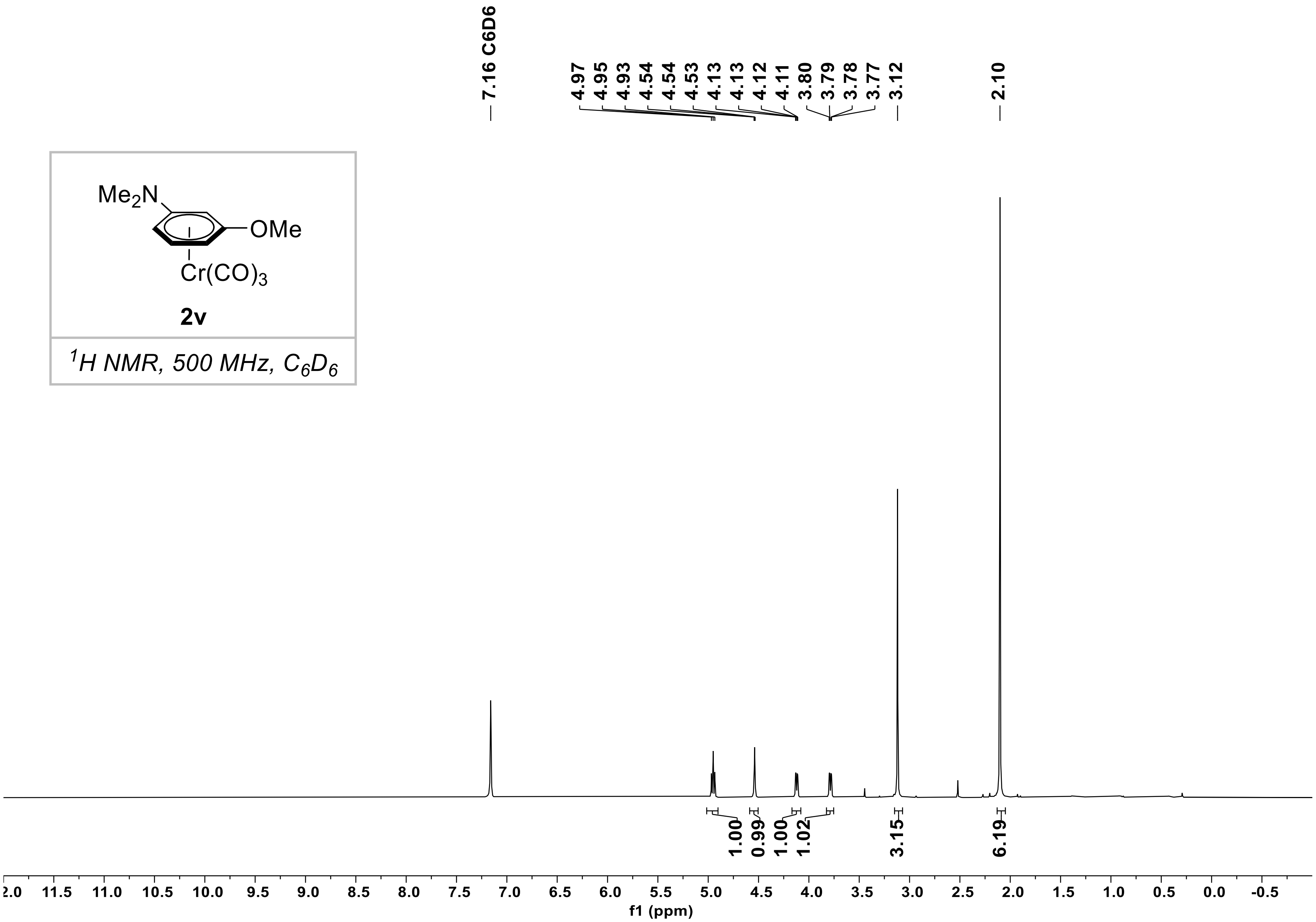
7.16 C6D6
4.97
4.95
4.93
4.54
4.54
4.53
4.13
4.13
4.12
4.11
3.80
3.79
3.78
3.77
3.12
2.10
Me2N
OMe
Cr(CO)3
2v
1H NMR, 500 MHz, C6D6
1.00
0.99
1.00
1.02
3.15
6.19
f1 (ppm)

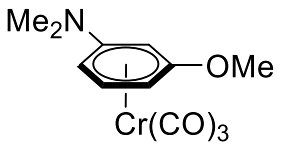

2v

^{13}C NMR, 125 MHz, C_6D_6

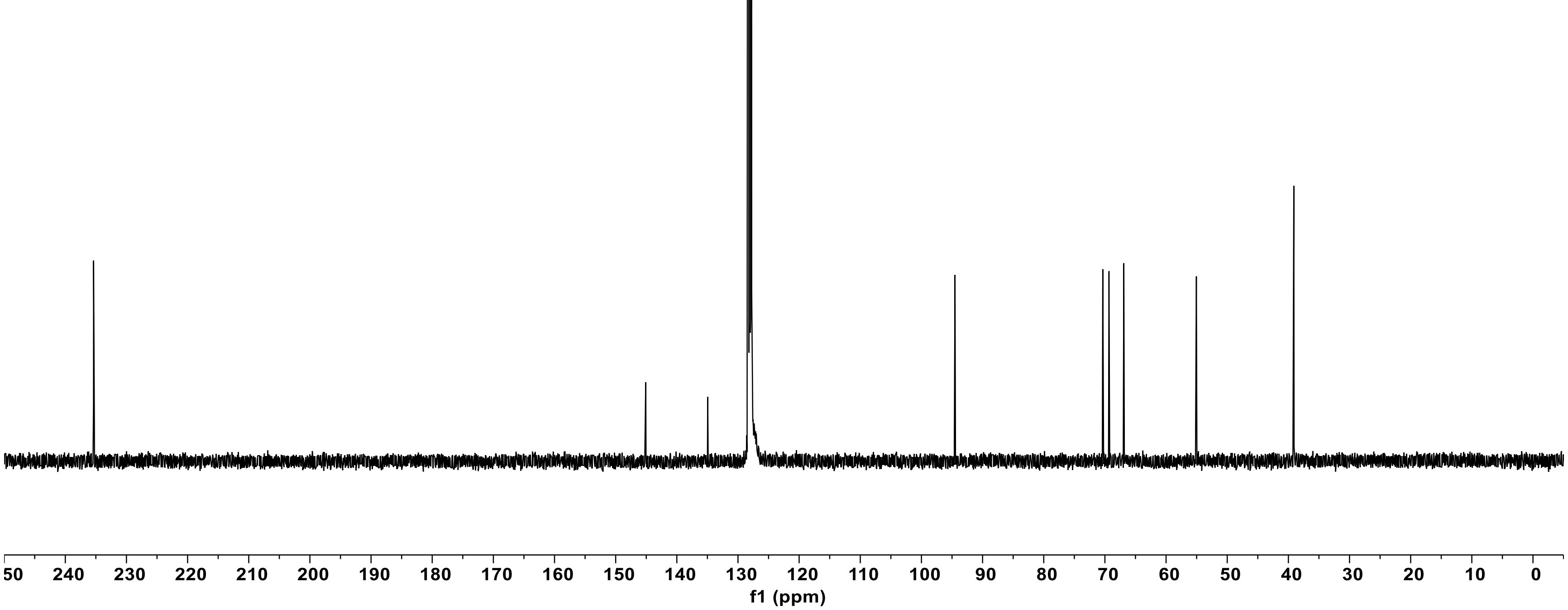

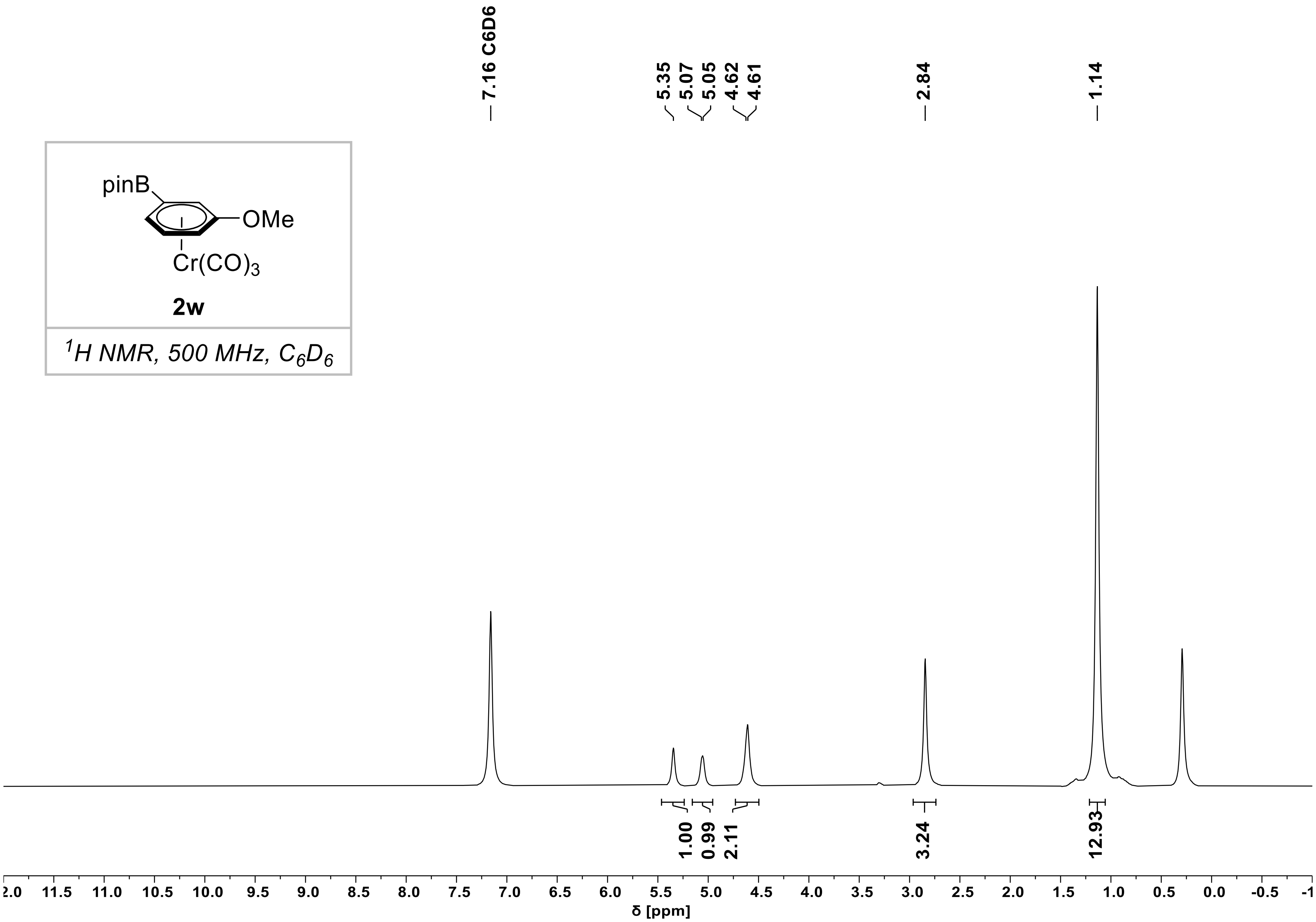
pinB
OMe
Cr(CO)3
2w
1H NMR, 500 MHz, C6D6
7.16 C6D6
5.35
5.07
5.05
4.62
4.61
2.84
1.14
1.00
0.99
2.11
3.24
12.93
12.0 11.5 11.0 10.5 10.0 9.5 9.0 8.5 8.0 7.5 7.0 6.5 6.0 5.5 5.0 4.5 4.0 3.5 3.0 2.5 2.0 1.5 1.0 0.5 0.0 -0.5 -1
δ [ppm]

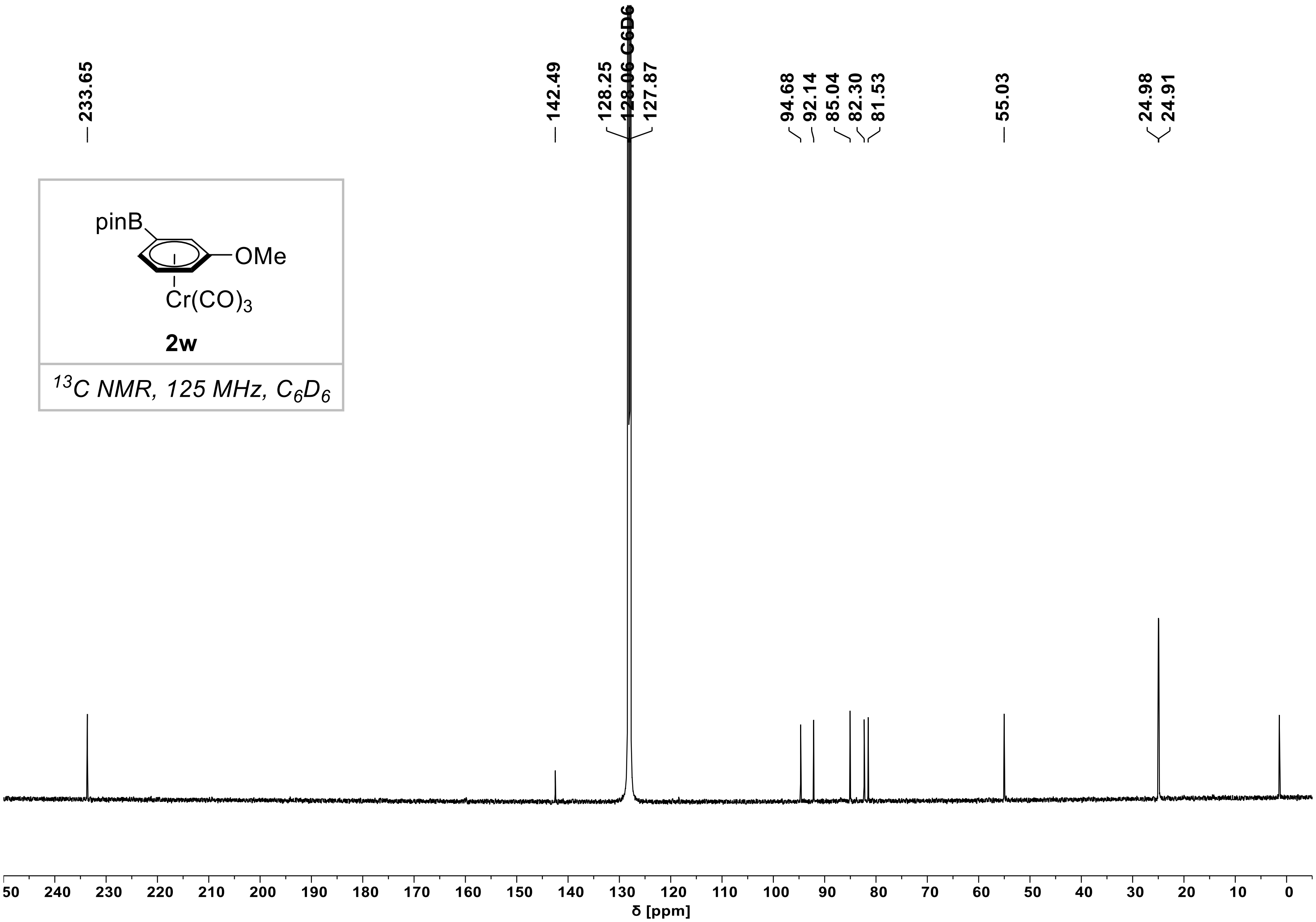
233.65
142.49
128.25
128.06 C6D6
127.87
94.68
92.14
85.04
82.30
81.53
55.03
24.98
24.91
pinB
OMe
Cr(CO)3
2w
13C NMR, 125 MHz, C6D6
50
240
230
220
210
200
190
180
170
160
150
140
130
120
110
100
90
80
70
60
50
40
30
20
10
0
δ [ppm]

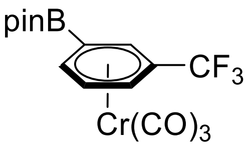

2x

^1H NMR, 500 MHz, C_6D_6

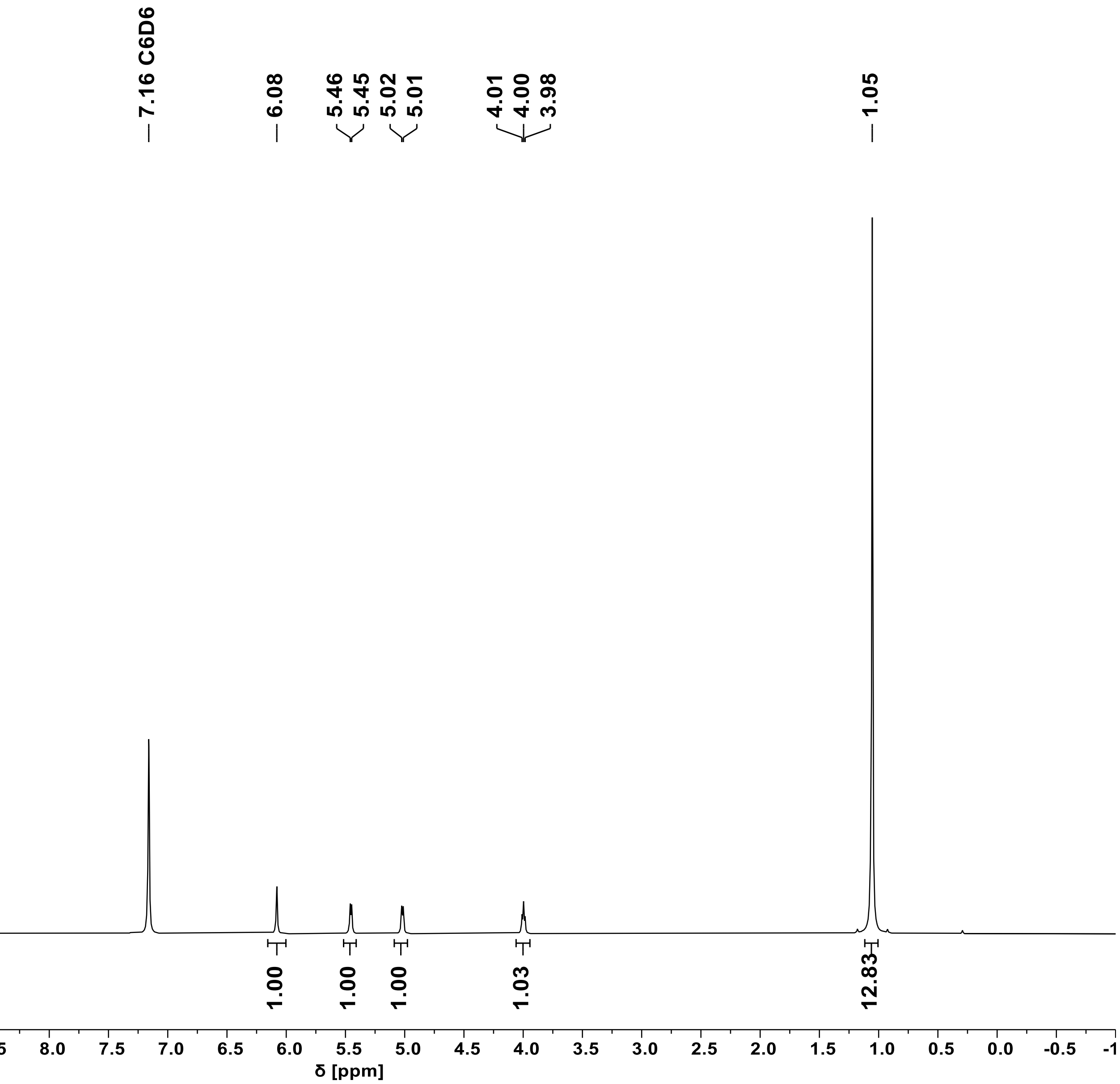

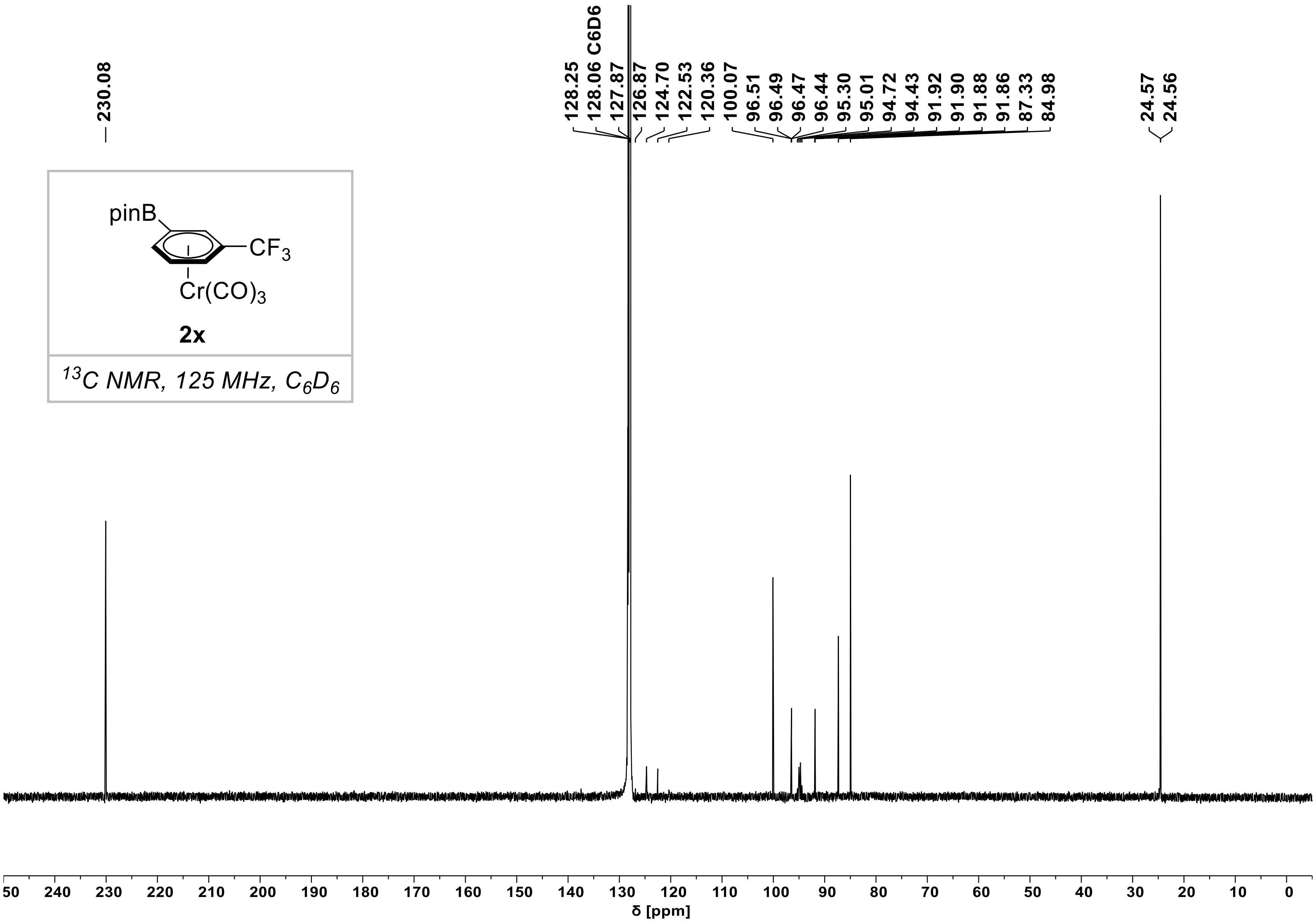
230.08
128.25
128.06 C6D6
127.87
126.87
124.70
122.53
120.36
100.07
96.51
96.49
96.47
96.44
95.30
95.01
94.72
94.43
91.92
91.90
91.88
91.86
87.33
84.98
24.57
24.56
pinB
CF3
Cr(CO)3
2x
13C NMR, 125 MHz, C6D6
50 240 230 220 210 200 190 180 170 160 150 140 130 120 110 100 90 80 70 60 50 40 30 20 10 0
δ [ppm]

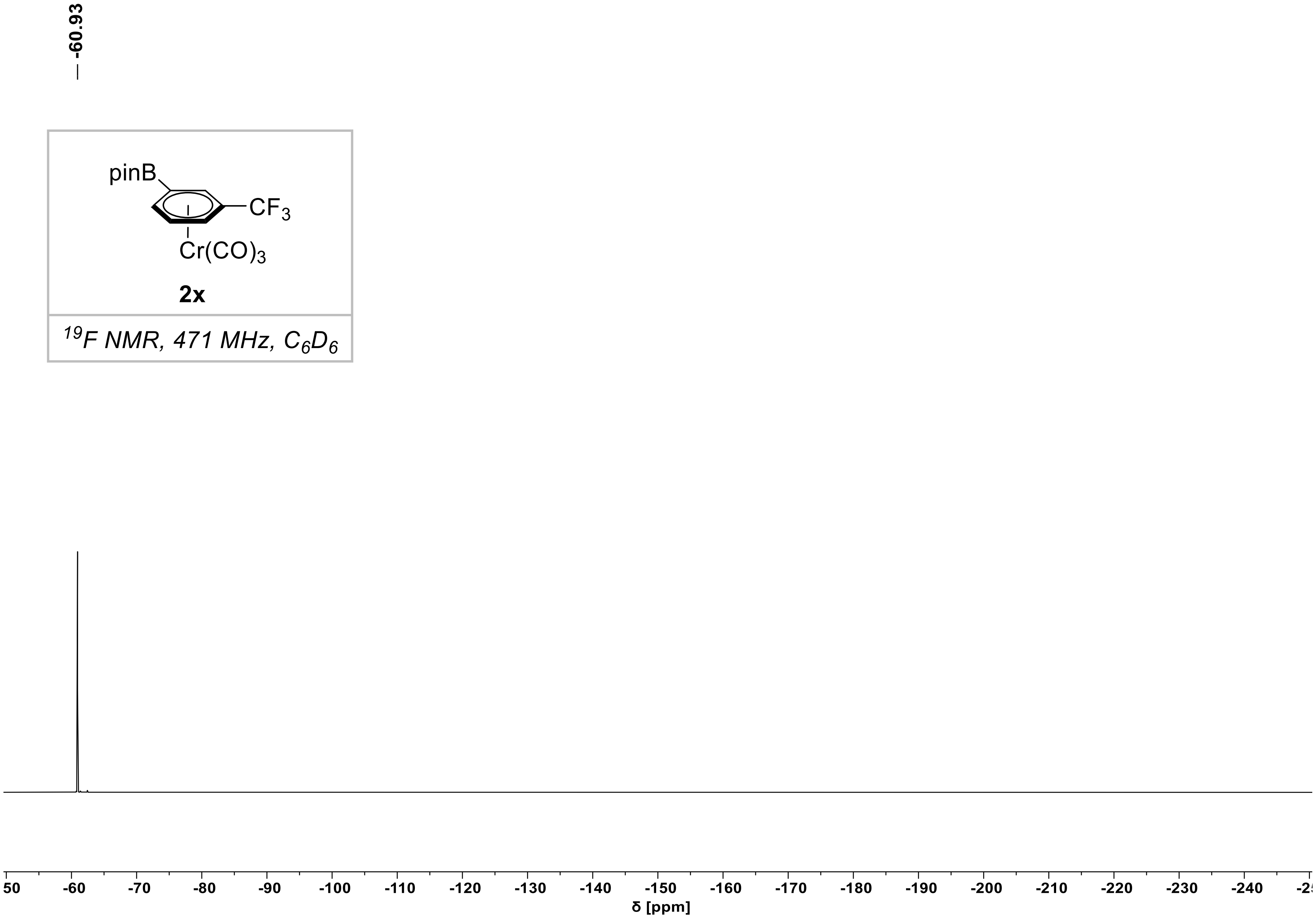

2x

^{19}F NMR, 471 MHz, C_6D_6

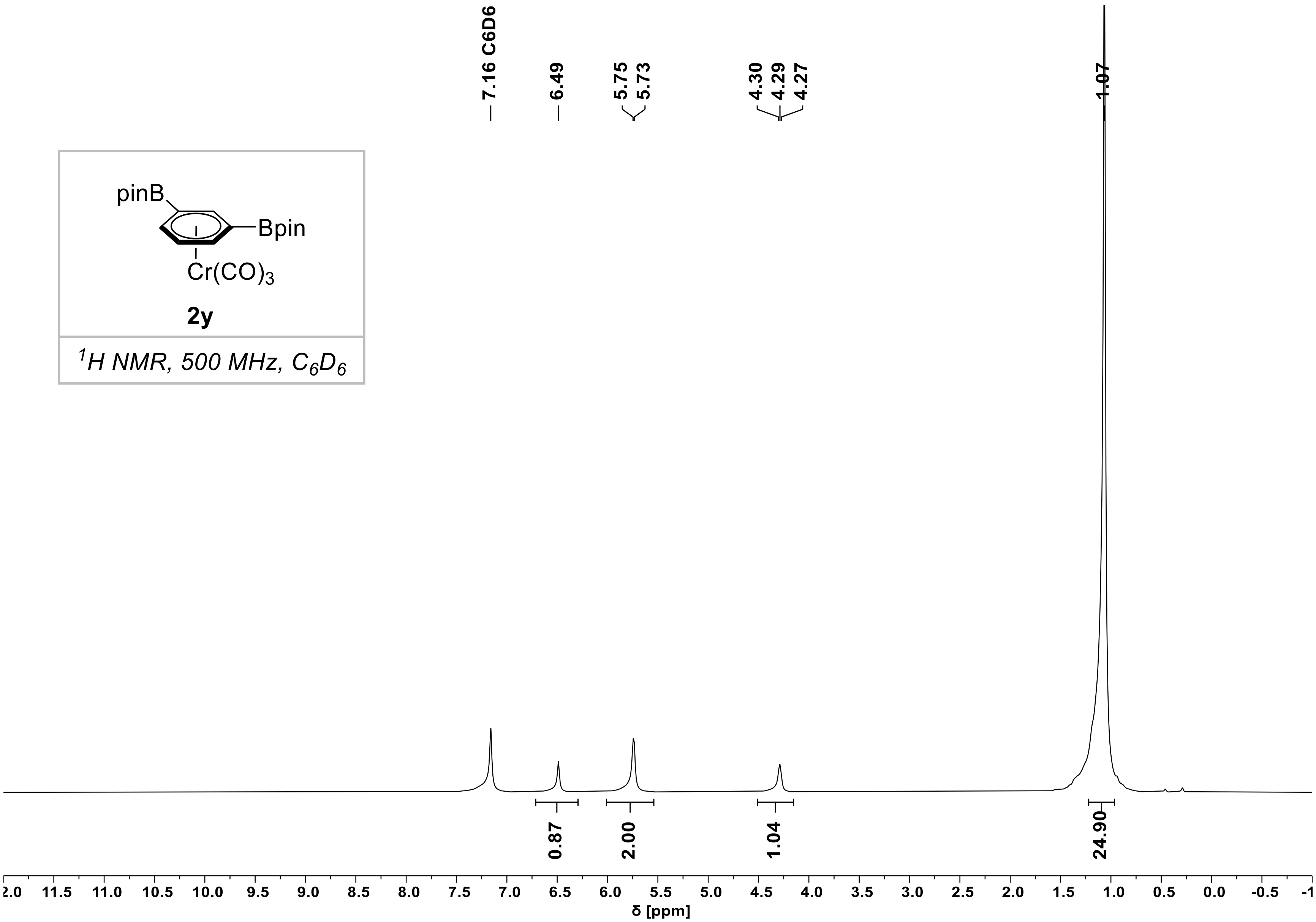
7.16 C6D6
6.49
5.75
5.73
4.30
4.29
4.27
1.07
pinB
Bpin
Cr(CO)3
2y
1H NMR, 500 MHz, C6D6
0.87
2.00
1.04
24.90
δ [ppm]

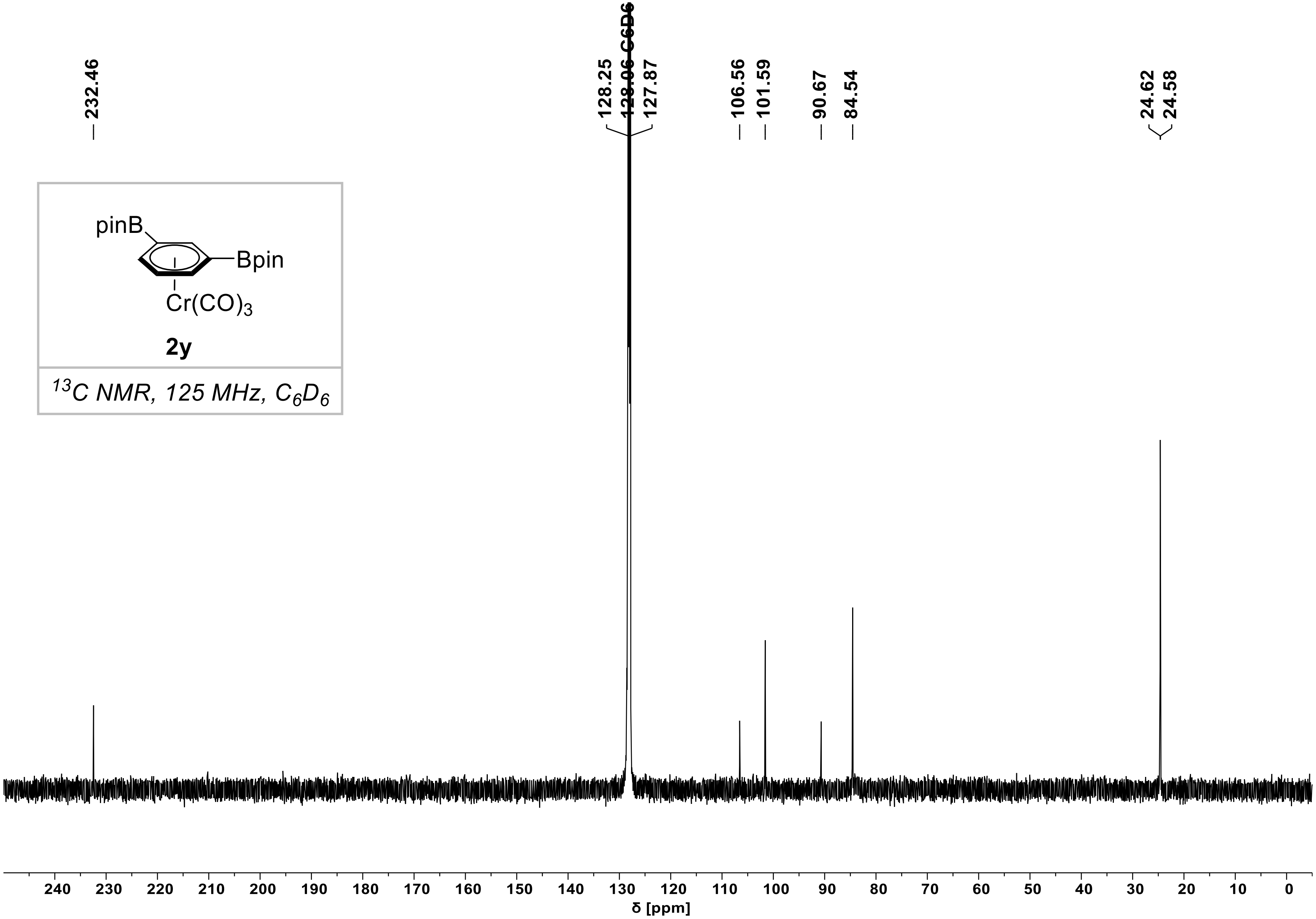
232.46
128.25
128.06 C6D6
127.87
106.56
101.59
90.67
84.54
24.62
24.58
pinB
Bpin
Cr(CO)3
2y
13C NMR, 125 MHz, C6D6
240 230 220 210 200 190 180 170 160 150 140 130 120 110 100 90 80 70 60 50 40 30 20 10 0
δ [ppm]

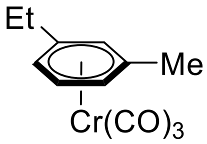

2z

^{1}H NMR, 500 MHz, C_6D_6

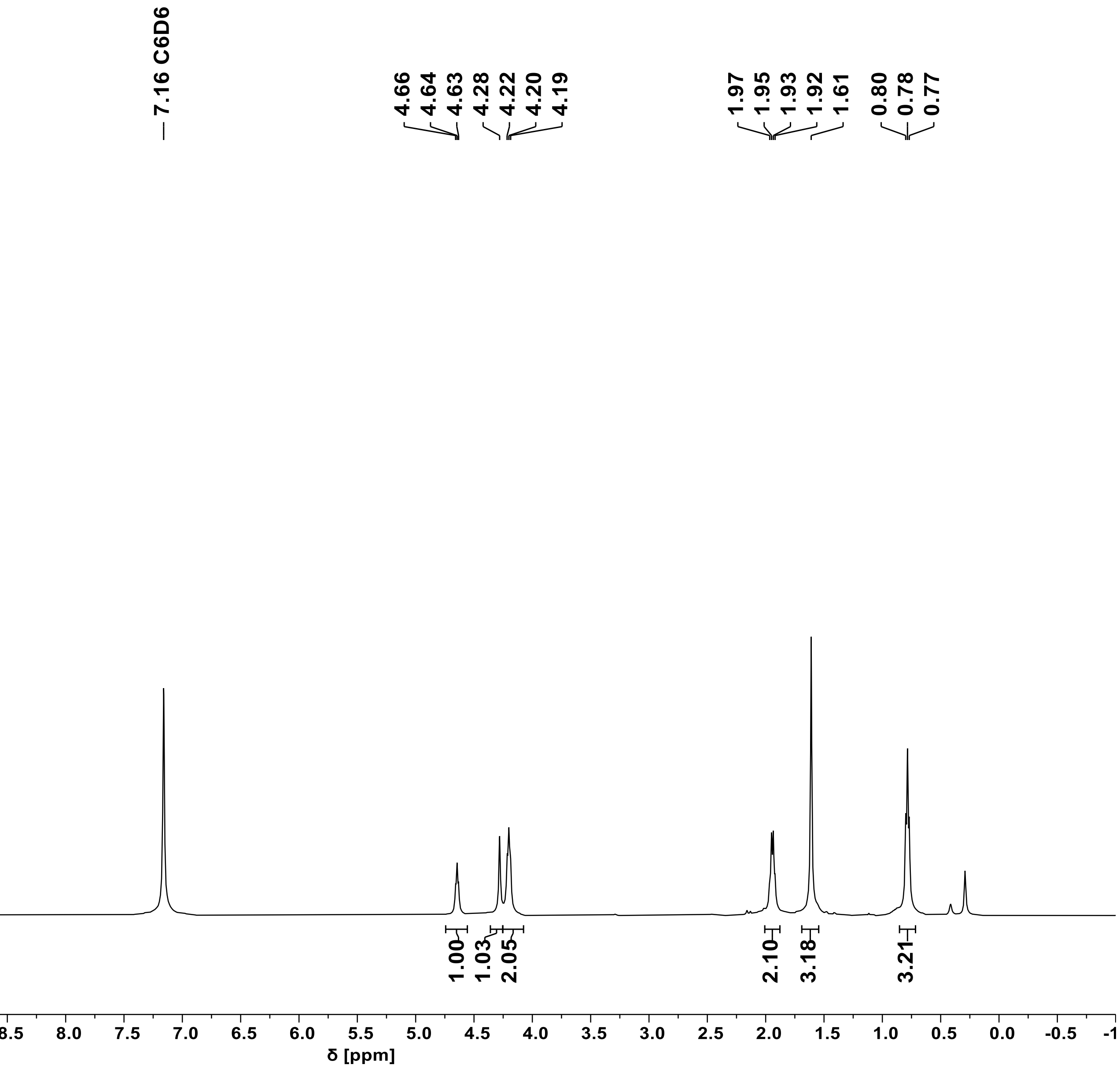

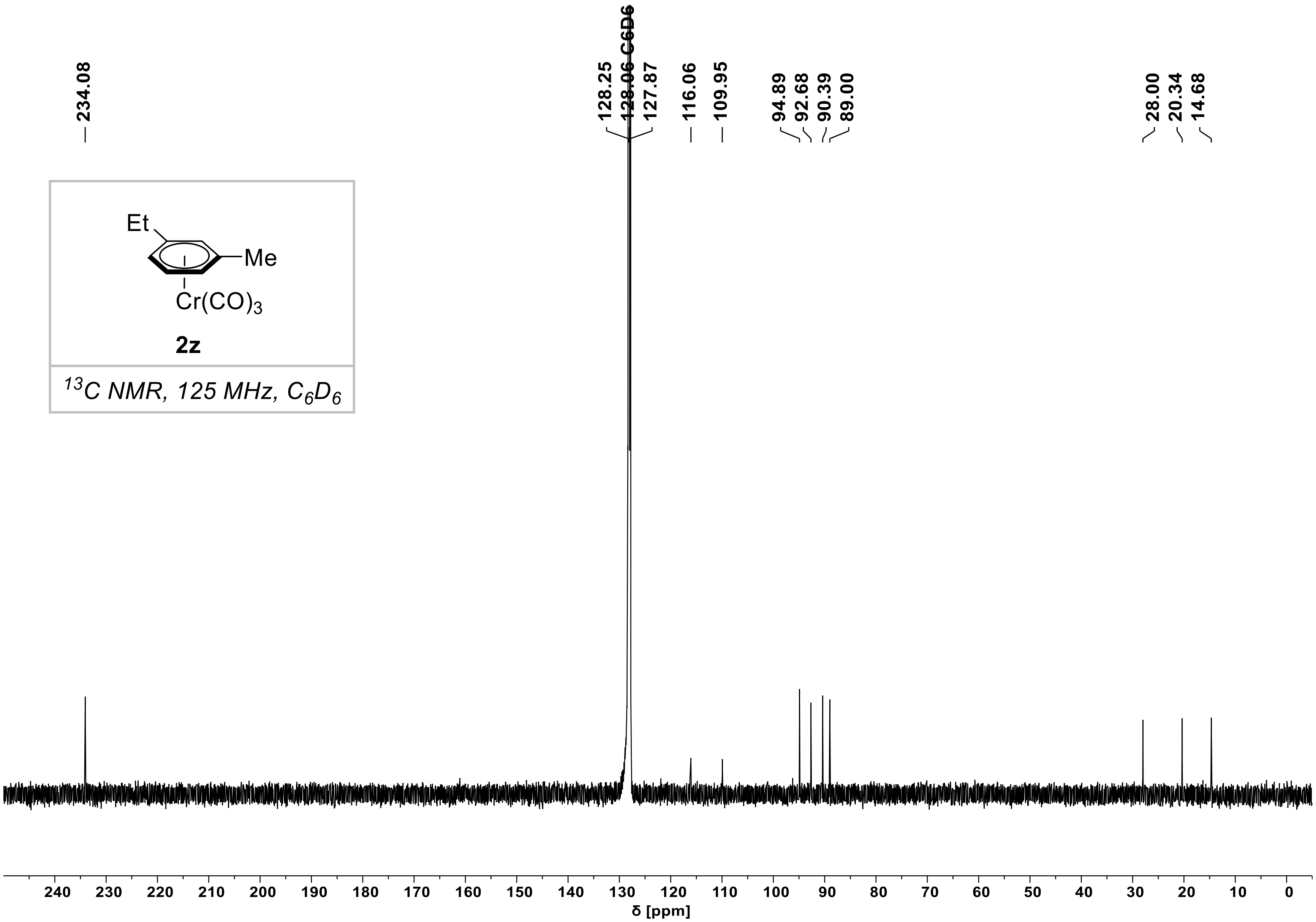
234.08
128.25
128.06 C6D6
127.87
116.06
109.95
94.89
92.68
90.39
89.00
28.00
20.34
14.68
Et
Me
Cr(CO)3
2z
13C NMR, 125 MHz, C6D6
240 230 220 210 200 190 180 170 160 150 140 130 120 110 100 90 80 70 60 50 40 30 20 10 0
δ [ppm]

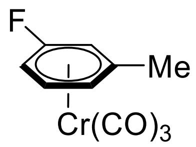

2aa

^{1}H NMR, 400 MHz, C_6D_6

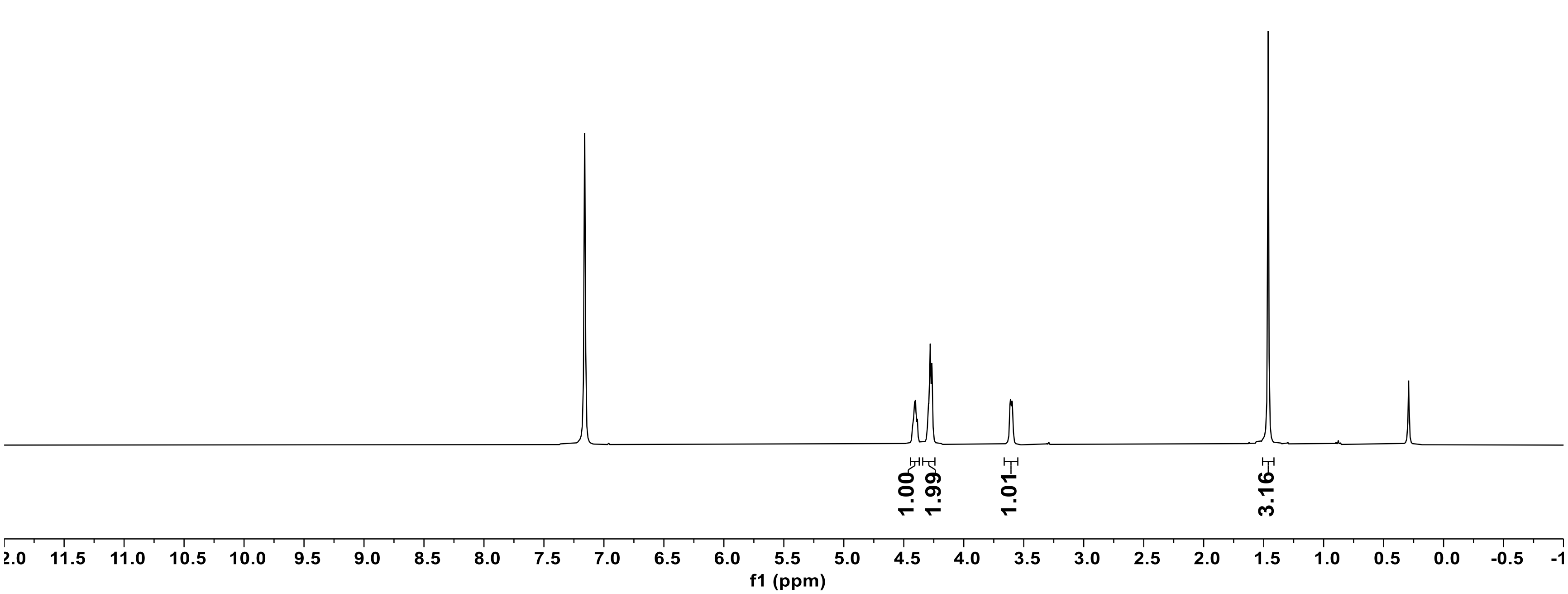

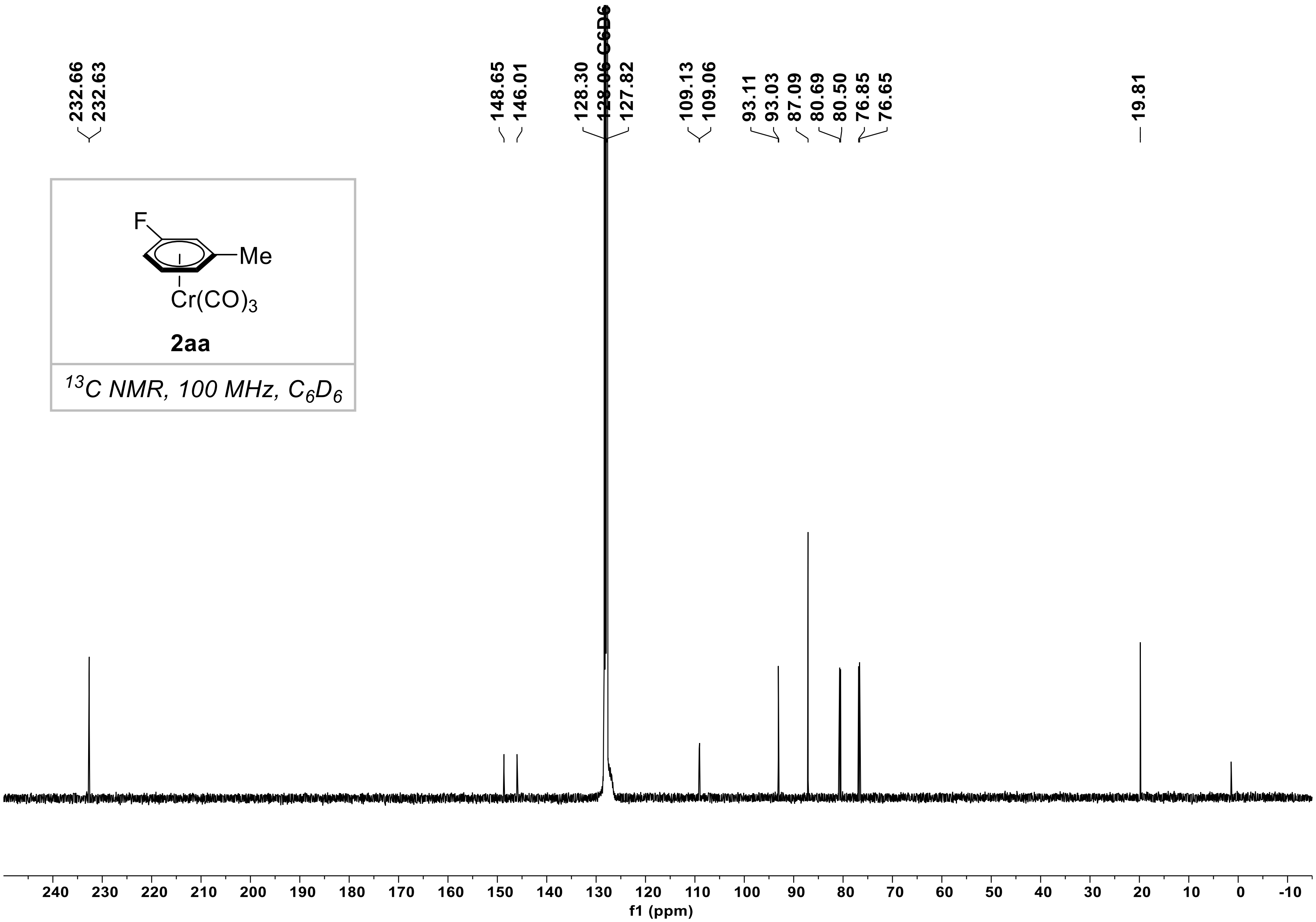
232.66
232.63
148.65
146.01
128.30
128.06 C6D6
127.82
109.13
109.06
93.11
93.03
87.09
80.69
80.50
76.85
76.65
19.81
F
Me
Cr(CO)3
2aa
13C NMR, 100 MHz, C6D6
f1 (ppm)

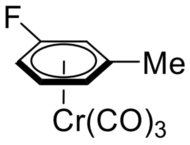

2aa

^{19}F NMR, 471 MHz, C_6D_6

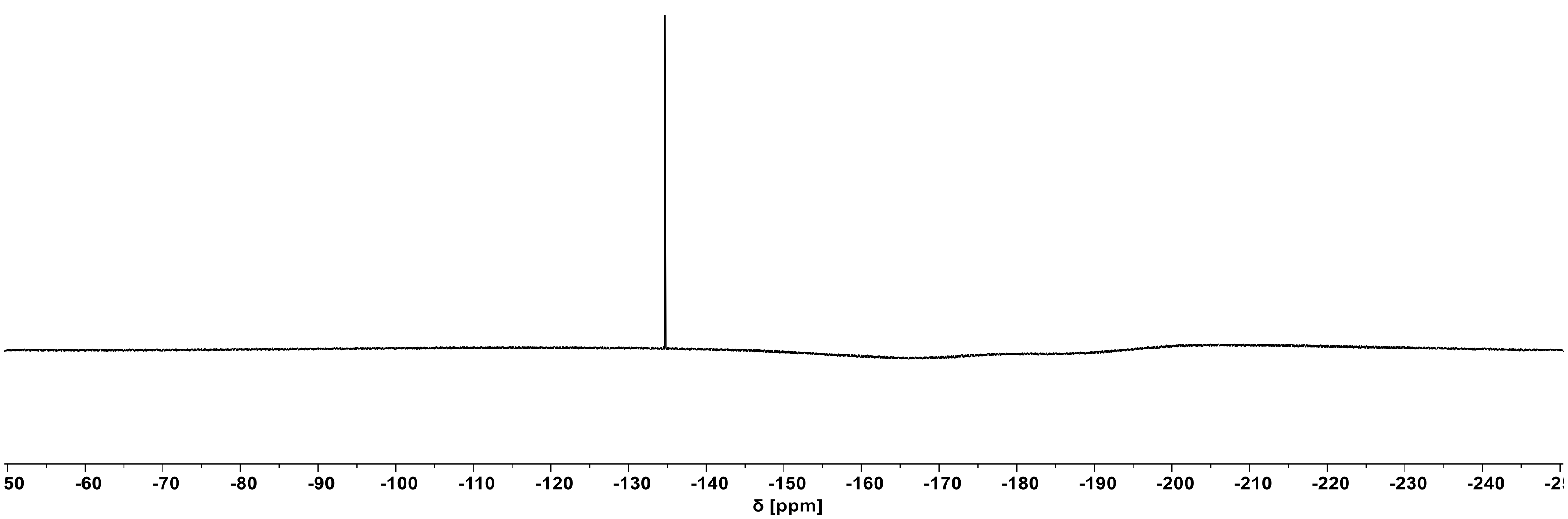

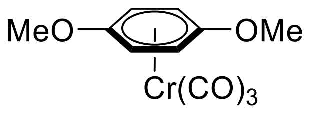

2ab

¹H NMR, 500 MHz, C_6D_6

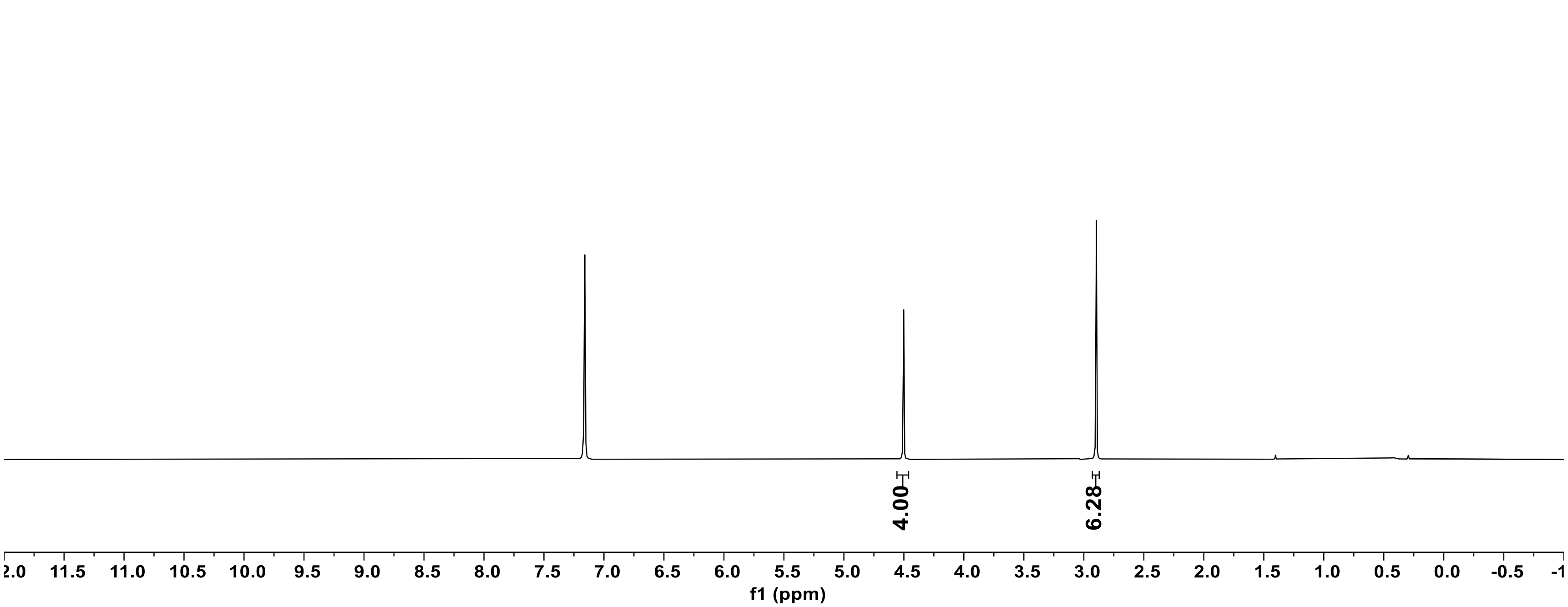

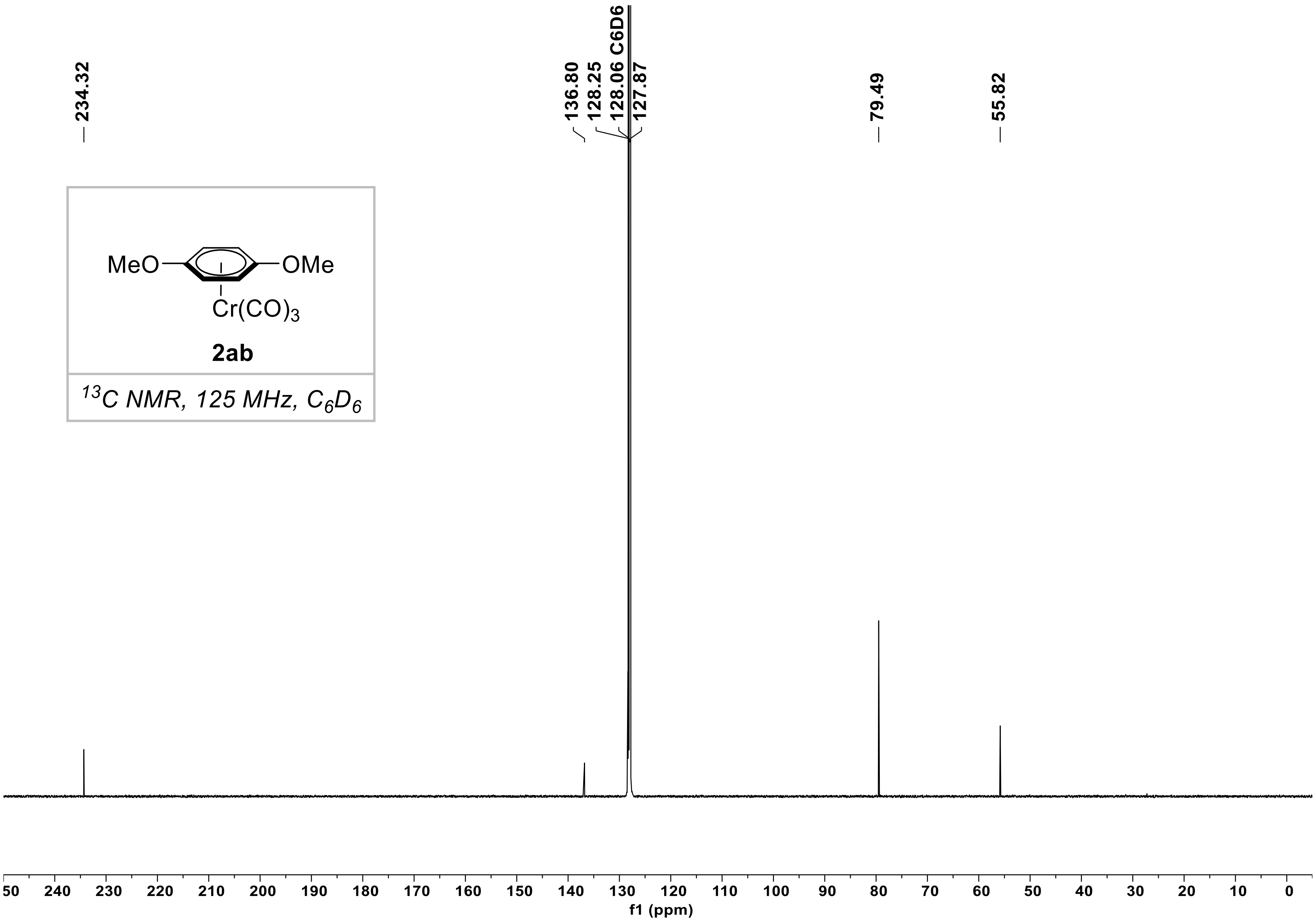
234.32
136.80
128.25
128.06 C6D6
127.87
79.49
55.82
MeO
OMe
Cr(CO)3
2ab
13C NMR, 125 MHz, C6D6
f1 (ppm)

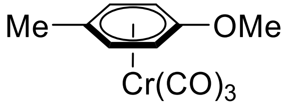

2ac

^{1}H NMR, 500 MHz, C_6D_6

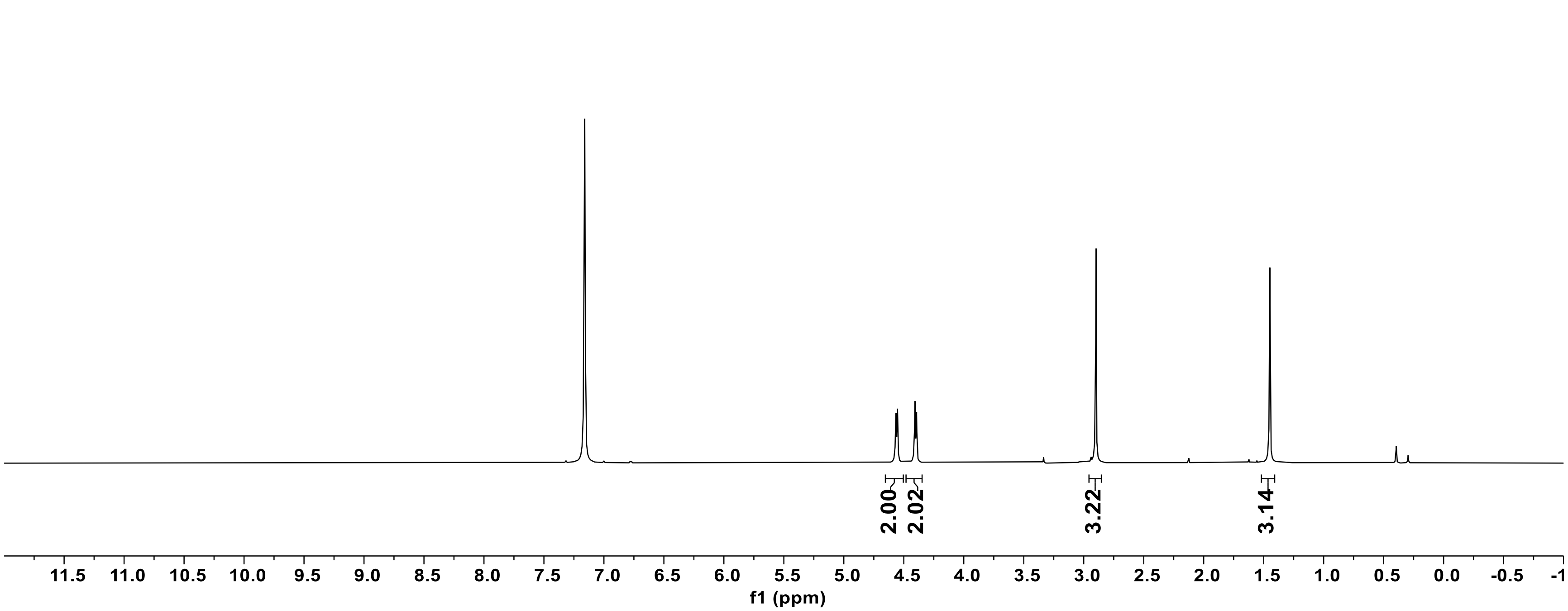

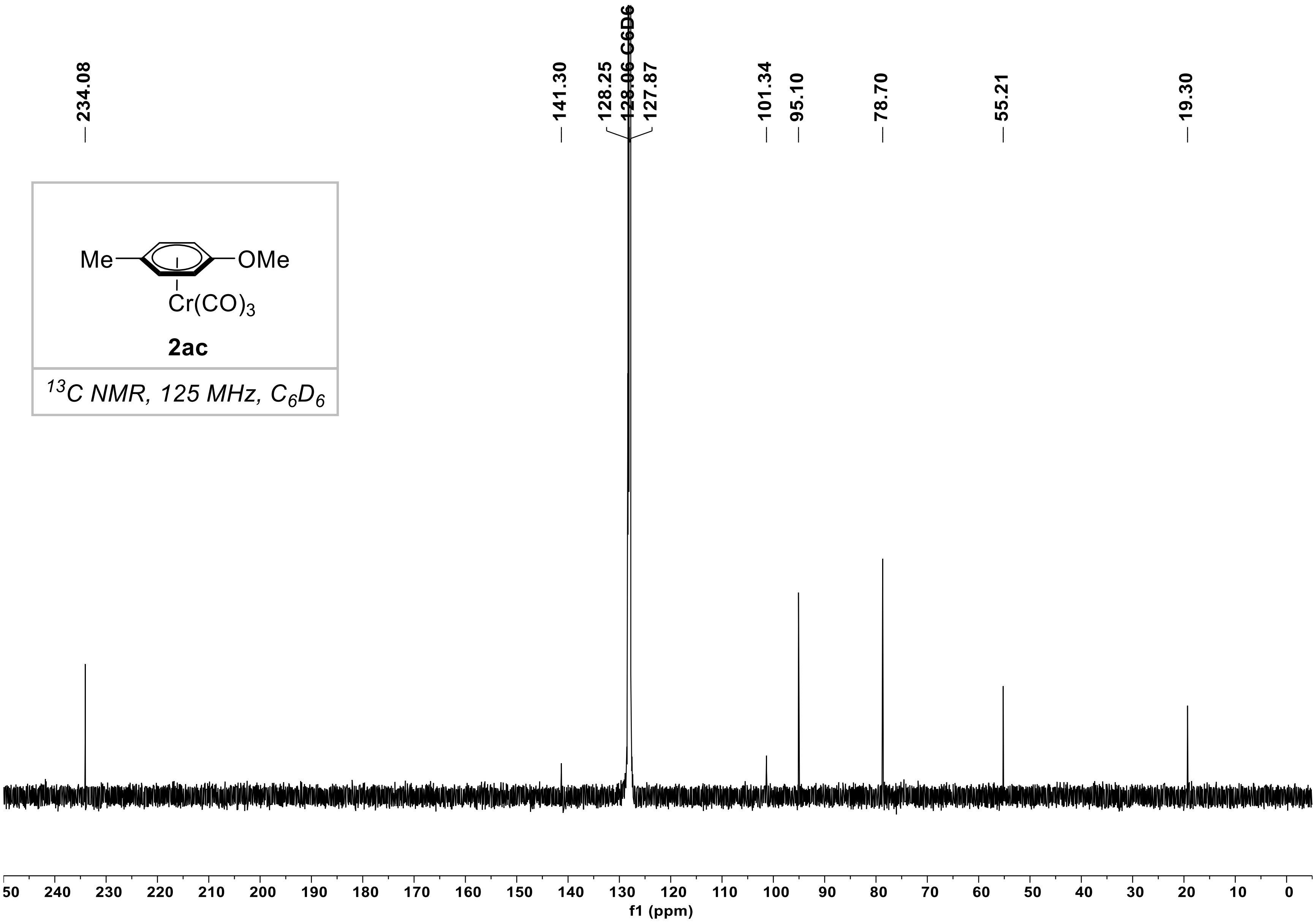
234.08
141.30
128.25
128.06 C6D6
127.87
101.34
95.10
78.70
55.21
19.30
Me
OMe
Cr(CO)3
2ac
13C NMR, 125 MHz, C6D6
50
240
230
220
210
200
190
180
170
160
150
140
130
120
110
100
90
80
70
60
50
40
30
20
10
0
f1 (ppm)

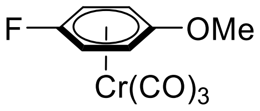

2ad

^{1}H NMR, 400 MHz, C_6D_6

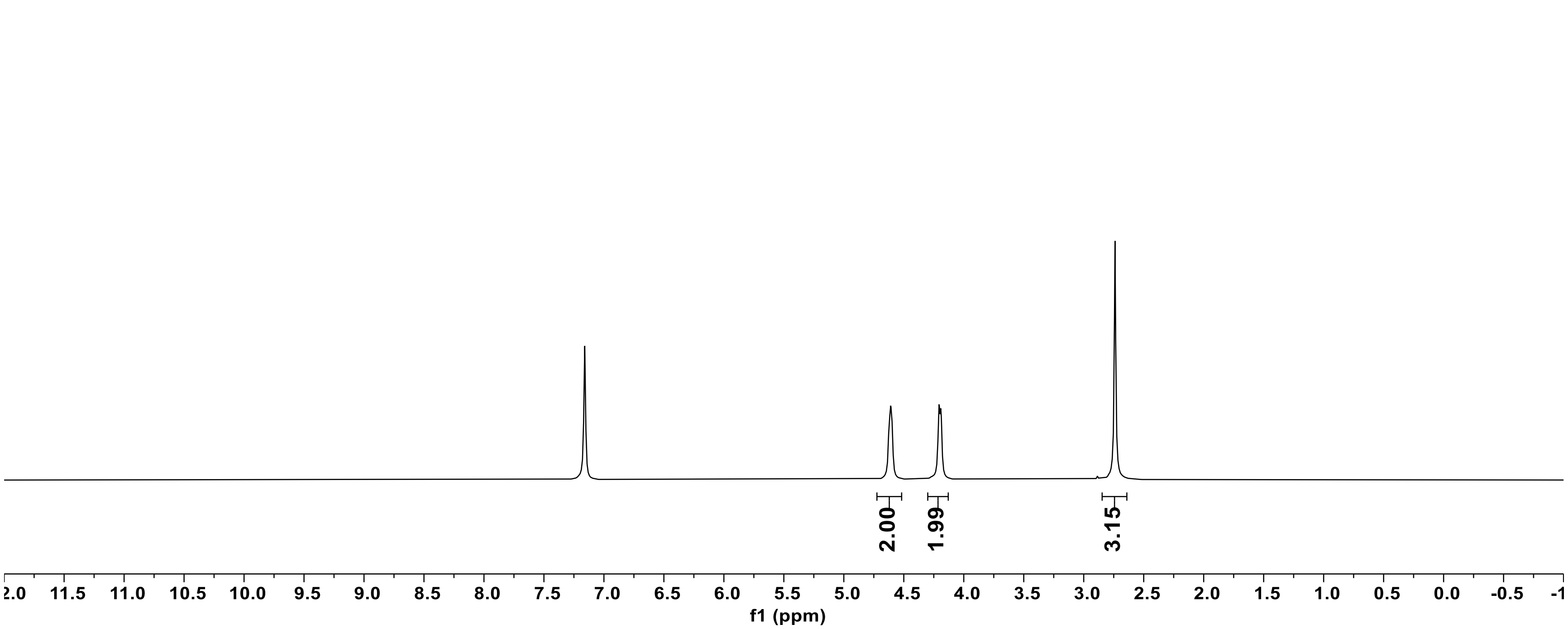

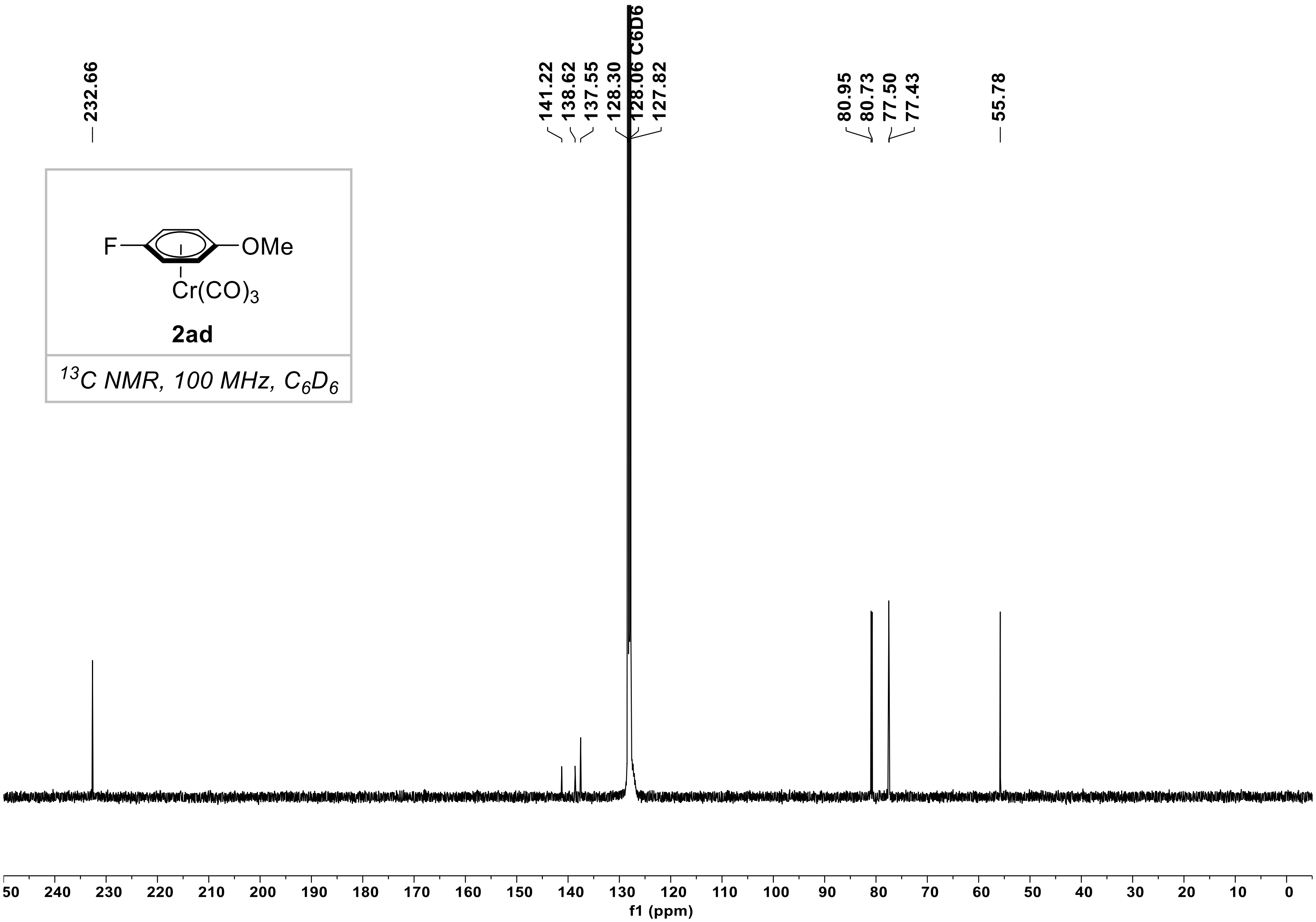

232.66
141.22
138.62
137.55
128.30
128.06 C6D6
127.82
80.95
80.73
77.50
77.43
55.78
F
OMe
Cr(CO)3
2ad
13C NMR, 100 MHz, C6D6
f1 (ppm)

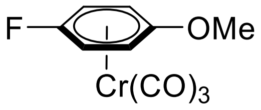

2ad

^{19}F NMR, 471 MHz, C_6D_6

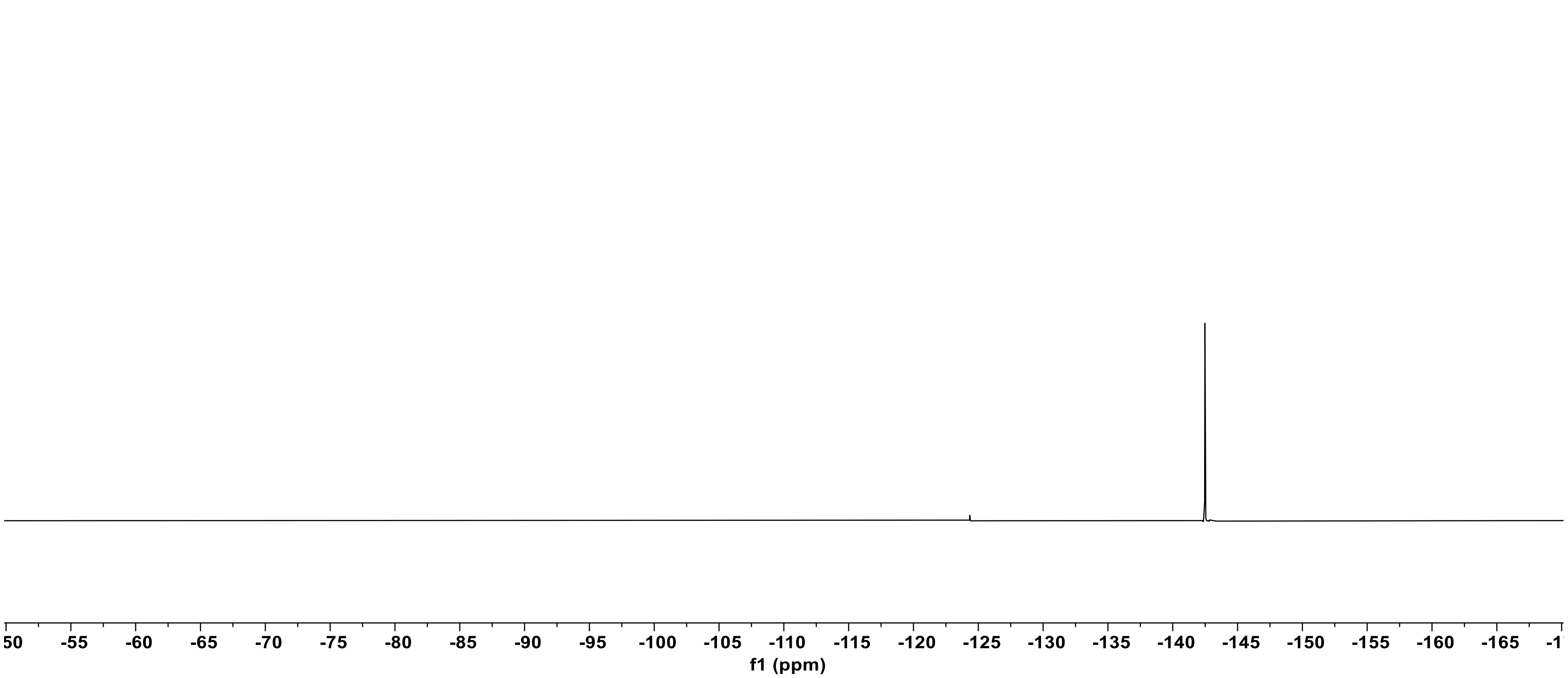

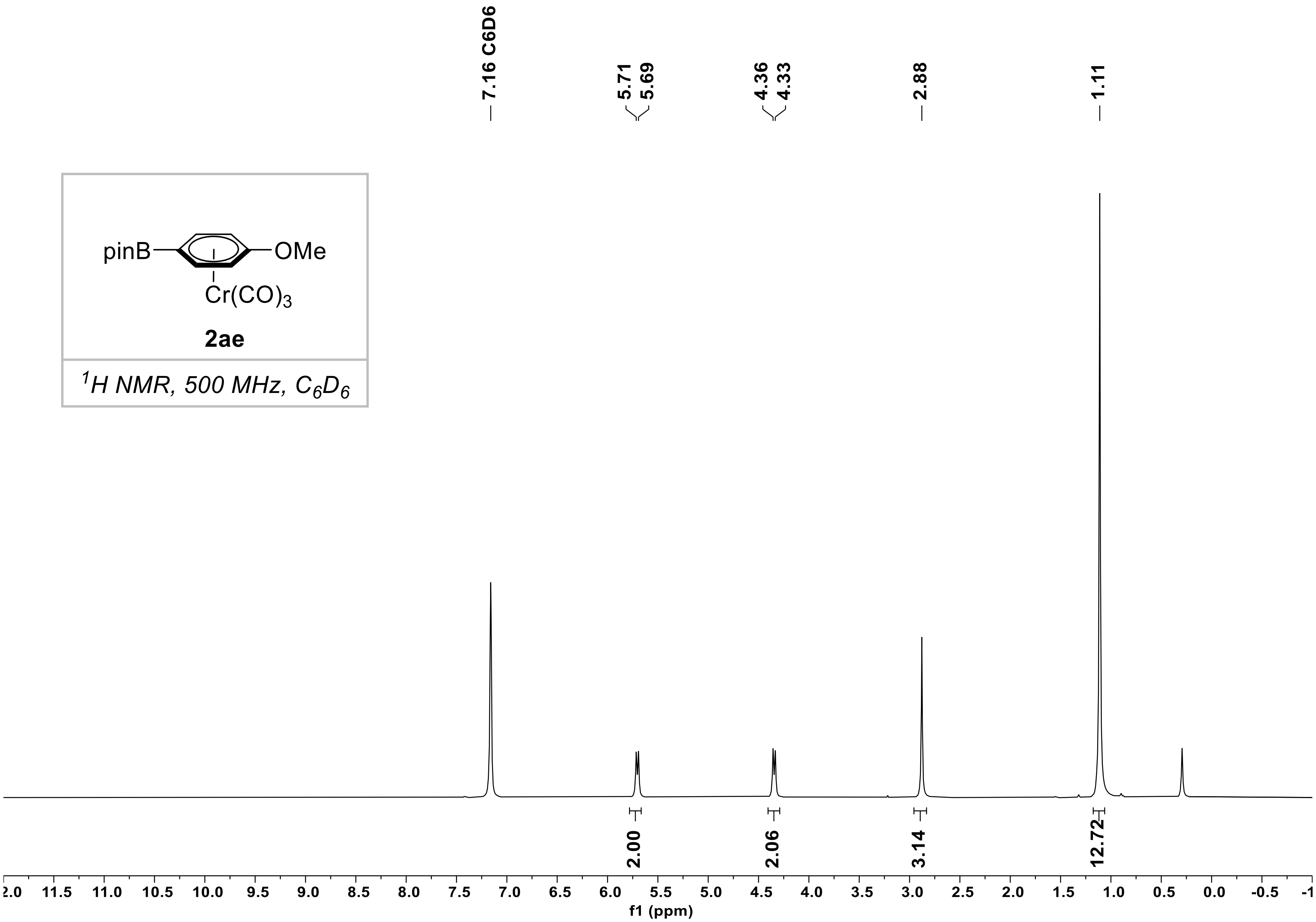
7.16 C6D6
5.71
5.69
4.36
4.33
2.88
1.11
pinB
OMe
Cr(CO)3
2ae
1H NMR, 500 MHz, C6D6
2.00
2.06
3.14
12.72
f1 (ppm)

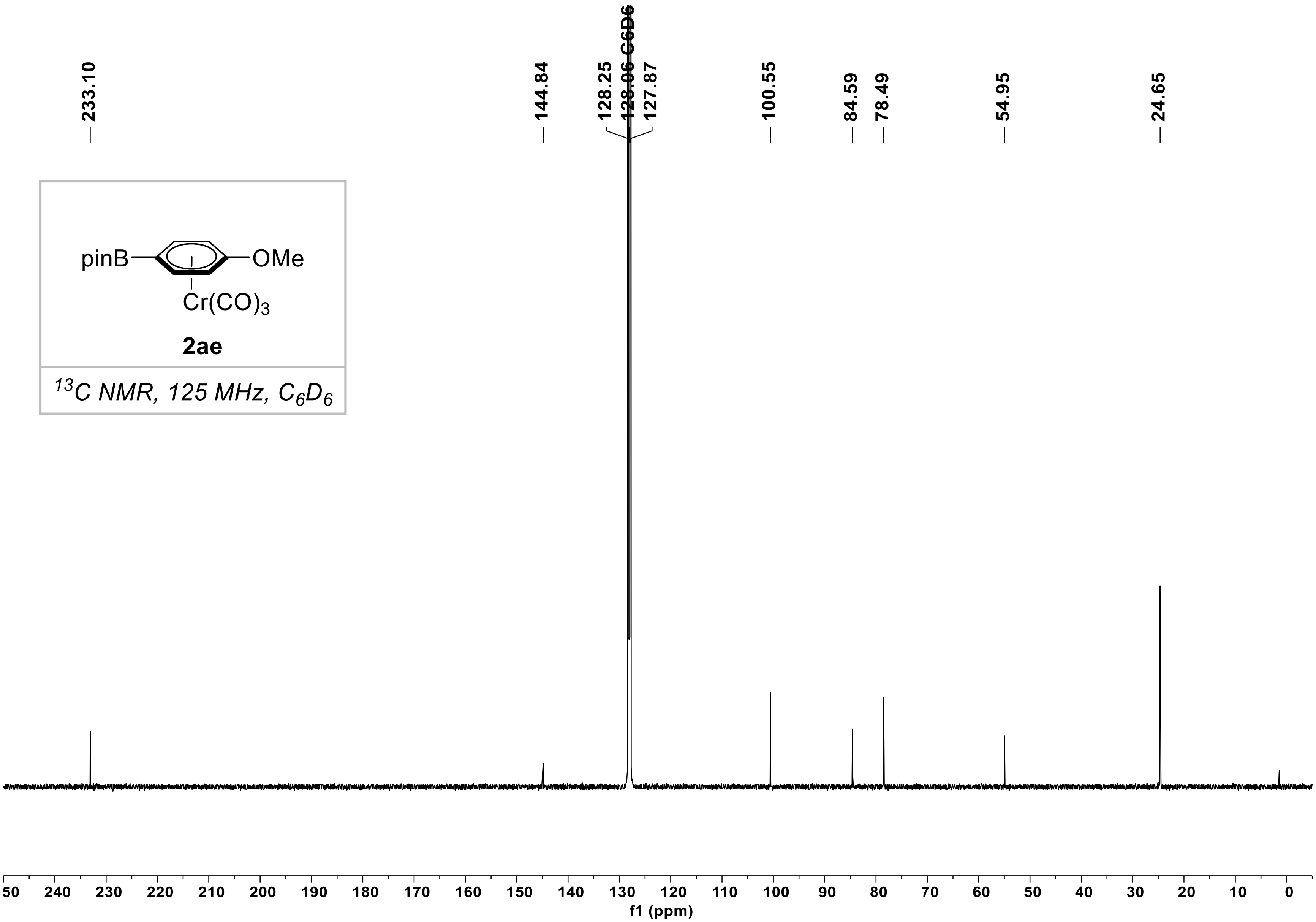

2ae

^{13}C NMR, 125 MHz, C_6D_6

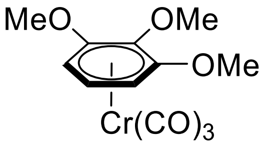

2af

^{1}H NMR, 500 MHz, C_6D_6

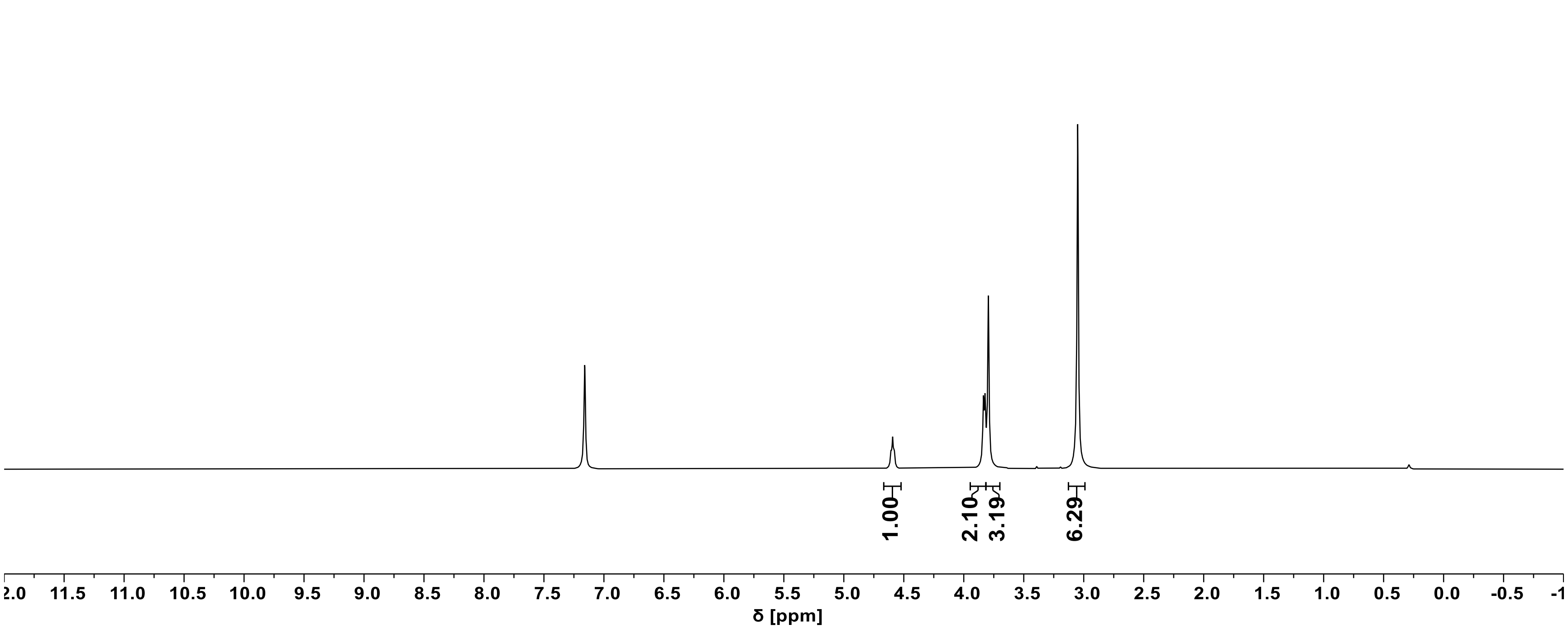

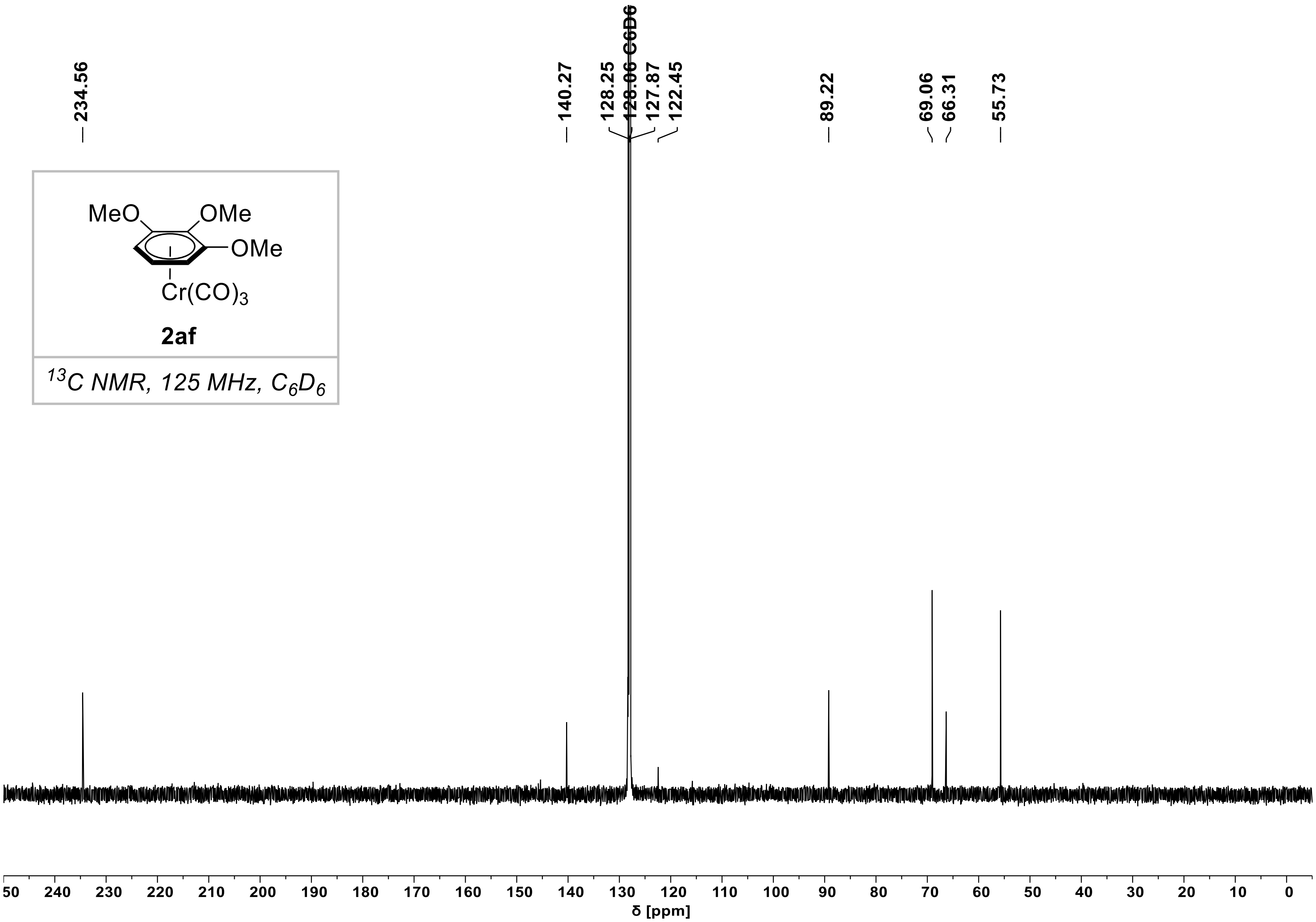
234.56
140.27
128.25
128.06 C6D6
127.87
122.45
89.22
69.06
66.31
55.73
MeO
OMe
OMe
Cr(CO)3
2af
13C NMR, 125 MHz, C6D6
250
240
230
220
210
200
190
180
170
160
150
140
130
120
110
100
90
80
70
60
50
40
30
20
10
0
δ [ppm]

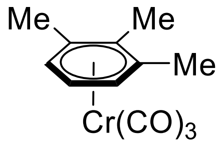

2ag

^{1}H NMR, 500 MHz, C_6D_6

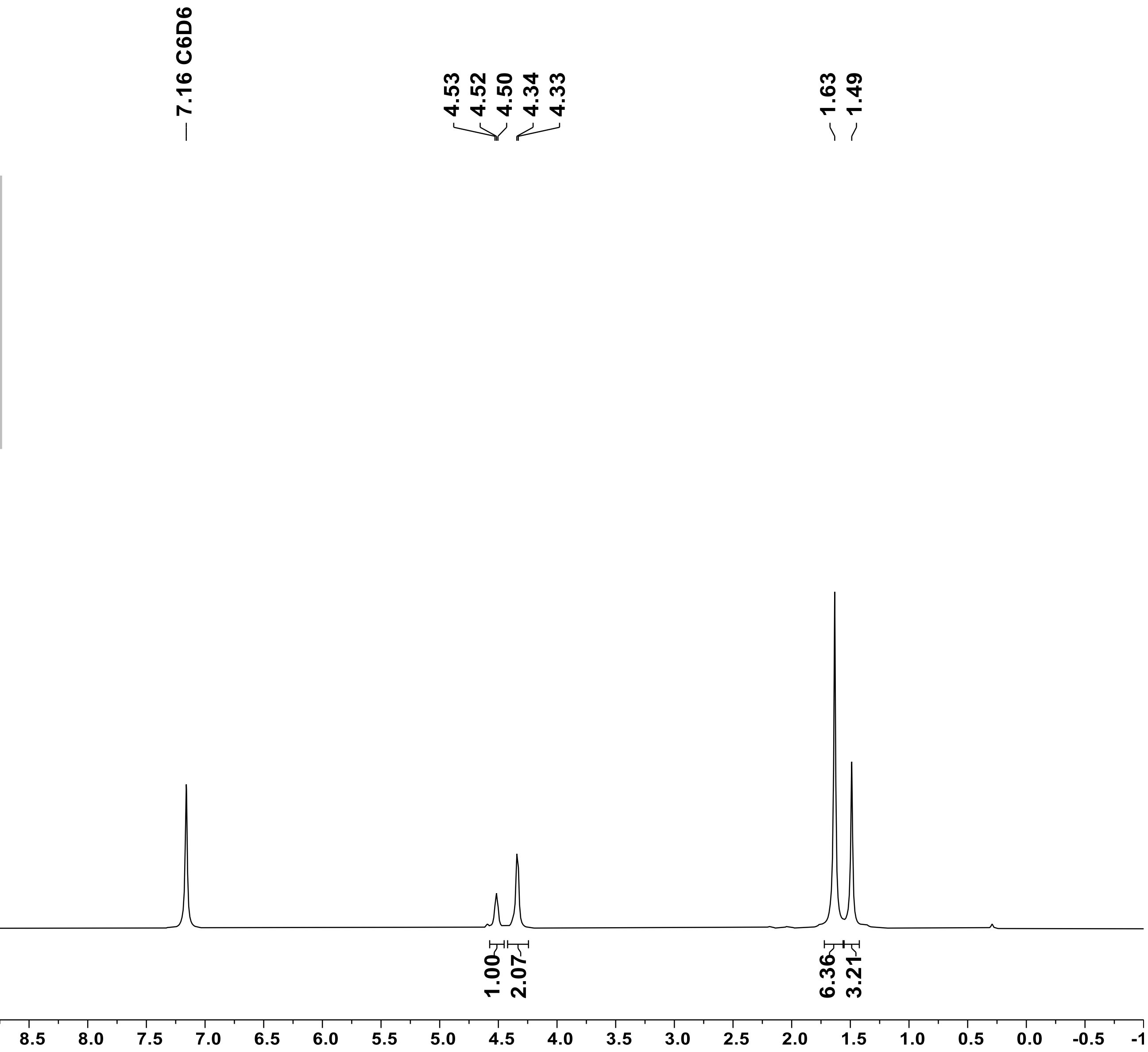

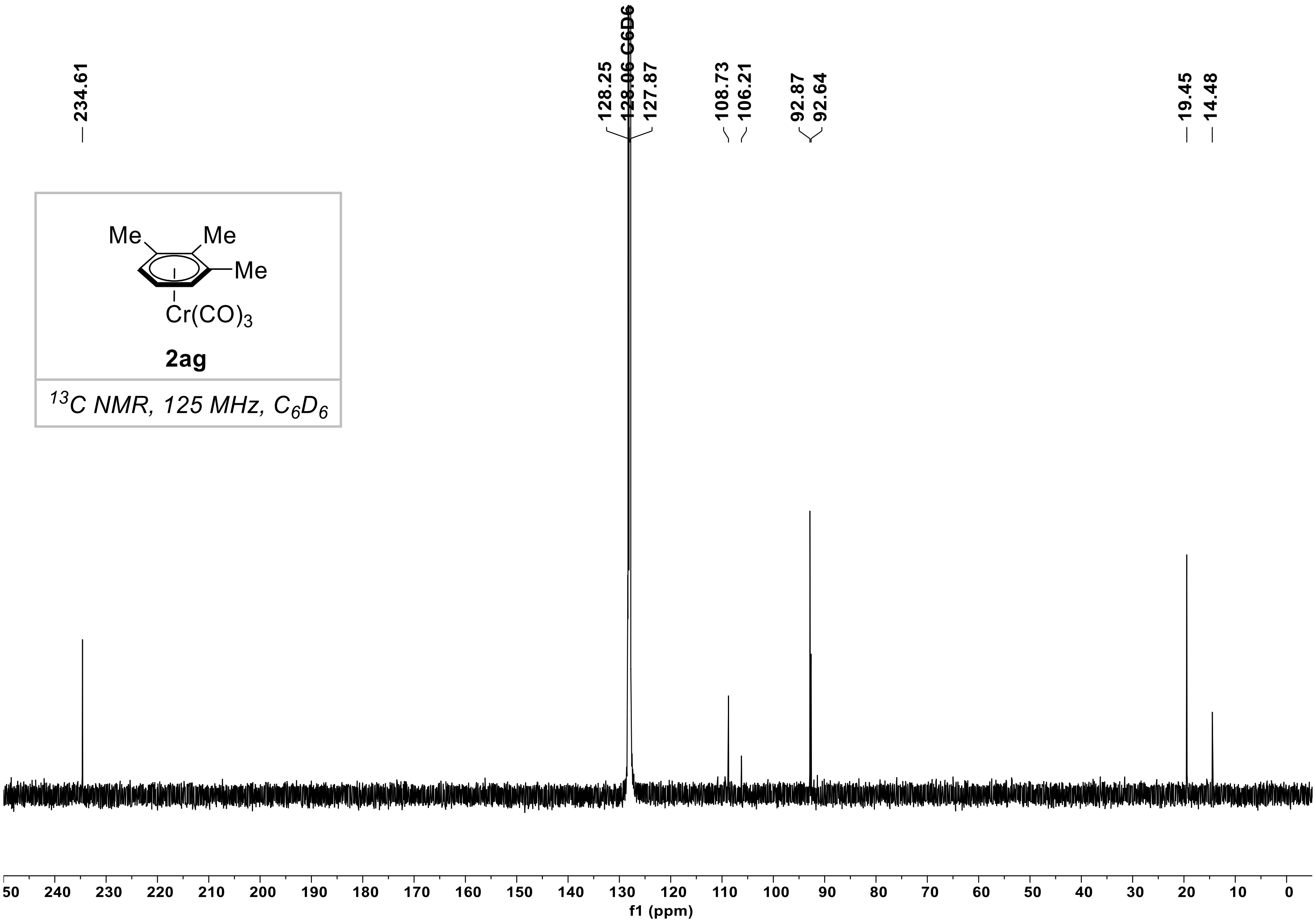
234.61
128.25
128.06 C6D6
127.87
108.73
106.21
92.87
92.64
19.45
14.48
Me
Me
Me
Cr(CO)3
2ag
13C NMR, 125 MHz, C6D6
250
240
230
220
210
200
190
180
170
160
150
140
130
120
110
100
90
80
70
60
50
40
30
20
10
0
f1 (ppm)

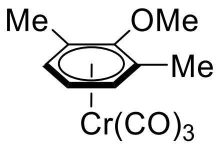

2ah

^{1}H NMR, 400 MHz, C_6D_6

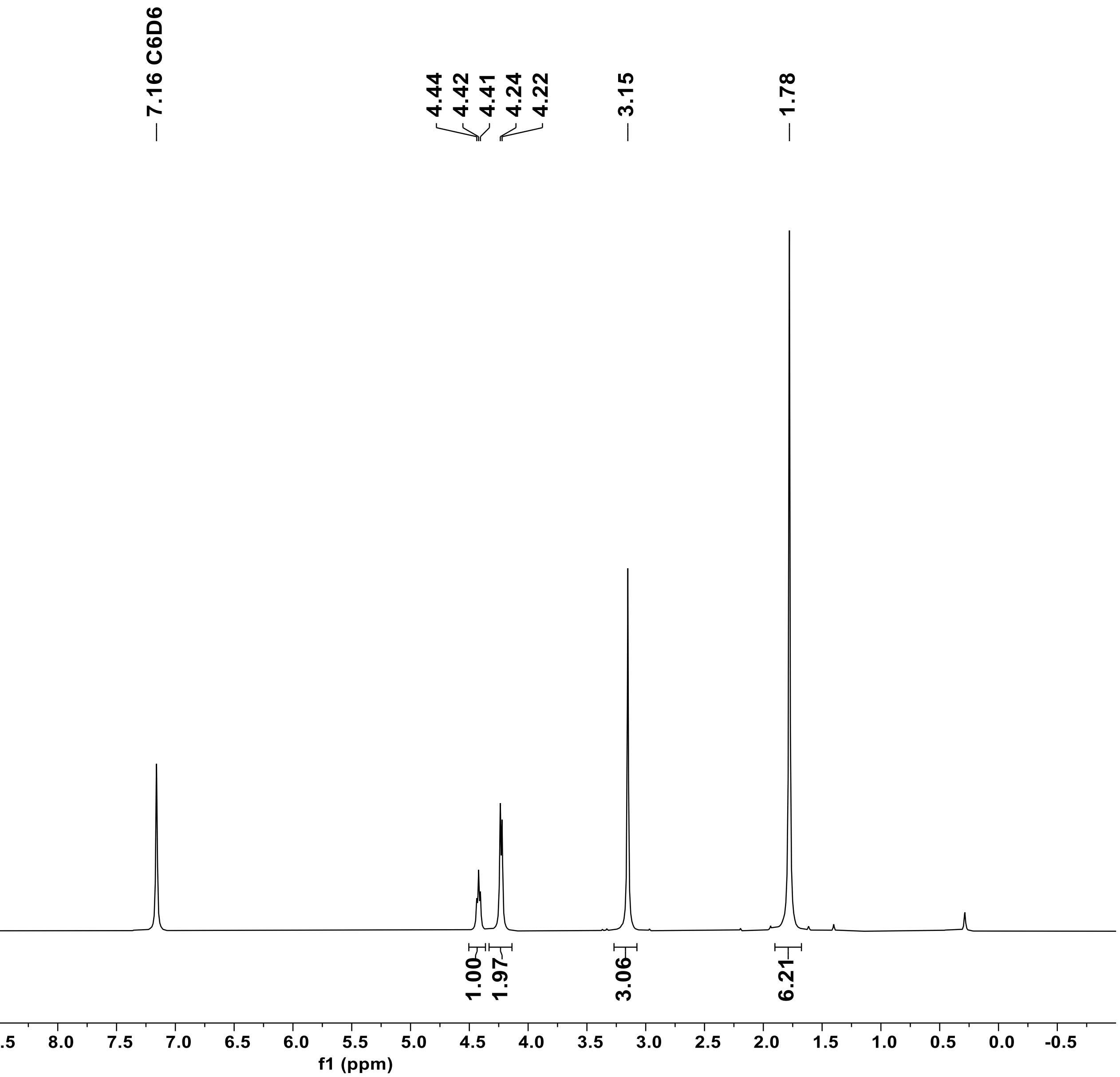

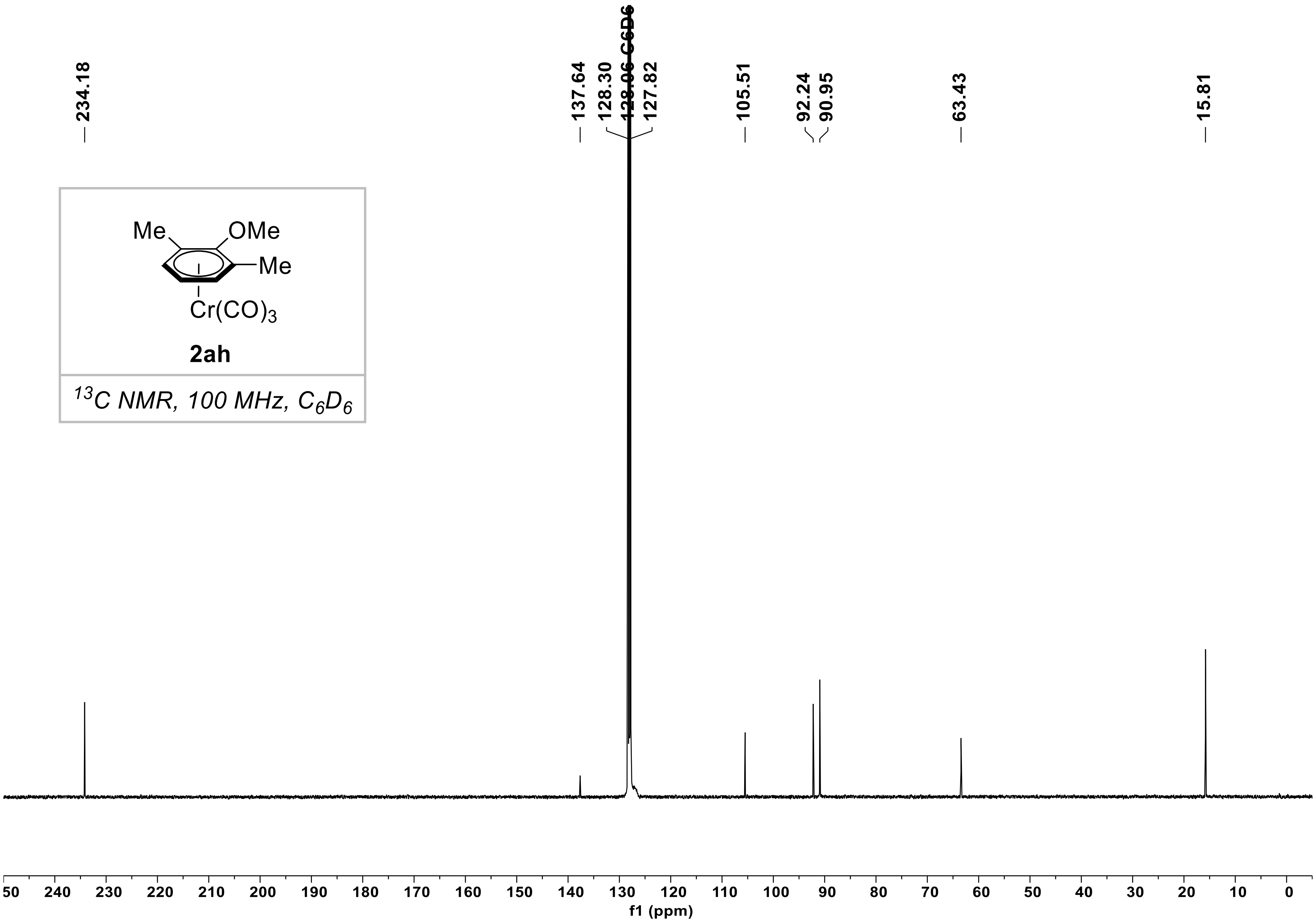
234.18
137.64
128.30
128.06 C6D6
127.82
105.51
92.24
90.95
63.43
15.81
Me
OMe
Me
Cr(CO)3
2ah
13C NMR, 100 MHz, C6D6
50 240 230 220 210 200 190 180 170 160 150 140 130 120 110 100 90 80 70 60 50 40 30 20 10 0
f1 (ppm)

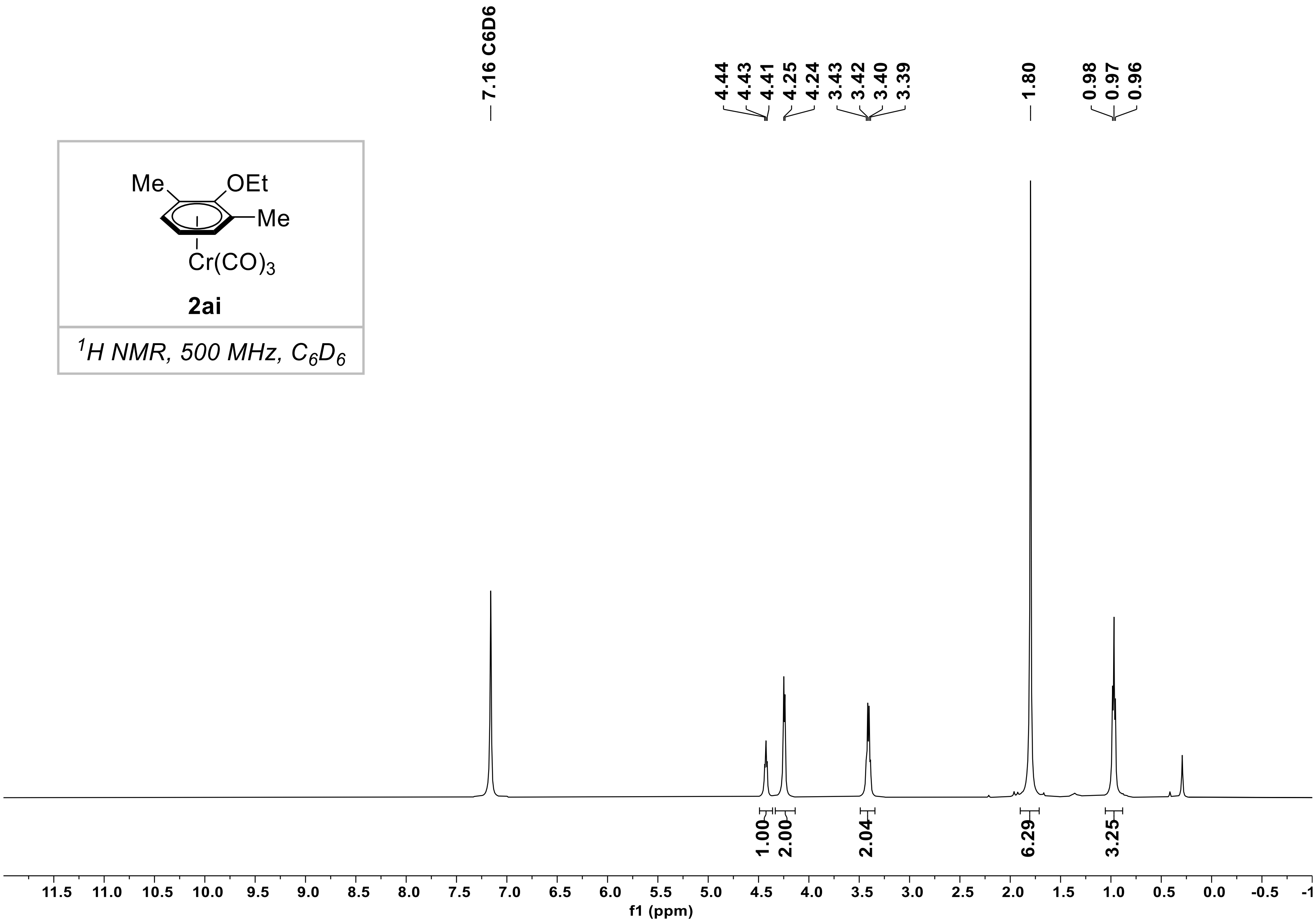
7.16 C6D6
4.44
4.43
4.41
4.25
4.24
3.43
3.42
3.40
3.39
1.80
0.98
0.97
0.96
Me
OEt
Me
Cr(CO)3
2ai
1H NMR, 500 MHz, C6D6
1.00
2.00
2.04
6.29
3.25
11.5
11.0
10.5
10.0
9.5
9.0
8.5
8.0
7.5
7.0
6.5
6.0
5.5
5.0
4.5
4.0
3.5
3.0
2.5
2.0
1.5
1.0
0.5
0.0
-0.5
-1
f1 (ppm)

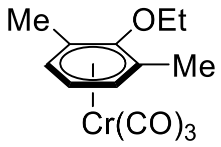

2ai

^{13}C NMR, 125 MHz, C_6D_6

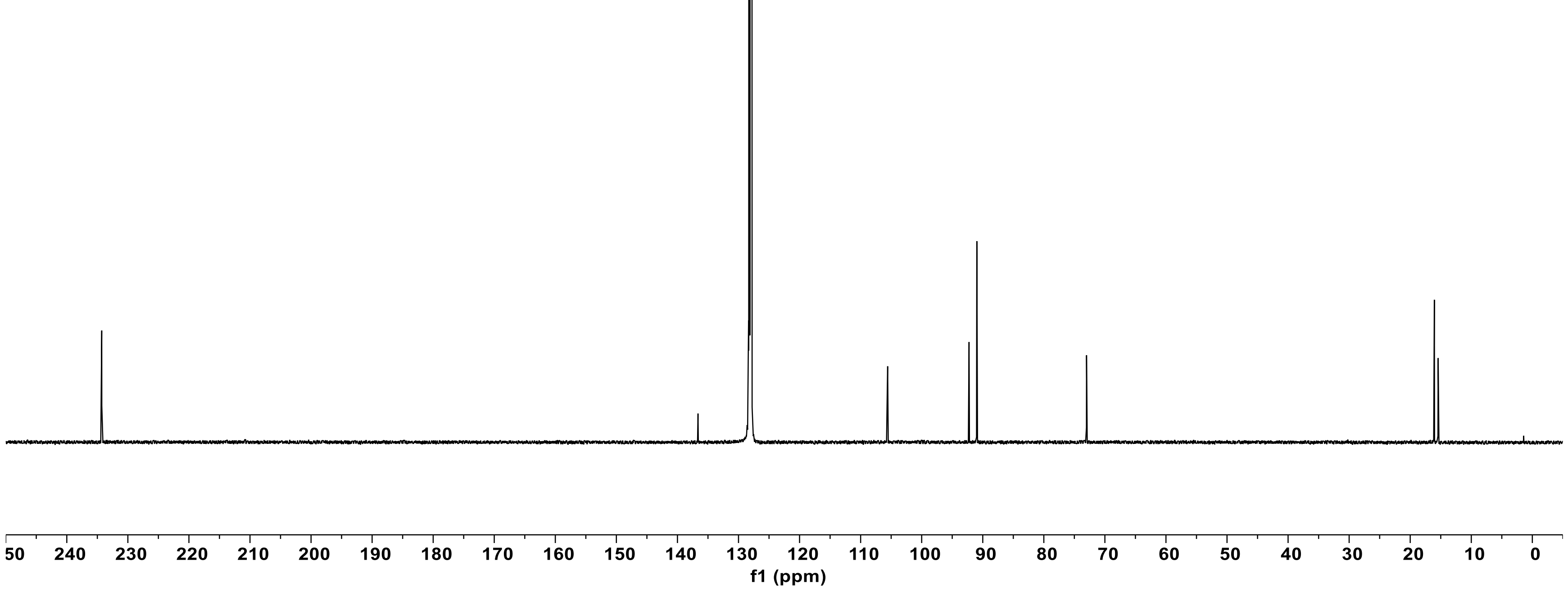

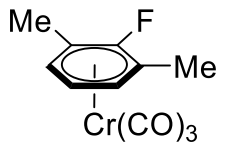

2aj

^{1}H NMR, 500 MHz, C_6D_6

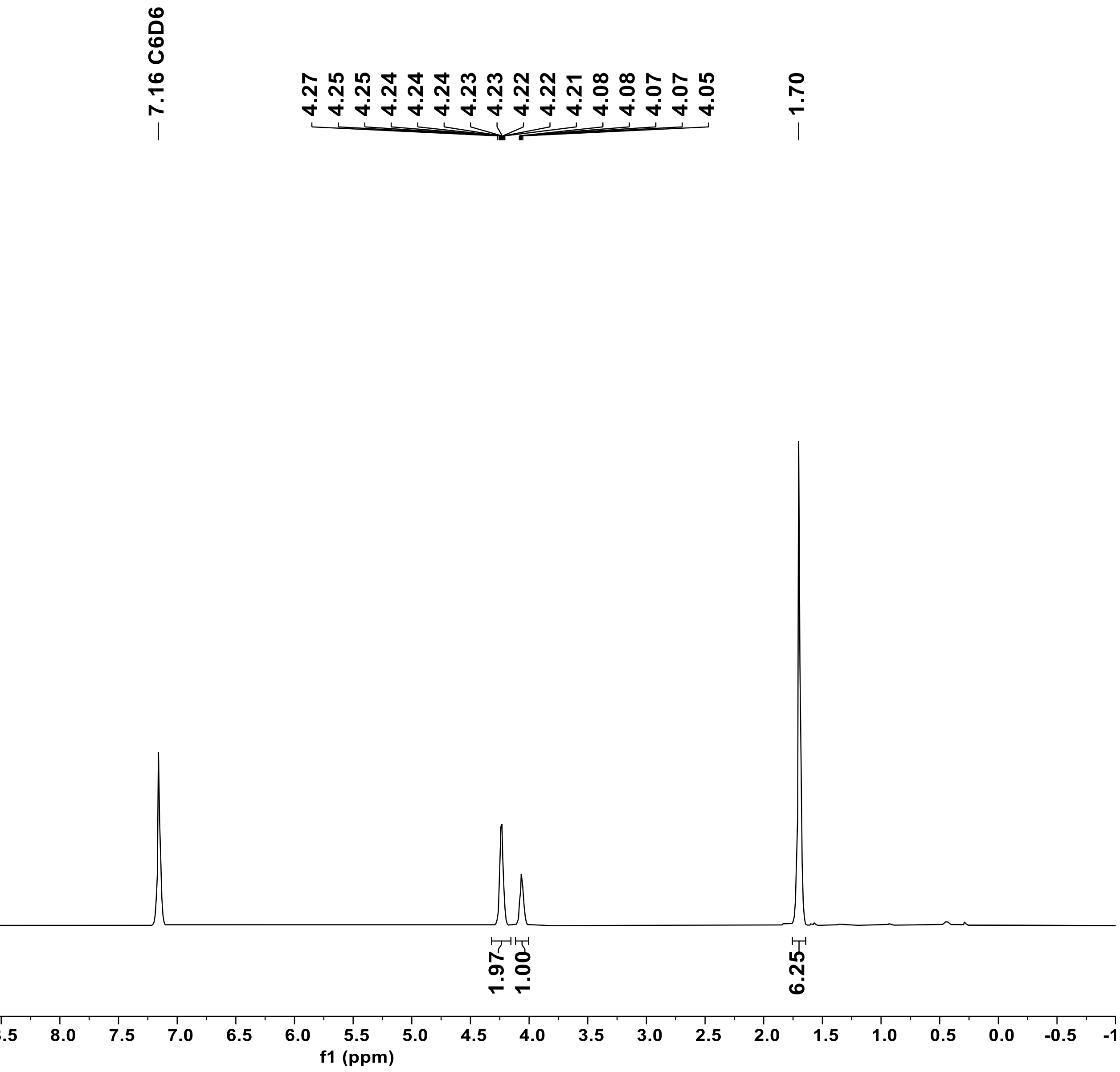

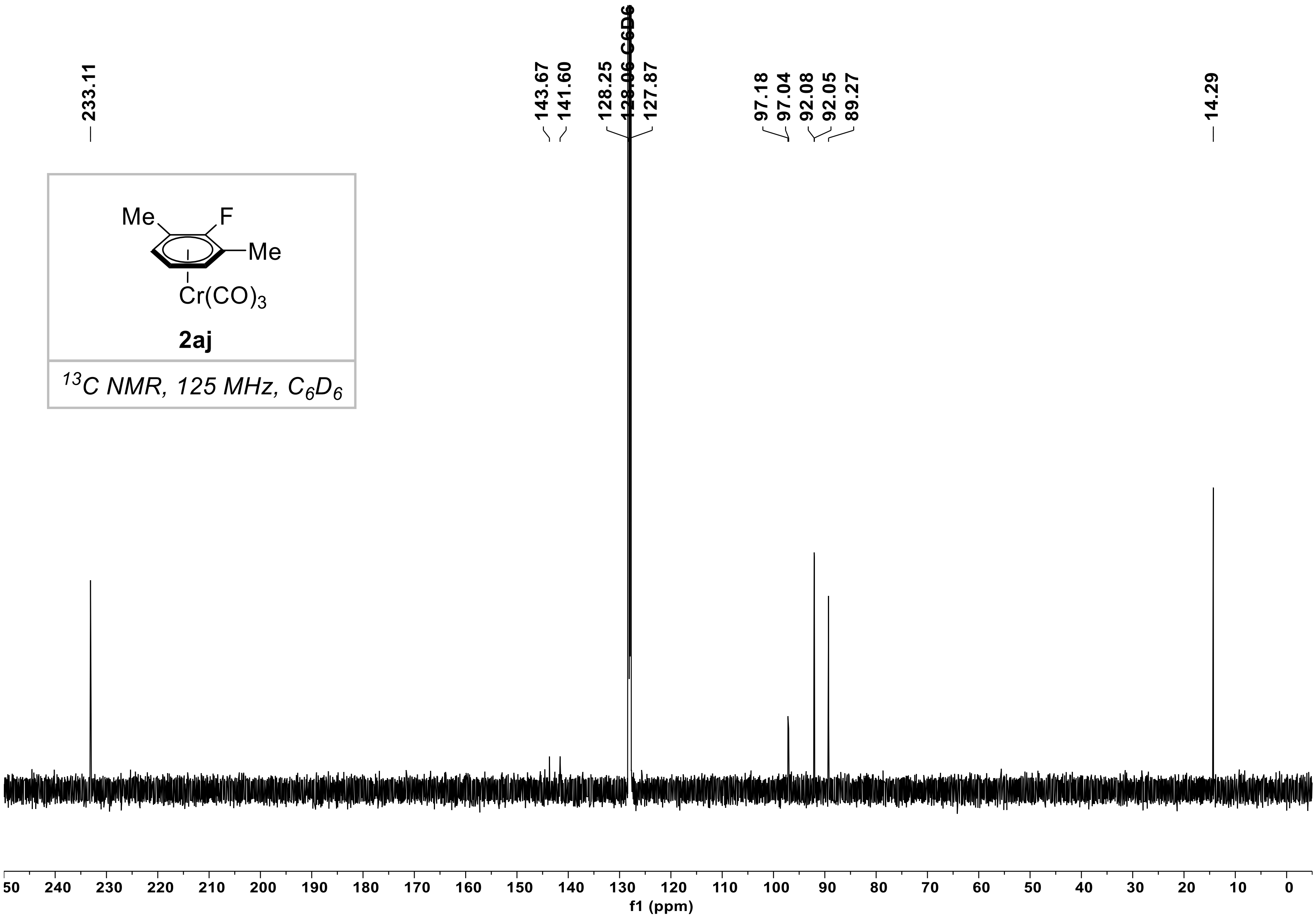
233.11
143.67
141.60
128.25
128.06 C6D6
127.87
97.18
97.04
92.08
92.05
89.27
14.29
Me
F
Me
Cr(CO)3
2aj
13C NMR, 125 MHz, C6D6
f1 (ppm)

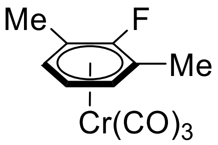

2aj

^{19}F NMR, 471 MHz, C_6D_6

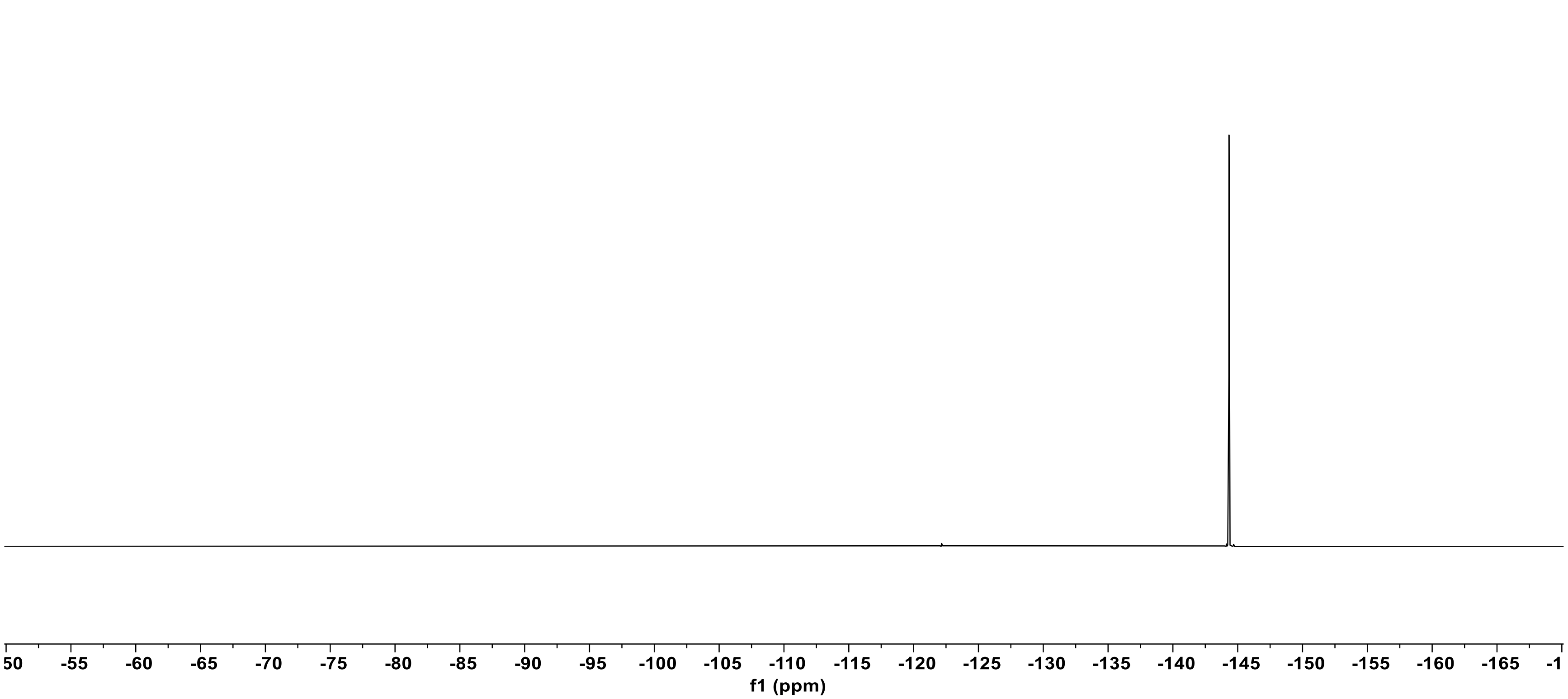

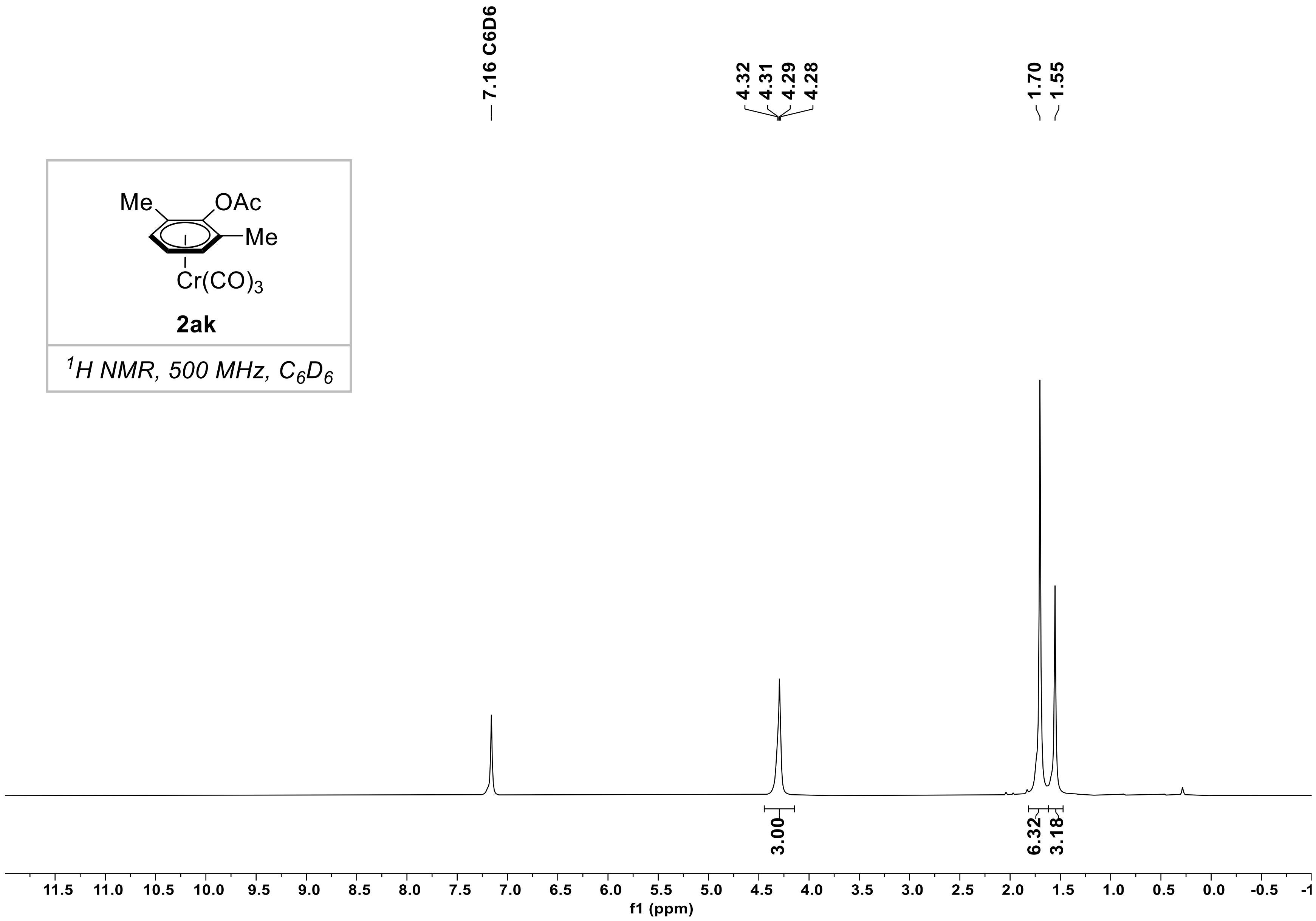
7.16 C6D6
4.32
4.31
4.29
4.28
1.70
1.55
Me
OAc
Me
Cr(CO)3
2ak
1H NMR, 500 MHz, C6D6
3.00
6.32
3.18
11.5 11.0 10.5 10.0 9.5 9.0 8.5 8.0 7.5 7.0 6.5 6.0 5.5 5.0 4.5 4.0 3.5 3.0 2.5 2.0 1.5 1.0 0.5 0.0 -0.5 -1
f1 (ppm)

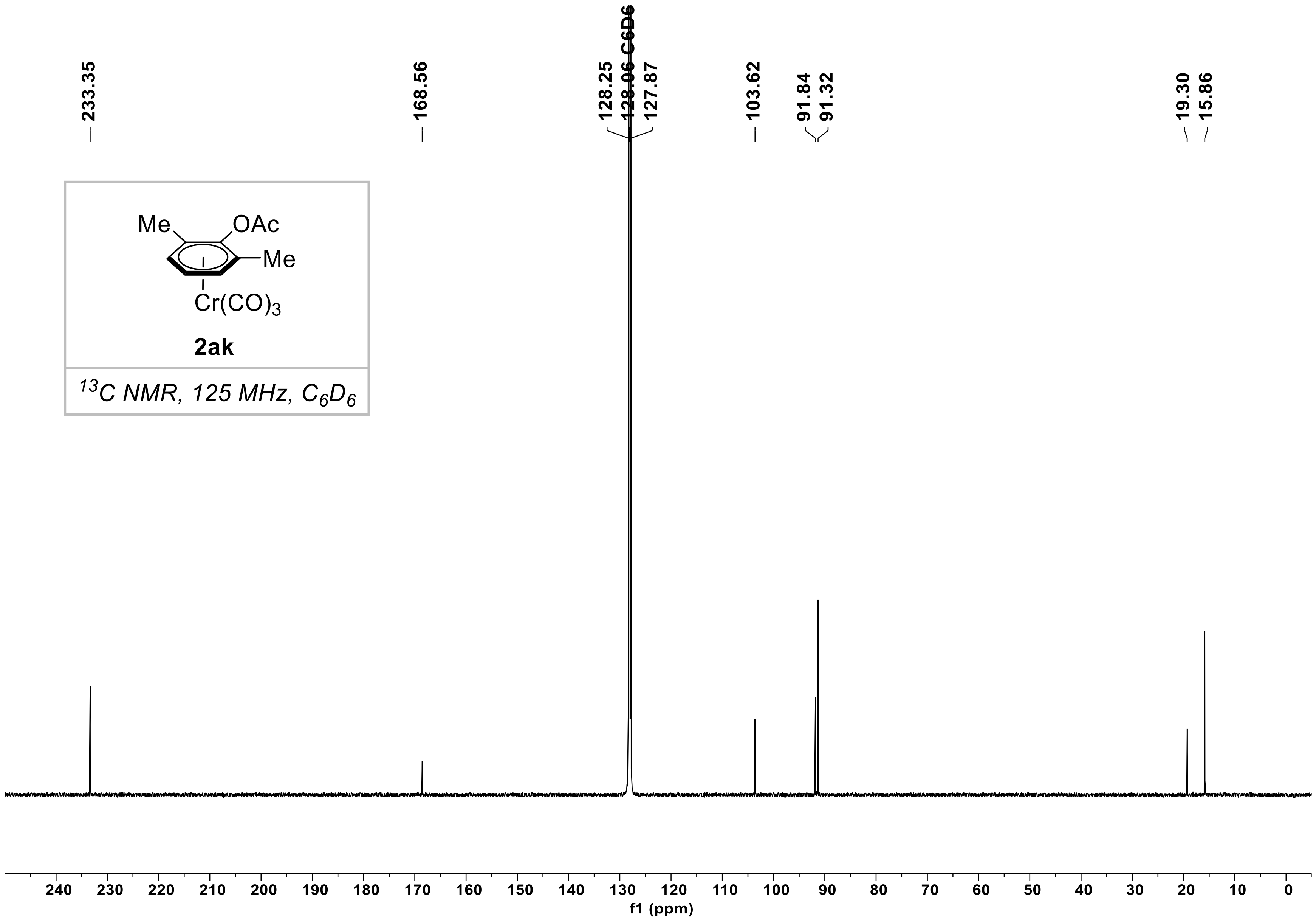
233.35
168.56
128.25
128.06 C6D6
127.87
103.62
91.84
91.32
19.30
15.86
Me
OAc
Me
Cr(CO)3
2ak
13C NMR, 125 MHz, C6D6
240 230 220 210 200 190 180 170 160 150 140 130 120 110 100 90 80 70 60 50 40 30 20 10 0
f1 (ppm)

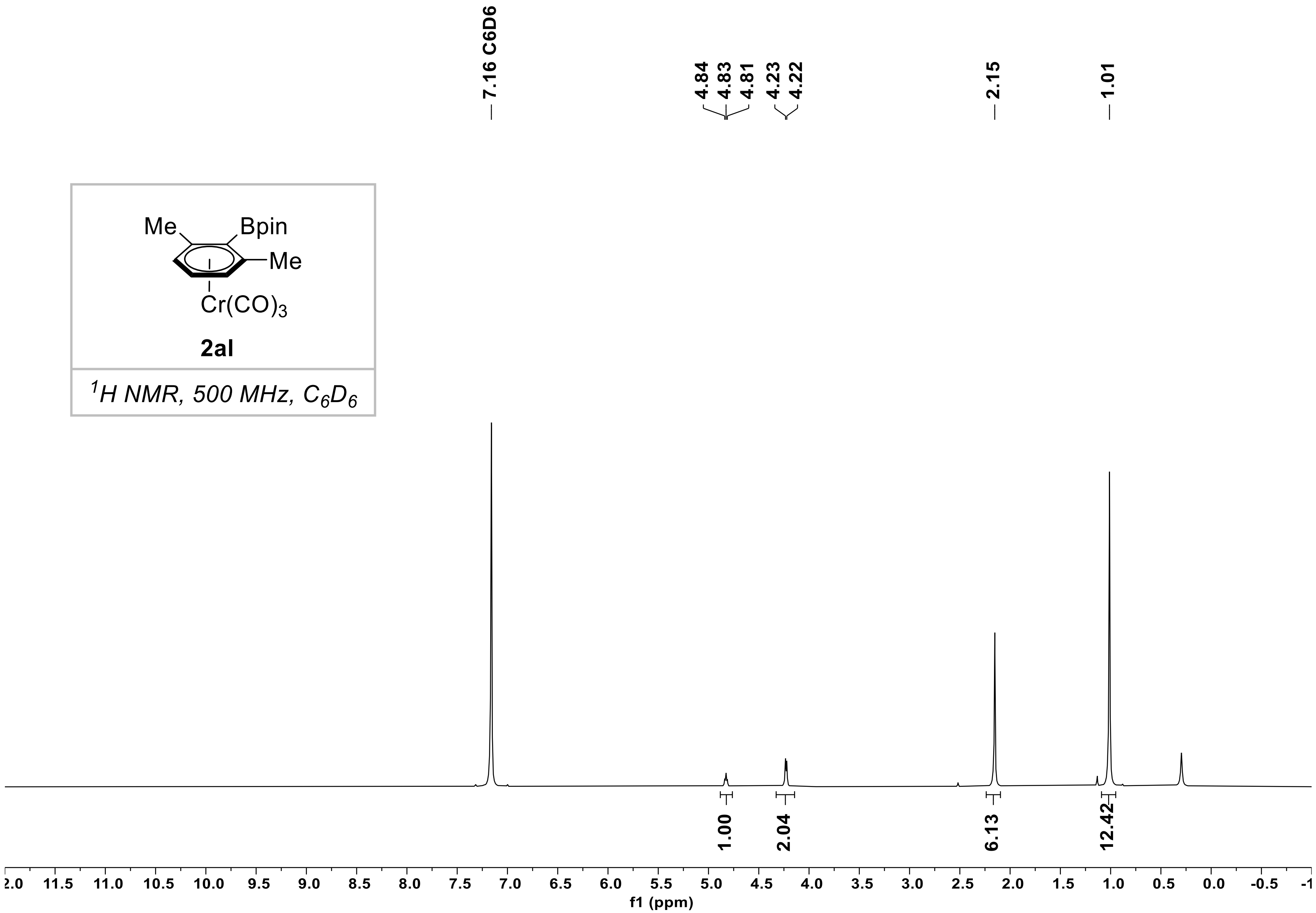
7.16 C6D6
4.84
4.83
4.81
4.23
4.22
2.15
1.01
Me
Bpin
Me
Cr(CO)3
2al
1H NMR, 500 MHz, C6D6
1.00
2.04
6.13
12.42
f1 (ppm)

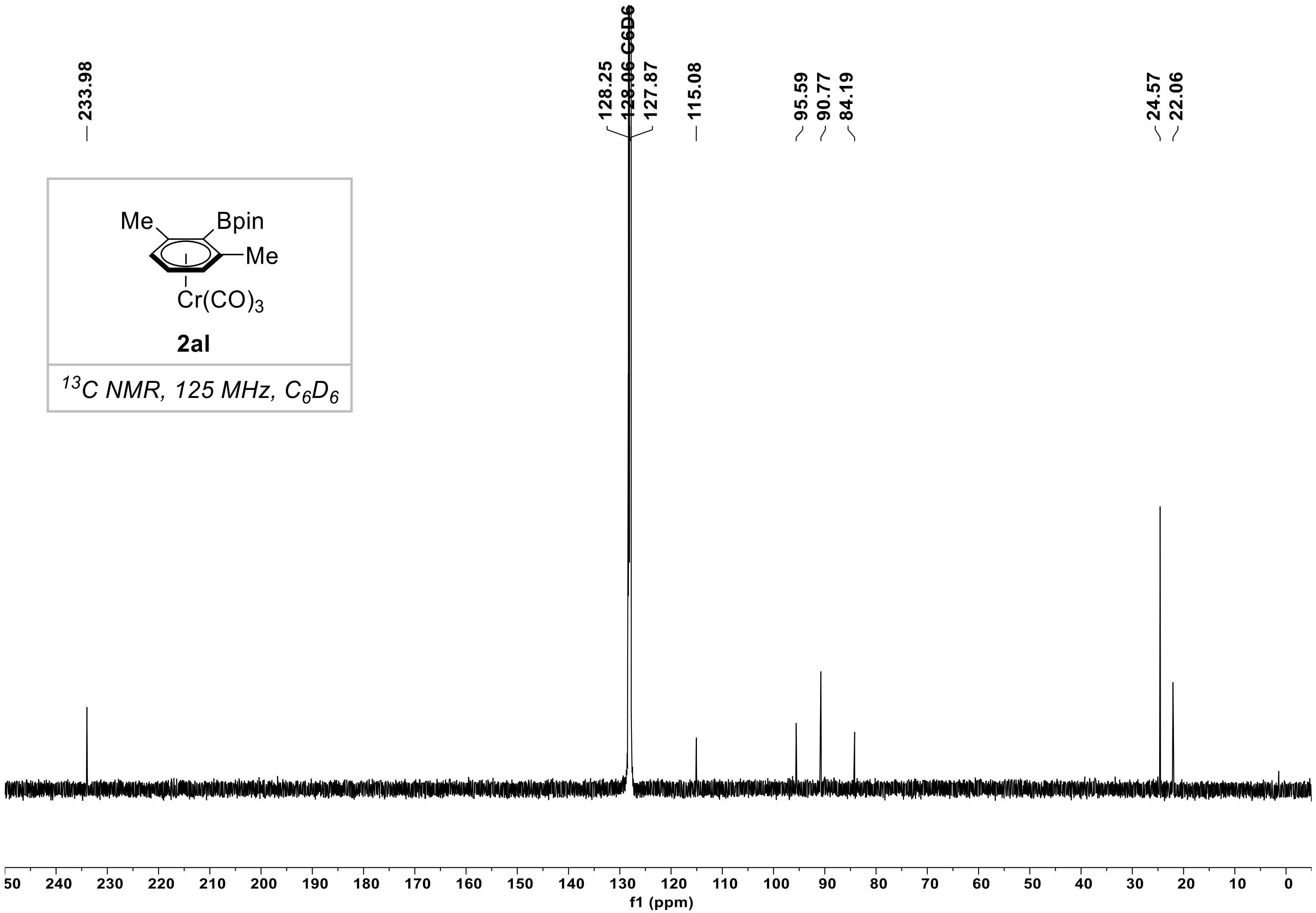
233.98
128.25
128.06 C6D6
127.87
115.08
95.59
90.77
84.19
24.57
22.06
Me
Bpin
Me
Cr(CO)3
2al
13C NMR, 125 MHz, C6D6
250
240
230
220
210
200
190
180
170
160
150
140
130
120
110
100
90
80
70
60
50
40
30
20
10
0
f1 (ppm)

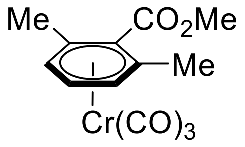

2am

^{1}H NMR, 400 MHz, C_6D_6

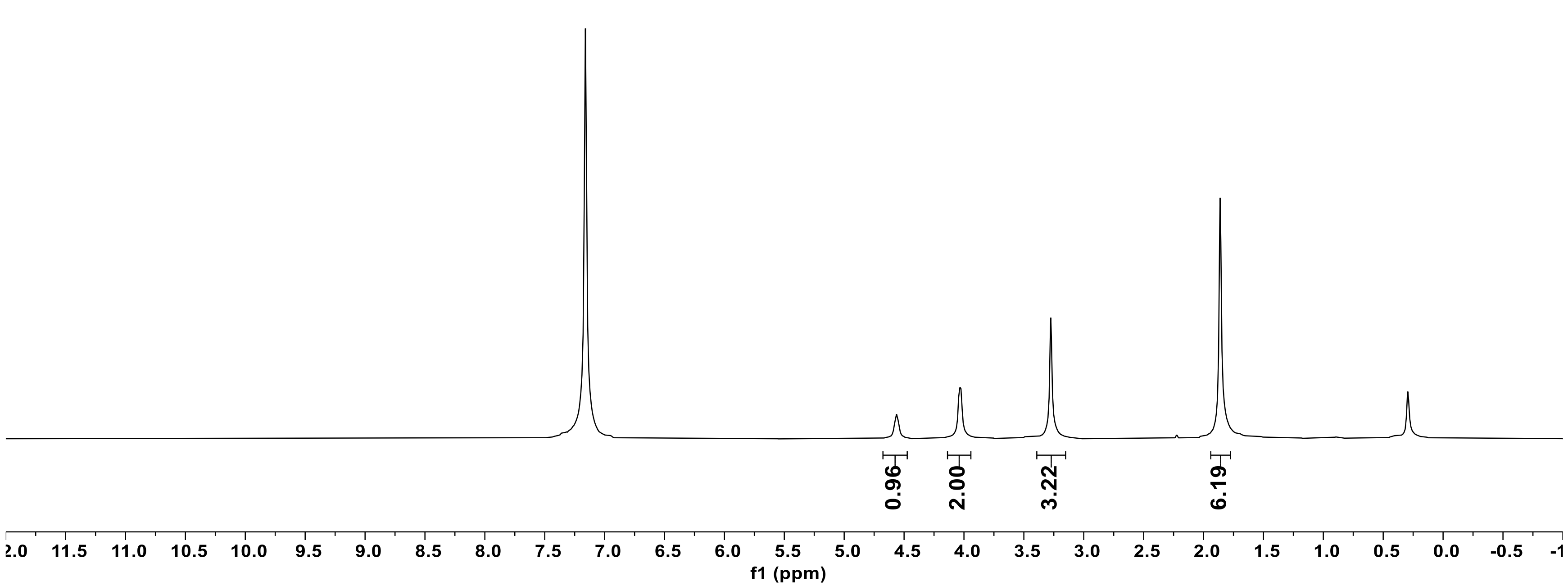

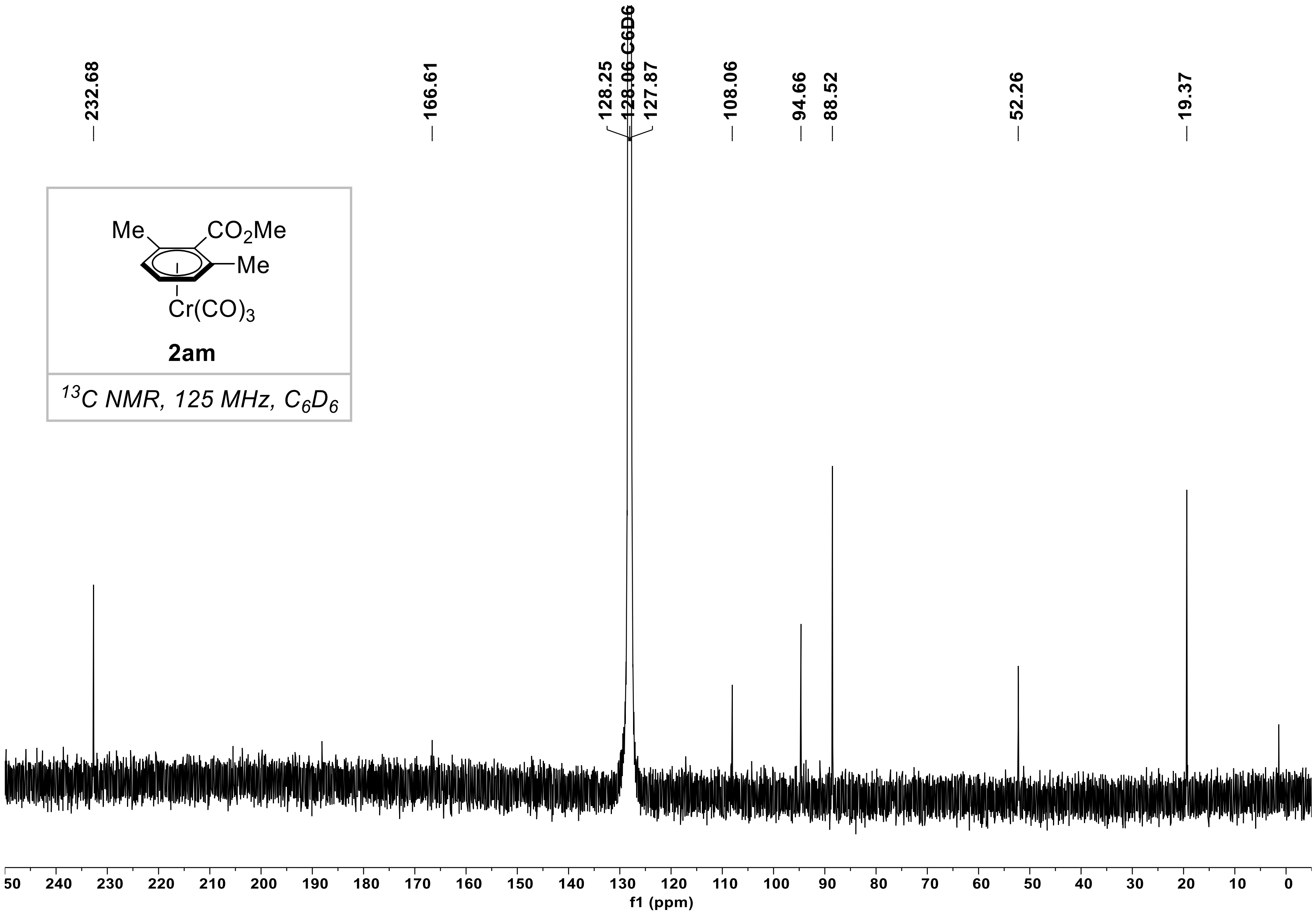
232.68
166.61
128.25
128.06 C6D6
127.87
108.06
94.66
88.52
52.26
19.37
Me
CO2Me
Me
Cr(CO)3
2am
13C NMR, 125 MHz, C6D6
f1 (ppm)

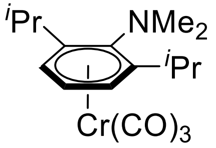

2an

^1H NMR, 400 MHz, C_6D_6

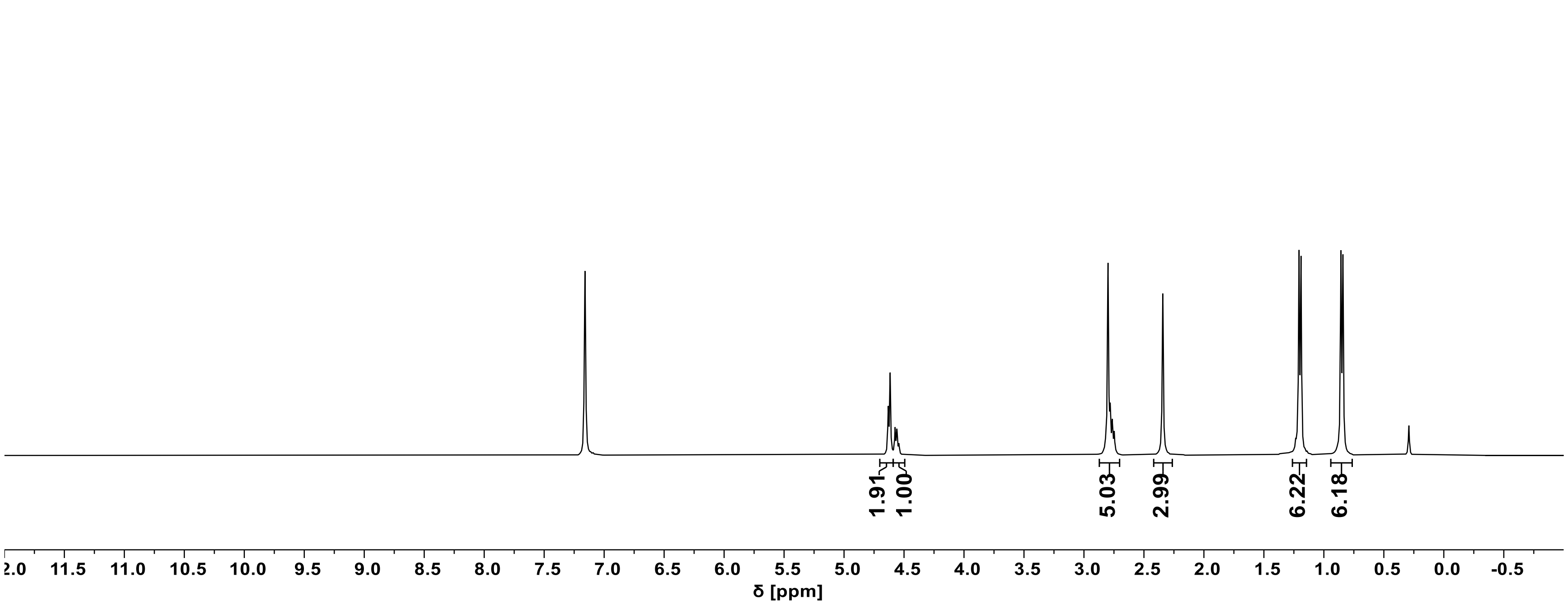

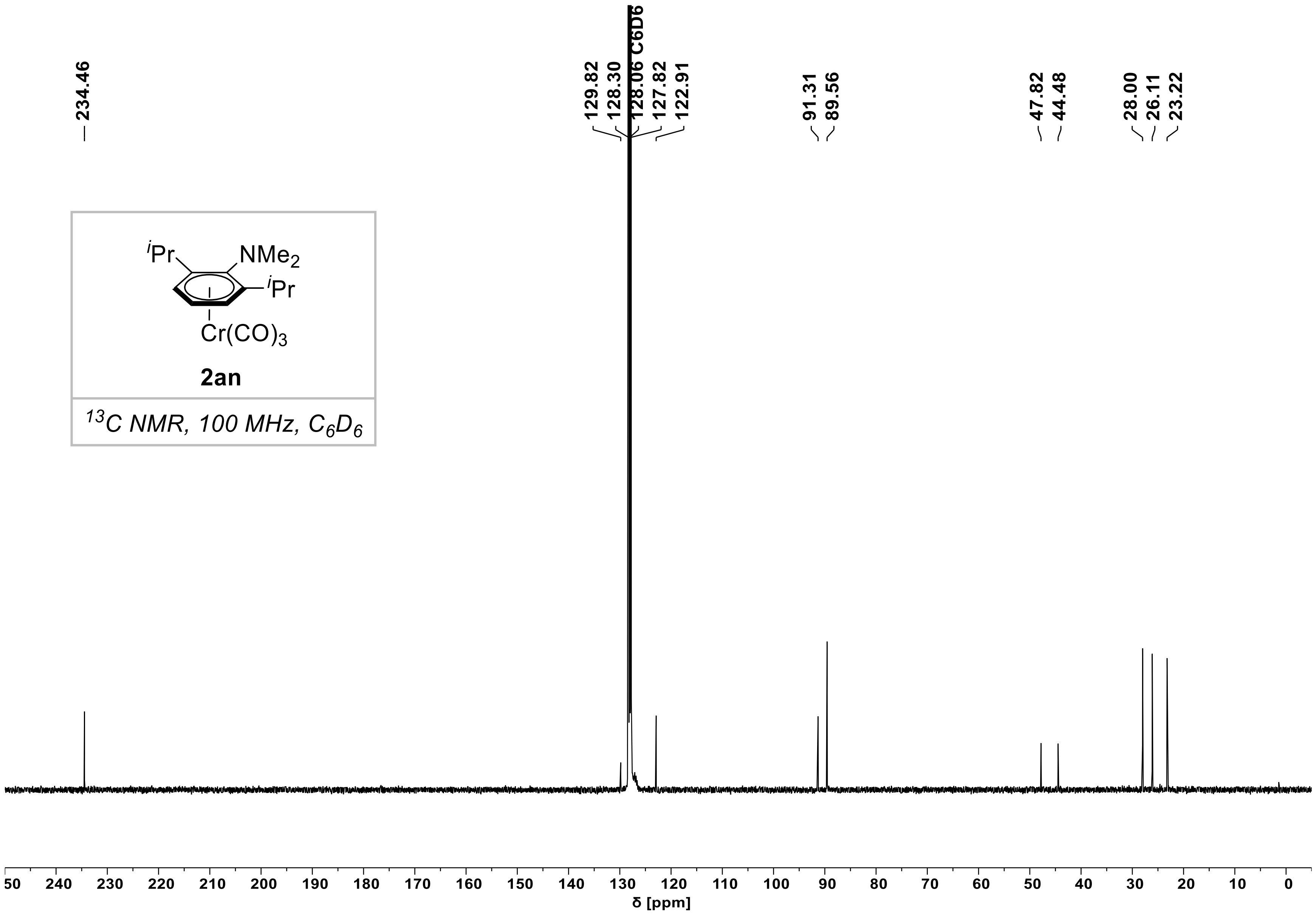

2an

^{13}C NMR, 100 MHz, C_6D_6

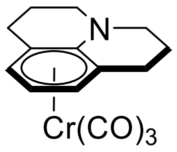

2ao

^{1}H NMR, 500 MHz, C_6D_6

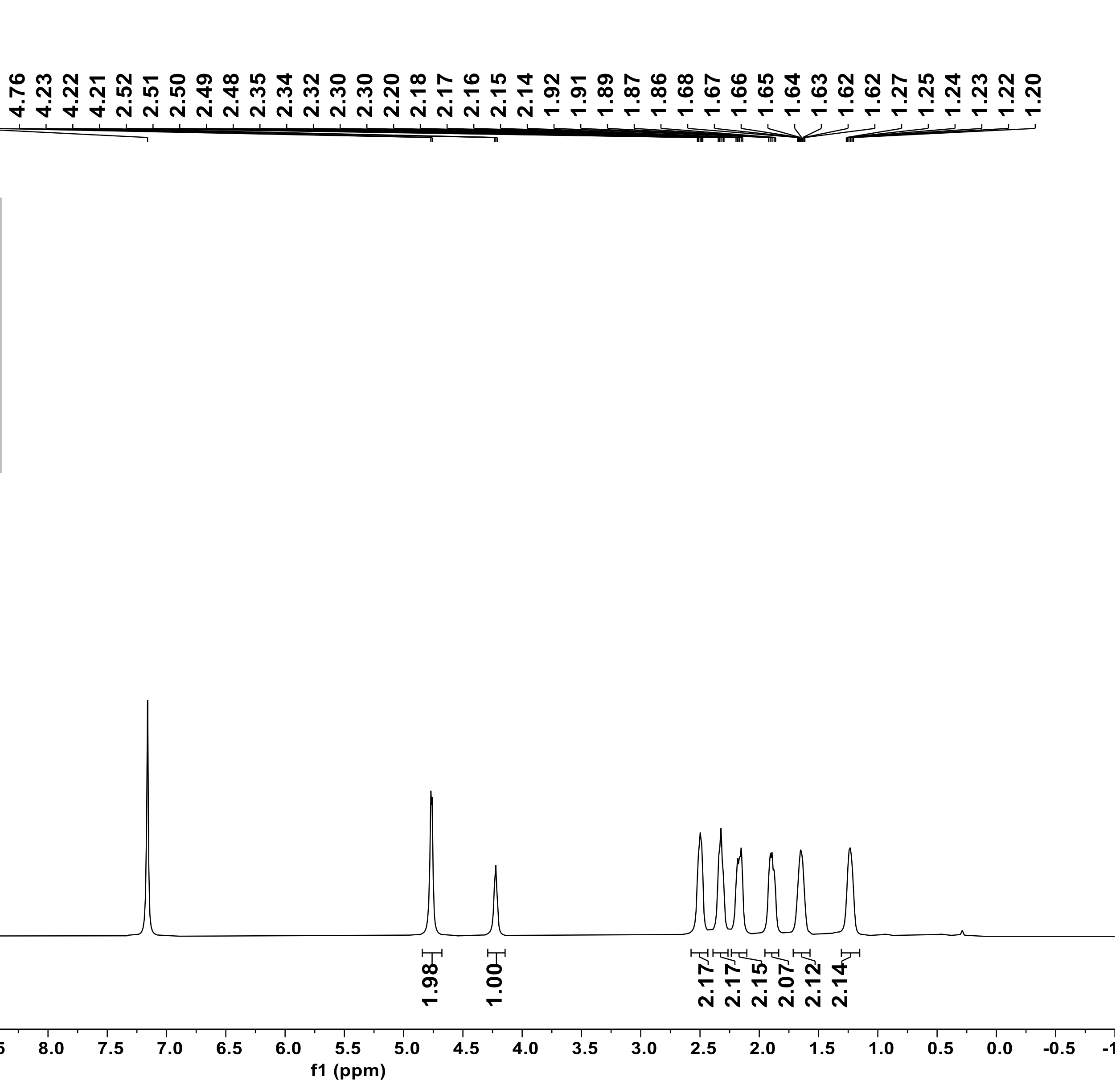

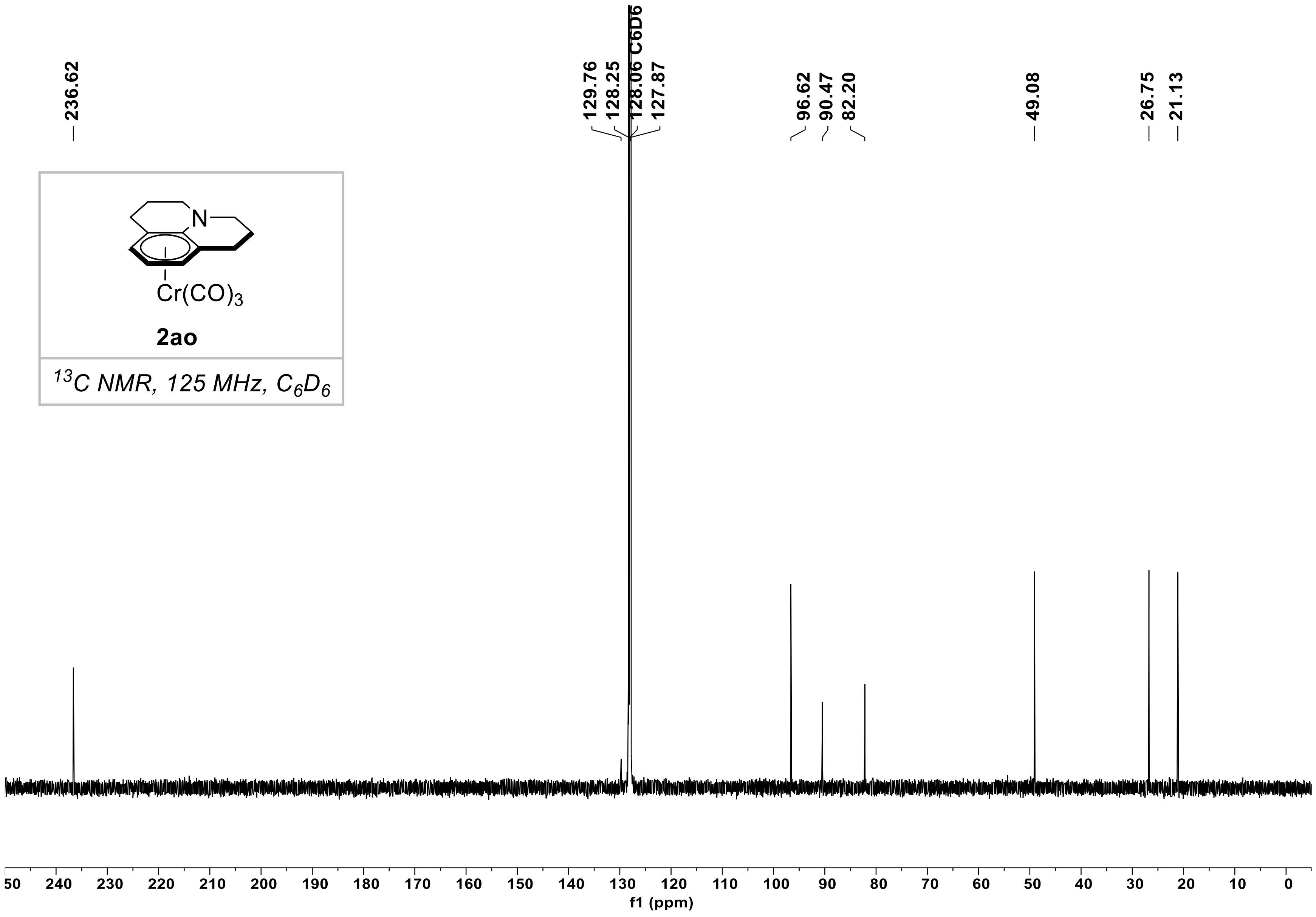
236.62
129.76
128.25
128.06 C6D6
127.87
96.62
90.47
82.20
49.08
26.75
21.13
N
Cr(CO)3
2ao
13C NMR, 125 MHz, C6D6
50
240
230
220
210
200
190
180
170
160
150
140
130
120
110
100
90
80
70
60
50
40
30
20
10
0
f1 (ppm)

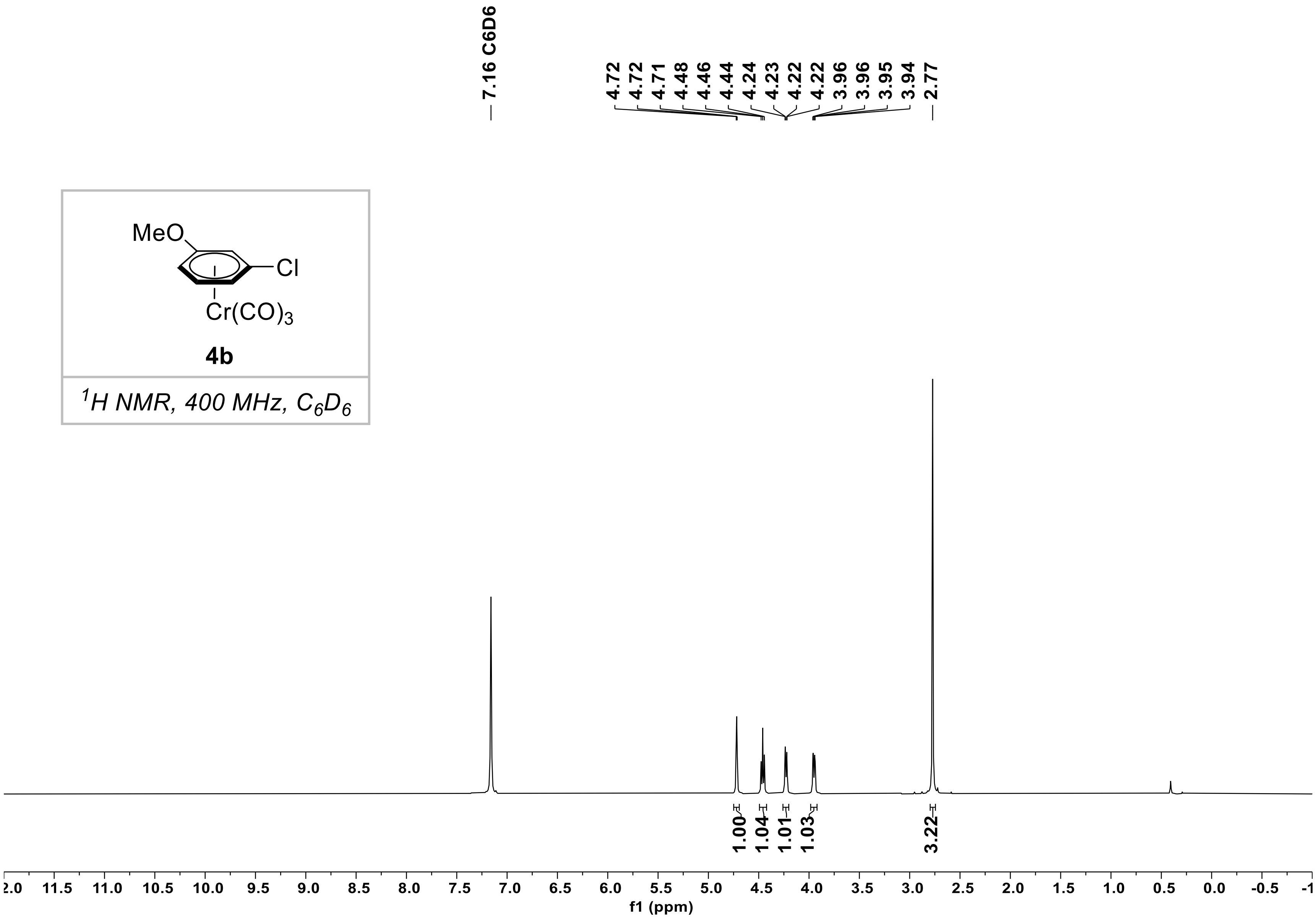

7.16 C6D6
4.72
4.72
4.71
4.48
4.46
4.44
4.24
4.23
4.22
4.22
3.96
3.96
3.95
3.94
2.77
MeO
Cl
Cr(CO)3
4b
1H NMR, 400 MHz, C6D6
1.00
1.04
1.01
1.03
3.22
f1 (ppm)

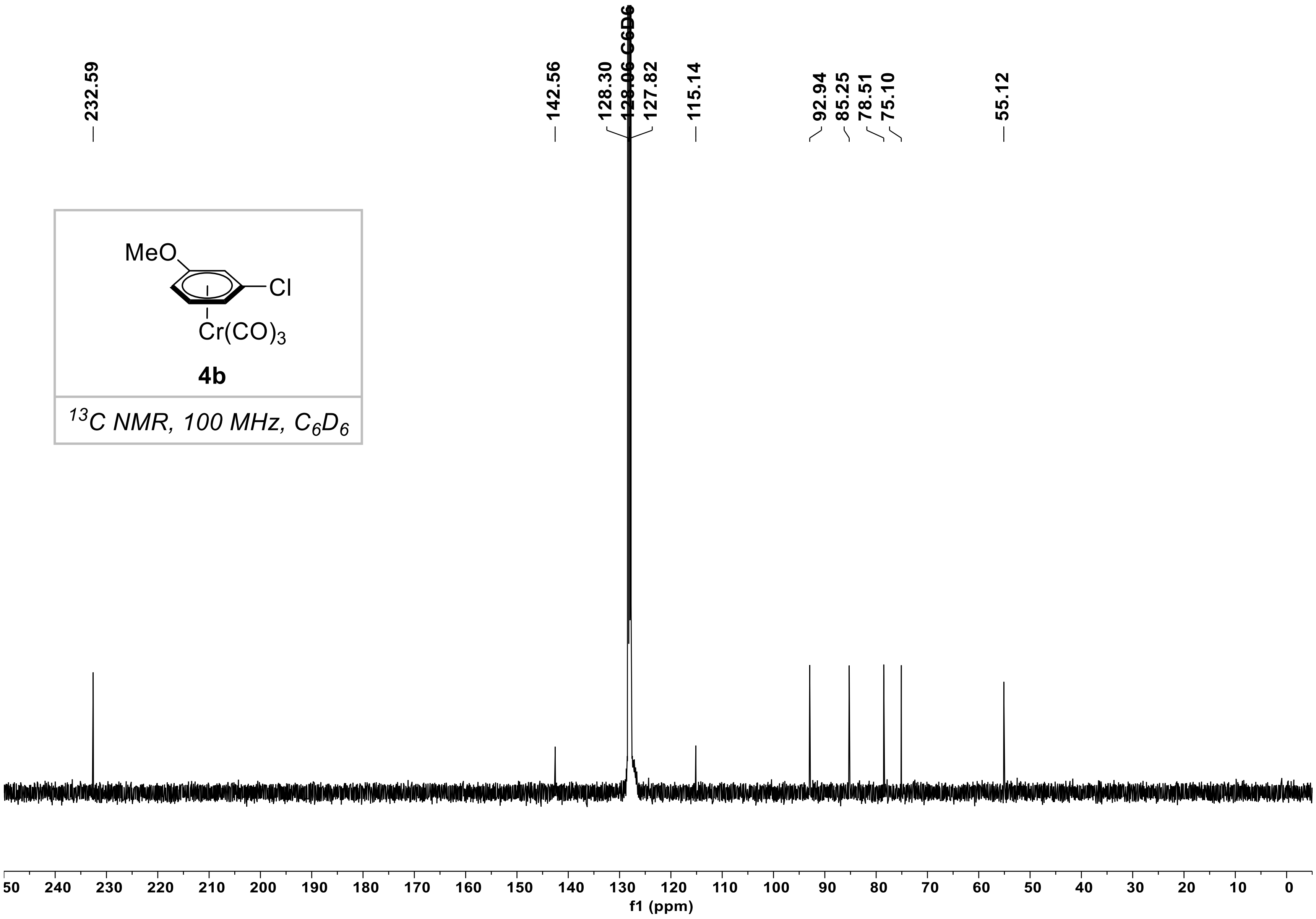
232.59
142.56
128.30
128.06 C6D6
127.82
115.14
92.94
85.25
78.51
75.10
55.12
MeO
Cl
Cr(CO)3
4b
13C NMR, 100 MHz, C6D6
50
240
230
220
210
200
190
180
170
160
150
140
130
120
110
100
90
80
70
60
50
40
30
20
10
0
f1 (ppm)

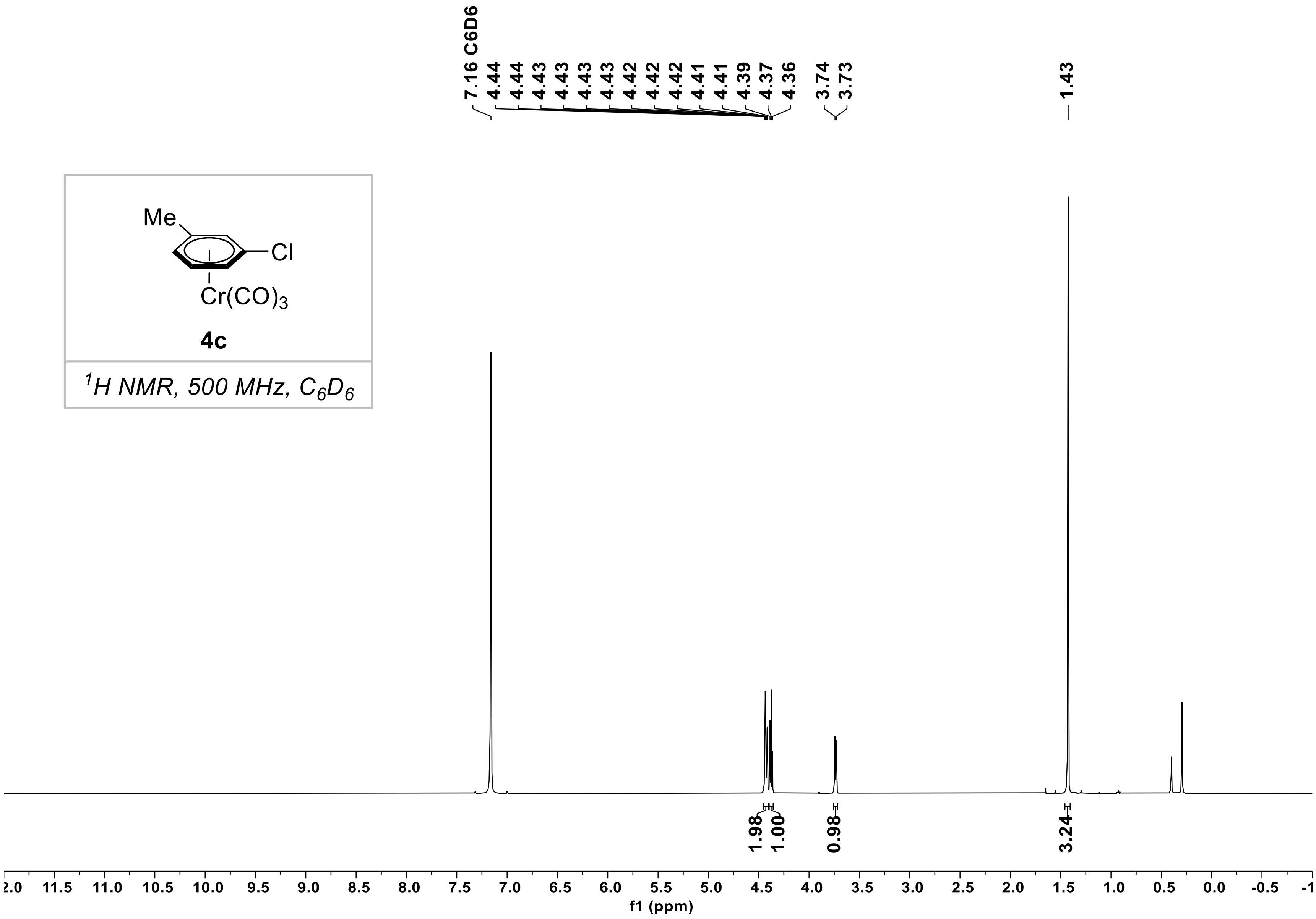
7.16 C6D6
4.44
4.44
4.43
4.43
4.43
4.43
4.42
4.42
4.42
4.41
4.41
4.39
4.37
4.36
3.74
3.73
1.43
Me
Cl
Cr(CO)3
4c
1H NMR, 500 MHz, C6D6
1.98
1.00
0.98
3.24
f1 (ppm)

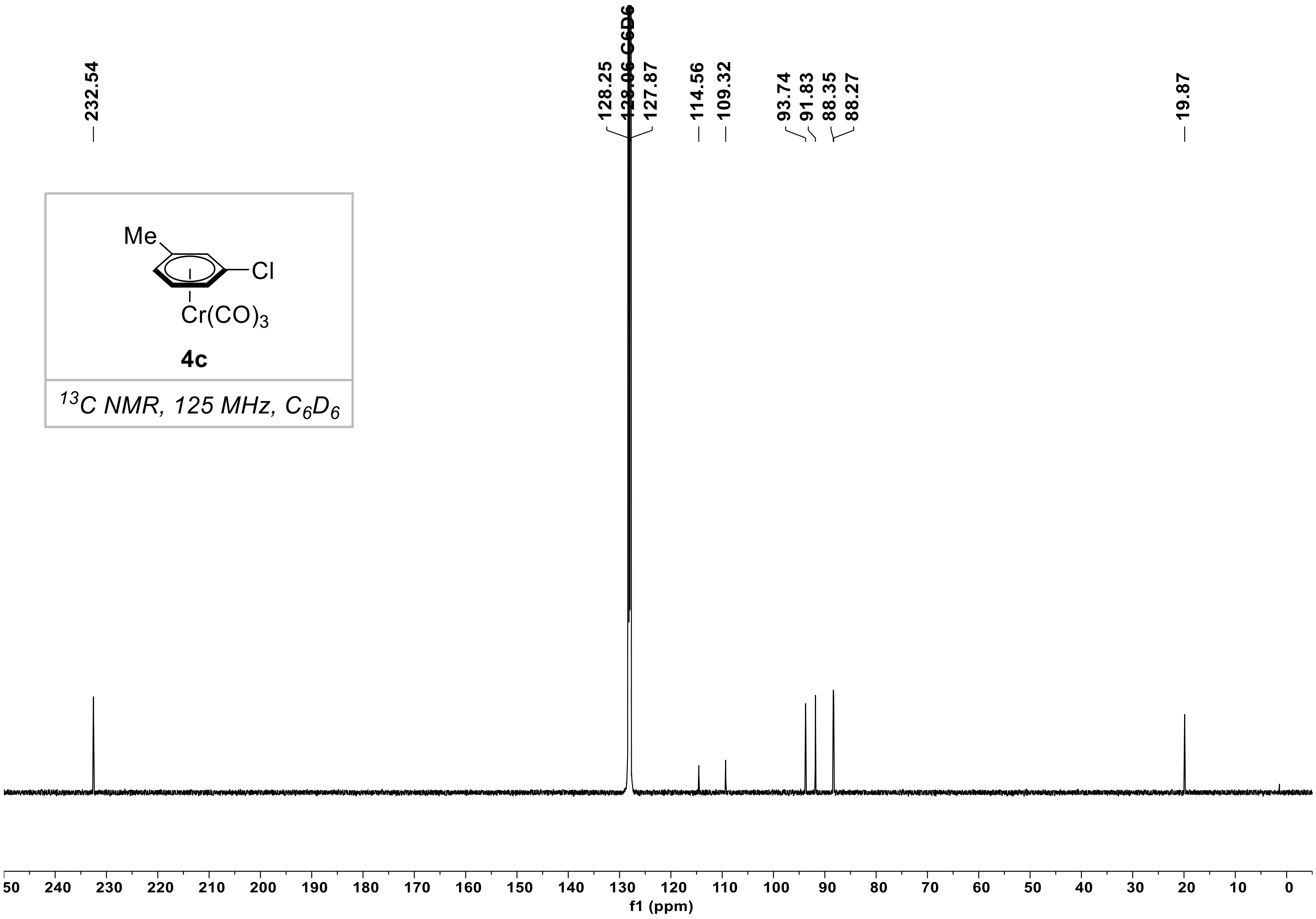
232.54
128.25
128.06
C6D6
127.87
114.56
109.32
93.74
91.83
88.35
88.27
19.87
Me
Cl
Cr(CO)3
4c
13C NMR, 125 MHz, C6D6
250
240
230
220
210
200
190
180
170
160
150
140
130
120
110
100
90
80
70
60
50
40
30
20
10
0
f1 (ppm)

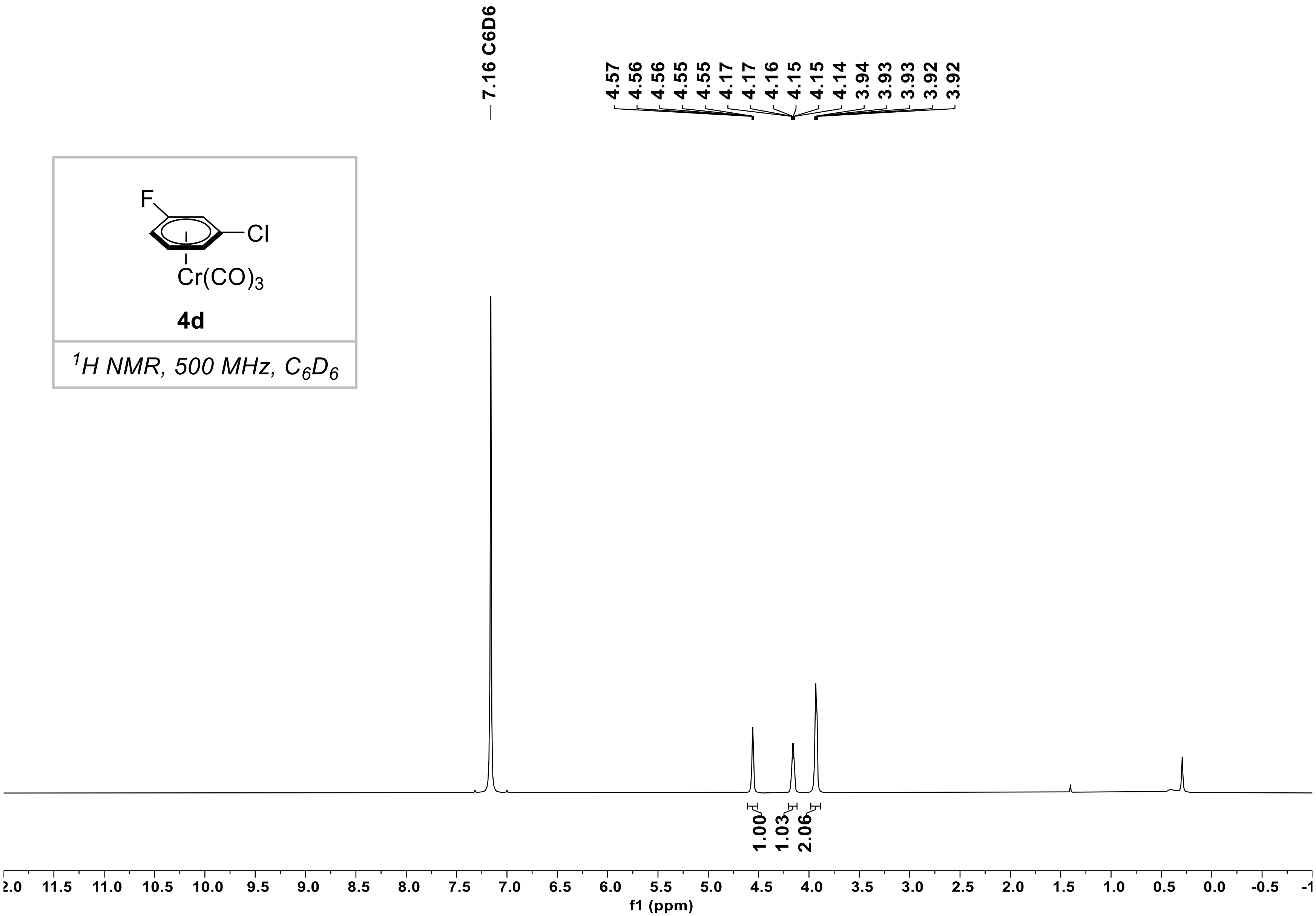
F
Cl
Cr(CO)3
4d
1H NMR, 500 MHz, C6D6
7.16 C6D6
4.57
4.56
4.56
4.55
4.55
4.17
4.17
4.16
4.15
4.15
4.14
3.94
3.93
3.93
3.92
3.92
1.00
1.03
2.06
2.0
11.5
11.0
10.5
10.0
9.5
9.0
8.5
8.0
7.5
7.0
6.5
6.0
5.5
5.0
4.5
4.0
3.5
3.0
2.5
2.0
1.5
1.0
0.5
0.0
-0.5
-1
f1 (ppm)

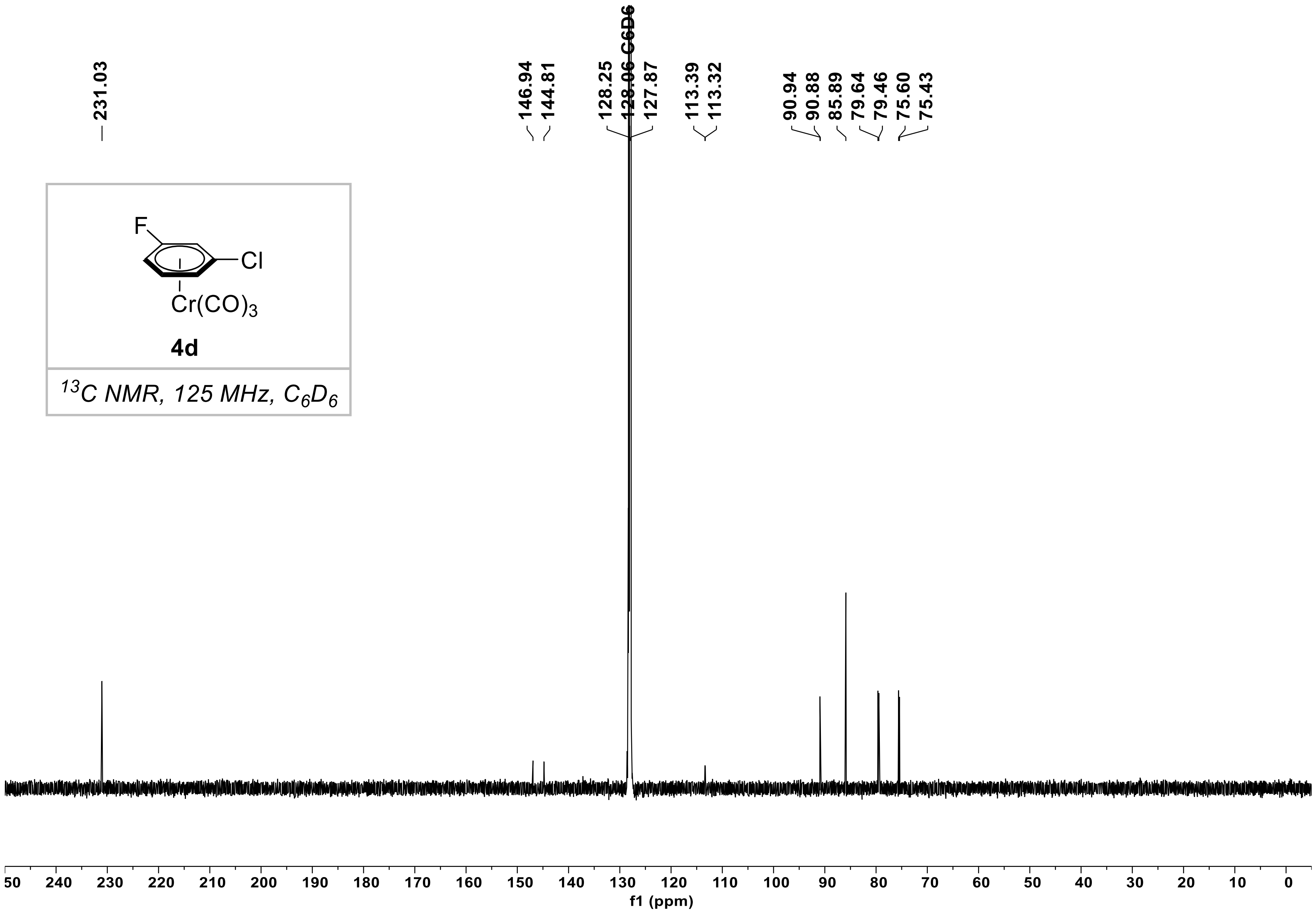
231.03
146.94
144.81
128.25
128.06 C6D6
127.87
113.39
113.32
90.94
90.88
85.89
79.64
79.46
75.60
75.43
F
Cl
Cr(CO)3
4d
13C NMR, 125 MHz, C6D6
250 240 230 220 210 200 190 180 170 160 150 140 130 120 110 100 90 80 70 60 50 40 30 20 10 0
f1 (ppm)

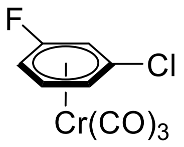

4d

^{19}F NMR, 471 MHz, C_6D_6

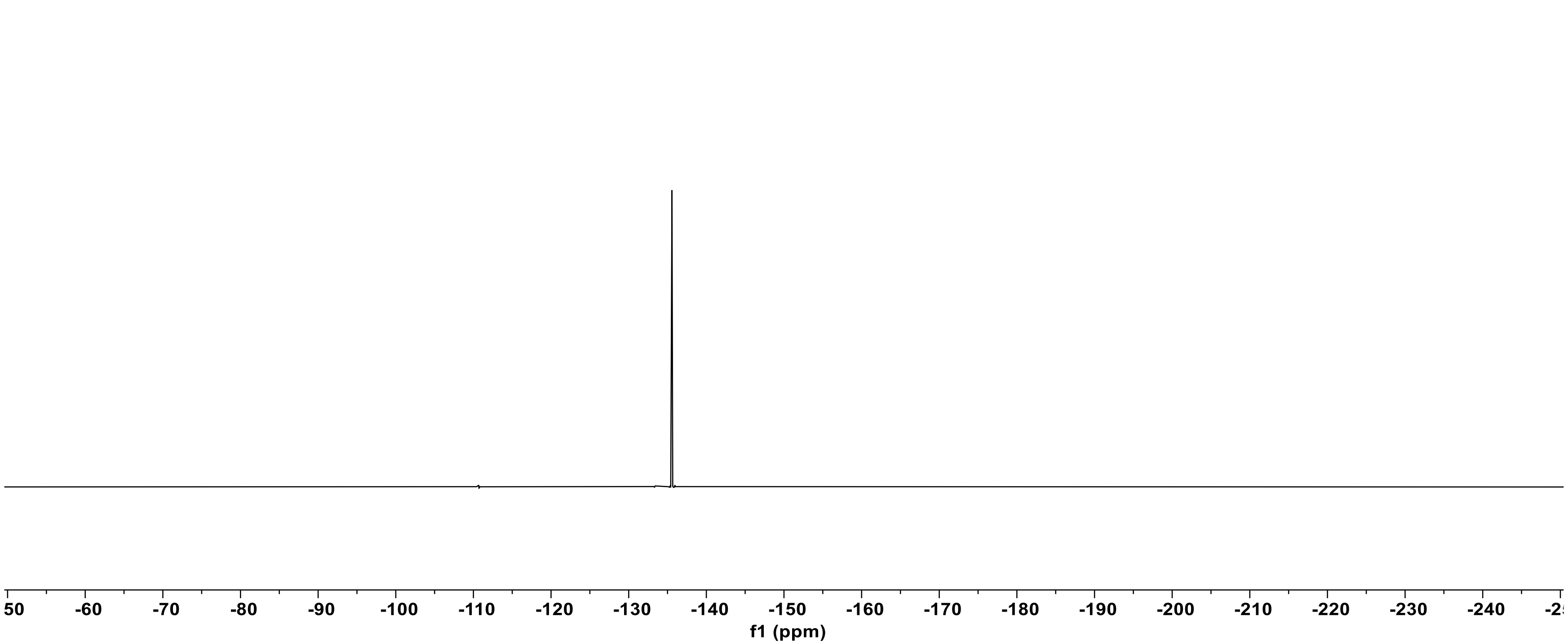

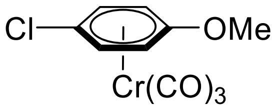

4e

^{1}H NMR, 500 MHz, C_6D_6

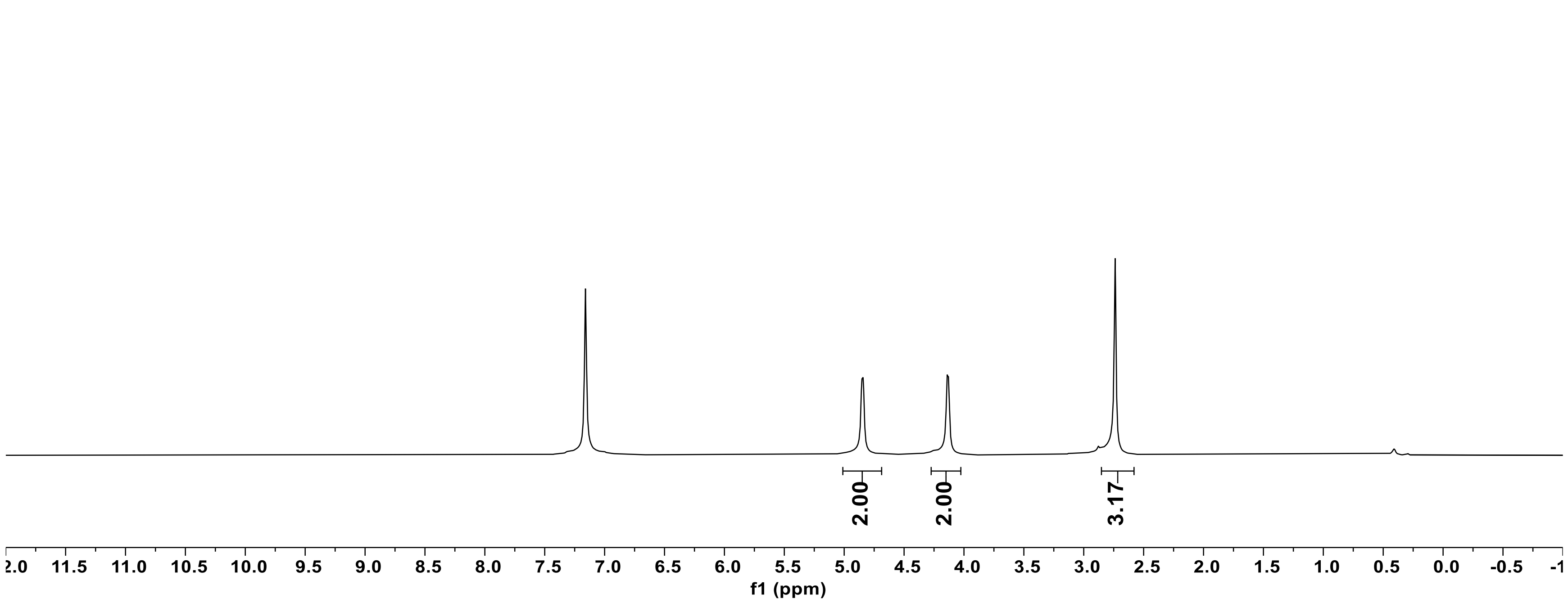

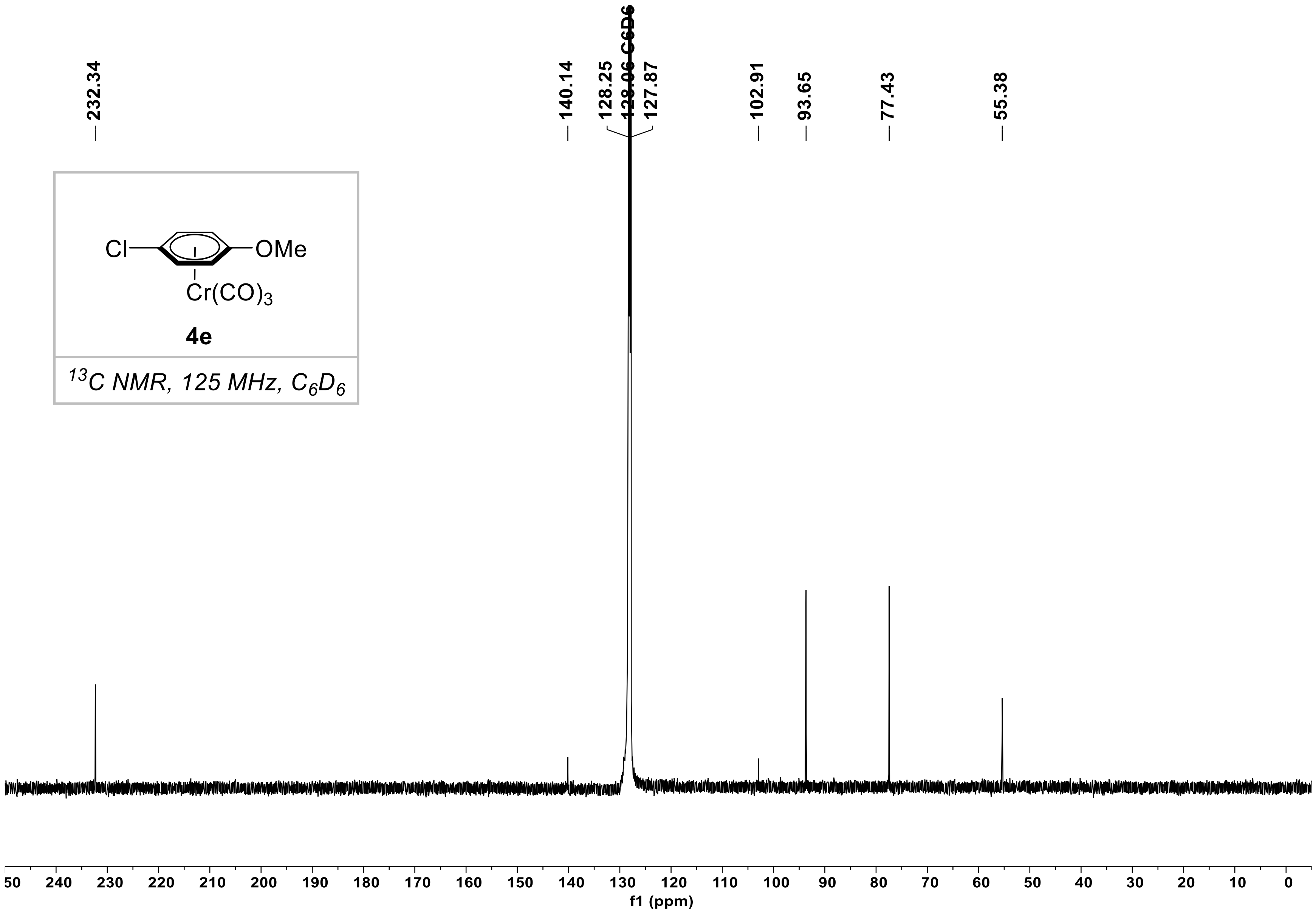
232.34
140.14
128.25
128.06 C6D6
127.87
102.91
93.65
77.43
55.38
Cl
OMe
Cr(CO)3
4e
13C NMR, 125 MHz, C6D6
50 240 230 220 210 200 190 180 170 160 150 140 130 120 110 100 90 80 70 60 50 40 30 20 10 0
f1 (ppm)

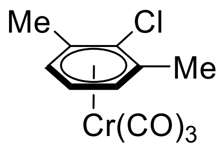

4f

^{1}H NMR, 400 MHz, C_6D_6

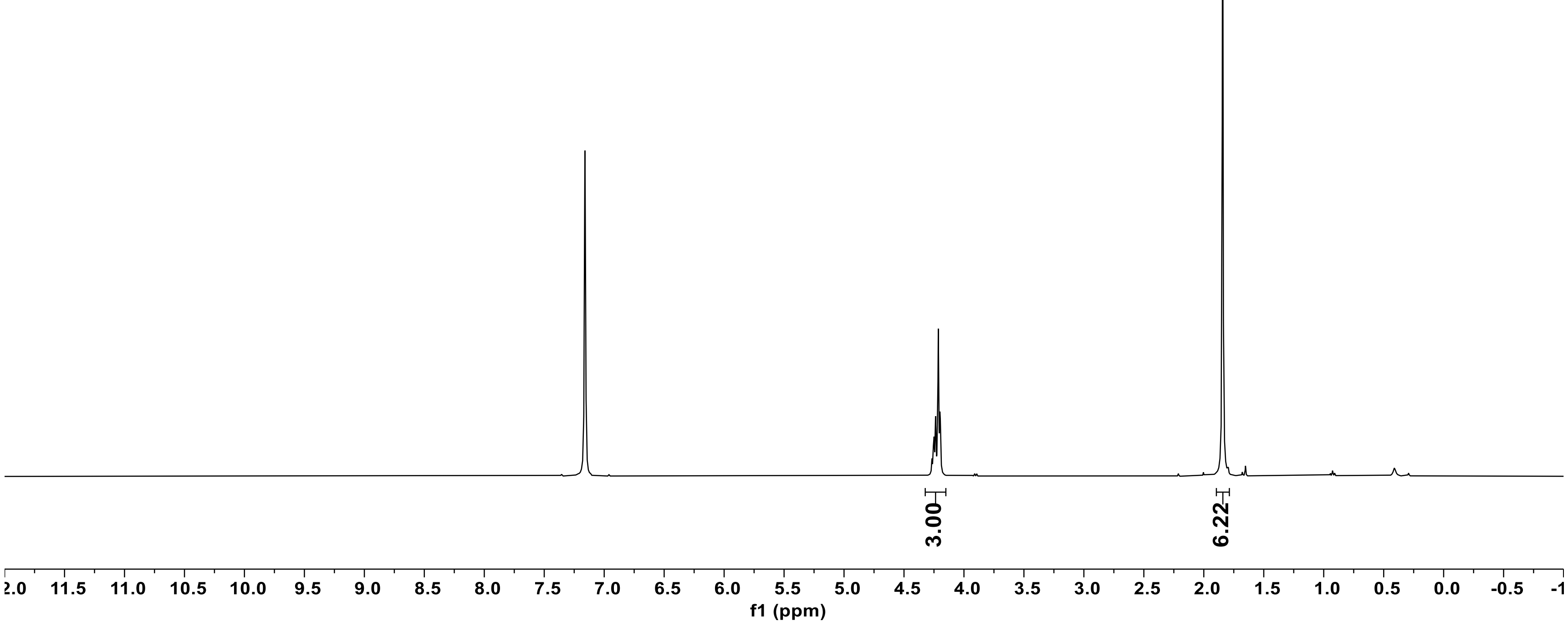

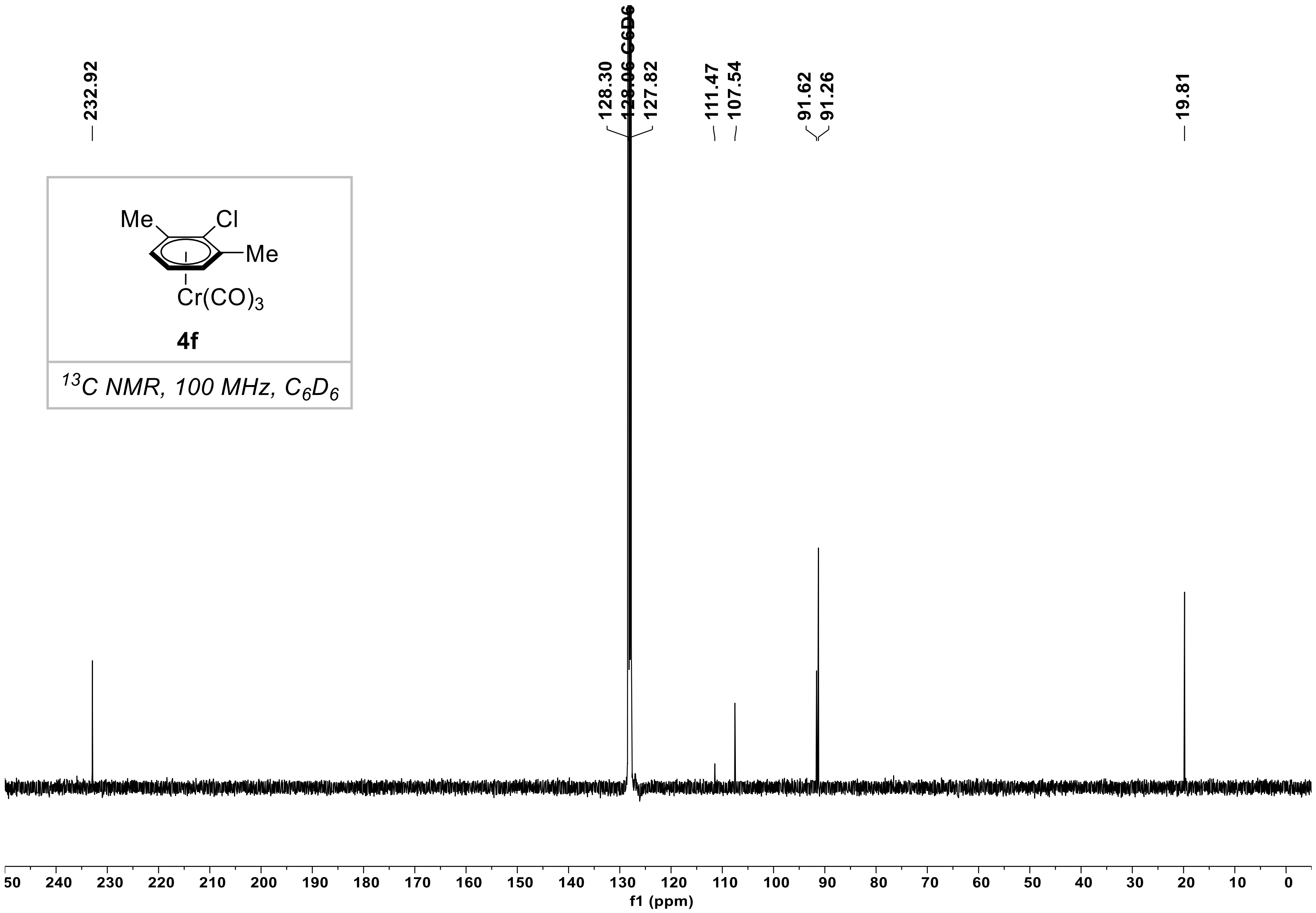

232.92
128.30
128.06 C6D6
127.82
111.47
107.54
91.62
91.26
19.81
Me
Cl
Me
Cr(CO)3
4f
13C NMR, 100 MHz, C6D6
50 240 230 220 210 200 190 180 170 160 150 140 130 120 110 100 90 80 70 60 50 40 30 20 10 0
f1 (ppm)

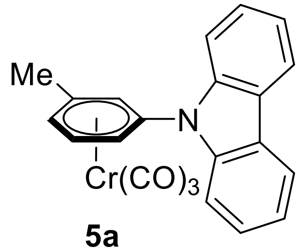

5a

^{1}H NMR, 400 MHz, $(CD_3)_2CO$

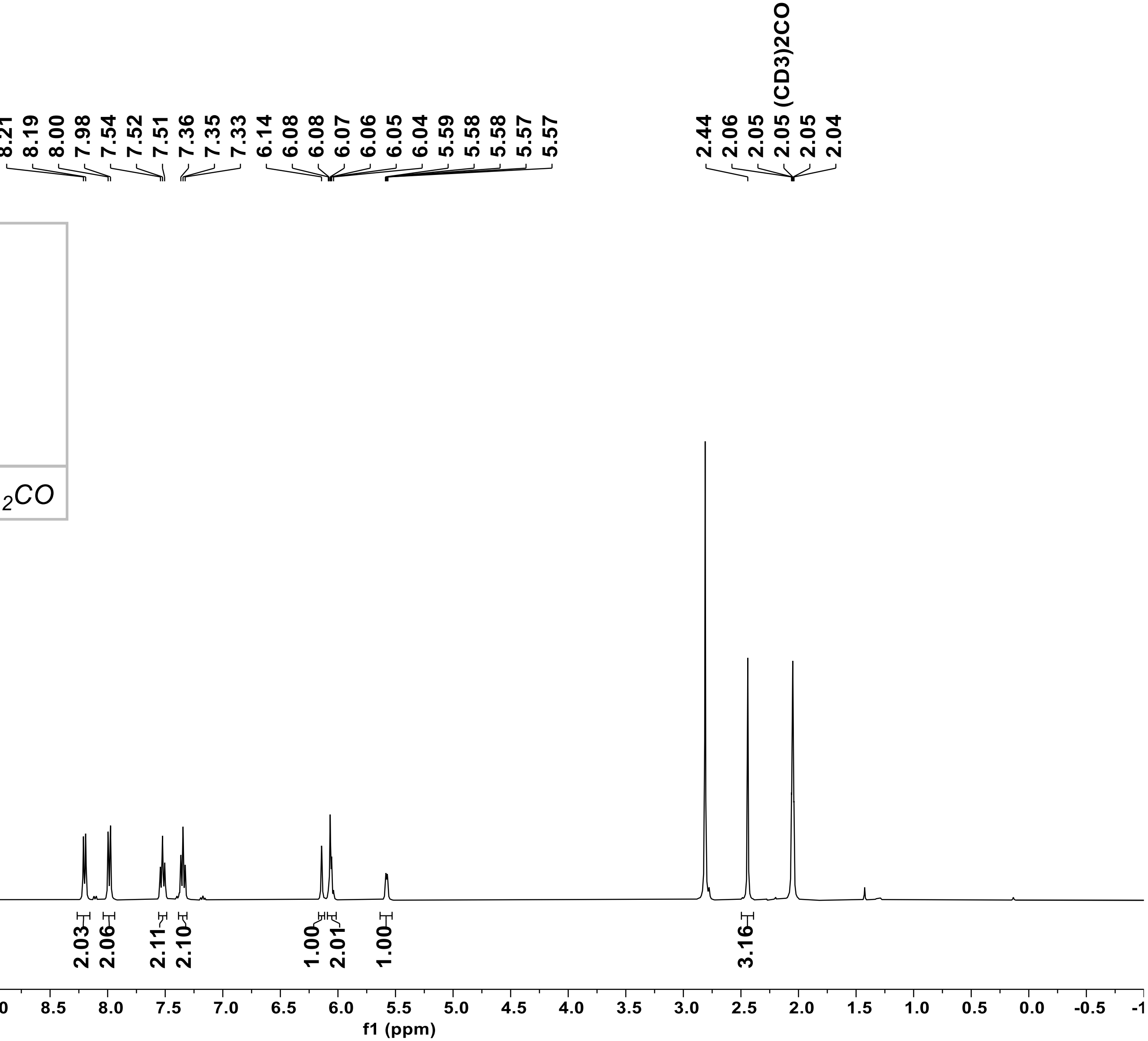

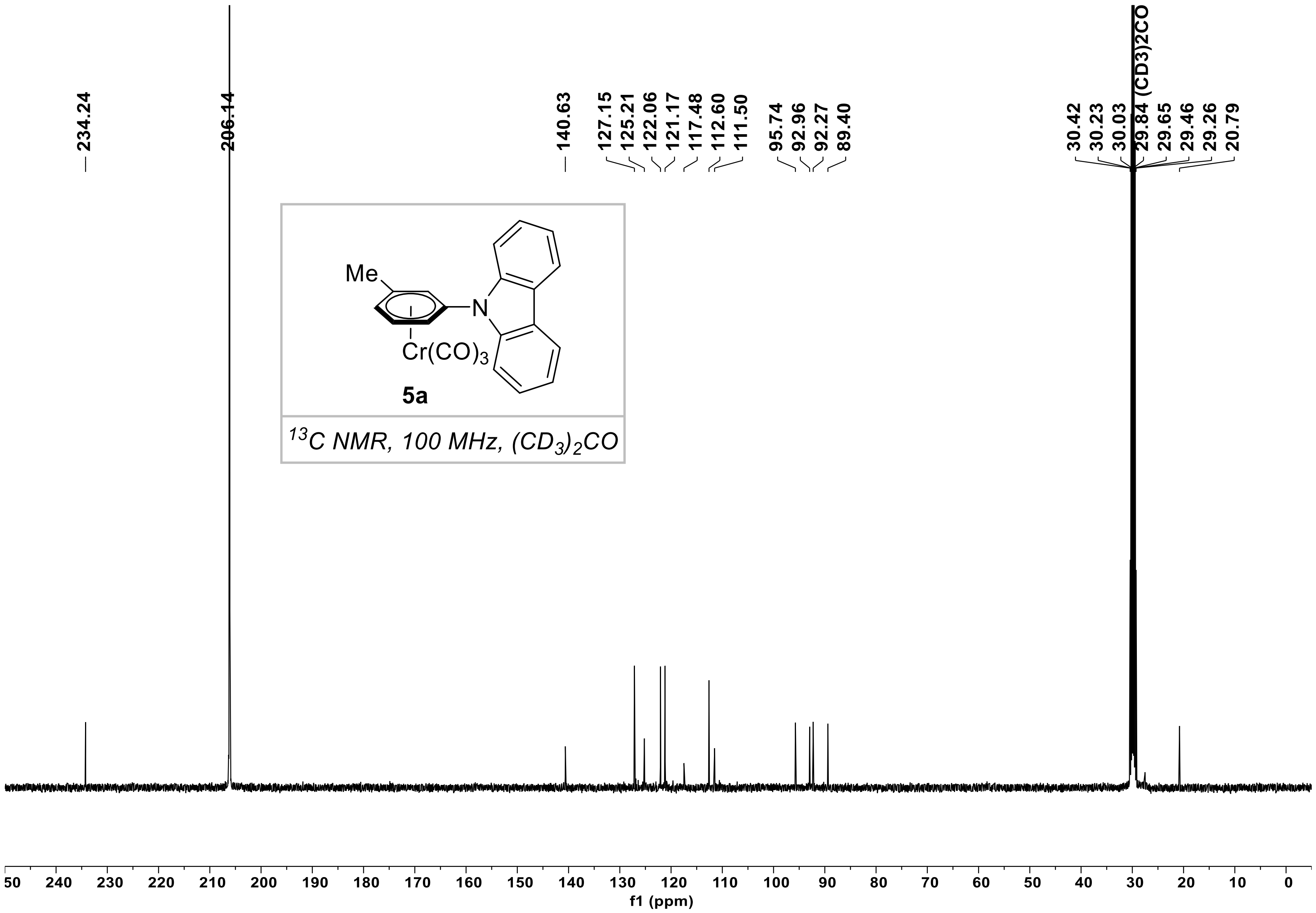

234.24
206.14
140.63
127.15
125.21
122.06
121.17
117.48
112.60
111.50
95.74
92.96
92.27
89.40
30.42
30.23
30.03
29.84 (CD3)2CO
29.65
29.46
29.26
20.79
Me
N
Cr(CO)3
5a
13C NMR, 100 MHz, (CD3)2CO
f1 (ppm)

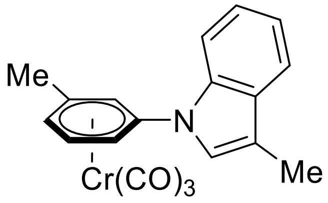

5b

¹H NMR, 500 MHz, (CD3)2CO

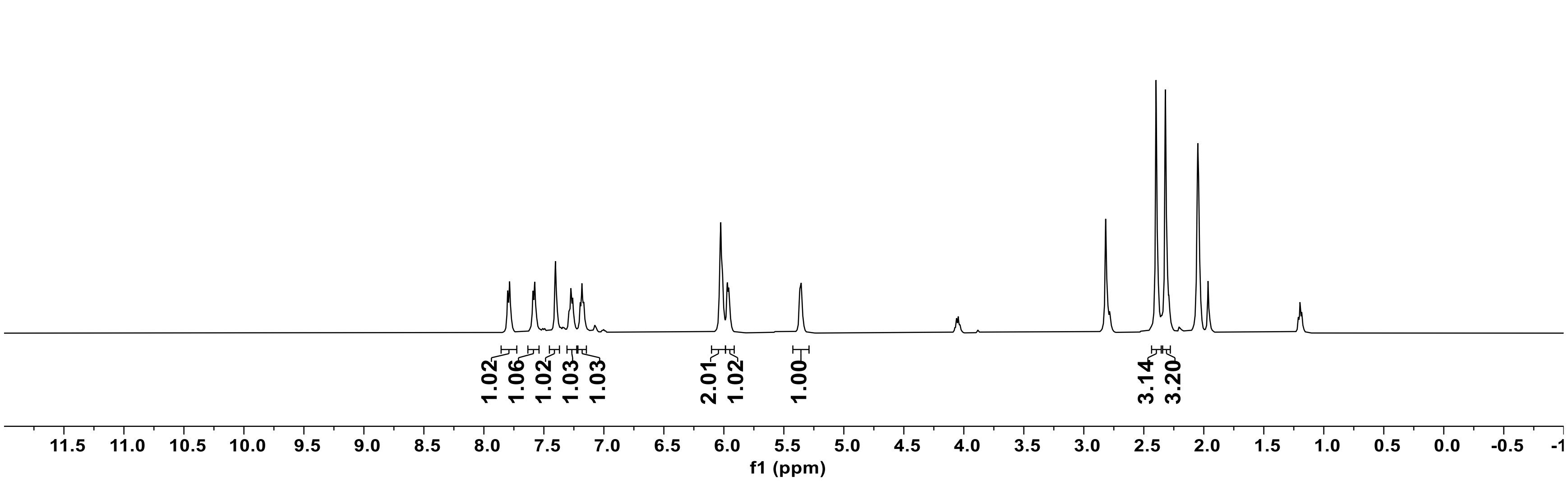

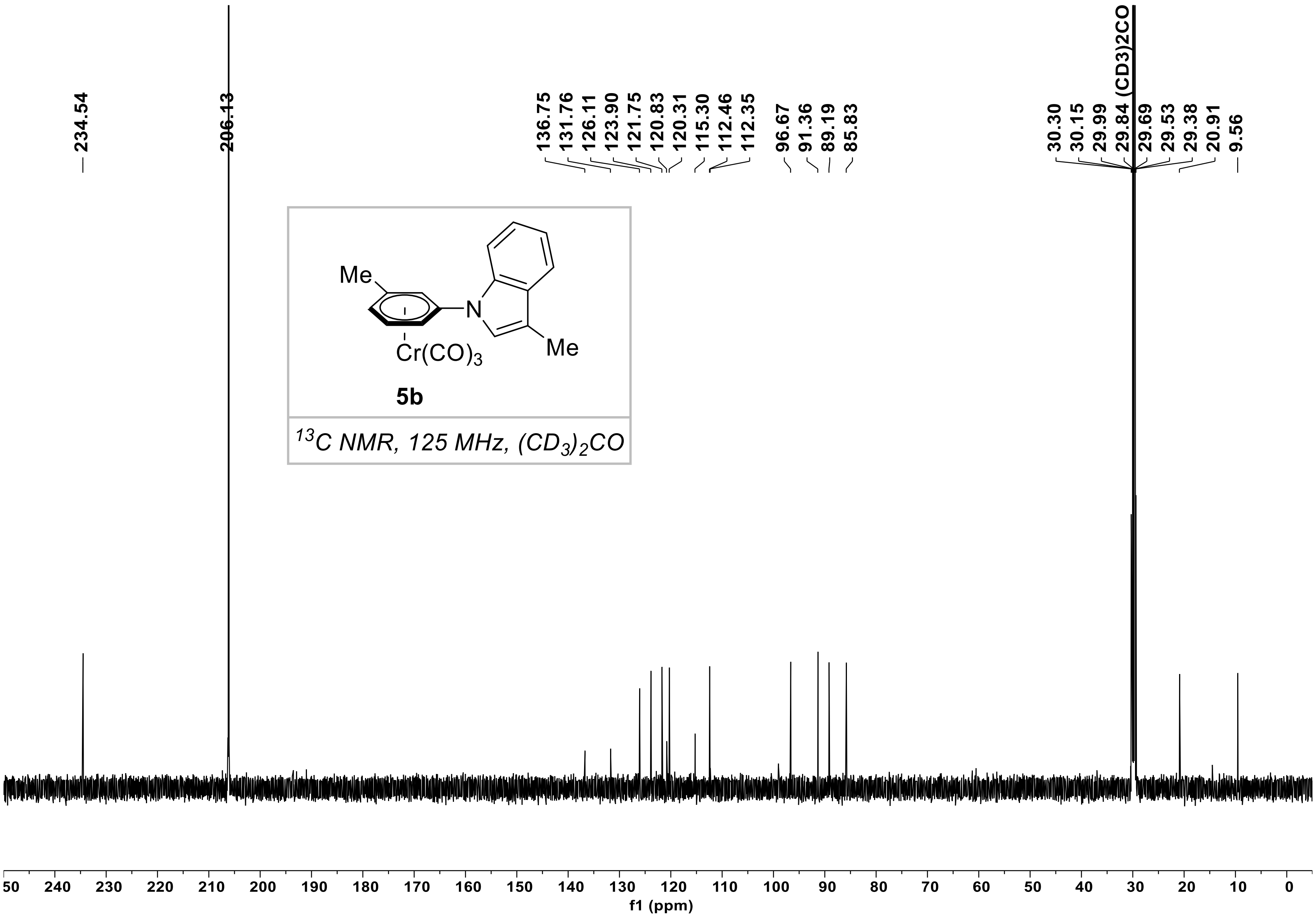
234.54
206.13
136.75
131.76
126.11
123.90
121.75
120.83
120.31
115.30
112.46
112.35
96.67
91.36
89.19
85.83
30.30
30.15
29.99
29.84 (CD3)2CO
29.69
29.53
29.38
20.91
9.56
Me
N
Cr(CO)3
Me
5b
13C NMR, 125 MHz, (CD3)2CO
250 240 230 220 210 200 190 180 170 160 150 140 130 120 110 100 90 80 70 60 50 40 30 20 10 0
f1 (ppm)

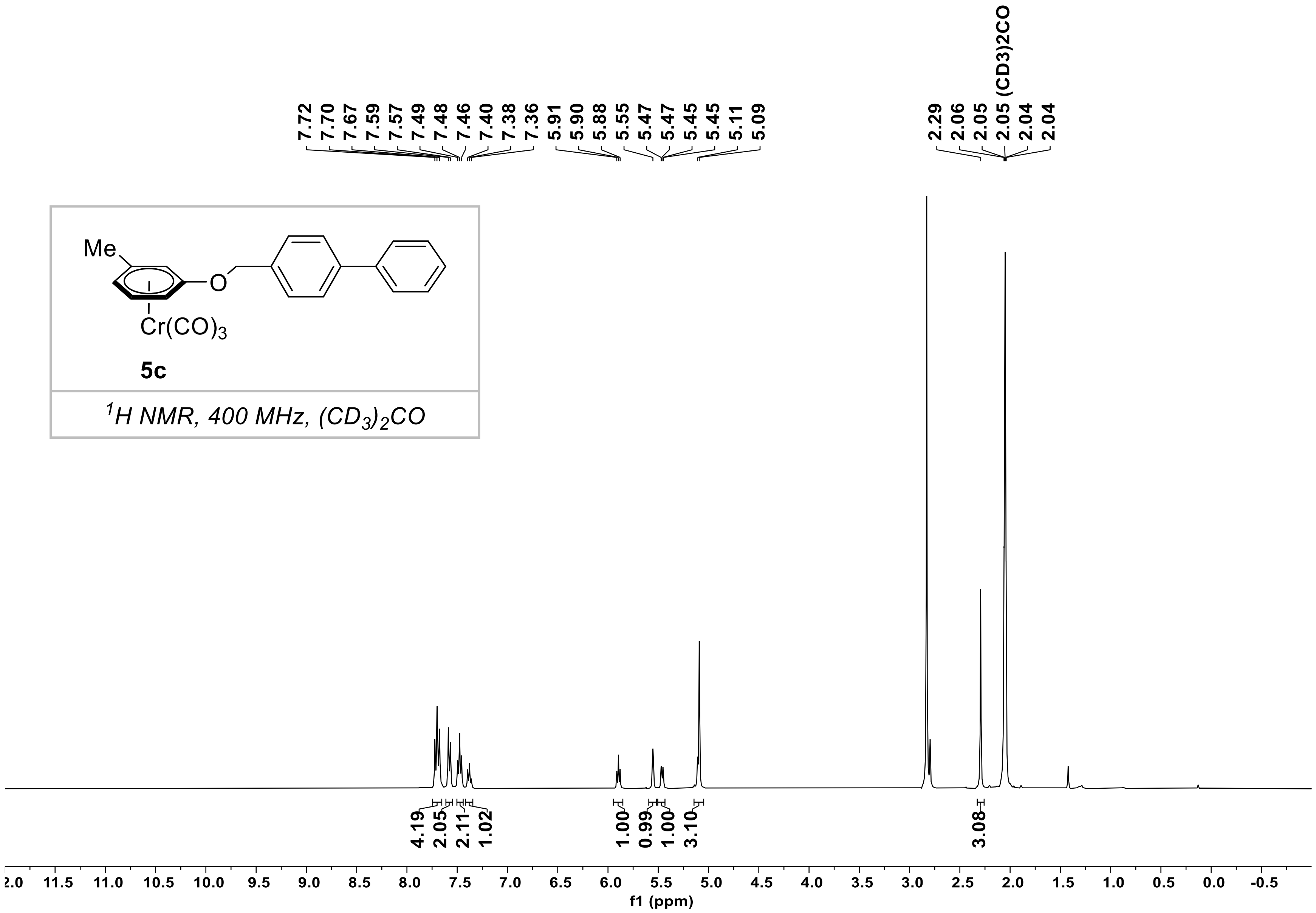
7.72
7.70
7.67
7.59
7.57
7.49
7.48
7.46
7.40
7.38
7.36
5.91
5.90
5.88
5.55
5.47
5.47
5.45
5.45
5.11
5.09
2.29
2.06
2.05
2.05 (CD3)2CO
2.04
2.04
Me
O
Cr(CO)3
5c
1H NMR, 400 MHz, (CD3)2CO
4.19
2.05
2.11
1.02
1.00
0.99
1.00
3.10
3.08
f1 (ppm)

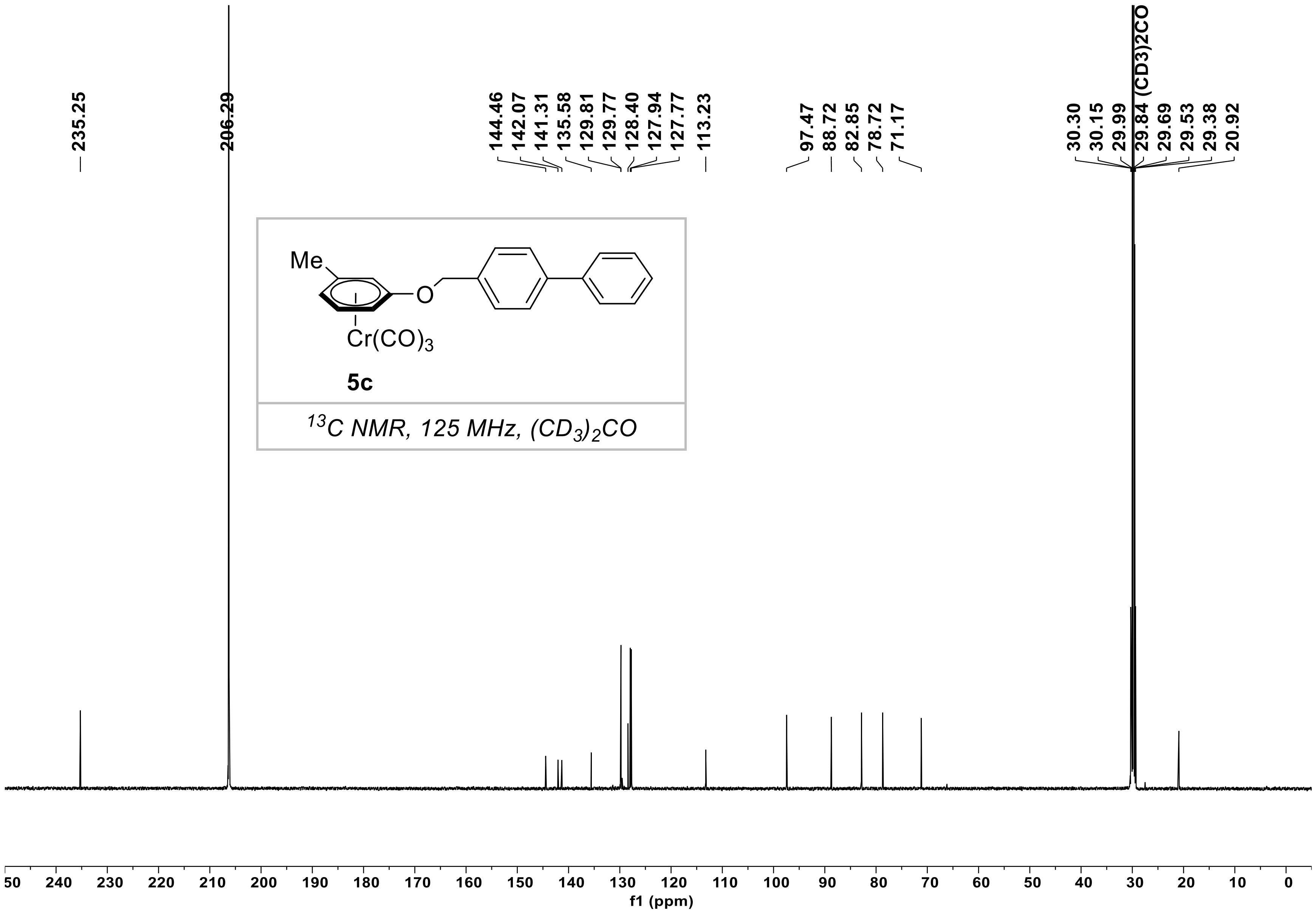
235.25
206.29
144.46
142.07
141.31
135.58
129.81
129.77
128.40
127.94
127.77
113.23
97.47
88.72
82.85
78.72
71.17
30.30
30.15
29.99
29.84 (CD3)2CO
29.69
29.53
29.38
20.92
Me
O
Cr(CO)3
5c
13C NMR, 125 MHz, (CD3)2CO
f1 (ppm)

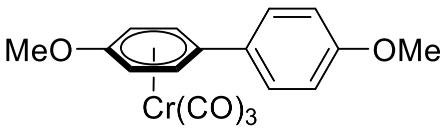

7a

^{1}H NMR, 500 MHz, $(CD_3)_2CO$

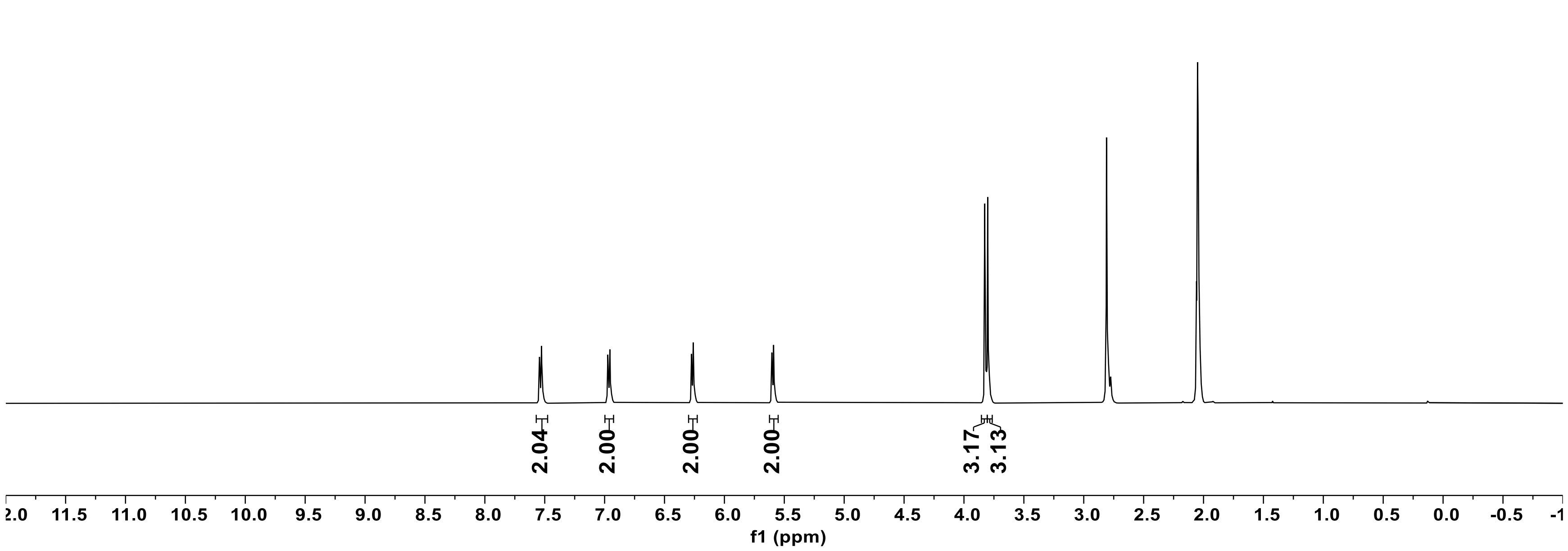

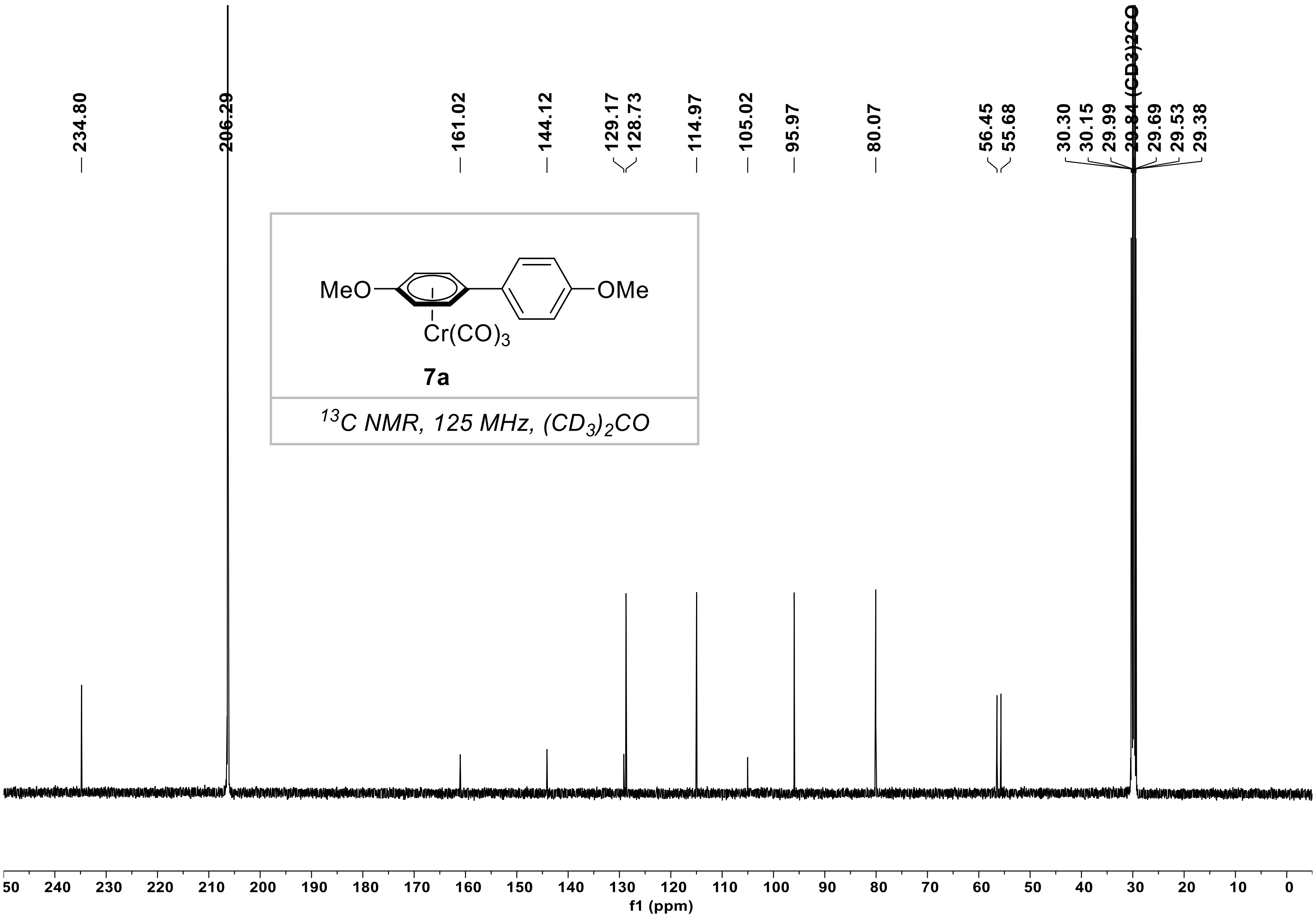
234.80
206.29
161.02
144.12
129.17
128.73
114.97
105.02
95.97
80.07
56.45
55.68
30.30
30.15
29.99
29.84 (CD3)2CO
29.69
29.53
29.38
MeO
OMe
Cr(CO)3
7a
13C NMR, 125 MHz, (CD3)2CO
50 240 230 220 210 200 190 180 170 160 150 140 130 120 110 100 90 80 70 60 50 40 30 20 10 0
f1 (ppm)

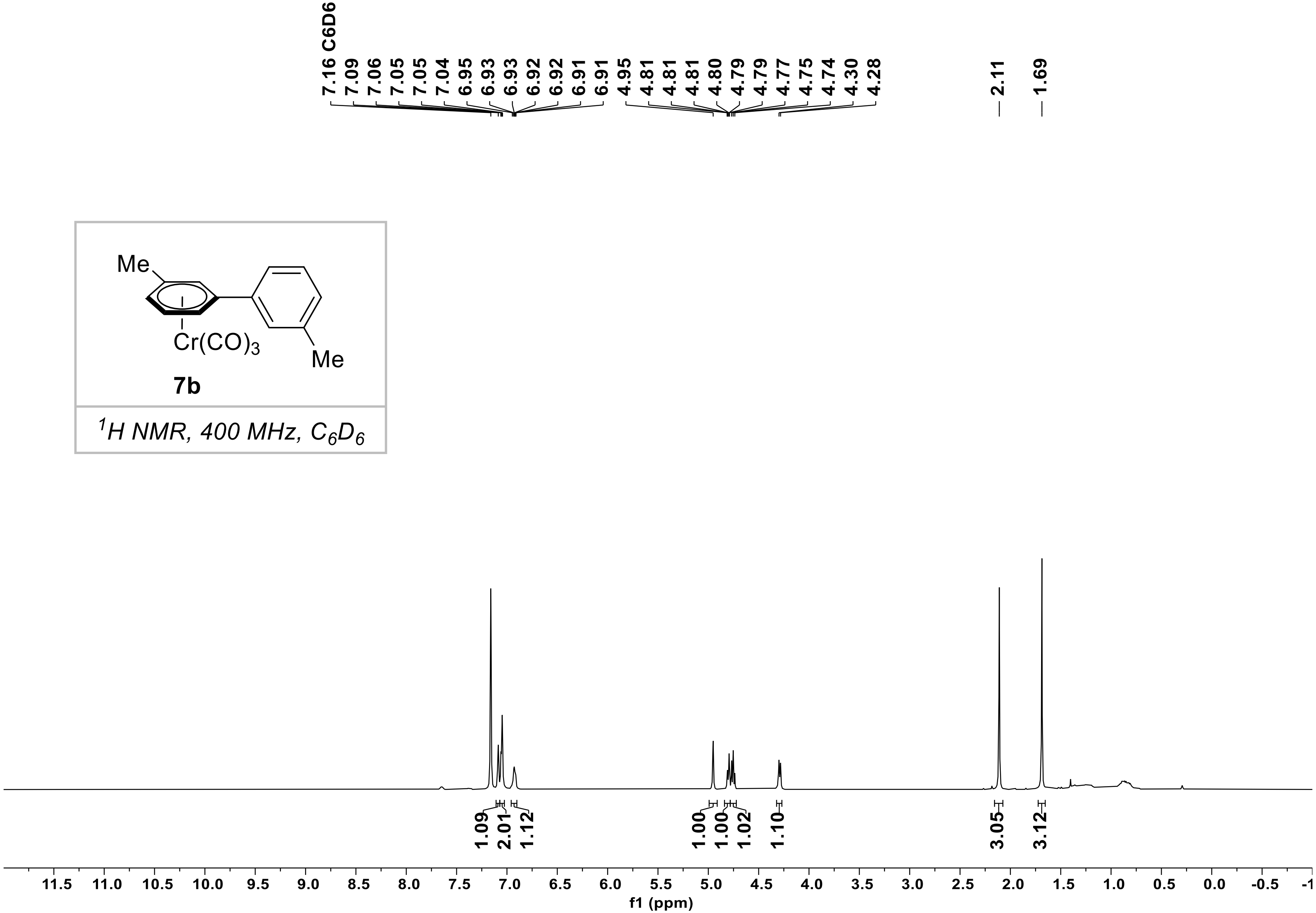
7.16 C6D6
7.09
7.06
7.05
7.05
7.04
6.95
6.93
6.93
6.92
6.92
6.91
6.91
4.95
4.81
4.81
4.81
4.80
4.79
4.79
4.77
4.75
4.74
4.30
4.28
2.11
1.69
Me
Cr(CO)3
Me
7b
1H NMR, 400 MHz, C6D6
1.09
2.01
1.12
1.00
1.00
1.02
1.10
3.05
3.12
11.5 11.0 10.5 10.0 9.5 9.0 8.5 8.0 7.5 7.0 6.5 6.0 5.5 5.0 4.5 4.0 3.5 3.0 2.5 2.0 1.5 1.0 0.5 0.0 -0.5 -1
f1 (ppm)

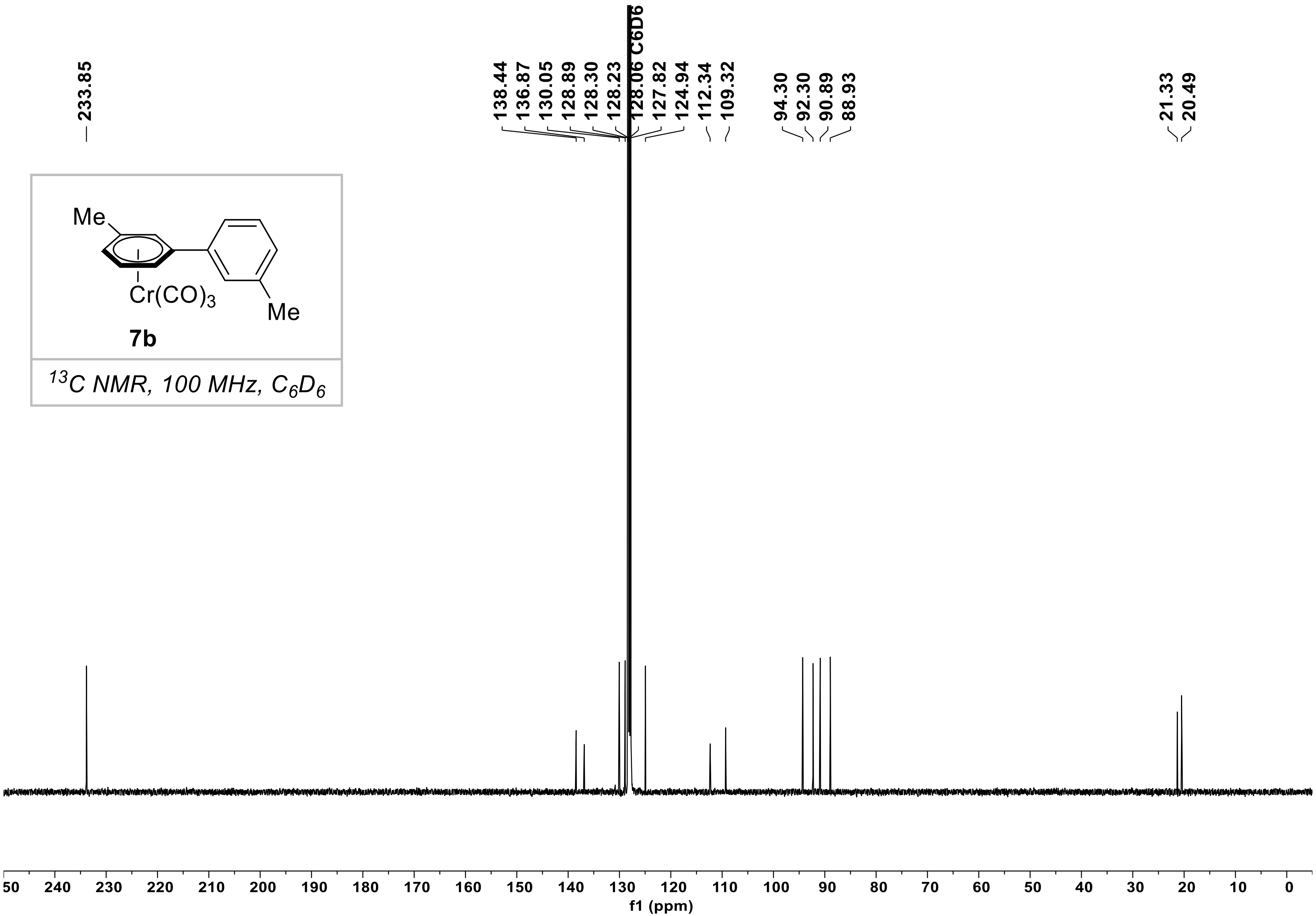

7b

^{13}C NMR, 100 MHz, C_6D_6

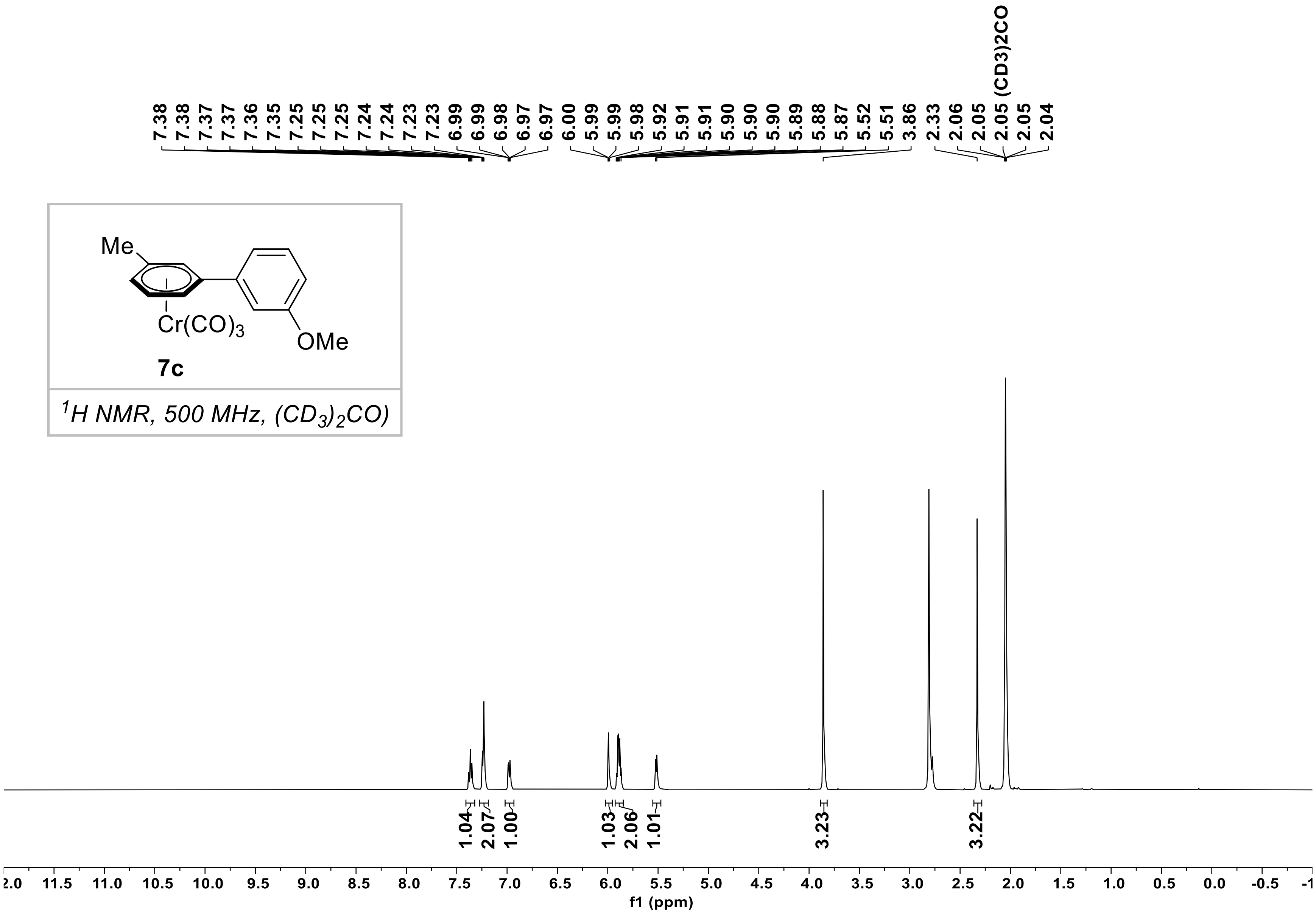
7.38
7.38
7.37
7.37
7.36
7.35
7.25
7.25
7.25
7.24
7.24
7.23
7.23
6.99
6.99
6.98
6.97
6.97
6.00
5.99
5.99
5.98
5.92
5.91
5.91
5.90
5.90
5.90
5.89
5.88
5.87
5.52
5.51
3.86
2.33
2.06
2.05
2.05 (CD3)2CO
2.05
2.04
Me
Cr(CO)3
OMe
7c
1H NMR, 500 MHz, (CD3)2CO
1.04
2.07
1.00
1.03
2.06
1.01
3.23
3.22
f1 (ppm)

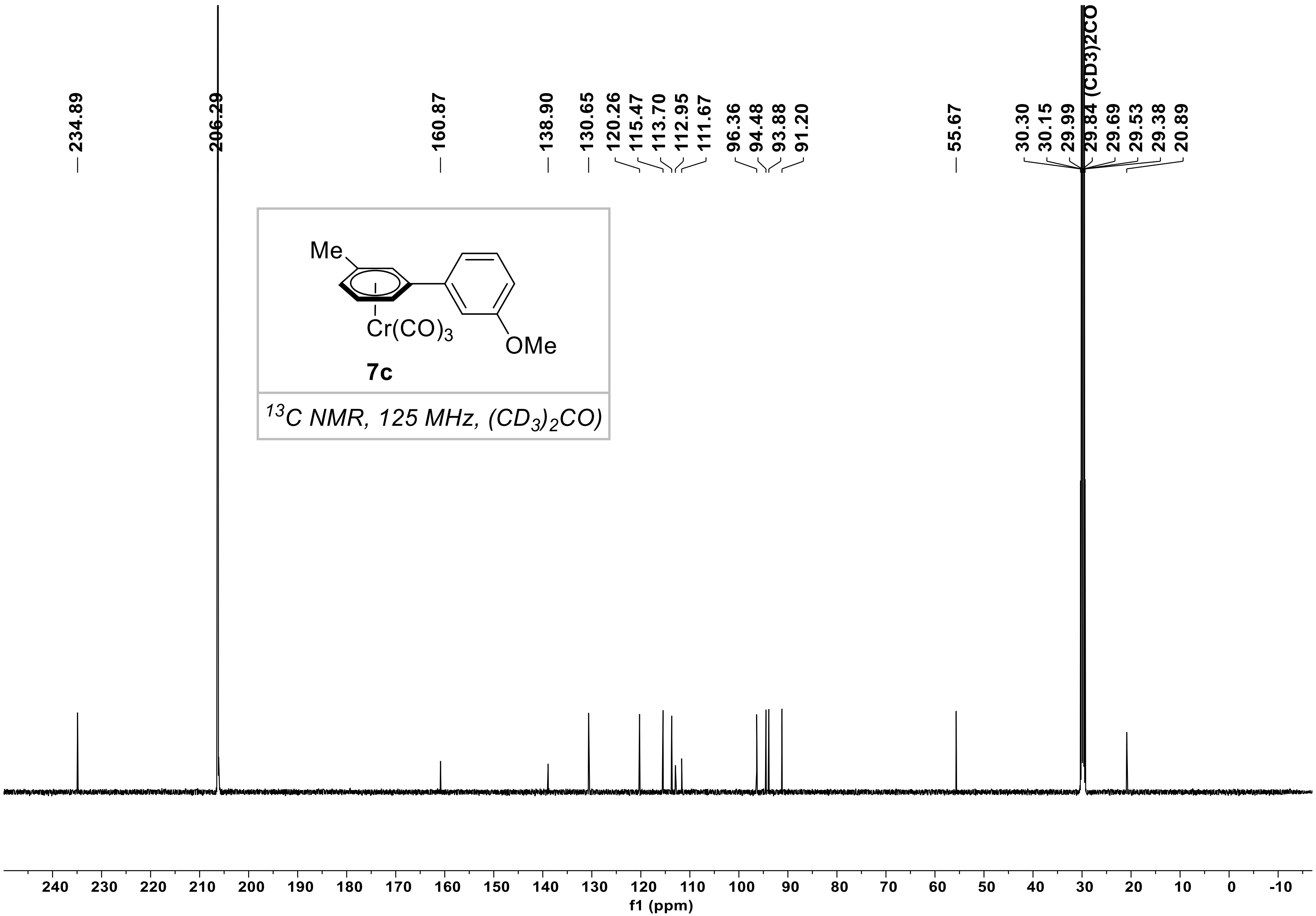
234.89
206.29
160.87
138.90
130.65
120.26
115.47
113.70
112.95
111.67
96.36
94.48
93.88
91.20
55.67
30.30
30.15
29.99
29.84 (CD3)2CO
29.69
29.53
29.38
20.89
Me
Cr(CO)3
OMe
7c
13C NMR, 125 MHz, (CD3)2CO
240 230 220 210 200 190 180 170 160 150 140 130 120 110 100 90 80 70 60 50 40 30 20 10 0 -10
f1 (ppm)

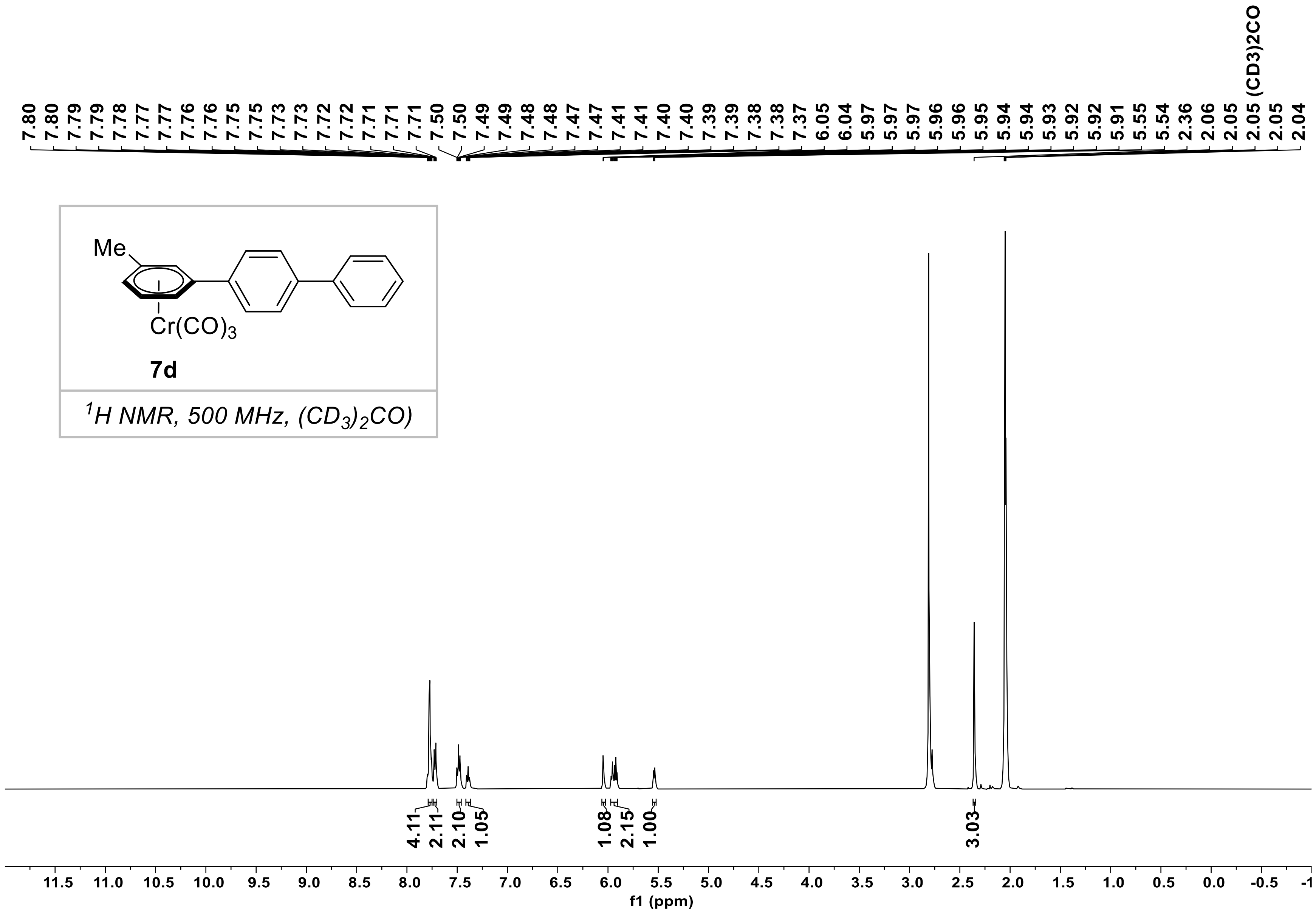
7.80 7.80 7.79 7.79 7.78 7.77 7.77 7.76 7.76 7.75 7.75 7.73 7.73 7.72 7.72 7.71 7.71 7.71 7.50 7.50 7.49 7.49 7.48 7.48 7.47 7.47 7.41 7.41 7.40 7.40 7.39 7.39 7.38 7.38 7.37 6.05 6.04 5.97 5.97 5.97 5.96 5.96 5.95 5.94 5.94 5.93 5.92 5.92 5.91 5.55 5.54 2.36 2.06 2.05 2.05 (CD3)2CO 2.05 2.04
Me
Cr(CO)3
7d
1H NMR, 500 MHz, (CD3)2CO)
4.11 2.11 2.10 1.05 1.08 2.15 1.00 3.03
11.5 11.0 10.5 10.0 9.5 9.0 8.5 8.0 7.5 7.0 6.5 6.0 5.5 5.0 4.5 4.0 3.5 3.0 2.5 2.0 1.5 1.0 0.5 0.0 -0.5 -1
f1 (ppm)

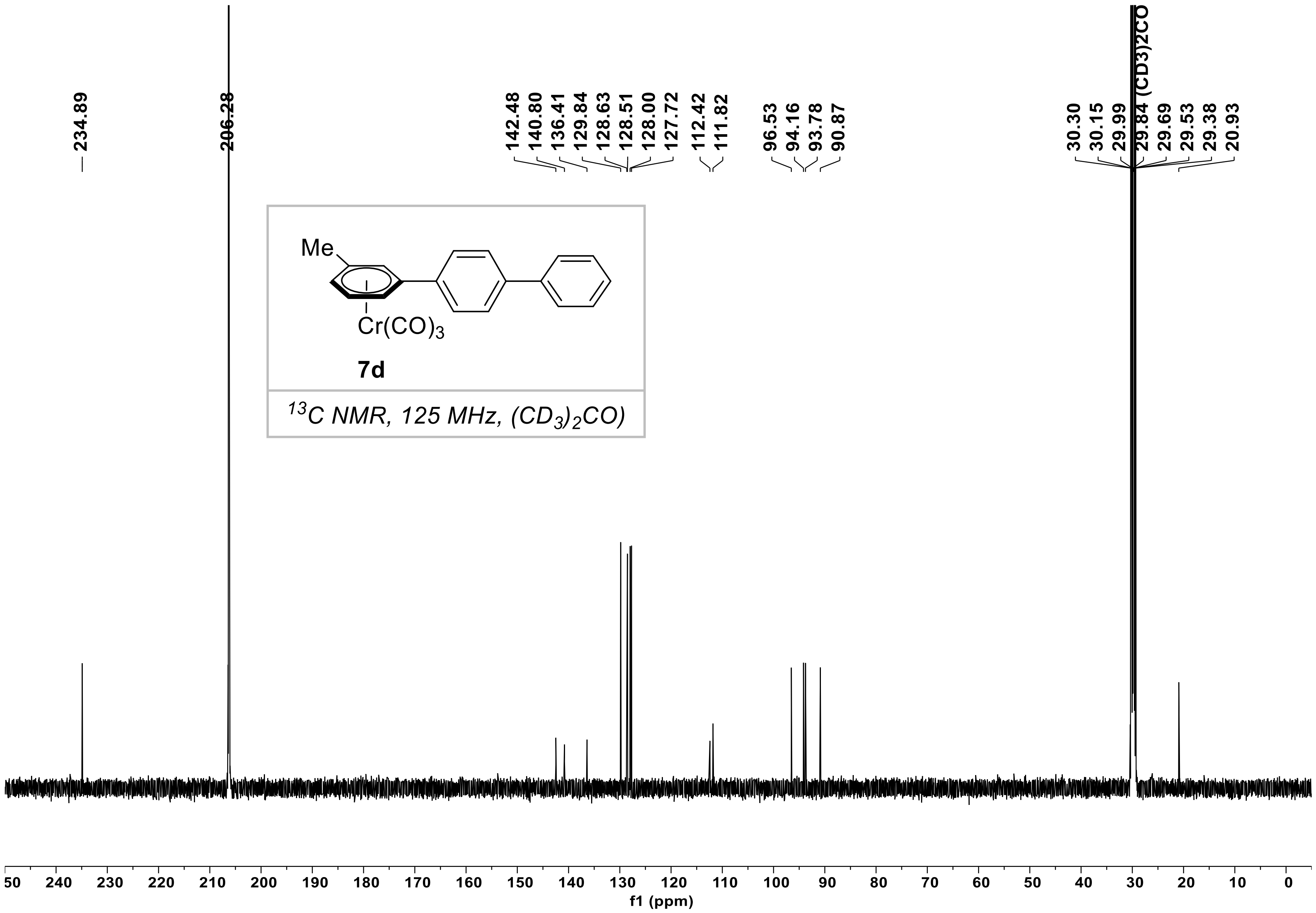
234.89
206.28
142.48
140.80
136.41
129.84
128.63
128.51
128.00
127.72
112.42
111.82
96.53
94.16
93.78
90.87
30.30
30.15
29.99
29.84 (CD3)2CO
29.69
29.53
29.38
20.93
Me
Cr(CO)3
7d
13C NMR, 125 MHz, (CD3)2CO)
50 240 230 220 210 200 190 180 170 160 150 140 130 120 110 100 90 80 70 60 50 40 30 20 10 0
f1 (ppm)

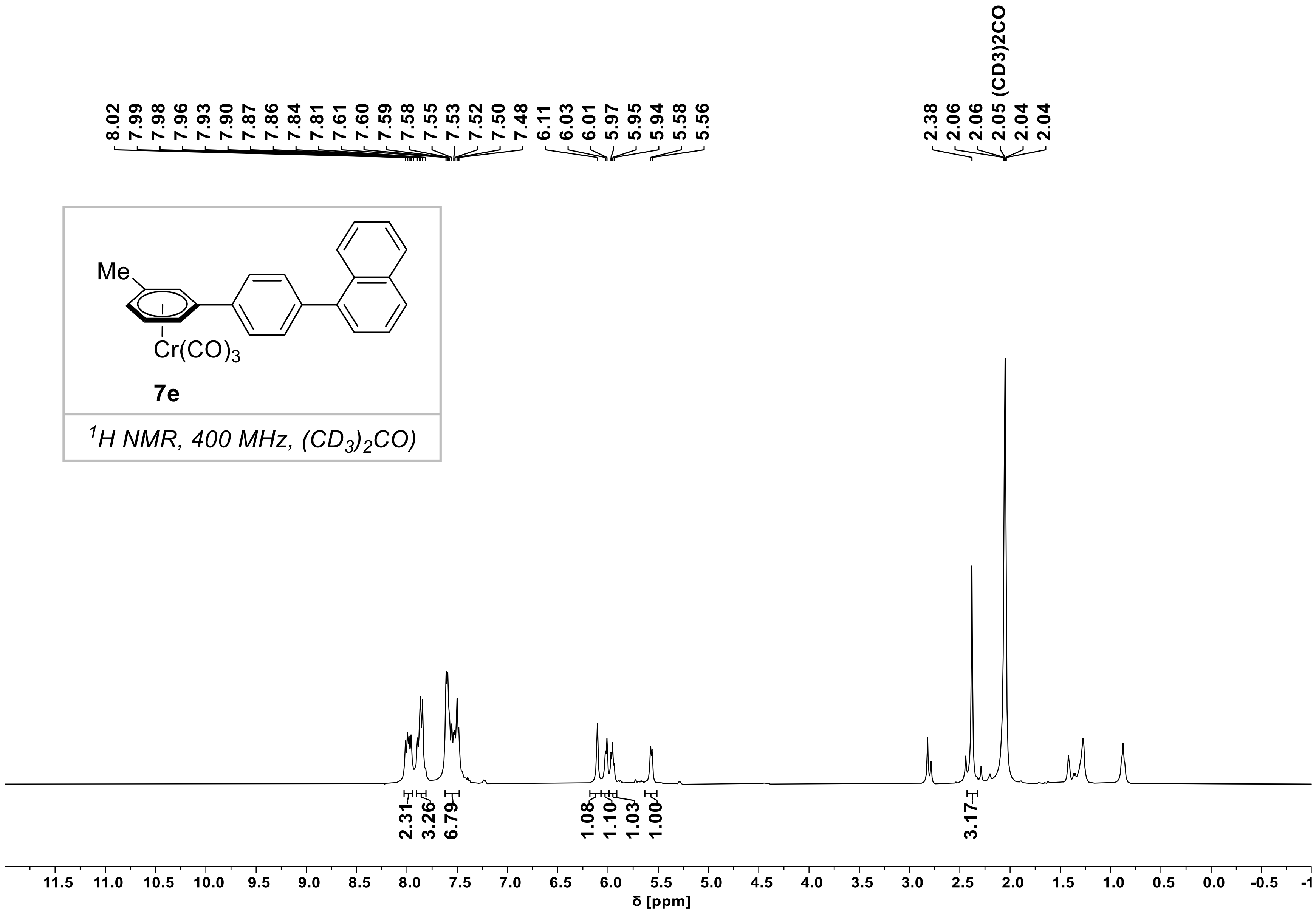
8.02
7.99
7.98
7.96
7.93
7.90
7.87
7.86
7.84
7.81
7.61
7.60
7.59
7.58
7.55
7.53
7.52
7.50
7.48
6.11
6.03
6.01
5.97
5.95
5.94
5.58
5.56
2.38
2.06
2.06
2.05 (CD3)2CO
2.04
2.04
Me
Cr(CO)3
7e
1H NMR, 400 MHz, (CD3)2CO
2.31
3.26
6.79
1.08
1.10
1.03
1.00
3.17
δ [ppm]

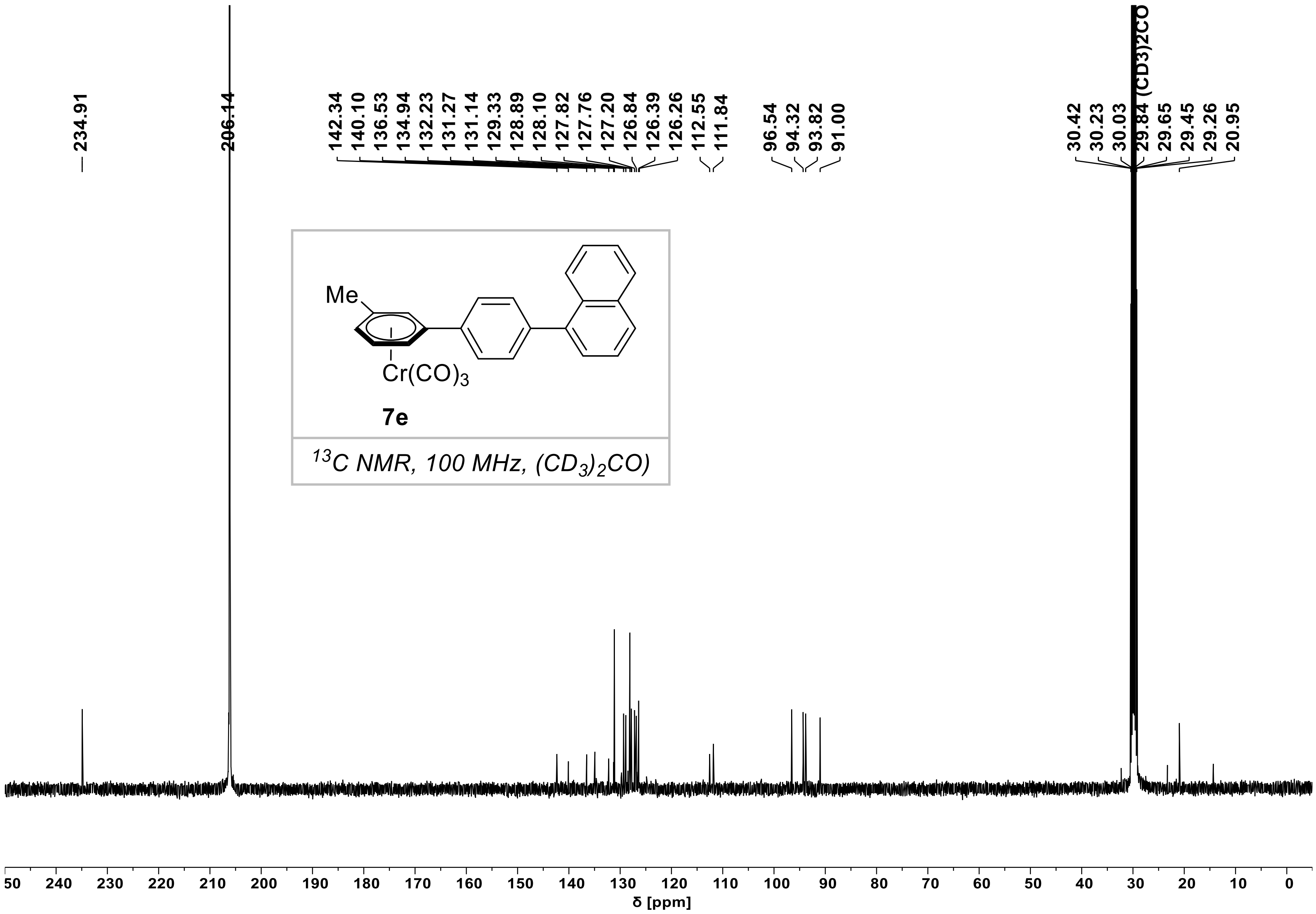
234.91
206.14
142.34
140.10
136.53
134.94
132.23
131.27
131.14
129.33
128.89
128.10
127.82
127.76
127.20
126.84
126.39
126.26
112.55
111.84
96.54
94.32
93.82
91.00
30.42
30.23
30.03
29.84 (CD3)2CO
29.65
29.45
29.26
20.95
Me
Cr(CO)3
7e
13C NMR, 100 MHz, (CD3)2CO
50 240 230 220 210 200 190 180 170 160 150 140 130 120 110 100 90 80 70 60 50 40 30 20 10 0
δ [ppm]

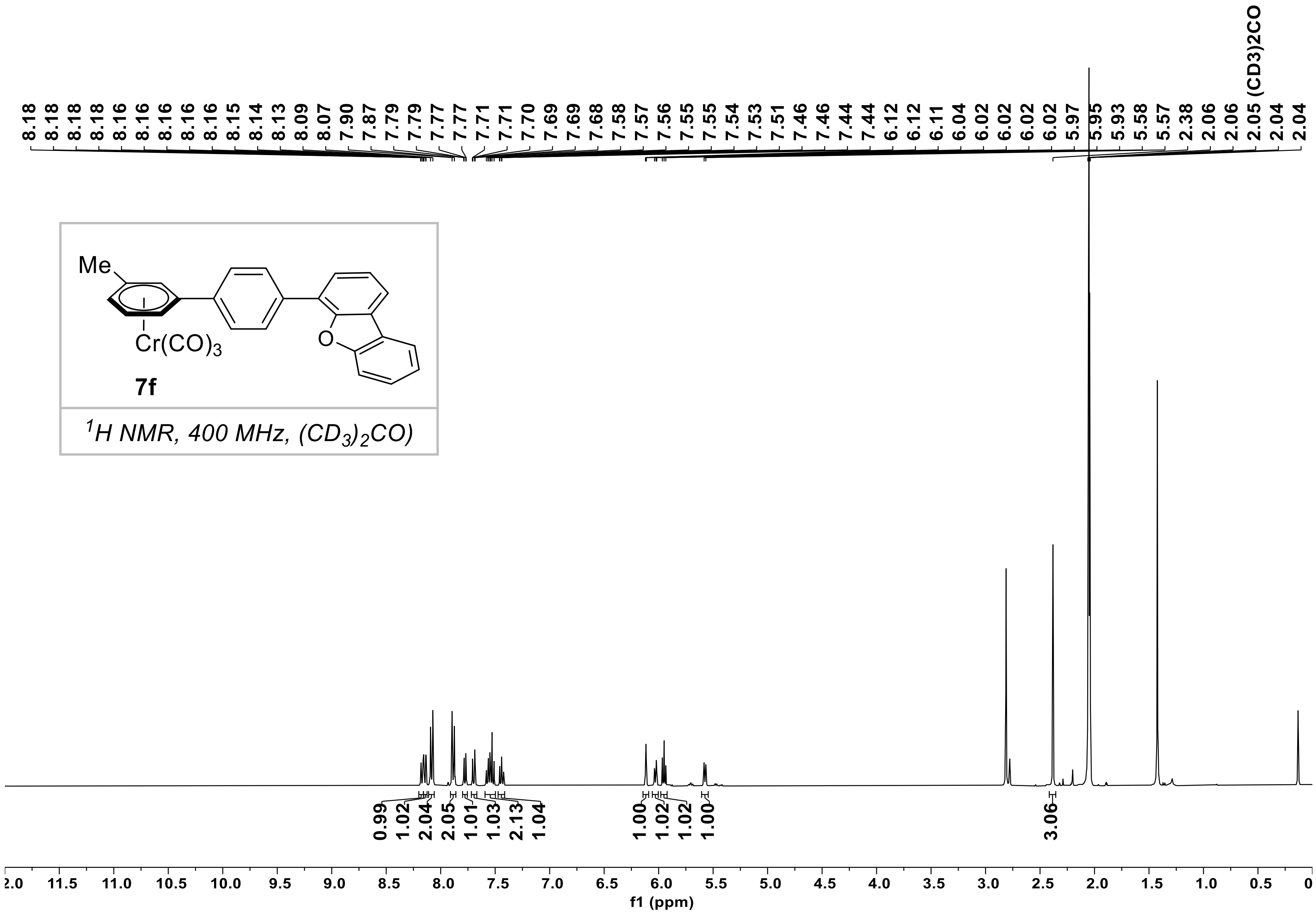

8.18 8.18 8.18 8.18 8.16 8.16 8.16 8.16 8.16 8.15 8.14 8.13 8.09 8.07 7.90 7.87 7.79 7.79 7.77 7.77 7.71 7.71 7.71 7.70 7.69 7.69 7.68 7.58 7.57 7.56 7.55 7.55 7.54 7.53 7.51 7.46 7.46 7.44 7.44 6.12 6.12 6.11 6.04 6.02 6.02 6.02 6.02 5.97 5.95 5.93 5.58 5.57 2.38 2.06 2.06 2.05 (CD3)2CO 2.04 2.04
Me
Cr(CO)3
O
7f
1H NMR, 400 MHz, (CD3)2CO
0.99 1.02 2.04 2.05 1.01 1.03 2.13 1.04 1.00 1.02 1.02 1.00 3.06
2.0 11.5 11.0 10.5 10.0 9.5 9.0 8.5 8.0 7.5 7.0 6.5 6.0 5.5 5.0 4.5 4.0 3.5 3.0 2.5 2.0 1.5 1.0 0.5 0
f1 (ppm)

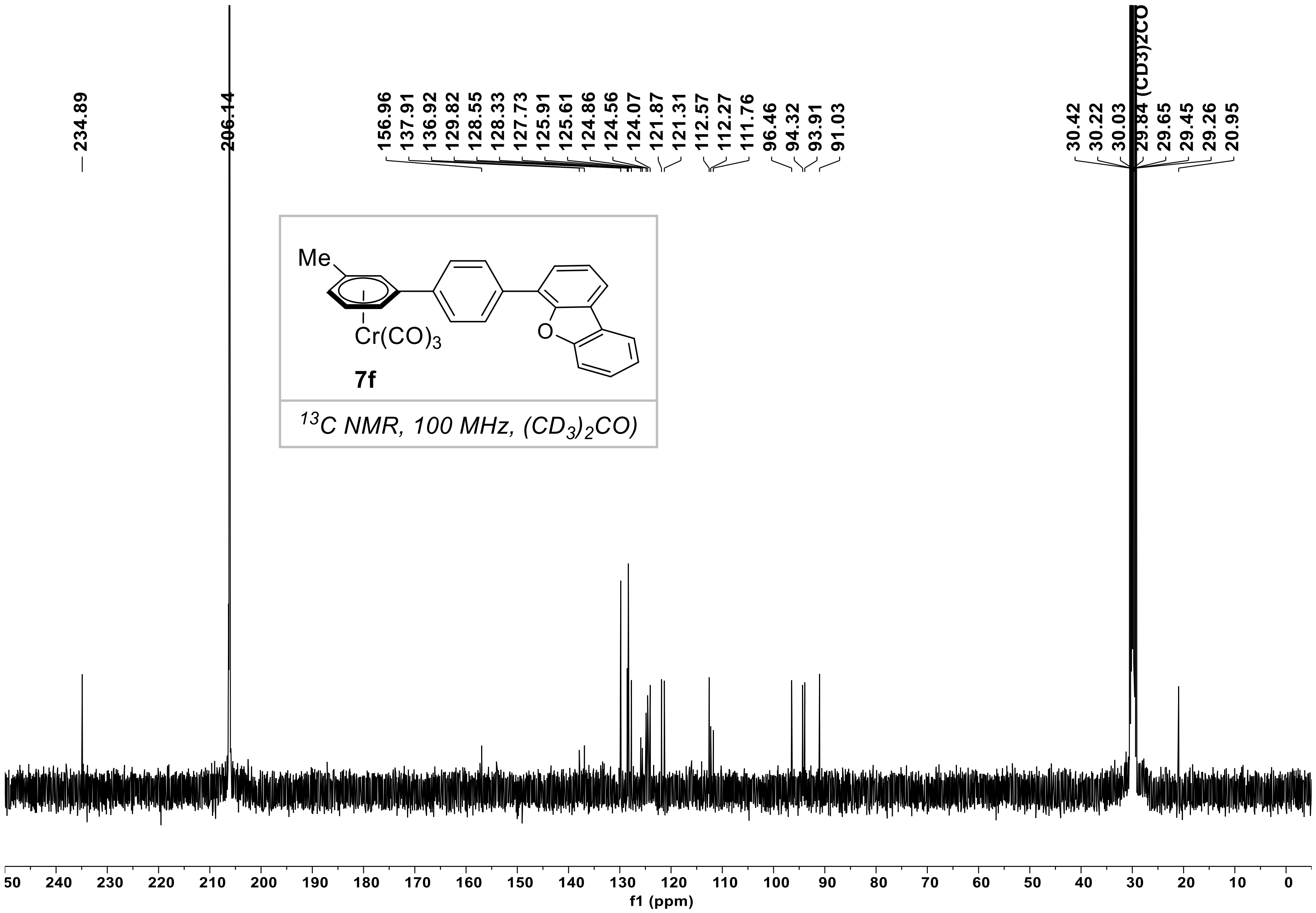
234.89
206.14
156.96
137.91
136.92
129.82
128.55
128.33
127.73
125.91
125.61
124.86
124.56
124.07
121.87
121.31
112.57
112.27
111.76
96.46
94.32
93.91
91.03
30.42
30.22
30.03
29.84 (CD3)2CO
29.65
29.45
29.26
20.95
Me
Cr(CO)3
O
7f
13C NMR, 100 MHz, (CD3)2CO)
f1 (ppm)

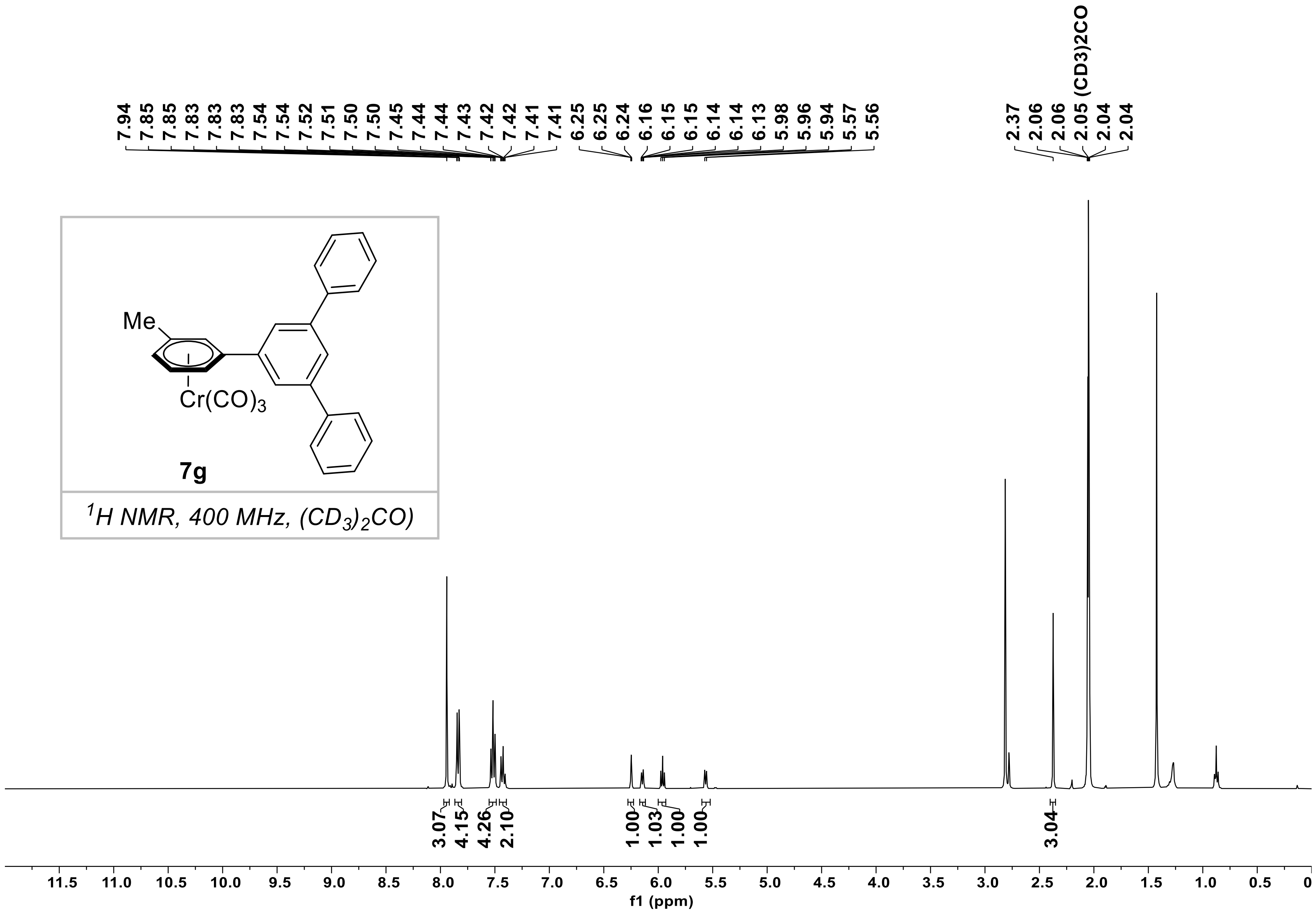
7.94 7.85 7.85 7.83 7.83 7.83 7.54 7.54 7.52 7.51 7.50 7.50 7.45 7.44 7.44 7.43 7.42 7.42 7.41 7.41 6.25 6.25 6.24 6.16 6.15 6.15 6.14 6.14 6.13 5.98 5.96 5.94 5.57 5.56
2.37 2.06 2.06 2.05 (CD3)2CO 2.04 2.04
Me
Cr(CO)3
7g
1H NMR, 400 MHz, (CD3)2CO)
3.07 4.15 4.26 2.10 1.00 1.03 1.00 1.00 3.04
11.5 11.0 10.5 10.0 9.5 9.0 8.5 8.0 7.5 7.0 6.5 6.0 5.5 5.0 4.5 4.0 3.5 3.0 2.5 2.0 1.5 1.0 0.5 0
f1 (ppm)

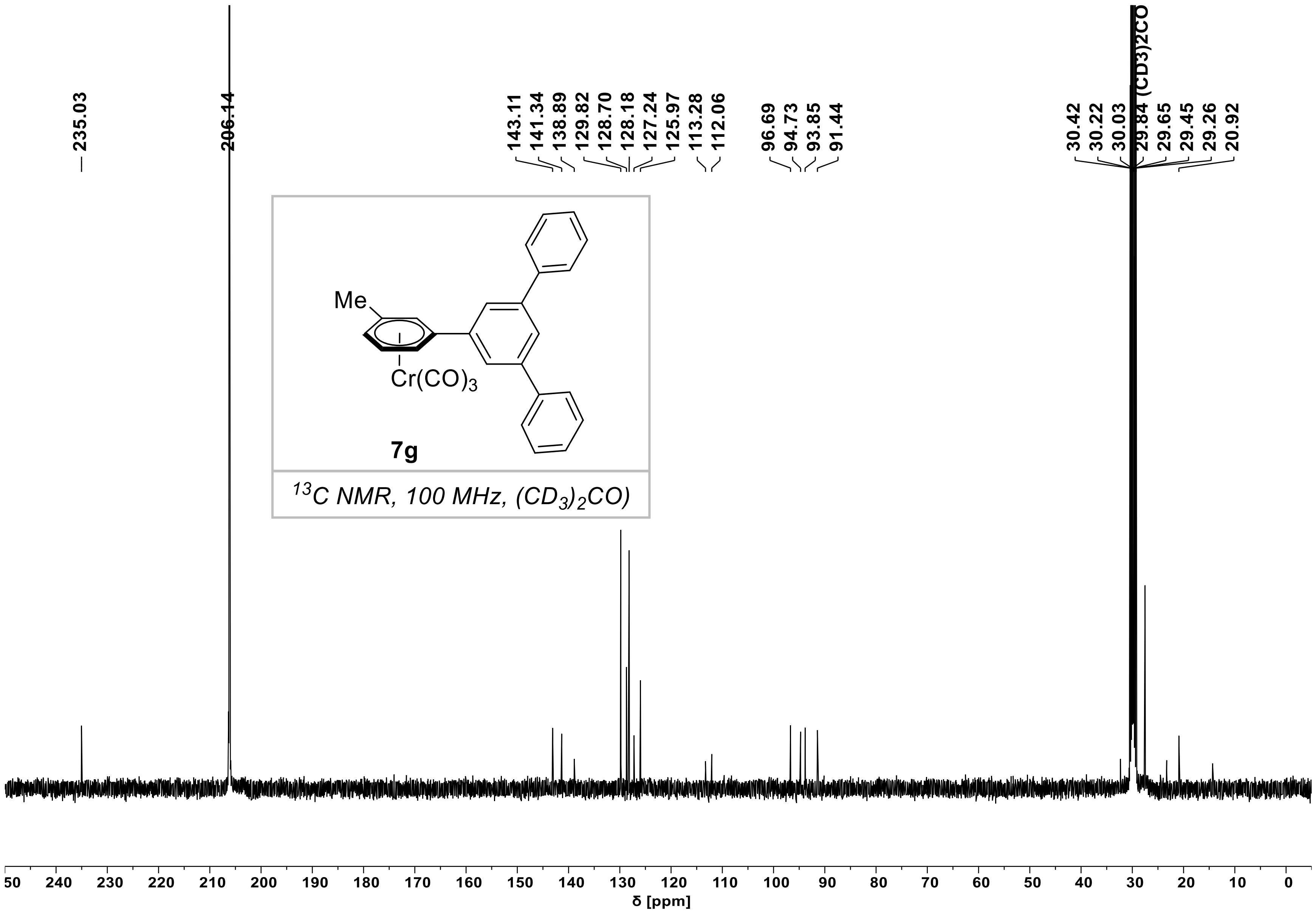
235.03
206.14
143.11
141.34
138.89
129.82
128.70
128.18
127.24
125.97
113.28
112.06
96.69
94.73
93.85
91.44
30.42
30.22
30.03
29.84 (CD3)2CO
29.65
29.45
29.26
20.92
Me
Cr(CO)3
7g
13C NMR, 100 MHz, (CD3)2CO
δ [ppm]

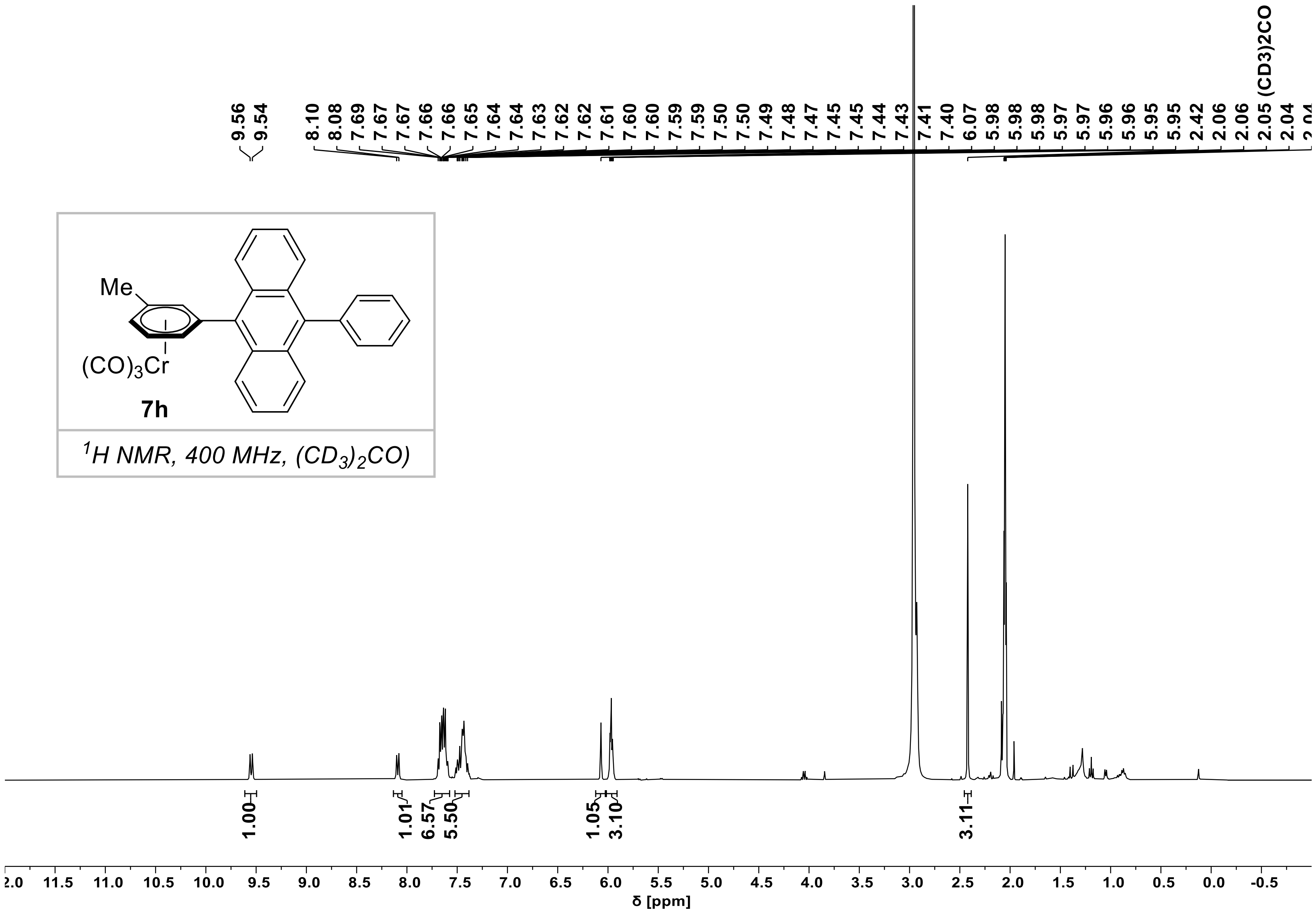

Me
(CO)3Cr
7h
1H NMR, 400 MHz, (CD3)2CO
(CD3)2CO
δ [ppm]

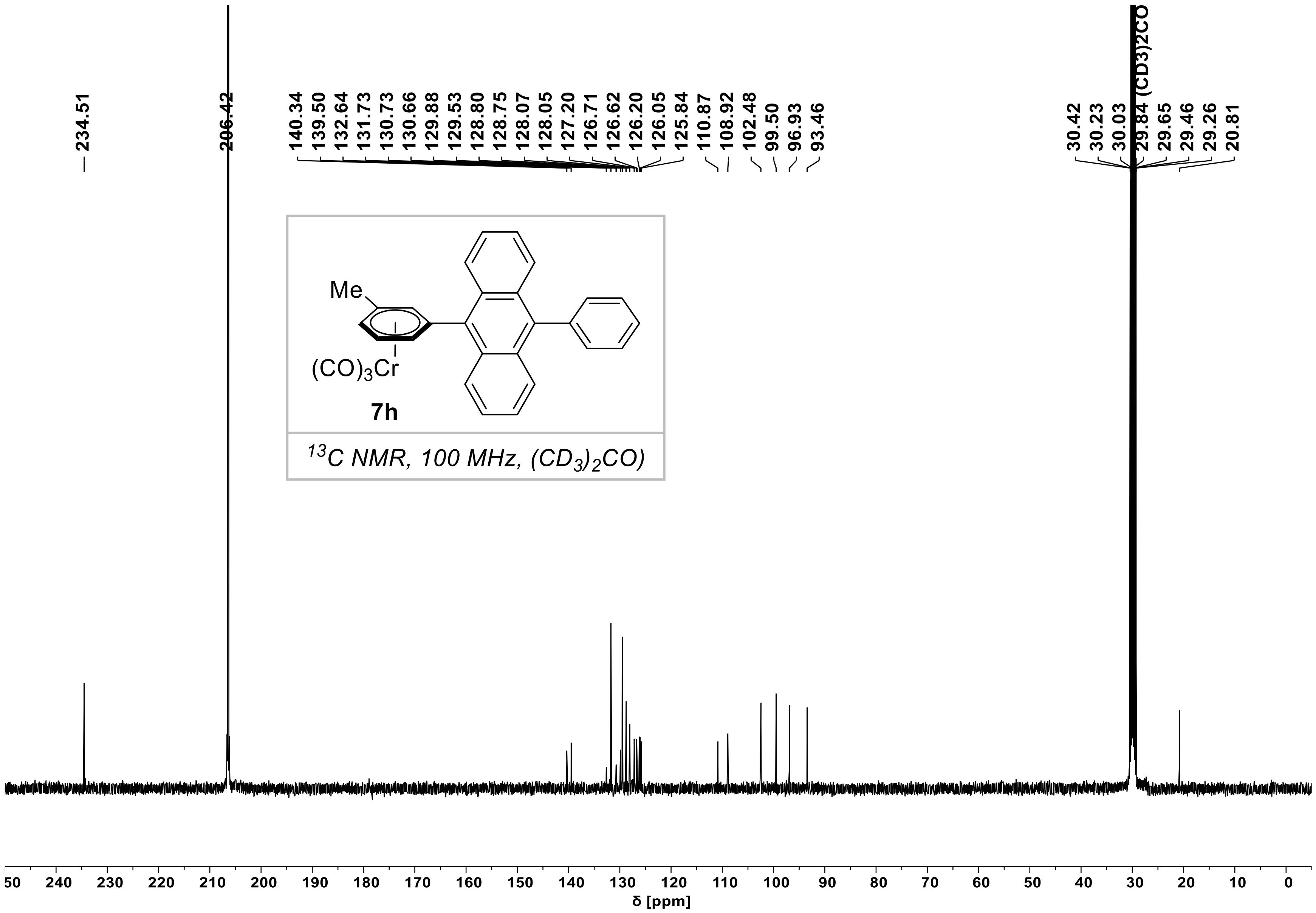
234.51
206.42
140.34
139.50
132.64
131.73
130.73
130.66
129.88
129.53
128.80
128.75
128.07
128.05
127.20
126.71
126.62
126.20
126.05
125.84
110.87
108.92
102.48
99.50
96.93
93.46
30.42
30.23
30.03
29.84 (CD3)2CO
29.65
29.46
29.26
20.81
Me
(CO)3Cr
7h
13C NMR, 100 MHz, (CD3)2CO
50 240 230 220 210 200 190 180 170 160 150 140 130 120 110 100 90 80 70 60 50 40 30 20 10 0
δ [ppm]

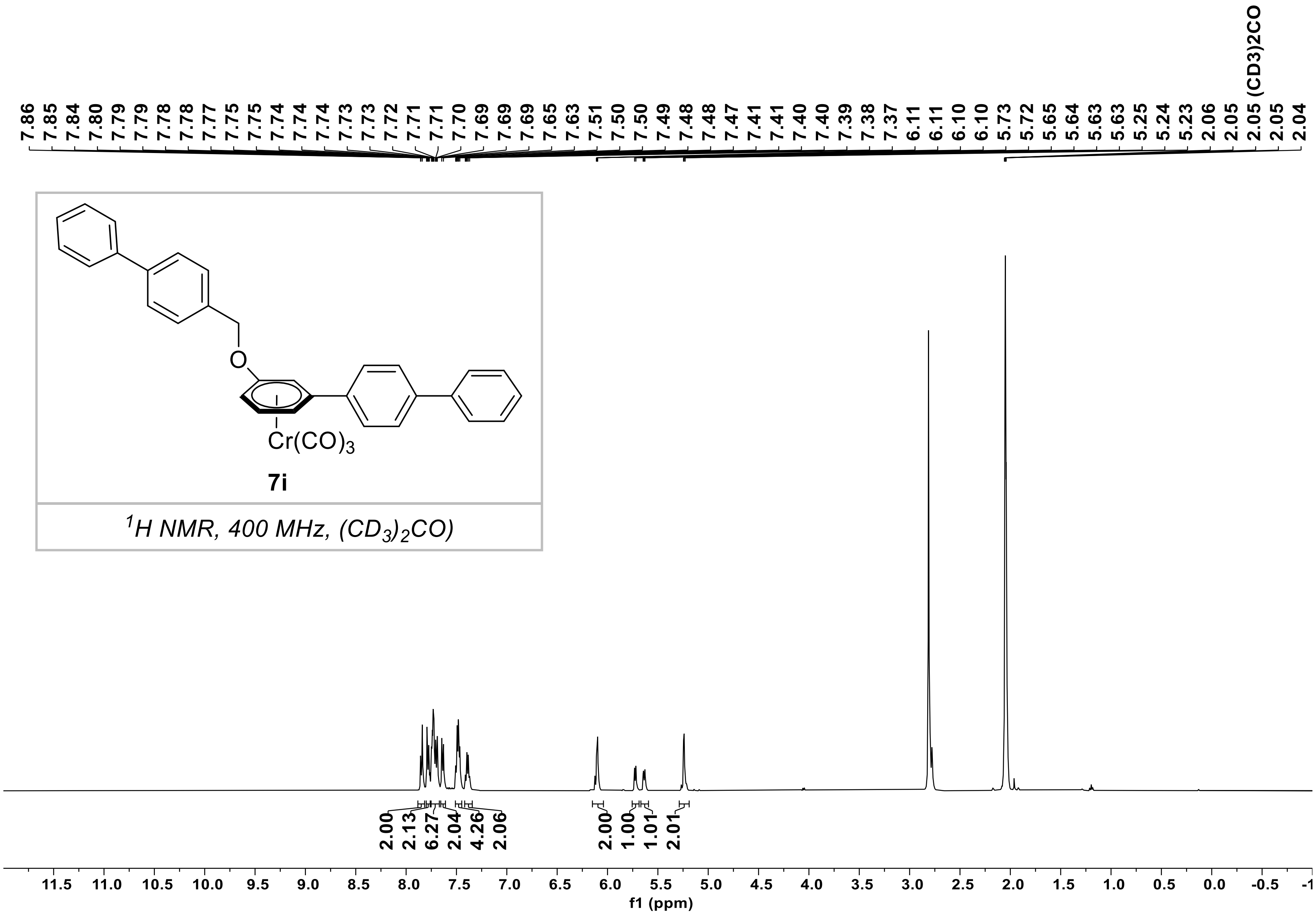
7.86
7.85
7.84
7.80
7.79
7.79
7.78
7.78
7.77
7.75
7.75
7.74
7.74
7.74
7.73
7.73
7.72
7.71
7.71
7.70
7.69
7.69
7.69
7.65
7.63
7.51
7.50
7.50
7.49
7.48
7.48
7.47
7.41
7.41
7.40
7.40
7.39
7.38
7.37
6.11
6.11
6.10
6.10
5.73
5.72
5.65
5.64
5.63
5.63
5.25
5.24
5.23
2.06
2.05
2.05 (CD3)2CO
2.05
2.04
O
Cr(CO)3
7i
1H NMR, 400 MHz, (CD3)2CO
2.00
2.13
6.27
2.04
4.26
2.06
2.00
1.00
1.01
2.01
11.5
11.0
10.5
10.0
9.5
9.0
8.5
8.0
7.5
7.0
6.5
6.0
5.5
5.0
4.5
4.0
3.5
3.0
2.5
2.0
1.5
1.0
0.5
0.0
-0.5
-1
f1 (ppm)

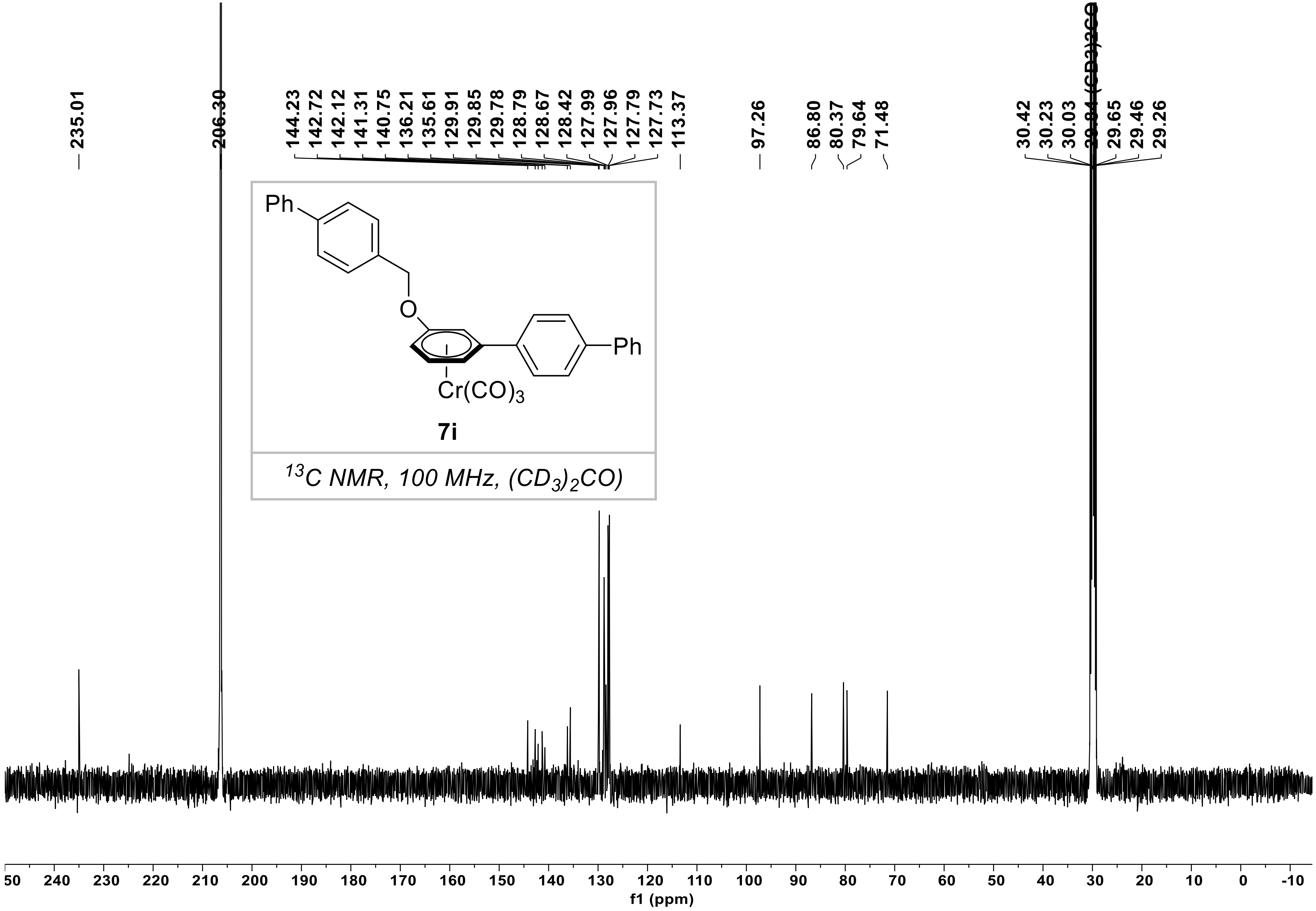
235.01
206.30
144.23
142.72
142.12
141.31
140.75
136.21
135.61
129.91
129.85
129.78
128.79
128.67
128.42
127.99
127.96
127.79
127.73
113.37
97.26
86.80
80.37
79.64
71.48
30.42
30.23
30.03
29.84 (CD3)2CO
29.65
29.46
29.26
Ph
O
Ph
Cr(CO)3
7i
13C NMR, 100 MHz, (CD3)2CO)
250 240 230 220 210 200 190 180 170 160 150 140 130 120 110 100 90 80 70 60 50 40 30 20 10 0 -10
f1 (ppm)

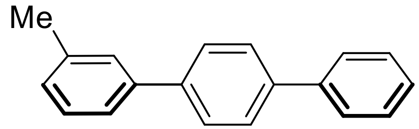

6d

^{1}H NMR, 400 MHz, $(CD_3)_2CO$

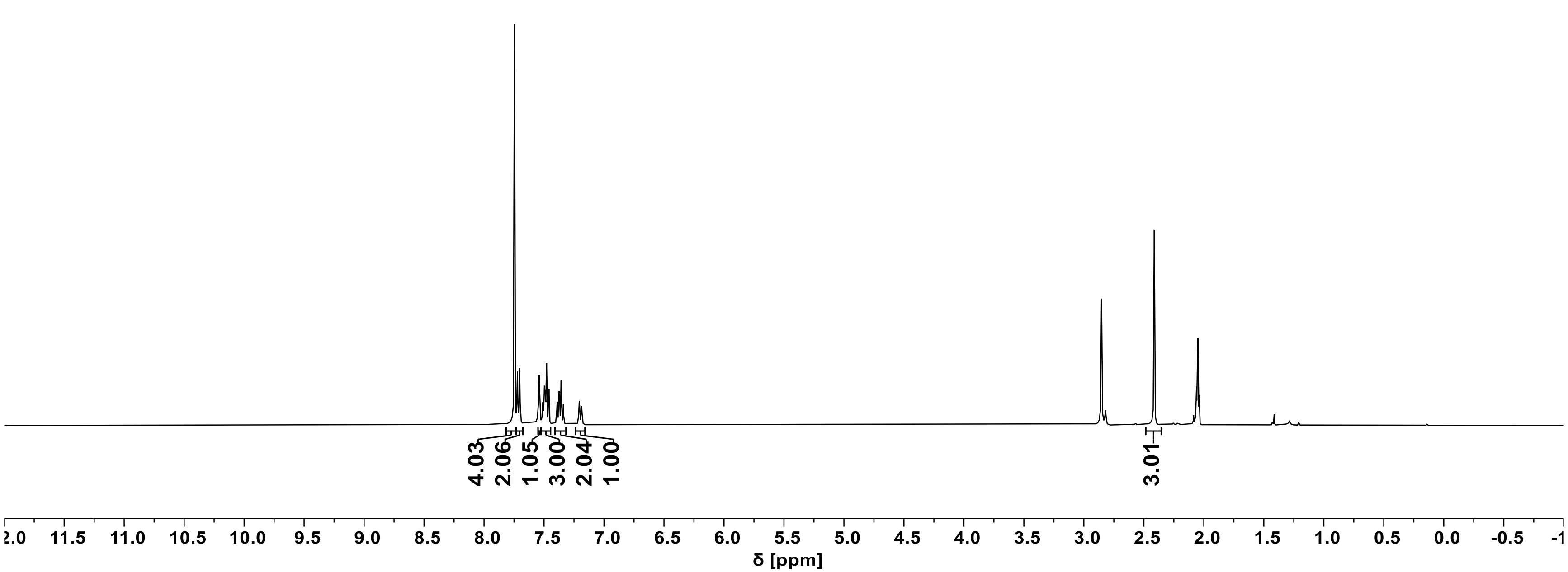

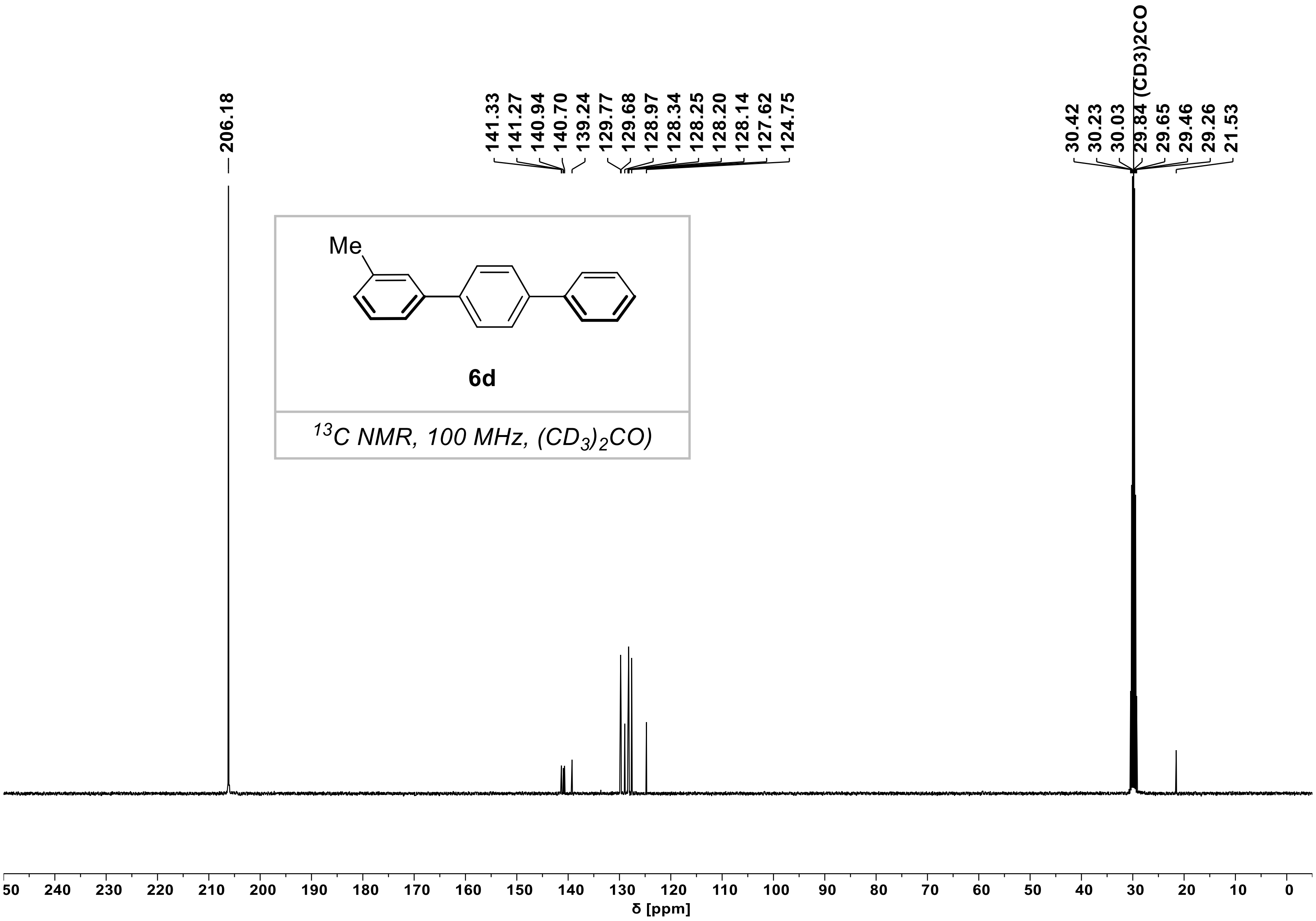
206.18
141.33
141.27
140.94
140.70
139.24
129.77
129.68
128.97
128.34
128.25
128.20
128.14
127.62
124.75
30.42
30.23
30.03
29.84 (CD3)2CO
29.65
29.46
29.26
21.53
Me
6d
^{13}C NMR, 100 MHz, $(CD_3)_2CO$)
δ [ppm]

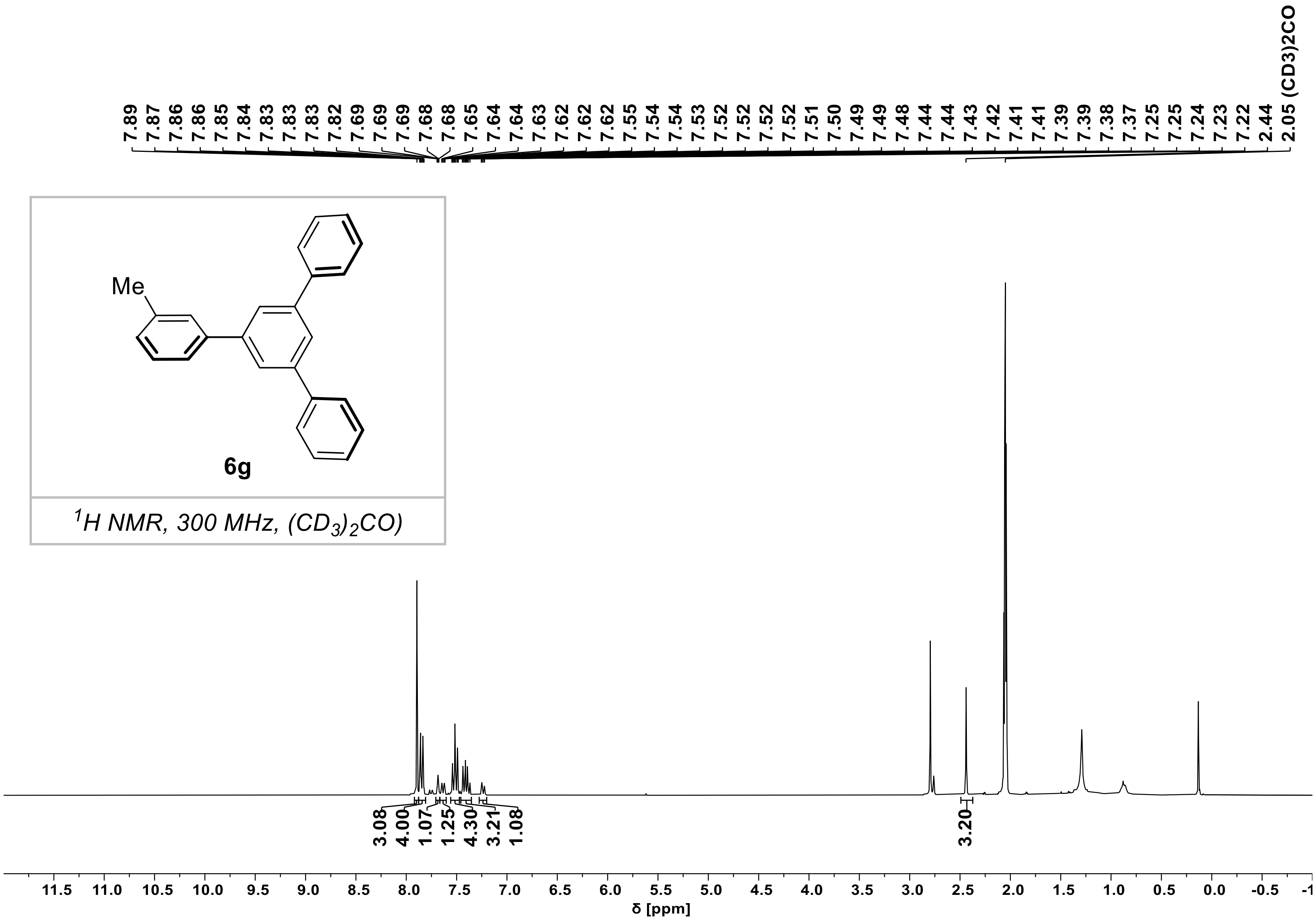

7.89 7.87 7.86 7.86 7.85 7.84 7.83 7.83 7.83 7.82 7.69 7.69 7.69 7.68 7.68 7.65 7.64 7.64 7.63 7.62 7.62 7.62 7.55 7.54 7.54 7.53 7.52 7.52 7.52 7.52 7.51 7.50 7.49 7.49 7.48 7.44 7.44 7.43 7.42 7.41 7.41 7.39 7.39 7.38 7.37 7.25 7.25 7.24 7.23 7.22 2.44 2.05 (CD3)2CO
Me
6g
1H NMR, 300 MHz, (CD3)2CO
3.08 4.00 1.07 1.25 4.30 3.21 1.08
3.20
11.5 11.0 10.5 10.0 9.5 9.0 8.5 8.0 7.5 7.0 6.5 6.0 5.5 5.0 4.5 4.0 3.5 3.0 2.5 2.0 1.5 1.0 0.5 0.0 -0.5 -1
δ [ppm]

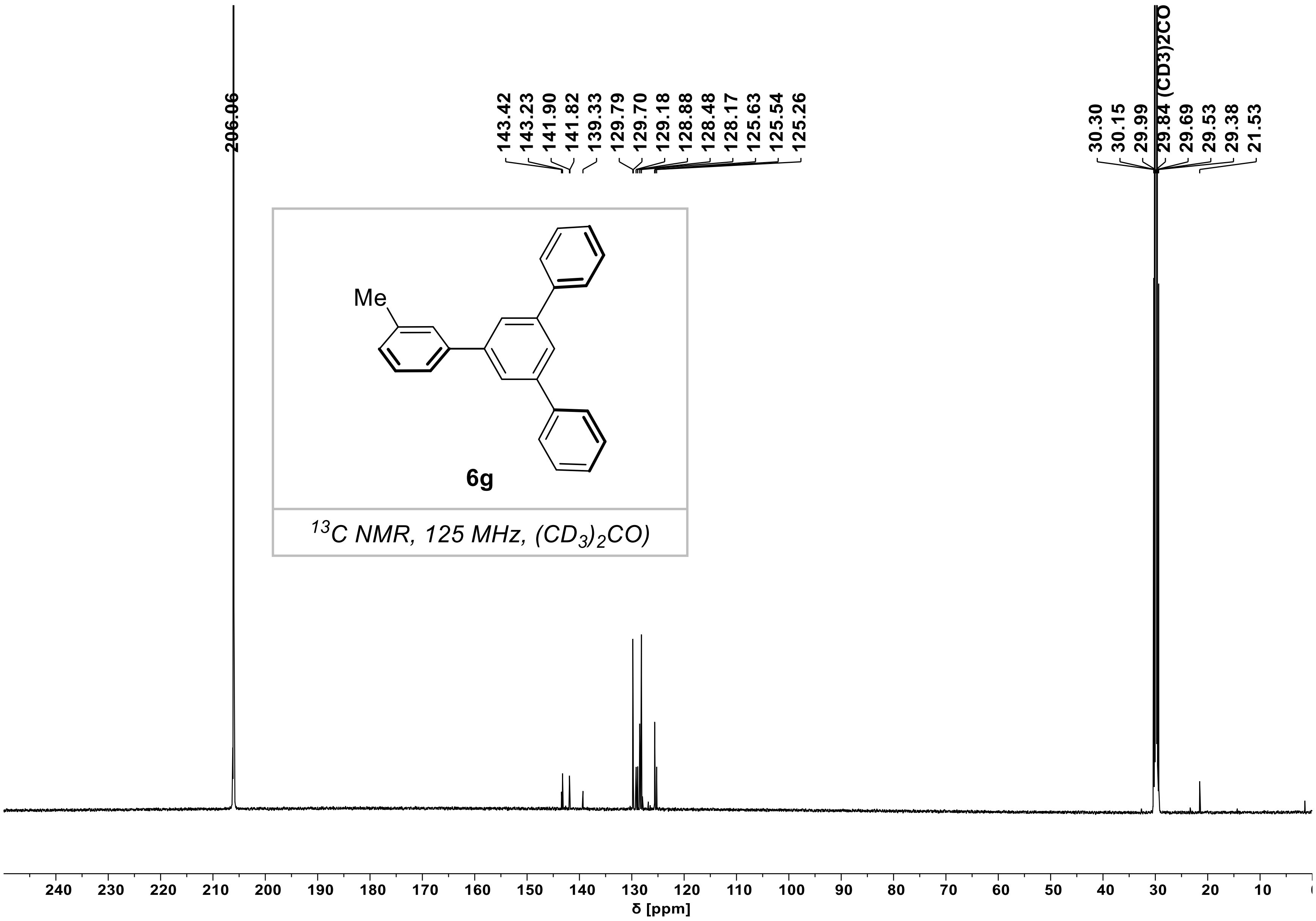
206.06
143.42
143.23
141.90
141.82
139.33
129.79
129.70
129.18
128.88
128.48
128.17
125.63
125.54
125.26
30.30
30.15
29.99
29.84 (CD3)2CO
29.69
29.53
29.38
21.53
Me
6g
^{13}C NMR, 125 MHz, $(CD_3)_2CO$
240
230
220
210
200
190
180
170
160
150
140
130
120
110
100
90
80
70
60
50
40
30
20
10
δ [ppm]

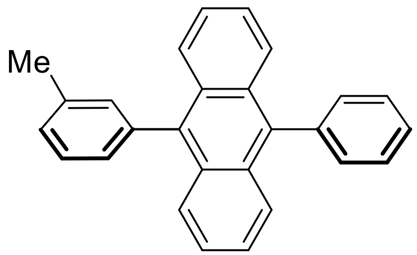

6h

^{1}H NMR, 400 MHz, $CDCl_3$

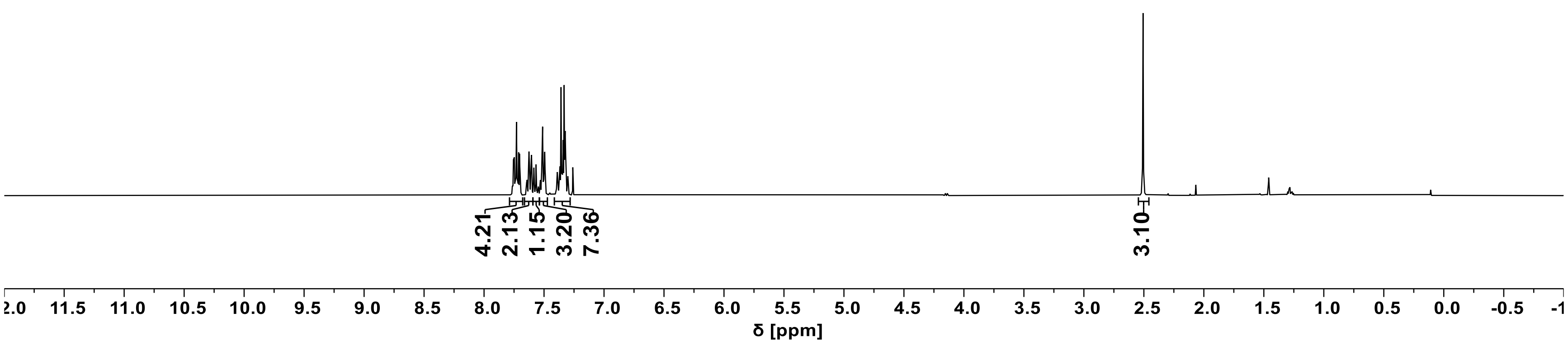

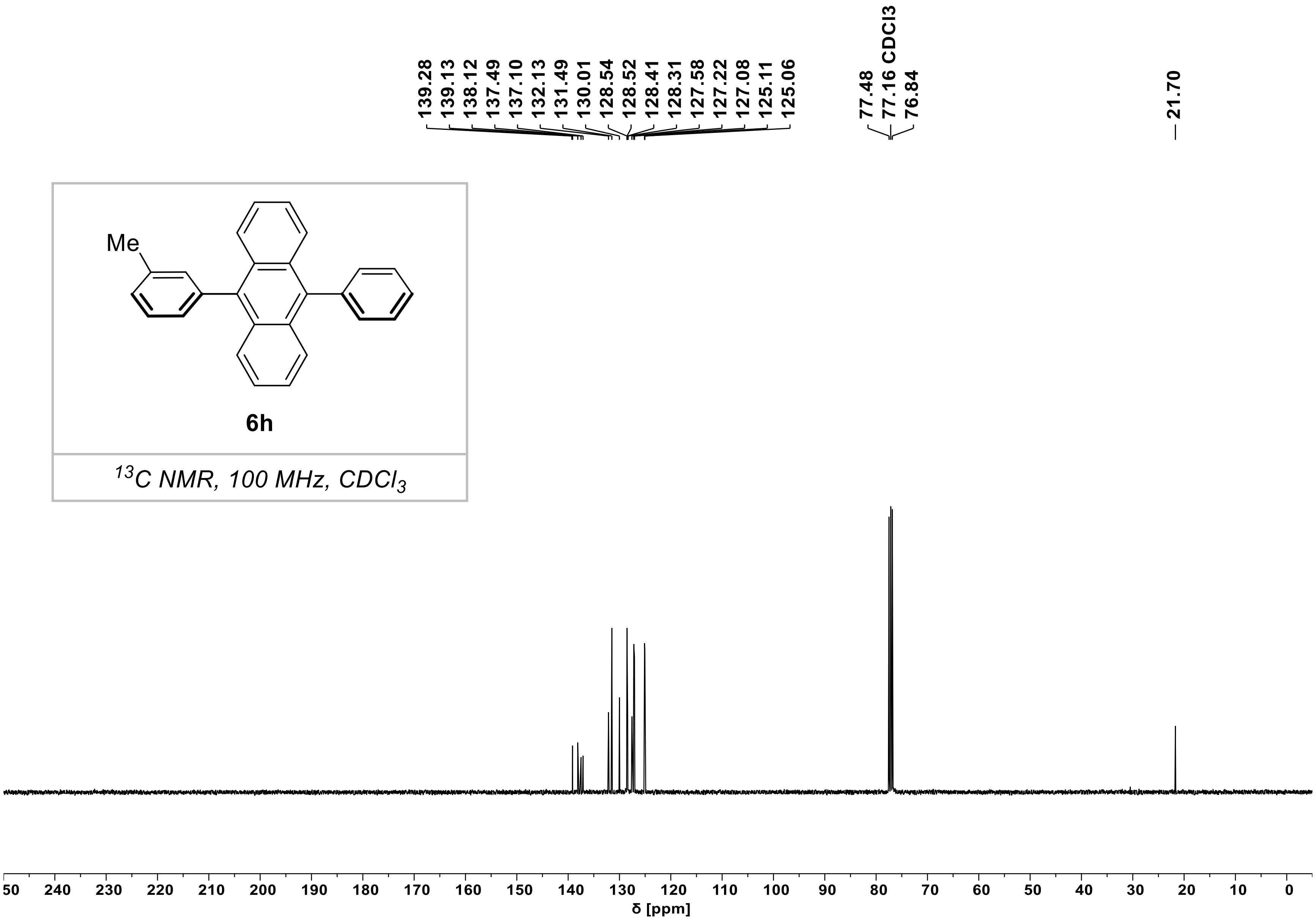
139.28
139.13
138.12
137.49
137.10
132.13
131.49
130.01
128.54
128.52
128.41
128.31
127.58
127.22
127.08
125.11
125.06
77.48
77.16 CDCl3
76.84
21.70
Me
6h
^{13}C NMR, 100 MHz, $CDCl_3$
50
240
230
220
210
200
190
180
170
160
150
140
130
120
110
100
90
80
70
60
50
40
30
20
10
0
δ [ppm]

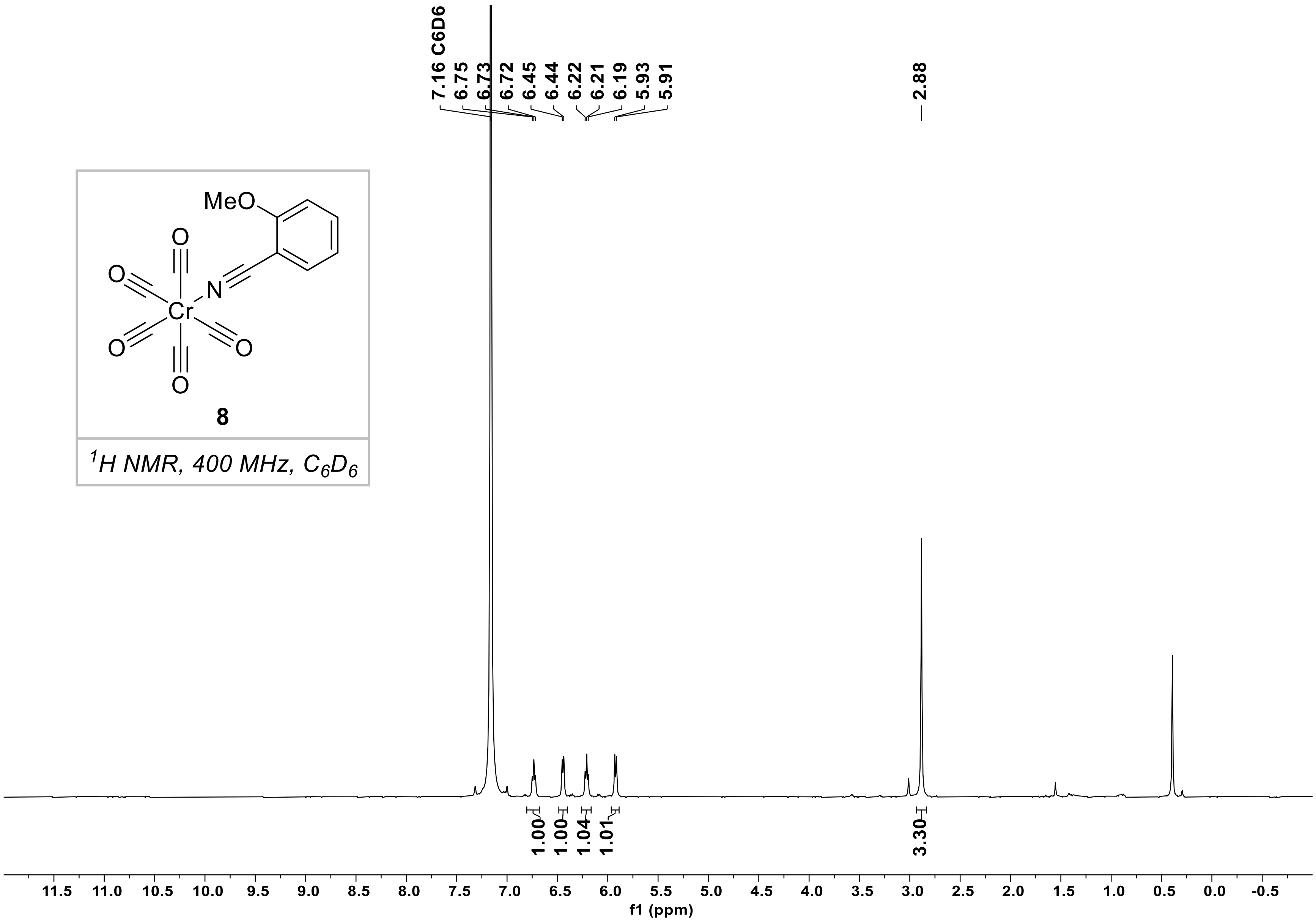
7.16 C6D6
6.75
6.73
6.72
6.45
6.44
6.22
6.21
6.19
5.93
5.91
2.88
MeO
O
O
N
Cr
O
O
O
8
1H NMR, 400 MHz, C6D6
1.00
1.00
1.04
1.01
3.30
11.5
11.0
10.5
10.0
9.5
9.0
8.5
8.0
7.5
7.0
6.5
6.0
5.5
5.0
4.5
4.0
3.5
3.0
2.5
2.0
1.5
1.0
0.5
0.0
-0.5
f1 (ppm)

5. References

[1] J. Koziskova, F. Hahn, J. Richter, J. Kožíšek, *Acta Chim. Slov.* **2016**, *9*, 136–140.

[2] SADABS-2016/2, Bruker AXS, 2016.

[3] J.-C. Boutonnet, J. Levisalles, F. Rose-Munch, E. Rose, G. Precigoux, F. Leroy, *J. Organomet. Chem.* **1985**, *290*, 153–164.

[4] Y. Tobe, A. Nakayama, K. Kobiro, K. Kakiuchi, Y. Odaira, *Chem. Lett.* **1989**, *18*, 1549–1550.

[5] F. van Meurs, J. M. van der Toorn, H. van Bekkum, *J. Organomet. Chem.* **1976**, *113*, 341–351.

[6] G. B. M. Kostermans, M. Bobeldijk, P. J. Kwakman, W. H. de Wolfand, F. Bickehaupt, *J. Organomet. Chem.* **1989**, *363*, 291–296.

[7] Hans G. Wey, P. Betz, H. Butenschön, *Chem. Ber.* **1991**, *124*, 465–474.

[8] a) B. Ohlsson, C. Ullenius, S. Jagner, C. Grivet, E. Wengner, E. P. Kundig, *J. Organomet. Chem.* **1989**, *365*, 243–267; b) R. C. Cambie, G. R. Clark, S. R. Gallagher, P. S. Rutledge, M. J. Stone, P. D. Woodgate, *J. Organomet. Chem.* **1988**, *342*, 315–337.

[9] S. J. Coote, S. G. Davies, D. Middlemiss, A. Naylor, *J. Organomet. Chem.* **1989**, *379*, 81−88.

[10] A. Meyer, *Annales de Chimie* **1973**, *8*, 397–404.

[11] W. McFarlane, S. O. Grim, *J. Organomet. Chem.* **1966**, *5*, 147–154.

[12] H.-G. Schmalz, O. Kiehl, B. Gotov, *Synlett* **2002**, *8*, 1253–1256.

[13] P. Ricci, K. Krämer, I. Larrosa, *J. Am. Chem. Soc.* **2014**, *136*, 18082–18086.

[14] R. Bigler, V. K. Aggarwal, *Angew. Chem. Int. Ed.* **2018**, *57*, 1082–1086.

[15] P. Ricci, K. Krämer, X. C. Cambeiro, I. Larrosa, *J. Am. Chem. Soc.* **2013**, *135*, 13258–13261.

[16] W.-L. Zeng , X. Jiang , W. Li, *Synlett* **2024**, *35*, 1101–1106.

[17] a) J. P. Gilday, J. T. Negri, D. A. Widdowson, *Tetrahedron* **1989**, *45*, 4605–4618; b) A. D. Hunter, V. Mozol, S. D. Tsai, *Organometallics* **1992**, *11*, 2251–2262.

[18] C.-H. Andersson, G. Berggren, S. Ott, H. Grennberg, *Eur. J. Inorg. Chem.* **2011**, 1744–1749.

[19] V. Gagliardini, V. Onnikian, F. Rose-Munch, E. Rose, *Inorg. Chim. Acta* **1997**, *259*, 265–271.

[20] R. J. Card, W. S. Trahanovsky, *J. Org. Chem.* **1980**, *45*, 2560–2566.

[21] F. Rose-Munch, E. Rose, A. Semra, L. Mignon, J. Garcia-Oricain, C. Knobler, *J. Organomet. Chem.* **1989**, *363*, 297–309.

[22] F. Rose-Munch, E. Rose, A. Semra, *J. Chem. Soc., Chem. Commun.* **1987**, 942–943.

[23] J. A. Heppert, M. A. Morgenstern, D. M. Scherubel, F. Takusagawa, M. R. Shaker, *Organometallics* **1988**, *7*, 1715–1723.

[24] a) D. Villemin, A. Jullien, N. Bar, *Tetrahedron Lett.* **2007**, *48*, 4191–4193; b) D. Prim, B. Andrioletti, F. Rose-Munch, E. Rose, F. Couty, *Tetrahedron* **2004**, *60*, 3325–3347; c) S. Maiorana, C. Baldoli, P. Del Buttero, M. Di Ciolo, A. Papagni, *Synthesis* **1998**, 735–738.
